# *LMNA* Knock-Down Affects Differentiation and Progression of Human Neuroblastoma Cells

**DOI:** 10.1371/journal.pone.0045513

**Published:** 2012-09-26

**Authors:** Giovanna Maresca, Manuela Natoli, Marta Nardella, Ivan Arisi, Daniela Trisciuoglio, Marianna Desideri, Rossella Brandi, Simona D’Aguanno, Maria Rita Nicotra, Mara D’Onofrio, Andrea Urbani, Pier Giorgio Natali, Donatella Del Bufalo, Armando Felsani, Igea D’Agnano

**Affiliations:** 1 CNR-Institute of Cell Biology and Neurobiology, Santa Lucia Foundation-IRCCS, Rome, Italy; 2 European Brain Research Institute, EBRI-Neurogenomics IIT Unit, Rome, Italy; 3 Experimental Chemotherapy Laboratory, Regina Elena National Cancer Institute, Rome, Italy; 4 Department of Internal Medicine, University of Tor Vergata, Laboratory of Proteomics, Santa Lucia Foundation-IRCCS, Rome, Italy; 5 Laboratory CINBO, University “G. d’Annunzio”, Chieti, Italy; Sun Yat-sen University Medical School, China

## Abstract

**Background:**

Neuroblastoma (NB) is one of the most aggressive tumors that occur in childhood. Although genes, such as *MYCN*, have been shown to be involved in the aggressiveness of the disease, the identification of new biological markers is still desirable. The induction of differentiation is one of the strategies used in the treatment of neuroblastoma. A-type lamins are components of the nuclear lamina and are involved in differentiation. We studied the role of Lamin A/C in the differentiation and progression of neuroblastoma.

**Methodology/Principal Findings:**

Knock-down of Lamin A/C (*LMNA*-KD) in neuroblastoma cells blocked retinoic acid-induced differentiation, preventing neurites outgrowth and the expression of neural markers. The genome-wide gene-expression profile and the proteomic analysis of *LMNA*-KD cells confirmed the inhibition of differentiation and demonstrated an increase of aggressiveness-related genes and molecules resulting in augmented migration/invasion, and increasing the drug resistance of the cells. The more aggressive phenotype acquired by *LMNA*-KD cells was also maintained *in vivo* after injection into nude mice. A preliminary immunohistochemistry analysis of Lamin A/C expression in nine primary stages human NB indicated that this protein is poorly expressed in most of these cases.

**Conclusions/Significance:**

We demonstrated for the first time in neuroblastoma cells that Lamin A/C plays a central role in the differentiation, and that the loss of this protein gave rise to a more aggressive tumor phenotype.

## Introduction

Neuroblastoma is an embryonic tumor of the sympathetic nervous system and is one of the most common cancers in childhood. The clinical presentation of neuroblastoma is extremely variable, ranging from benign to highly aggressive tumors [1]. *MYCN* amplification, which occurs in about 25% primary tumors, is considered to be the most important oncogenic marker in neuroblastoma, correlating with unfavorable prognosis [2]. Additionally, a high differentiation stage has been associated with a favorable outcome [3]. Nevertheless, the mechanisms governing neuroblastoma cell differentiation are not completely understood.

The ability of many neuroblastoma cell lines to differentiate and to present unique markers of neuronal differentiation in the presence of various agents has led to the use of these cell lines as model systems to study human neuronal differentiation [4], [5].

Lamins, type V intermediate filaments, are the major components of the nuclear lamina. They are divided into A and B types based on similarities in their primary sequence and biological properties. In mammals, two major A-type (Lamin A and C) and two major B-type (Lamin B1 and B2) lamins have been characterized. The most important and well-established function of nuclear lamins is to provide shape and mechanical stability to the nucleus [6]. Furthermore, several lines of evidence have supported a role for the lamins in maintaining the mechanical properties of the entire cell. Indeed, Lamin A/C deficiency causes impaired mechanotransduction and decreased mechanical stiffness [7].

**Figure 1:**
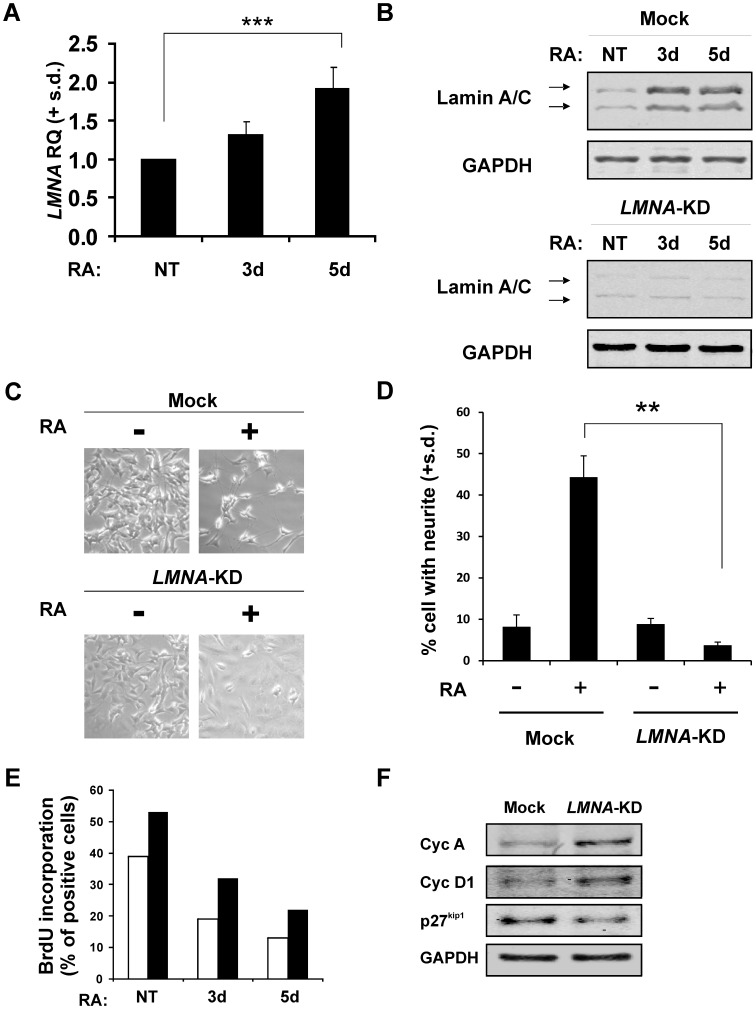
Silencing of *LMNA* in the SH-SY5Y cells during RA-induced differentiation. A: Real-time RT-PCR of *LMNA* mRNA in SH-SY5Y cells NT (not treated) or RA-treated for 3 and 5 days. Data are reported as the level of mRNA relative to NT cells and are the means + standard deviation (s.d.; n = 5). Statistical significance: ***p<0.001. **B:** Representative western blotting of Lamin A/C protein in Mock and *LMNA*-KD cells NT or RA-treated for 3d and 5d. The experiment was repeated three times showing similar results. **C:** Inverted light microscope images of Mock and *LMNA*-KD cells NT or RA-treated for 5d. Original magnification: 200X. **D:** Quantification of the percentage of Mock and *LMNA*-KD cells bearing neurite outgrowths NT or RA-treated for 5d. The data represent the means + s.d. (n = 3). Statistical significance: **p<0.01. **E:** Percent of BrdU positive cells as evaluated by FACS in Mock (white) and *LMNA*-KD (black) cells. The experiment was repeated three times with similar results. **F:** Representative western blotting of cell cycle related protein in Mock and *LMNA*-KD cells. The experiment was repeated three times showing similar results.

Recent models of nuclear architecture describe lamins as determining factor for chromosome positioning throughout the nucleus [8], [9], anchoring chromatin to the nuclear lamina and functioning as a nucleoplasmic scaffold for nuclear chromatin. Indeed, many aspects of nuclear activity, such as DNA replication and transcription, are affected by modifications of the nuclear lamina [10], [11].

The individual types of lamins are differentially expressed during development [12]–[14]. A role of these proteins in the differentiation processes has been demonstrated in the muscle and adipocyte differentiation [15], [16] Additionally, the expression of the A-type lamins is often reduced or absent in subsets of cells with a low degree of differentiation and/or cells that are highly proliferative, including various human malignancies [17]–[21], even though some authors have reported in colorectal tumors that A-type lamins may play a role as risk biomarker [22].

The main objective of this work was to investigate whether Lamin A/C is involved in neuroblastoma differentiation. Moreover, we studied a possible role of Lamin A/C in the progression of this neuronal cancer.

## Results

### *LMNA* Gene Knock-down Inhibits Retinoic Acid (RA)-induced Differentiation in Neuroblastoma Cells

We chose a neuroblastoma model that can differentiate *in vitro* and that has high expression level of Lamin A/C. As a differentiating stimulus, we used RA. Exposure of SH-SY5Y cells to RA increased *LMNA* gene expression (Figure 1A).

**Figure 2:**
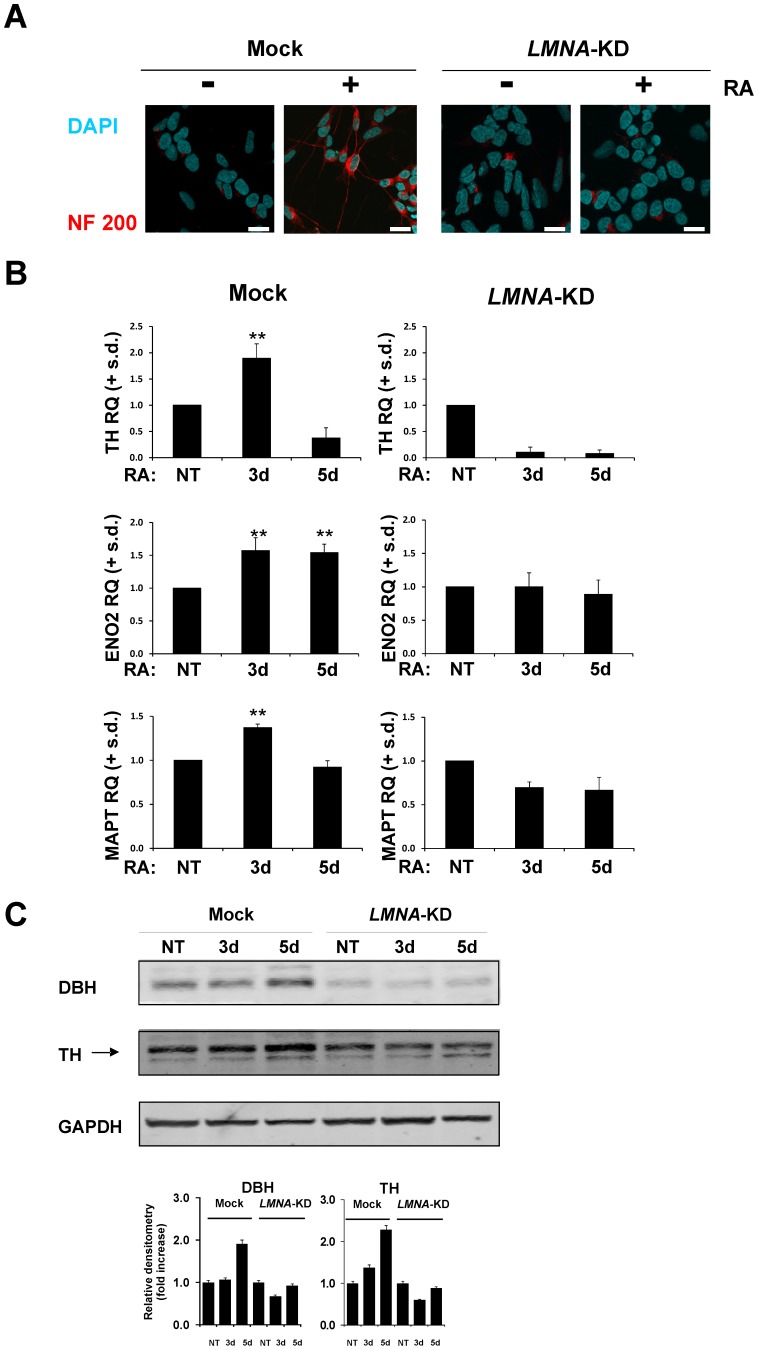
Silencing of *LMNA* impairs RA-induced differentiation in the SH-SY5Y cells. A: Representative confocal images of Mock and *LMNA*-KD cells NT or RA-treated for 5d. Red, NF200 immunostaining and blue, DAPI. Scale bar: 15 microns. **B:** Levels of the indicated mRNA in Mock and *LMNA*-KD cells NT or RA-treated for 3d and 5d. The data are reported as the level of mRNA relative to the respective NT cells and are the means + s.d. (n = 3). Statistical significance: **p<0.01. **C:** *Top*, representative blots of the indicated proteins in Mock and *LMNA*-KD cells NT or RA-treated for 3d and 5d. The experiment was repeated three times showing similar results; *bottom*, blots densitometry as analyzed by ImageJ software. Values are averages + s.d. (n = 3), relative to NT of three independent experiments with similar results.

We silenced the *LMNA* gene in this cell line and studied the effects on the differentiation processes. *LMNA* knock-down (KD) was obtained by infecting the cells with a lentiviral vector that allows the simultaneous expression of the EmGFP reporter gene and of an artificial miRNA targeting the *LMNA* mRNA. The *LMNA-*KD cells showed a reduction in the Lamin A/C protein of approximately 70% compared to Mock cells, infected with the same lentiviral vector carrying a non-targeting artificial EmGFP-miRNA (Figure 1B). The reduction of Lamin A/C expression resulted in a cell population exhibiting a flatter shape without significant neurite outgrowths, indicating an impairment of neuronal differentiation (Figure 1C and D). Consistent with the impairment of neuritis outgrowth is the increase of DNA synthesis as evaluated by BrdU incorporation during the differentiation process in *LMNA*-KD compared to Mock cells (Figure 1E). Additionally, changes in some cell cycle related molecules were also evidenced by an increase of cyclins A and D1, and a decrease of p27^kip1^ in *LMNA*-KD compared to Mock cells (Figure 1F).

**Figure 3:**
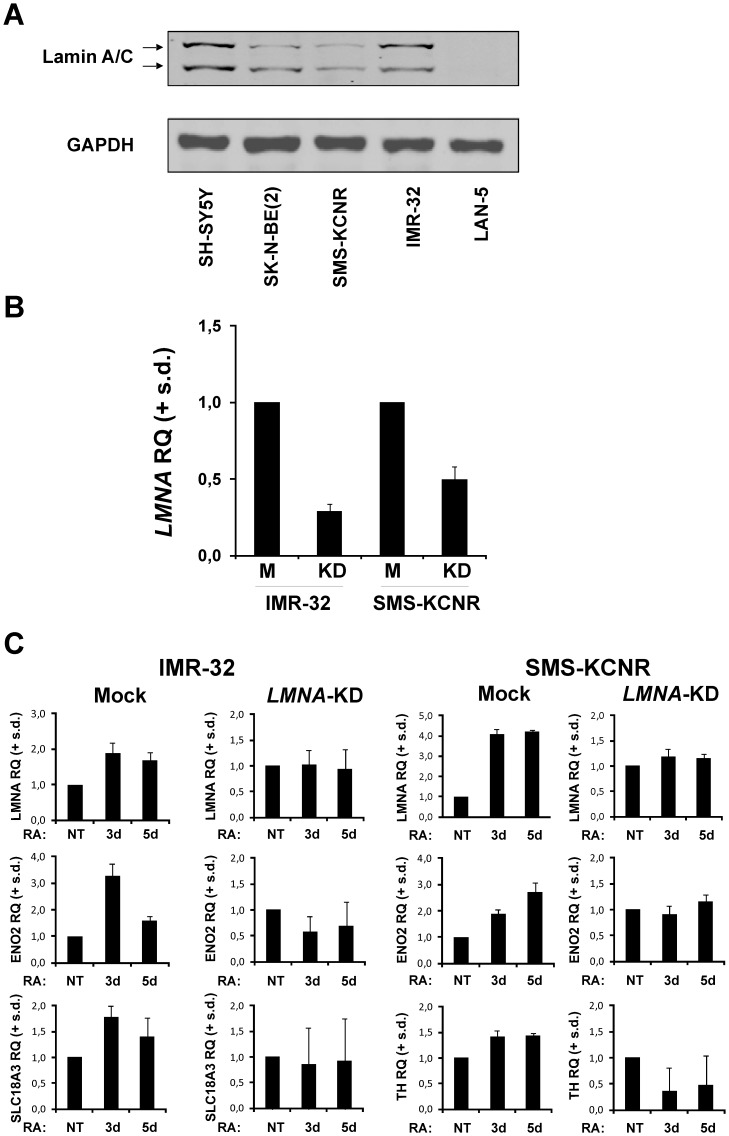
Silencing of *LMNA* impairs RA-induced differentiation in the IMR-32 and SMS-KCNR cell lines. A: Representative blot of lamin A/C expression in 5 different neuroblastoma cell lines. The experiment was repeated three times showing similar results. **B:** Levels of *LMNA* mRNA in Mock (M) and *LMNA*-KD (KD), IMR-32 and SMS-KCNR cell lines. The data are reported as the level of mRNA relative to the respective M cells and are the means + s.d. (n = 3). **C:** Levels of the indicated mRNA in Mock and *LMNA*-KD, IMR-32 and SMS-KCNR cells, NT or RA-treated for 3d and 5d. The data are reported as the level of mRNA relative to the respective NT cells and are the means + s.d. (n = 3).

Consistent with the impairment in neurite formation in *LMNA*-KD cells, we observed decreased induction of the primary neuronal differentiation markers (Figure 2). Figure 2A shows fluorescent images of the expression of neurofilament-200 (NF200) in *LMNA*-KD compared to Mock cells. The inhibition of the NF200 expression is evident in *LMNA-*KD cells. The impairment of differentiation in *LMNA-*KD cells was further confirmed by the loss of induction of some neuronal differentiation markers such as tyrosine hydroxylase (*TH*), dopamine-β-hydroxylase (*DBH*) and enolase 2 (*ENO2*), as evaluated by qRT-PCR (Figure 2B) and Western blot (TH and DBH, Figure 2C).

We have also studied the effect of *LMNA* knock-down in other two neuroblastoma cell lines, IMR-32 and SMS-KCNR. We chose these two cell lines because of their different expression of Lamin A/C protein (Figure 3A). We silenced *LMNA* gene and the level of gene knock-down for the two cell lines is shown in Figure 3B. We demonstrated that also in these cell lines silencing of *LMNA* gene resulted in the inhibition of cell differentiation as evidenced by the loss of induction of the indicated markers after 3 and 5 days of differentiation (Figure 3C).

**Figure 4:**
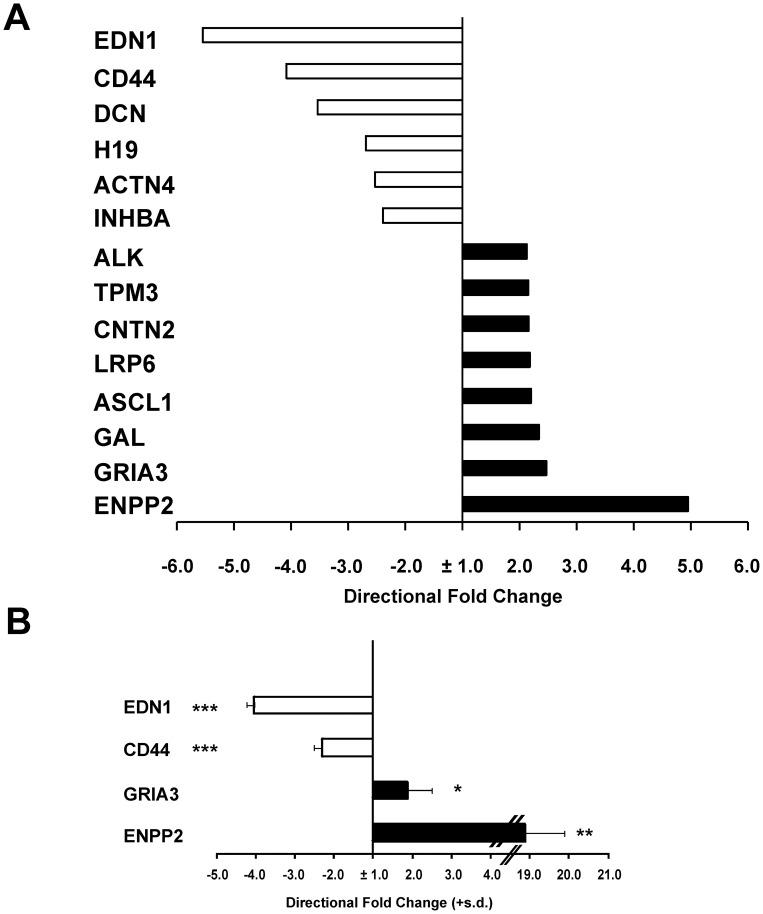
Relative expression of tumor aggressiveness-related genes in *LMNA*-KD *versus* Mock cells. A: Directional fold-change values for a subset of gene, related to tumor aggressiveness identified in the microarray experiment as either up-regulated (black) or down-regulated (white), upon comparison of *LMNA*-KD *versus* Mock cells. The gene symbols are indicated on the left (Table S3). The fold-change (fc) values are on a linear scale. To visualize the changes relative to the down-regulated genes, the fc values of the down-regulated genes were converted into negative values using the formula (−1/fc). **B:** Levels of the indicated mRNA in *LMNA*-KD *versus* Mock cells. The data are reported as the directional fold-change values calculated as above and are the means + s.d. (n = 3). Statistical significance: *p<0.05; **p<0.01; ***p<0.001.

### Gene Expression Profiling of *LMNA*-KD Cells Confirms an Impairment of Differentiation and Evidences the Establishment of a More Aggressive Phenotype

We compared the gene expression profile of Mock and *LMNA-*KD cells, both in RA-treated and untreated cells, using Agilent microarray technology. We verified that the *LMNA* expression was induced by RA in Mock cells (approximately 1.6 folds), while the silencing effect in *LMNA*-KD cells was 0.2 fold. We identified a set of 1320 genes differentially expressed at least 2.0-fold by RA in both Mock and *LMNA-*KD cells. We defined two sets of genes that were obtained by comparing the RA-treated samples with the untreated samples in the Mock (Mock_RAvsNT_), or *LMNA-*KD (*LMNA-*KD_RAvsNT_) cells. The Venn diagram in Figure S1 shows that 1741 genes are modulated by RA only in Mock cells (1519 up-regulated and 222 down-regulated genes), while 769 are modulated only in *LMNA-*KD cells (499 up-regulated and 270 down-regulated genes).

**Figure 5:**
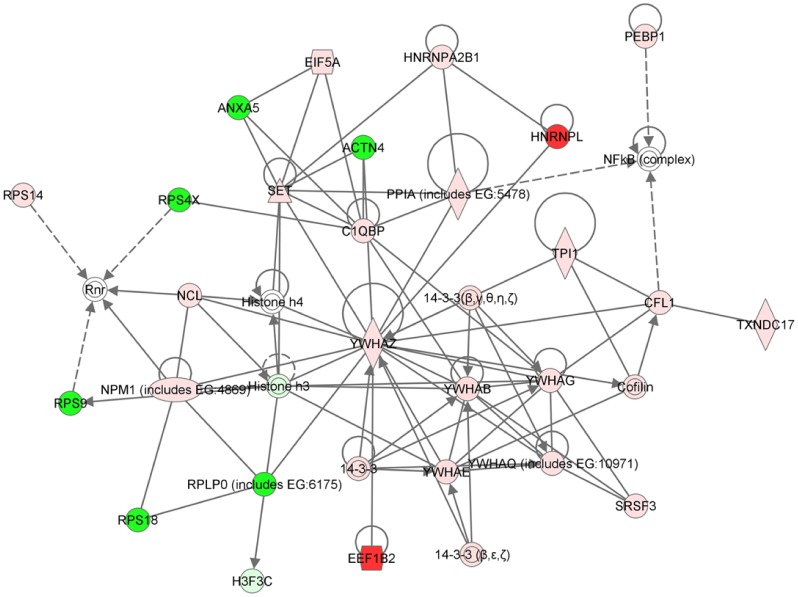
Pathway analyses. The network with highest score (*67*) is shown. The main network associated functions are: *Protein Trafficking, Cellular Assembly and Organization, DNA Replication, Recombination, and Repair*. The nodes represent proteins (Table S7): the coloured features indicate proteins identified (green  =  down-regulated and red  =  up-regulated in *LMNA-*KD compared to Mock), whereas the un-coloured features indicate additional members of this network that were not detected by the proteomic analysis. The node shapes indicate function: enzymes (diamonds), transcription regulators (ovals), nuclear receptors (rectangles), cytokines (squares), transporter (trapezoids), and “other” (circles). Protein–protein associations are indicated by edges containing single lines, whereas proteins acting upon another protein (controlling their expression) are indicated by arrows. Continuous or dotted lines indicate direct or indirect protein interactions, respectively.

The functional analysis of the differentially expressed genes, performed using the DAVID web tool, evidenced significant modulation (*p*<0.05) of functional categories in the Mock cells which includes categories related to neuron differentiation among the up-regulated genes and to cell cycle among the down-regulated genes (Table S1). Similarly, the categories modulated by RA only in *LMNA-*KD cells include categories related to cell migration for the up-regulated genes and to neuron differentiation for the down-regulated genes (Table S2).

We examined whether the impairment of cell differentiation that occurs in *LMNA*-KD cells is associated with a more aggressive phenotype. We defined a list of genes associated with specific cancer features using the Cancer Gene Index database of the NCI. Analyzing the *LMNA-*KD *versus* Mock cells whole gene expression profiles using the gene annotations of Cancer Gene Index, we identified 14 genes (eight up-regulated and six down-regulated) of which differential expression is related to a more aggressive phenotype. Among these, we found some tumor suppressor genes (e.g. *H19*, *ACTN4*) that were down-regulated and some genes related to tumor progression and metastasis formation (e.g. *ENPP2, GRIA3, TPM3*) that were up-regulated (Figure 4A and Table S3). The expression levels of the four most modulated genes (up- or down-regulated) were confirmed by qRT-PCR (Figure 4B). In addition, “cancer” was the top category found, even when the list of differentially modulated genes between Mock and *LMNA-*KD cells were analyzed by Ingenuity Pathway Analysis (IPA) (368 genes, 5.38×10^−43^<*p*<9.63×10^−6^). In this analysis (Table S4) 184 modulated genes were included in the “malignant tumor” function (*p* = 2.16×10^−21^), while 116 genes were associated with a “cell movement” function (*p = *5.89×10^−3^).

**Figure 6:**
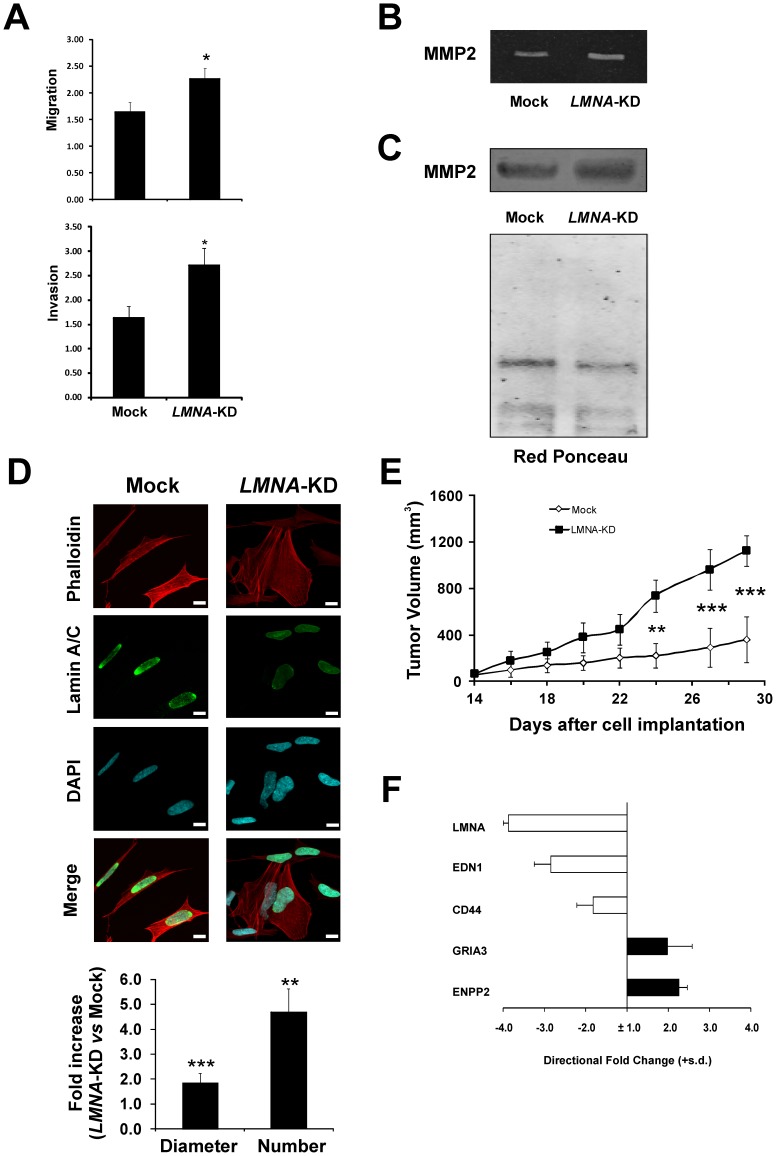
Silencing of *LMNA* induces a more aggressive tumor phenotype in SH-SY5Y neuroblastoma cells. A: Migration and Invasion assays. CM, conditioned medium. The data are reported as the fold-increase in migration/invasion relative to control and are the means + s.d. (n = 3). Statistical significance: *p<0.05. **B:** Representative zymographic analysis of matrix metalloprotease secretion. The experiment was repeated three times showing similar results. **C:** Western blot of MMP2 protein in CM from Mock and *LMNA*-KD cell cultures. Red Ponceau staining of the filter was used to confirm equal loading of proteins. A representative blot of three independent experiments is shown. **D:** Representative confocal images of Mock and *LMNA*-KD cells. Green, Lamin A/C immunostaining; red, F-actin; blue, DAPI. Scale bar: 10 microns. It is worthwhile to note that nuclear shapes are more irregular in *LMNA*-KD cells. In the bottom panels are reported the quantitative analysis of the fibers’ diameter and number in the cells. Statistical significance: **p<0.01; ***p<0.001. **E:** *In vivo* tumor growth of Mock and *LMNA*-KD xenogarfts. The means + s.d. of tumor volume of a representative experiment out of two is reported. Statistical significance: **p<0.01; ***p<0.001. **F:** Levels of the indicated mRNA in tumor samples obtained by injection of *LMNA*-KD cells in nude mice relative to tumor samples obtained by injection of Mock cells. The data are reported as the directional fold-change values calculated as in figure 3 and are the means (+ s.d.) of five tumor samples.

### Comparative Proteome Profiling by Label-free LC-MS^e^ of *LMNA*-KD and Mock SH-SY5Y Cells

To validate also at protein level the results obtained in the microarray experiments, a comparative proteome profiling of Mock and *LMNA-*KD cells was performed. A total of 442 proteins were qualitatively identified across both conditions. Only proteins identified in at least two out of three injections with a fold-change greater than 1.3 were considered in the subsequent analysis. Applying these filtering criteria, a total of 68 significant, differentially expressed proteins were selected (Table S5 and S6). In order to highlight the possible key candidates responsible for the different cellular properties of Mock and *LMNA-*KD cells, we performed an unsupervised bioinformatic analysis using the proteomic dataset of modulated proteins. The complete list of modulated proteins was loaded into IPA software and analysis was performed without any pre-established criteria of inclusion or exclusion. Data investigation revealed a muddled association of interactions among identified proteins and their direct or indirect interactors.

**Figure 7:**
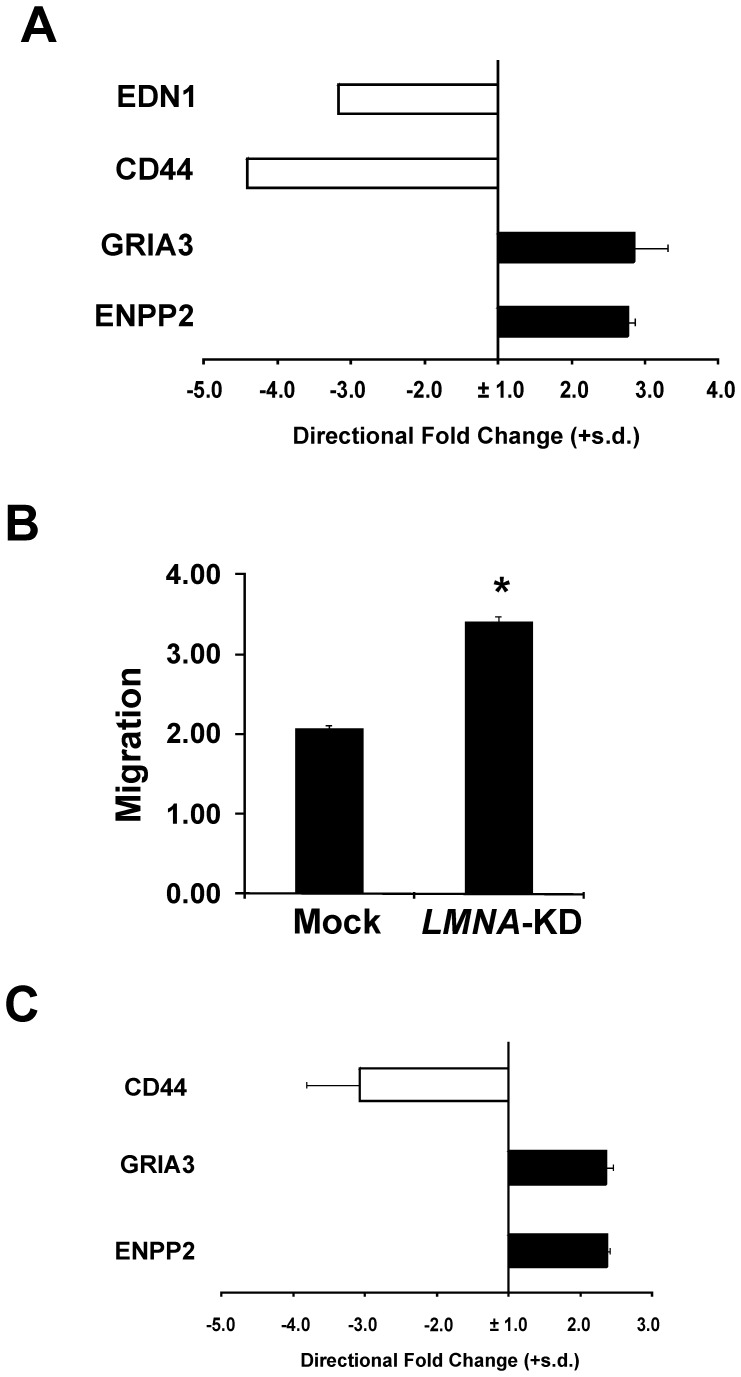
Silencing of *LMNA* induces a more aggressive tumor phenotype in IMR-32 and SMS-KCNR neuroblastoma cells. A: Relative expression of tumor aggressiveness-related genes in *LMNA*-KD *versus* Mock IMR-32 cells. Data are shown as directional fold-change values either up-regulated (black) or down-regulated (white). The gene symbols are indicated on the left (see Table S3 for specification). The fold-change (fc) values are on a linear scale and are the means + s.d. (n = 3), calculated as in figure 4. **B:** Migration assay in Mock and *LMNA*-KD IMR-32 cells as performed in figure 6. The data are reported as the fold-increase in migration relative to control and are the means + s.d. (n = 3). Statistical significance: *p<0.05. **C:** Relative expression of tumor aggressiveness-related genes in *LMNA*-KD *versus* Mock SMS-KCNR cells. Data, analyzed as above, are shown as directional fold-change values either up-regulated (black) or down-regulated (white). The gene symbols are indicated on the left. The fold-change (fc) values are on a linear scale and are the means + s.d. (n = 3), calculated as above.

The resulting network with the highest score (*67*) is shown in Figure 5 (See Table S7 for proteins specifications). Among the most modulated proteins, we noted the up-regulation of the heterogeneous nuclear ribonucleoprotein L (HNRPNL) and the down-regulation of the alpha-actinin 4 (ACTN4). The expression changes of these two proteins correlate with a similar modulation at the RNA level (*LMNA*-KD/Mock *HNRPNL* expression ratio, 1.4; *ACTN4* expression ratio, 0.4).

Additionally, members of 14-3-3 scaffold proteins family such as β, γ and ζ were found to be slightly increased in *LMNA-*silenced cells. The changes in expression of these proteins correlate with a similar modulation at the RNA level (*LMNA*-KD/Mock *YWHA* gene expression ratio, 1.4).

**Figure 8:**
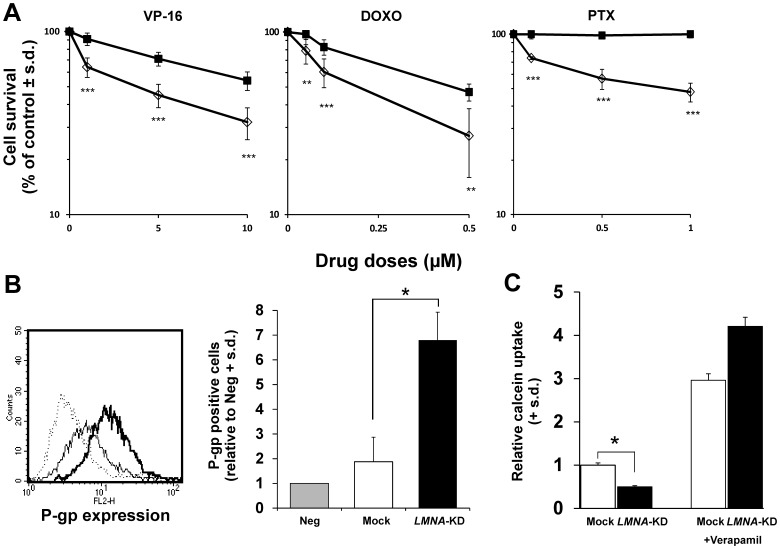
Reduced Lamin A/C expression resulted in increased drug resistance in SH-SY5Y cells. **A:** Effect of etoposide (VP-16), doxorubicin (DOXO) or paclitaxel (PTX) in Mock (open diamond) and *LMNA*-KD (black square) cells. The cells were treated for 2 hours with different doses of each drug. Cell survival data are reported as the percent of control and are the means ± s.d. (n = 3). Statistical differences between the *LMNA*-KD *versus* the Mock groups are indicated as follows: **p<0.01; ***p<0.001. **B:** FACS analysis of P-gp expression. *Left panel*: dotted line, negative control (Neg, mouse IgG), standard line (Mock) and bold line *(LMNA*-KD). *Right panel*: P-gp positive cells relative to Neg. **C:** P-gp functionality using the calcein assay. The mean fluorescence values for calcein in each sample were compared to the respective control. Verapamil was used as competitive inhibitor of P-gp function. The data are the means + s.d. (n = 3). Statistical significance: *p<0.05.

### The Knock-down of *LMNA* Increases Cell Motility and Invasion *in vitro*, and the Tumorigenicity *in vivo*

We examined the motility and the invasion ability of Mock and *LMNA-*KD cells in response to the conditioned medium of NIH3T3 cells (Figure 6A). The migratory ability was enhanced in *LMNA*-KD (approximately 1.5-fold) compared to Mock cells. Similarly, the ability to invade matrigel-coated filters was enhanced in *LMNA-*KD (approximately 1.7-fold).

We then examined how *LMNA* silencing could affect the expression and activity of matrix metalloproteases (MMPs). A zymographic analysis of the conditioned medium from Mock and *LMNA*-KD cells showed an increase in the secretion of the gelatinase 72-kDa MMP2 after *LMNA* silencing (Figure 6B) compared to Mock cells. A western blot analysis of MMP2 expression in the same samples, also demonstrated an increased expression of MMP2 protein by approximately 1.6-fold in *LMNA* silenced compared to Mock cells (Figure 6C). Consistent with these results is the increased expression of MMP2 protein in the lysates of the Mock and *LMNA*-KD cells from the same experiment (Figure S2). The activity of MMP9 was undetectable in our experimental conditions.

Because cell invasion involves cytoskeleton rearrangements, we studied the intracellular organization of actin filaments by staining actin with phalloidin (Figure 6D). Knock-down of *LMNA* increased the number and tickness of cytoplasmic actin-containing fibers. Noteworthy, this effect was particularly evident in the *LMNA*-KD cells showing the lowest staining for Lamin A/C.

To evaluate whether *LMNA* silencing can affect tumorigenesis, *LMNA-*KD and Mock cells were injected in nude mice and tumor weight was monitored. *LMNA*-KD tumors grew significantly faster than Mock tumors, the volume of *LMNA*-KD tumors being about 4-fold increased when compared to Mock tumors at day 29 after implantation (p<0.001), thus demonstrating an increased aggressiveness after reduction of *LMNA* expression (Figure 6E). The expression levels of the most modulated genes related to tumor aggressiveness, previously identified in the microarray analysis, were evaluated in Mock and *LMNA*-KD xenografts, showing that the expression levels of these aggressiveness-related genes were similar to those obtained in the *in vitro* analyses (Figure 6F).

We analyzed the effect of the knock-down of *LMNA* also in the IMR-32 and SMS-KCNR neuroblastoma cell lines. In Fig. 7A and C are reported the changes of the expression levels (up- or down-regulated) of the genes which were related to a more aggressive phenotype in the SH-SY5Y cells, evaluated by qRT-PCR in the IMR-32 and SMS-KCNR, Mock and *LMNA*-KD cells. We found a similar behavior of the indicated genes also in these neuroblastoma cell lines compared to the SH-SY5Y cells. *EDN1* gene was found not expressed in the SMS-KCNR cell line. In Fig. 7B is shown that the IMR-32 *LMNA-*KD cells have an enhanced ability to invade matrigel-coated filters (approximately 1.6-fold).

### *In vivo* Expression of Lamin A/C in Early Stage NB

Nine primary, therapy-free, early stage (I and II) neuroblastomas (NB) were obtained upon informed parents’ or guardian’s consent [23]. Tumours were classified according to the International Neuroblastoma Pathology Classification (INPC) [24] Lamin A/C specific nuclear stain was observed in scattered interstitial cells and in some vessels in eight out of the nine tumours analyzed. On the contrary neuroblastoma cells displayed very rarely nuclear stain. Only in two cases, namely a stage I and a stage II lesion, scattered areas displayed nuclear stain of the tumor cells.

### Reduced Lamin A/C Expression is Associated with Increased Drug Resistance

We studied the effects of different doses of etoposide (VP-16), doxorubicin (DOXO) and paclitaxel (PTX) on the cell survival of the Mock and *LMNA-*KD cells. The silencing of *LMNA* resulted in a significant increase in cell survival for all of the compounds employed (Figure 8A). Because the three drugs are all substrates of the P-glycoprotein, we also studied whether *LMNA* knock-down affects the expression and/or function of this membrane pump. Indeed, the expression of the P-gp protein was increased by approximately 3.4-fold in *LMNA*-KD compared to Mock cells (Figure 8B). In addition, the activity of the pump was analyzed by using a functional assay which employs as probe the fluorescent dye precursor calcein-acetoxymethylester (calcein AM). This precursor is an excellent substrate of the P-gp transporter and therefore is suitable as model to determine *in vitro* drug resistance of cells, to screen transporter substrates, and to monitor the P-gp mediated efflux of drugs which are substrate of the pump. As shown in Fig. 8C the P-gp activity was decreased by approximately 50% in LMNA-KD compared to Mock cells. The P-gp pump inhibitor Verapamil was used as control to completely block the activity of the pump in both conditions.

## Discussion

Our results demonstrate for the first time in neuroblastoma cells that Lamin A/C expression was necessary for differentiation and the loss of this protein gave rise to a more aggressive tumor phenotype.

In agreement with studies on the development of mammals as well as *Xenopus* and *Drosophila,* which showed that the nuclear lamina composition correlates with cell differentiation[14], [25]–[27], we showed that Lamin A/C expression increases in three different neuroblastoma cell lines during the differentiation program induced by retinoic acid.

Silencing the *LMNA* gene inhibited the differentiation program as first evidenced by the reduced ability of the cells to form mature neurite-networks and to increase their proliferative potential. The impairment of the differentiation program was further confirmed by the decreased levels of several known differentiation markers such as the proteins NF200, TH, DBH, and the genes *ENO2* and *MAPT*. The genome-wide gene expression profile data also confirmed the inhibition of cell differentiation observed in *LMNA-*silenced cells. Noteworthy, the number of RA induced genes is considerably lower in *LMNA*-KD compared to the Mock cells. Indeed, the cell cycle-related genes down-regulated in Mock cells to favor differentiation, were not modulated in *LMNA*-KD cells. Notably, the signaling pathway engaged by RA is still functional in these cells since the expression of the RA receptors as well as of the retinoid-binding proteins [28], is not changed in *LMNA*-KD cells (data not shown; see the microarray data deposited in the Gene Expression Omnibus (GEO) repository, accession GSE30677).

Analyzing the subset of *LMNA*-KD genes that is the most diagnostic of the differences between untreated and RA-treated samples we demonstrated an increased expression of genes related to tumor aggressiveness and not to differentiation, as would have been expected given the differentiation stimulus. Indeed, focusing on the samples not induced to differentiate, we clearly demonstrate that Lamin A/C contributes to the maintenance of normal cellular processes, highlighting the up-regulation of genes related to cancer progression and the down-regulation of some tumor suppressor genes in the *LMNA*-KD cells. Specifically, we observed the down-regulation of the *ACTN4* gene, which was also confirmed by the proteomic analysis. Even though the roles of ACTN4 in tumors reported in the literature are contradictory [29], [30] our data are in agreement with those of authors who demonstrated the role of *ACTN4* gene in suppressing tumorigenicity in different tumor types [31]–[33]. Additionally, our data, showing the increase of some members of the 14-3-3 protein family, are in agreement with those of other authors, who demonstrated a positive role of such proteins in the induction of oncogenic transformation and in promoting cancer cell survival [34], [35].

We demonstrated that alterations in the expression of genes involved in tumor aggressiveness resulted in the augmented ability of the cells to migrate increasing their proteolytic activity. Cell migration is a dynamic process that involves cyclical cytoskeletal rearrangements, and the formation of actin stress fibers could be responsible for changing the physical location of the cell and increasing its migratory ability [36]. Indeed, an increased presence of actin filament bundles (stress fibers), particularly at the periphery of the cell, was evident. Our data are in contrast with those of Lee *et al*. obtained in mouse embryonic fibroblasts showing that Lamin A/C deficiency reduces the speed of cell migration [37]. However, the assay and the model used to analyze cell migration is evidently different from that we used in this work. The more aggressive phenotype of the *LMNA*-KD cells was also demonstrated *in vivo* since tumors grown from injected *LMNA*-KD cells were significantly more tumorigenic than their control counterparts and exhibited the same modifications in gene expression previously observed *in vitro.*

The increased expression of the HNRNPL protein is consistent with an impairment of the nuclear pore complex activity (data not shown). HNRNPs are shuttling proteins that participate in the export of mature mRNA from the nucleus to the cytoplasm [38]. It is possible that the increase in the HNRNPL protein represents an attempt of the cells to compensate for the nucleo-cytoplasm transport altered by the loss of Lamin A/C. A-type lamins have also been reported to function as nucleoplasmic scaffolds anchoring heterochromatin to the nuclear lamina, possibly intervening in the epigenetic regulation of gene transcription [8], [9], [39], [40]. It is possible that the reduced expression of Lamin A/C disrupts the peripheral organization of the transcriptionally silent chromatin thus allowing some of the genes previously repressed to be transcribed. Indeed, Shevelyov *et al.* [41] demonstrated that the transcriptional activation of the lamina-bound testis-specific gene cluster in the male germ line is coupled with its translocation away from the nuclear envelope, thus corroborating our hypothesis.

We demonstrated increased expression levels and activity of the P-gp as indicated by FACS analyses in the *LMNA*-KD cells. This could be responsible for increased resistance of the *LMNA*-KD cells toward substrates of the efflux pump P-gp. However, no modification in the expression of genes coding for P-gp was observed, strongly indicating that a post-transcriptional event regulated the expression of this protein as recently reported by others [42].

Importantly, our preliminary data on the expression of Lamin A/C in NB tumor samples show that this protein is poorly expressed in most of the first stages cases analyzed, suggesting that an accurate analysis of Lamin A/C expression in early stage NB could provide important information on the tumor development.

In conclusion, our data demonstrate that Lamin A/C is required for the differentiation program of neuroblastoma cells. In addition, our data strongly suggest that low levels of Lamin A/C could favor a more aggressive tumor phenotype. The characterization of neuroblastoma tumors based on A-type lamin expression may help to develop rational therapeutic protocols based on the molecular profile of the individual tumor.

## Materials and Methods

### Ethics Statement

All procedures involving animals and their care were authorized and certified by the decree n. 67/97A of the Italian Minister of Health and protocol 2560/97 of the Rome Health Service Unit (ASL – RMB).

### Cell Line Maintenance, Differentiation and Treatment

The three human malignant neuroblastoma (SH-SY5Y, IMR-32 and SMS-KCNR) cell lines were purchased from the American Type Culture Collection (ATCC) in November 2007 and the cells maintained the characteristics of neuroblastoma and the ability to differentiate *in vitro*. SH-SY5Y and IMR-32 cells were grown in a 1∶1 mixture of Eagle’s Minimum Essential Medium and F12 medium (Gibco) or Eagle’s Minimum Essential Medium, respectively, supplemented with 0.5% non-essential amino acids, 0.5% sodium pyruvate. SMS-KCNR cells were grown in RPMI 1640. All the mediums were supplemented with 10% fetal bovine serum (FBS, Hyclone), 2 mM L-glutamine and 1% penicillin and streptomycin and the cells maintained in a fully-humidified incubator containing 5% CO_2_ at 37°C. For all the differentiation experiments, SH-SY5Y and SMS-KCNR cells were seeded at a density of 5×10^3^ cells/cm^2^. The following day cells were induced to differentiate by 10 µM all-trans retinoic acid (ATRA, named RA throughout the paper; Sigma Chemical) in a “differentiation medium” (DM, composed of 50% fresh and 50% conditioned culture medium). For the differentiation experiments, IMR-32 cells were seeded at a density of 1.5×10^3^ cells/cm^2^, the following day cells were induced to differentiate by 10 µM RA (Sigma Chemical) in a DM composed of medium plus 1% FBS. The cells were fed with DM containing fresh RA after 3 days. RA was dissolved in dimethyl sulfoxide (DMSO, Sigma Chemical). The concentration of DMSO in each experiment was always ≤0.01%, which was not toxic and did not induce differentiation. For the drug experiments, the SH-SY5Y cells were seeded at a density of 5×10^3^ cells/cm^2^. Twenty-four hours after plating, fresh medium was added, and after another 24 hours (48 hours of growth), the cells were treated for 2 hours with different doses of etoposide (VP-16) or paclitaxel (PTX), both dissolved in DMSO, or doxorubicin (DOXO), dissolved in water.

### Generation of Lentiviral Vectors

The *LMNA*-knockdown SH-SY5Y, SMS-KCNR and IMR-32 cell lines were generated by using a lentiviral expression vector containing a double-strand oligo encoding a pre-miRNA sequence as the “knockdown cassette”, thus enabling the expression of an engineered miRNA sequence directed against the *LMNA* mRNA. The lentiviral expression vector was produced using the BLOCK-iT Lentiviral Pol II miR RNAi Expression System (Invitrogen) to obtain a Gateway®-adapted lentiviral destination vector named pLenti6/V5-GW/EmGFP-miR-*LMNA* from pcDNA6.2-GW/EmGFP-miR-*LMNA* (Invitrogen), according to the manufacturer’s instructions. As a negative control, we generated the pLenti6/V5-GW/EmGFP-miR-Neg control plasmid from the pcDNA™6.2-GW/EmGFP-miR-Neg control plasmid (Invitrogen), which contains an insert that can form a hairpin structure that is processed into mature miRNA but is not predicted to target any known vertebrate gene.

### Generation of Recombinant Viruses and Lentiviral Infection of Cells

To produce lentiviral stocks, the lentiviral vector (3 µg) and the ViraPower™ Packaging Mix (9 µg) were cotransfected into 6×10^6^ packaging 293FT cells using Lipofectamine 2000 (Invitrogen), according to the manufacturer’s instructions. The supernatants were collected after 48 and 72 hours, and were used for infection. The cells were seeded into 6-well plates at a concentration of 2×10^5^ cells/well. The following day, the cells were infected for 18 hours with the virus-containing supernatant from pLenti6/V5-GW/EmGFP-miR-*LMNA* or pLenti6/V5-GW/EmGFP-miR-Neg, and then fresh medium was added. After 6 hours, the cells were subjected to a second round of infection for an additional 18 hours. Stably transduced cells were selected for by culturing the cells in the presence of blasticidin at 3 µg/mL (Invitrogen). The transduced cells were screened using western blotting and real-time RT-PCR assays to determine the levels of *LMNA* expression and the miRNA expression was monitored by checking the simultaneous coexpression of the EmGFP reporter gene by fluorescence microscopy.

### Animal Experiments

Female CD-1 nude (nu/nu) mice were purchased from Charles River Laboratories (Calco). All procedures involving animals and their care were authorized and certified by the decree n. 67/97A of the Italian Minister of Health and protocol 2560/97 of the Rome Health Service Unit (ASL – RMB). Each experimental group included 8 mice. Cells in the exponential growth phase were harvested from the culture, washed with medium and resuspended in Matrigel (2.5 mg/ml; BD Biosciences) and 10^7^ cells implanted subcutaneously in the dorsal region of the mice. The tumor weight was calculated as previously reported [43]. Two different experiments were performed.

### Tissues Immunohistochemistry

Nine primary, therapy-free, early stage (I and II) neuroblastomas (NB) were obtained upon informed parents’ or guardian’s consent [23]. Tumours were classified according to the International Neuroblastoma Pathology Classification (INPC) [24]. Upon collection specimens were snap frozen in liquid nitrogen. Cryostat sections (4 µm thick) were fixed in cold absolute acetone and stained by indirect immunoperoxidase method using the murine monoclonal antibody (clone JOL2) to lamin A/C (Chemicon International) according to the manufacturer’s instruction. Staining was revealed by a supersensitive immunohistochemistry kit as described [44]. Positive and negative controls of the stain were acetone fixed cytospins of Mock and *LMNA*-KD SH-SY5Y cells. While in the former over 40% of the cells displayed a clear nuclear stain, in the latter only isolated nuclear stain was detectable.

### Whole Genome Expression Profiling

The microarray data are MIAME compliant and have been deposited in the Gene Expression Omnibus (GEO) repository (Accession number GSE30677).

## Supporting Information

Figure S1 **Venn diagram of the genes differentially expressed between Mock and** ***LMNA*** **-KD cells after RA treatment.** The sets contain genes with an average relative ratio greater than 2 or less than 0.5 on a linear scale. The whole {Mock_RAvsNT_} set, obtained by comparing RA-treated samples with untreated (NT) ones in Mock cells and by calculating the Mock RA/Mock NT ratio, is composed of a total of 3061 genes, while the whole {*LMNA*-KD_RAvsNT_} set, obtained by comparing RA-treated samples with NT ones in *LMNA*-KD cells and by calculating the *LMNA*-KD RA/*LMNA*-KD NT ratio, is composed by a total of 2089 genes. The {Mock_RAvsNT_}\{*LMNA*-KD_RAvsNT_} subset (in white) is composed of 1519 up-regulated and 222 down-regulated genes. The {*LMNA*-KD_RAvsNT_}\{Mock_RAvsNT_} subset (in dark grey) is composed of 499 up-regulated and 270 down-regulated genes.
(TIF)

Figure S2 **Western blot analysis of MMP2 expression in the cell lysates.** Western blot analysis of MMP-2 proteins in total cell lysates obtained from Mock and LMNA-KD cells. HSP70/72 protein amount were used to check equal loading and transfer of proteins. Representative of three independent experiments is shown.
(TIF)

Table S1 **Functional analysis of differential gene lists performed by the DAVID web tool showing statistically over-represented functional groups in {Mock_RAvsNT_}\{** ***LMNA*** **-KD_RAvsNT_}.**
(DOC)

Table S2 **Functional analysis of differential gene lists performed by the DAVID web tool showing statistically over-represented functional groups in {** ***LMNA*** **-KD_RAvsNT_}\{Mock_RAvsNT_}.**
(DOC)

Table S3 **List of aggressiveness-related genes modulated in** ***LMNA*** **-KD cells.** This gene set was selected according to the annotations in the NCI Cancer Gene Index database.
(DOC)

Table S4 **List of genes associated to “malignant tumor” and “cell movement” function in** ***LMNA*** **-KD/Mock cells resulting by IPA analysis.**
(DOC)

Table S5 **Significant differentially regulated proteins in** ***LMNA*** **-KD and Mock cells identified by label-free LC-MS^E^.**
(DOC)

Table S6 **Peptide table of differentially expressed proteins identified by nLC-MS^E^ reported for each run.**
(DOC)

Table S7 **Protein specifications of the network shown in** **Figure 4** **.**
(DOC)

Table S8 **Primers sequences for SYBR green assays.**
(DOC)

Table S9 **TaqMan assays validated for the indicated human genes.**
(DOC)

Materials and Methods S1 **Supporting materials and methods and supporting references.**
(DOC)
