# Supplementary figures and images for: LMNA Knock-Down Affects Differentiation and Progression of Human Neuroblastoma Cells
Source: PLoS One. 2012 Sep 26;7(9):e45513. doi: 10.1371/journal.pone.0045513 (PMC3458895; doi:10.1371/journal.pone.0045513)

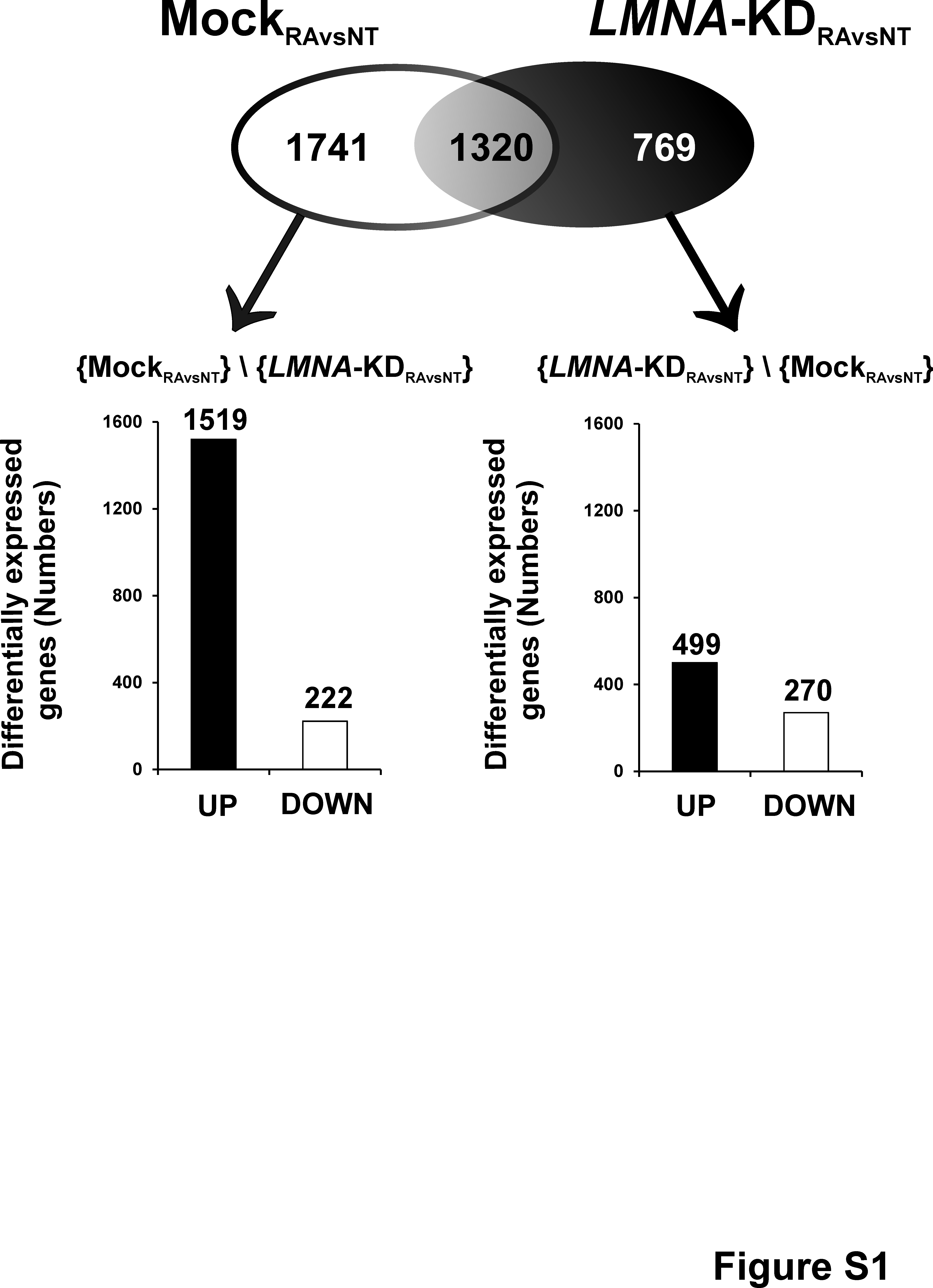

Supplement: Figure S1 — Venn diagram of the genes differentially expressed between Mock and LMNA -KD cells after RA treatment. The sets contain genes with an average relative ratio greater than 2 or less than 0.5 on a linear scale. The whole {MockRAvsNT} set, obtained by comparing RA-treated samples with untreated (NT) ones in Mock cells and by calculating the Mock RA/Mock NT ratio, is composed of a total of 3061 genes, while the whole {LMNA-KDRAvsNT} set, obtained by comparing RA-treated samples with NT ones in LMNA-KD cells and by calculating the LMNA-KD RA/LMNA-KD NT ratio, is composed by a total of 2089 genes. The {MockRAvsNT}\{LMNA-KDRAvsNT} subset (in white) is composed of 1519 up-regulated and 222 down-regulated genes. The {LMNA-KDRAvsNT}\{MockRAvsNT} subset (in dark grey) is composed of 499 up-regulated and 270 down-regulated genes. (TIF) [file pone.0045513.s001.tif]

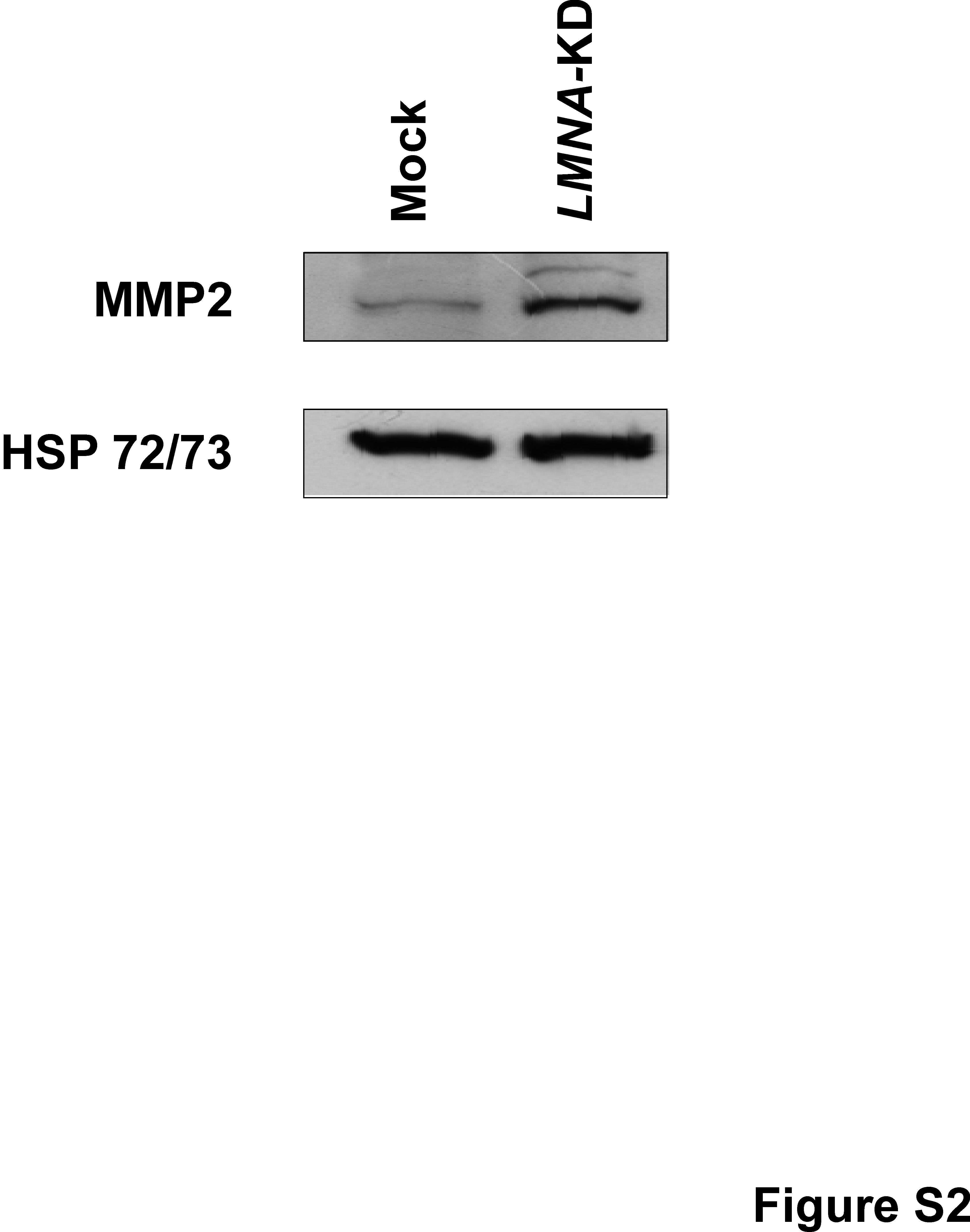

Supplement: Figure S2 — Western blot analysis of MMP2 expression in the cell lysates. Western blot analysis of MMP-2 proteins in total cell lysates obtained from Mock and LMNA-KD cells. HSP70/72 protein amount were used to check equal loading and transfer of proteins. Representative of three independent experiments is shown. (TIF) [file pone.0045513.s002.tif]
