# Supplementary material for: LMNA Knock-Down Affects Differentiation and Progression of Human Neuroblastoma Cells
Source: PLoS One. 2012 Sep 26;7(9):e45513. doi: 10.1371/journal.pone.0045513 (PMC3458895; doi:10.1371/journal.pone.0045513)
Supplement: Table S2 — Functional analysis of differential gene lists performed by the DAVID web tool showing statistically over-represented functional groups in { LMNA -KDRAvsNT}\{MockRAvsNT}. (DOC) [file pone.0045513.s004.doc]

**Table S2**

Functional analysis of differential gene lists performed by the DAVID web tool showing statistically over-represented functional groups in

{*LMNA*-KD_RAvsNT_} \ {Mock_RAvsNT_}
