# Supplementary material for: LMNA Knock-Down Affects Differentiation and Progression of Human Neuroblastoma Cells
Source: PLoS One. 2012 Sep 26;7(9):e45513. doi: 10.1371/journal.pone.0045513 (PMC3458895; doi:10.1371/journal.pone.0045513)
Supplement: Table S3 — List of aggressiveness-related genes modulated in LMNA -KD cells. This gene set was selected according to the annotations in the NCI Cancer Gene Index database. (DOC) [file pone.0045513.s005.doc]

**Table S3**

List of aggressiveness-related genes modulated in *LMNA*-KD cells. This gene set was selected according to the annotations in the NCI Cancer Gene Index database.

| **Gene** | **Genbank** | **Gene** | **Reference** |
| --- | --- | --- | --- |
| **Symbol** | **Accession** | **Name** |  |
| EDN1 | NM_001955 | endothelin 1 | [10] |
| CD44 | NM_000610 | CD44 molecule | [11] |
|  |  | (Indian blood group) |  |
| DCN | NM_001920 | decorin | [12] |
| H19 | NR_002196 | H19, imprinted maternally expressed | [13] |
|  |  | transcript (non-protein coding) |  |
| ACTN4 | NM_004924 | actinin, alpha 4 | [14] |
| INHBA | NM_002192 | inhibin, beta A |  |
| ALK | NM_004304 | anaplastic lymphoma receptor tyrosine kinase | [15] |
| TPM3 | NM_001043352 | tropomyosin 3 | [16] |
| CNTN2 | NM_005076 | contactin 2 (axonal) | [17] |
| LRP6 | NM_002336 | low density lipoprotein receptor-related | [18] |
|  |  | protein 6 |  |
| ASCL1 | NM_004316 | achaete-scute complex homolog 1 | [19] |
|  |  | (Drosophila) |  |
| GAL | NM_015973 | galanin prepropeptide | [20] |
| GRIA3 | NM_000828 | glutamate receptor, ionotrophic, AMPA 3 | [21] |
| ENPP2 | NM_006209 | ectonucleotide pyrophosphatase/ | [22] |
|  |  | phosphodiesterase 2 |  |
