# Supplementary material for: LMNA Knock-Down Affects Differentiation and Progression of Human Neuroblastoma Cells
Source: PLoS One. 2012 Sep 26;7(9):e45513. doi: 10.1371/journal.pone.0045513 (PMC3458895; doi:10.1371/journal.pone.0045513)
Supplement: Table S4 — List of genes associated to “malignant tumor” and “cell movement” function in LMNA -KD/Mock cells resulting by IPA analysis. (DOC) [file pone.0045513.s006.doc]

**Table S4**

List of genes associated to “malignant tumor” and “cell movement” function in *LMNA*-KD/Mock cells resulting by IPA analysis.
