# Supplementary material for: LMNA Knock-Down Affects Differentiation and Progression of Human Neuroblastoma Cells
Source: PLoS One. 2012 Sep 26;7(9):e45513. doi: 10.1371/journal.pone.0045513 (PMC3458895; doi:10.1371/journal.pone.0045513)
Supplement: Table S5 — Significant differentially regulated proteins in LMNA -KD and Mock cells identified by label-free LC-MSE. (DOC) [file pone.0045513.s007.doc]

**Table S5**

Significant differentially regulated proteins in *LMNA*-KD and Mock cells identified by label-free LC-MSE

| **Accession^a^** | **Description^b^** | **Score^c^** | ***LMNA*-KD/Mock ratio^d^** | **Log(e)Ratio^e^** | **Log(e)StdDev^f^** |
| --- | --- | --- | --- | --- | --- |
| Q6NXT2 | Histone H3.3C OS=Homo sapiens GN=H3F3C | 145.7 | 0.68 | -0.38 | 0.19 |
| P62805 | Histone H4 OS=Homo sapiens GN=HIST1H4A | 297.46 | 0.68 | -0.38 | 0.15 |
| A5A3E0 | POTE ankyrin domain family member F OS=Homo sapiens GN=POTEF | 608.91 | 0.71 | -0.34 | 0.12 |
| Q6S8J3 | POTE ankyrin domain family member E OS=Homo sapiens GN=POTEE | 723.89 | 0.74 | -0.3 | 0.1 |
| P23528 | Cofilin-1 OS=Homo sapiens GN=CFL1 | 708.22 | 1.35 | 0.3 | 0.1 |
| P27348 | 14-3-3 protein theta OS=Homo sapiens GN=YWHAQ | 327.34 | 1.36 | 0.31 | 0.16 |
| P67936 | Tropomyosin alpha-4 chain OS=Homo sapiens GN=TPM4 | 319.39 | 1.36 | 0.31 | 0.16 |
| P63104 | 14-3-3 protein zeta/delta OS=Homo sapiens GN=YWHAZ | 360.59 | 1.38 | 0.32 | 0.11 |
| Q01105 | Protein SET OS=Homo sapiens GN=SET | 144.04 | 1.38 | 0.32 | 0.21 |
| P61981 | 14-3-3 protein gamma OS=Homo sapiens GN=YWHAG | 336.92 | 1.39 | 0.33 | 0.15 |
| P62937 | Peptidyl-prolyl cis-trans isomerase A OS=Homo sapiens GN=PPIA | 630.73 | 1.39 | 0.33 | 0.07 |
| P11021 | 78 kDa glucose-regulated protein OS=Homo sapiens GN=HSPA5 | 829.65 | 1.4 | 0.34 | 0.08 |
| P07737 | Profilin-1 OS=Homo sapiens GN=PFN1 PE=1 SV=2 | 607.73 | 1.4 | 0.34 | 0.08 |
| P49458 | Signal recognition particle 9 kDa protein OS=Homo sapiens GN=SRP9 | 81.93 | 1.4 | 0.34 | 0.4 |
| P62263 | 40S ribosomal protein S14 OS=Homo sapiens GN=RPS14 | 126.42 | 1.42 | 0.35 | 0.27 |
| P30101 | Protein disulfide-isomerase A3 OS=Homo sapiens GN=PDIA3 | 445.94 | 1.42 | 0.35 | 0.11 |
| P38159 | Heterogeneous nuclear ribonucleoprotein G OS=Homo sapiens GN=RBMX | 264 | 1.43 | 0.36 | 0.18 |
| P0C7M2 | Putative heterogeneous nuclear ribonucleoprotein A1-like 3 OS=Homo sapiens GN=HNRPA1L3 | 450.65 | 1.45 | 0.37 | 0.13 |
| P16949 | Stathmin OS=Homo sapiens GN=STMN1 | 386.93 | 1.45 | 0.37 | 0.14 |
| P43243 | Matrin-3 OS=Homo sapiens GN=MATR3 | 220.99 | 1.46 | 0.38 | 0.33 |
| P25398 | 40S ribosomal protein S12 OS=Homo sapiens GN=RPS12 | 123.51 | 1.48 | 0.39 | 0.22 |
| P19338 | Nucleolin OS=Homo sapiens GN=NCL | 313.43 | 1.48 | 0.39 | 0.18 |
| P22626 | Heterogeneous nuclear ribonucleoproteins A2/B1 OS=Homo sapiens GN=HNRNPA2B1 | 626.23 | 1.49 | 0.4 | 0.09 |
| P06748 | Nucleophosmin OS=Homo sapiens GN=NPM1 | 283.17 | 1.51 | 0.41 | 0.11 |
| P84103 | Serine/arginine-rich splicing factor 3 OS=Homo sapiens GN=SRSF3 | 108.38 | 1.52 | 0.42 | 0.22 |
| Q9BRA2 | Thioredoxin domain-containing protein 17 OS=Homo sapiens GN=TXNDC17 | 114.47 | 1.52 | 0.42 | 0.37 |
| P60174 | Triosephosphate isomerase OS=Homo sapiens GN=TPI1 | 1179.31 | 1.52 | 0.42 | 0.09 |
| P35268 | 60S ribosomal protein L22 OS=Homo sapiens GN=RPL22 | 103.26 | 1.54 | 0.43 | 0.26 |
| P27797 | Calreticulin OS=Homo sapiens GN=CALR | 366.42 | 1.54 | 0.43 | 0.14 |
| P52272 | Heterogeneous nuclear ribonucleoprotein M OS=Homo sapiens GN=HNRNPM | 342.46 | 1.54 | 0.43 | 0.19 |
| P10809 | 60 kDa heat shock protein, mitochondrial OS=Homo sapiens GN=HSPD1 | 771.85 | 1.55 | 0.44 | 0.1 |
| Q07021 | Complement component 1 Q subcomponent-binding protein, mitochondrial OS=Homo sapiens GN=C1QBP | 101.04 | 1.55 | 0.44 | 0.33 |
| P62318 | Small nuclear ribonucleoprotein Sm D3 OS=Homo sapiens GN=SNRPD3 | 85.15 | 1.55 | 0.44 | 0.31 |
| Q8NBS9 | Thioredoxin domain-containing protein 5 OS=Homo sapiens GN=TXNDC5 | 216.8 | 1.55 | 0.44 | 0.28 |
| P63241 | Eukaryotic translation initiation factor 5A-1 OS=Homo sapiens GN=EIF5A | 140.64 | 1.6 | 0.47 | 0.31 |
| P30086 | Phosphatidylethanolamine-binding protein 1 OS=Homo sapiens GN=PEBP1 | 175.33 | 1.6 | 0.47 | 0.23 |
| Q99497 | Protein DJ-1 OS=Homo sapiens GN=PARK7 | 251.04 | 1.63 | 0.49 | 0.21 |
| P09936 | Ubiquitin carboxyl-terminal hydrolase isozyme L1 OS=Homo sapiens GN=UCHL1 | 345.72 | 1.63 | 0.49 | 0.14 |
| P62258 | 14-3-3 protein epsilon OS=Homo sapiens GN=YWHAE | 420.13 | 1.65 | 0.5 | 0.13 |
| P31946 | 14-3-3 protein beta/alpha OS=Homo sapiens GN=YWHAB | 240.91 | 1.7 | 0.53 | 0.28 |
| P60660 | Myosin light polypeptide 6 OS=Homo sapiens GN=MYL6 | 164.63 | 1.86 | 0.62 | 0.2 |
| Q16629 | Serine/arginine-rich splicing factor 7 OS=Homo sapiens GN=SRSF7 | 172.11 | 2.1 | 0.74 | 0.25 |
| P05387 | 60S acidic ribosomal protein P2 OS=Homo sapiens GN=RPLP2 | 135.33 | 2.89 | 1.06 | 0.29 |
| P62269 | 40S ribosomal protein S18 OS=Homo sapiens GN=RPS18 | 104.92 | Mock | Mock | - |
| P62701 | 40S ribosomal protein S4, X isoform OS=Homo sapiens GN=RPS4X | 155.62 | Mock | Mock | - |
| P46781 | 40S ribosomal protein S9 OS=Homo sapiens GN=RPS9 | 115.73 | Mock | Mock | - |
| P05388 | 60S acidic ribosomal protein P0 OS=Homo sapiens GN=RPLP0 | 143.04 | Mock | Mock | - |
| P61353 | 60S ribosomal protein L27 OS=Homo sapiens GN=RPL27 | 160.02 | Mock | Mock | - |
| P62917 | 60S ribosomal protein L8 OS=Homo sapiens GN=RPL8 | 128.35 | Mock | Mock | - |
| O43707 | Alpha-actinin-4 OS=Homo sapiens GN=ACTN4 | 502.52 | Mock | Mock | - |
| P08758 | Annexin A5 OS=Homo sapiens GN=ANXA5 | 150.04 | Mock | Mock | - |
| Q14195 | Dihydropyrimidinase-related protein 3 OS=Homo sapiens GN=DPYSL3 | 250.81 | Mock | Mock | - |
| P63244 | Guanine nucleotide-binding protein subunit beta-2-like 1 OS=Homo sapiens GN=GNB2L1 | 265.7 | Mock | Mock | - |
| P48643 | T-complex protein 1 subunit epsilon OS=Homo sapiens GN=CCT5 | 187.39 | Mock | Mock | - |
| P62851 | 40S ribosomal protein S25 OS=Homo sapiens GN=RPS25 | 121.23 | *LMNA*-KD | *LMNA*-KD | - |
| P15121 | Aldose reductase OS=Homo sapiens GN=AKR1B1 | 167.23 | *LMNA*-KD | *LMNA*-KD | - |
| Q15417 | Calponin-3 OS=Homo sapiens GN=CNN3 | 213.86 | *LMNA*-KD | *LMNA*-KD | - |
| O43852 | Calumenin OS=Homo sapiens GN=CALU | 252.67 | *LMNA*-KD | *LMNA*-KD | - |
| P24534 | Elongation factor 1-beta OS=Homo sapiens GN=EEF1B2 | 136.08 | *LMNA*-KD | *LMNA*-KD | - |
| Q15056 | Eukaryotic translation initiation factor 4H OS=Homo sapiens GN=EIF4H | 97.28 | *LMNA*-KD | *LMNA*-KD | - |
| P14314 | Glucosidase 2 subunit beta OS=Homo sapiens GN=PRKCSH | 196.22 | *LMNA*-KD | *LMNA*-KD | - |
| Q13151 | Heterogeneous nuclear ribonucleoprotein A0 OS=Homo sapiens GN=HNRNPA0 | 113.35 | *LMNA*-KD | *LMNA*-KD | - |
| O14979 | Heterogeneous nuclear ribonucleoprotein D-like OS=Homo sapiens GN=HNRPDL | 146.21 | *LMNA*-KD | *LMNA*-KD | - |
| P14866 | Heterogeneous nuclear ribonucleoprotein L OS=Homo sapiens GN=HNRNPL | 242.51 | *LMNA*-KD | *LMNA*-KD | - |
| P07910 | Heterogeneous nuclear ribonucleoproteins C1/C2 OS=Homo sapiens GN=HNRNPC | 330.53 | *LMNA*-KD | *LMNA*-KD | - |
| P67809 | Nuclease-sensitive element-binding protein 1 OS=Homo sapiens GN=YBX1 | 160.51 | *LMNA*-KD | *LMNA*-KD | - |
| P07602 | Proactivator polypeptide OS=Homo sapiens GN=PSAP | 155.99 | *LMNA*-KD | *LMNA*-KD | - |
| P26368 | Splicing factor U2AF 65 kDa subunit OS=Homo sapiens GN=U2AF2 | 168.53 | *LMNA*-KD | *LMNA*-KD | - |

a) Accession no. according to SwissProt database. b) Protein description. c) Score of protein identification given by PLGS software. d) Ratio between *LMNA*-KD and Mock cells expressed in decimal scale. e) Ratio between *LMNA*-KD and Mock cells expressed in logarithm scale. f) Standard deviation expressed on logarithm scale. *LMNA*-KD and Mock in ratio columns represent “unique” proteins, which are protein identified in one of the two conditions.
