# Supplementary material for: LMNA Knock-Down Affects Differentiation and Progression of Human Neuroblastoma Cells
Source: PLoS One. 2012 Sep 26;7(9):e45513. doi: 10.1371/journal.pone.0045513 (PMC3458895; doi:10.1371/journal.pone.0045513)
Supplement: Table S6 — Peptide table of differentially expressed proteins identified by nLC-MSE reported for each run. (DOC) [file pone.0045513.s008.doc]

**Table S6.**

Peptide table of differentially expressed proteins identified by nLC-MS^E^ reported for each run.

**Mock_1**

**Protein score^b^ avg Matched Matched seq peptide seq^h^ seq seq score^m^ z^n^ mz^o^**

**Name^a^ Mass^c^ Products^d^ Peptides^e^ Cover(%)^f^  modification^g^ Start^i^ Length^l^**

sp|P07737|PROF1_HUMAN 607.73 15054 75 9 69.29 TFVNITPAEVGVLVGK 38 16 205.48 2 822.4682

Profilin-1 607.73 15054 75 9 69.29 STGGAPTFNVTVTK 91 14 153.73 2 690.3574

607.73 15054 75 9 69.29 TLVLLMGK 108 8 56.65 2 437.7727

607.73 15054 75 9 69.29 DSPSVWAAVPGK 26 12 101.89 2 607.3157

607.73 15054 75 9 69.29 SSFYVNGLTLGGQK 56 14 91.13 2 735.8835

607.73 15054 75 9 69.29 DSLLQDGEFSMDLR 75 14 32.96 2 813.3799

607.73 15054 75 9 69.29 EGVHGGLINK 116 10 28.67 2 512.2822

607.73 15054 75 9 69.29 Carbamidomethyl+C(1) CYEMASHLR 127 9 47.97 3 389.5027

607.73 15054 75 9 69.29 PSVWAAVPGK 28 10 22.45 2 506.2839

sp|P60174|TPIS_HUMAN 1179.31 26669 166 24 72.69 VVLAYEPVWAIGTGK 160 15 215.92 2 801.9466

Triosephosphate isomerase 1179.31 26669 166 24 72.69 Carbamidomethyl+C(2) DCGATWVVLGHSER 85 14 133.12 3 529.5805

1179.31 26669 166 24 72.69 Carbamidomethyl+C(12) IIYGGSVTGATCK 206 13 101.96 2 663.8391

1179.31 26669 166 24 72.69 HVFGESDELIGQK 100 13 137.14 2 729.8661

1179.31 26669 166 24 72.69 Carbamidomethyl+C(9) VPADTEVVCAPPTAYIDFAR 33 20 184.4 3 731.3603

1179.31 26669 166 24 72.69 VVFEQTK 142 7 42.4 2 425.734

1179.31 26669 166 24 72.69 QSLGELIGTLNAAK 19 14 40.95 2 707.8995

1179.31 26669 166 24 72.69 Carbamidomethyl+C(14) VAHALAEGLGVIACIGEK 113 18 168.15 3 603.3264

1179.31 26669 166 24 72.69 ELASQPDVDGFLVGGASLKPEF 219 29 150.31 3 1010.5318 VDIINAK

1179.31 26669 166 24 72.69 VTNGAFTGEISPGMIK 69 16 72.46 2 811.4208

1179.31 26669 166 24 72.69 FFVGGNWK 6 8 34.97 2 477.7382

1179.31 26669 166 24 72.69 KQSLGELIGTLNAAK 18 15 64.12 3 514.9645

1179.31 26669 166 24 72.69 RHVFGESDELIGQK 99 14 62.74 3 538.9415

1179.31 26669 166 24 72.69 KFFVGGNWK 5 9 32.31 2 541.7897

1179.31 26669 166 24 72.69 LDEREAGITEK 131 11 26.01 2 630.8199

1179.31 26669 166 24 72.69 Carbamidomethyl+C(18) VTNGAFTGEISPGMIKDCGATW 69 30 11.99 5 641.9103

Oxidation+M(14) VVLGHSER

1179.31 26669 166 24 72.69 GATWVVLGHSER 87 12 7.48 2 656.3471

1179.31 26669 166 24 72.69 PPTAYIDFAR 43 10 21.54 2 575.798

1179.31 26669 166 24 72.69 Carbamidomethyl+C(1) CGATWVVLGHSER 86 13 16.45 3 491.2447

1179.31 26669 166 24 72.69 TWVVLGHSER 89 10 16.45 2 592.3165

1179.31 26669 166 24 72.69 LAYEPVWAIGTGK 162 13 21.73 2 702.88

1179.31 26669 166 24 72.69 Carbamidomethyl+C(5) GVIACIGEK 122 9 21.52 2 473.7548

1179.31 26669 166 24 72.69 Carbamidomethyl+C(9) VPADTEVVCAPPTAYIDFAR 33 20 5.2 3 725.3567

sp|P23528|COF1_HUMAN 708.22 18502 85 10 51.2 YALYDATYETK 81 11 105.53 2 669.3153

Cofilin-1 708.22 18502 85 10 51.2 Carbamidomethyl+C(7) HELQANCYEEVKDR 132 14 62.74 3 597.6058

708.22 18502 85 10 51.2 LGGSAVISLEGKPL 152 14 72.96 2 670.895

708.22 18502 85 10 51.2 NIILEEGKEILVGDVGQTVDDPY ATFVK 45 28 245.08 3 1021.5357

708.22 18502 85 10 51.2 Carbamidomethyl+C(7) MLPDKDCR 73 8 70.56 2 517.7417

708.22 18502 85 10 51.2 Carbamidomethyl+C(5) AVLFCLSEDK 34 10 64.26 2 591.2997

708.22 18502 85 10 51.2 EILVGDVGQTVDDPYATFVK 53 20 95.12 2 1083.5582

708.22 18502 85 10 51.2 GDVGQTVDDPYATFVK 57 16 42.85 2 856.4193

708.22 18502 85 10 51.2 PYATFVK 66 7 22.35 2 413.231

708.22 18502 85 10 51.2 NIILEEGKEILVGDVGQTVDDPY 45 28 7.97 3 1015.5289

ATFVK

sp|P16949|STMN1_HUMAN 386.93 17302 40 8 30.87 ASGQAFELILSPR 14 13 172.73 2 694.8795

Stathmin 386.93 17302 40 8 30.87 AIEENNNFSK 85 10 54.34 2 583.2767

386.93 17302 40 8 30.87 DLSLEEIQK 43 9 95.7 2 537.7881

386.93 17302 40 8 30.87 ESVPEFPLSPPK 29 12 31.13 2 663.8542

386.93 17302 40 8 30.87 SKESVPEFPLSPPK 27 14 54.29 3 514.6097

386.93 17302 40 8 30.87 PEFPLSPPK 32 9 7.88 2 506.2768

386.93 17302 40 8 30.87 SLEEIQK 45 7 9.27 1 846.4572

386.93 17302 40 8 30.87 EENNNFSK 87 8 1.73 1 981.424

sp|P62937|PPIA_HUMAN 630.73 18012 99 15 69.09 FEDENFILK 82 9 64.88 2 577.7871

Peptidyl-prolyl cis-trans isomerase A 630.73 18012 99 15 69.09 VSFELFADK 19 9 84.81 2 528.2697

630.73 18012 99 15 69.09 TEWLDGK 118 7 42.4 2 424.7065

630.73 18012 99 15 69.09 Carbamidomethyl+C(7) IIPGFMCQGGDFTR 55 14 75.75 2 799.8755

630.73 18012 99 15 69.09 EGMNIVEAMER 133 11 85.5 2 639.7973

630.73 18012 99 15 69.09 VNPTVFFDIAVDGEPLGR 1 18 116.24 2 973.5114

630.73 18012 99 15 69.09 Carbamidomethyl+C(24) HTGPGILSMANAGPNTNGSQFFICTAK 91 27 105.98 3 931.1146

630.73 18012 99 15 69.09 VKEGMNIVEAMER 131 13 97.77 2 753.3808

630.73 18012 99 15 69.09 SIYGEKFEDENFILK 76 15 59.11 3 611.306

630.73 18012 99 15 69.09 Carbamidomethyl+C(7) KITIADCGQLE 154 11 39.12 2 624.3181

630.73 18012 99 15 69.09 FELFADK 21 7 24.42 1 869.4325

630.73 18012 99 15 69.09 Carbamidomethyl+C(5) PGFMCQGGDFTR 57 12 7.48 2 686.7977

630.73 18012 99 15 69.09 DENFILK 84 7 24.42 1 878.4577

630.73 18012 99 15 69.09 SFELFADK 20 8 24.42 1 956.4692

630.73 18012 99 15 69.09 VDGEPLGR 11 8 21.52 1 842.4272

sp|P0C7M2|RA1L3_HUMAN 450.65 34223 75 10 33.75 IEVIEIMTDR 130 10 132.99 2 609.8213

Putative heterogeneous nuclear 450.65 34223 75 10 33.75 EDSQRPGAHLTVK 92 13 44.3 3 479.9146

ribonucleoprotein A1-like 3 450.65 34223 75 10 33.75 DYFEQYGK 122 8 37.98 2 525.2334

450.65 34223 75 10 33.75 GFAFVTFDDHDSVDK 146 15 137.83 3 567.2537

450.65 34223 75 10 33.75 LFIGGLSFETTDESLR 15 16 92.03 2 892.9604

450.65 34223 75 10 33.75 IFVGGIK 106 7 56.31 2 367.2299

450.65 34223 75 10 33.75 GFGFVTYATVEEVDAAMNARPHK 55 23 65.8 4 628.3013

450.65 34223 75 10 33.75 KLFIGGLSFETTDESLR 14 17 50.23 3 638.3333

450.65 34223 75 10 33.75 ALSKQEMASASSSQR 179 15 27.69 3 527.5982

450.65 34223 75 10 33.75 AFVTFDDHDSVDK 148 13 7.37 2 748.3412

sp|P62258|1433E_HUMAN 420.13 29173 62 9 23.14 NLLSVAYK 42 8 56.65 2 454.2613

14-3-3 protein epsilon 420.13 29173 62 9 23.14 Carbamidomethyl+C(3) LICCDILDVLDK 94 12 129.7 2 738.8761

Carbamidomethyl+C(4)

420.13 29173 62 9 23.14 HLIPAANTGESK 106 12 52.7 2 619.329

420.13 29173 62 9 23.14 DSTLIMQLLR 215 10 105.18 2 595.3351

420.13 29173 62 9 23.14 YLAEFATGNDRK 130 12 82.84 3 462.2296

420.13 29173 62 9 23.14 YLAEFATGNDR 130 11 68.59 2 628.8086

420.13 29173 62 9 23.14 VFYYK 118 5 13.9 2 360.1897

420.13 29173 62 9 23.14 Carbamidomethyl+C(3) LICCDILDVLDKHLIPAANTGESK 94 24 38.73 4 674.5939

Carbamidomethyl+C(4)

420.13 29173 62 9 23.14 YLAEFATGNDRK 130 12 39.59 3 462.232

sp|P11021|GRP78_HUMAN 829.65 72333 154 32 41.28 VEIIANDQGNR 49 11 117.21 2 614.8157

78 kDa glucose-regulated protein 829.65 72333 154 32 41.28 IINEPTAAAIAYGLDKR 197 17 80.69 3 605.9978

829.65 72333 154 32 41.28 IINEPTAAAIAYGLDK 197 16 101.16 2 830.4535

829.65 72333 154 32 41.28 IEWLESHQDADIEDFK 601 16 18.58 3 658.9741

829.65 72333 154 32 41.28 VTHAVVTVPAYFNDAQR 164 17 96.25 3 629.9924

829.65 72333 154 32 41.28 NELESYAYSLK 562 11 89.4 2 658.8275

829.65 72333 154 32 41.28 ELEEIVQPIISK 621 12 47.17 2 699.4003

829.65 72333 154 32 41.28 TFAPEEISAMVLTK 138 14 71.2 2 768.9028

829.65 72333 154 32 41.28 TWNDPSVQQDIK 101 12 37.15 2 715.8512

829.65 72333 154 32 41.28 NQLTSNPENTVFDAK 81 15 73.01 2 839.4124

829.65 72333 154 32 41.28 ITPSYVAFTPEGER 60 14 33.75 2 783.8982

829.65 72333 154 32 41.28 SQIFSTASDNQPTVTIK 447 17 48.5 2 918.9753

829.65 72333 154 32 41.28 TKPYIQVDIGGGQTK 123 15 25.63 3 535.6235

829.65 72333 154 32 41.28 FEELNMDLFR 326 10 43.44 2 657.3152

829.65 72333 154 32 41.28 VYEGERPLTK 464 10 30.43 3 397.8783

829.65 72333 154 32 41.28 LSSEDK 585 6 14.25 1 678.3372

829.65 72333 154 32 41.28 VVEK 118 4 13.56 1 474.2909

829.65 72333 154 32 41.28 DAGTIAGLNVMR 185 12 67.27 2 609.3196

829.65 72333 154 32 41.28 GTGNK 516 5 13.9 1 476.2463

829.65 72333 154 32 41.28 FLPFK 113 5 13.9 2 326.1973

829.65 72333 154 32 41.28 ETAEAYLGK 154 9 32.31 2 491.2532

829.65 72333 154 32 41.28 AKFEELNMDLFR 324 12 95.08 3 504.9186

829.65 72333 154 32 41.28 KSDIDEIVLVGGSTR 352 15 25.63 3 530.2837

829.65 72333 154 32 41.28 EFFNGKEPSR 376 10 28.67 3 404.2031

829.65 72333 154 32 41.28 ITPSYVAFTPEGERLIGDAAK 60 21 23.45 4 559.5432

829.65 72333 154 32 41.28 LGGKLSSEDK 581 10 28.67 2 517.2823

829.65 72333 154 32 41.28 NQLTSNPENTVFDAK 81 15 25.63 3 586.5971

829.65 72333 154 32 41.28 TFAPEEISAMVLTK 138 14 26.26 3 539.5905

829.65 72333 154 32 41.28 NEPTAAAIAYGLDKR 199 15 4.16 2 795.417

829.65 72333 154 32 41.28 PAYFNDAQR 172 9 4.16 2 541.2603

829.65 72333 154 32 41.28 YFNDAQR 174 7 4.16 2 457.2159

829.65 72333 154 32 41.28 WLESHQDADIEDFK 603 14 3.81 2 866.9038

sp|P09936|UCHL1_HUMAN 345.72 24824 49 10 27.35 Carbamidomethyl+C(17) NEAIQAAHDAVAQEGQCR 135 18 129.55 3 656.6353

Ubiquitin carboxyl-terminal hydrolase 345.72 24824 49 10 27.35 LGFEDGSVLK 105 10 61.25 2 532.7847

isozyme L1 345.72 24824 49 10 27.35 LGVAGQWR 19 8 65.79 2 443.7458

345.72 24824 49 10 27.35 Carbamidomethyl+C(7) FSAVALCK 213 8 70.56 2 448.2366

345.72 24824 49 10 27.35 EFTEREQGEVR 202 11 26.01 2 690.3351

345.72 24824 49 10 27.35 QIEELK 65 6 14.25 1 759.4249

345.72 24824 49 10 27.35 Oxidation+M(1) MQLKPMEINPEMLNK 0 15 19.61 3 611.3047

345.72 24824 49 10 27.35 NEAIQAAHDAV 135 11 4.51 2 569.7779

345.72 24824 49 10 27.35 Carbamidomethyl+C(15) AIQAAHDAVAQEGQCR 137 16 7.27 2 862.9062

345.72 24824 49 10 27.35 Carbamidomethyl+C(7) FSAVALCK 213 8 10.17 2 439.2358

sp|P22626|ROA2_HUMAN 626.23 37429 82 16 33.14 IDTIEIITDR 137 10 136 2 594.8266

Heterogeneous nuclear 626.23 37429 82 16 33.14 GGGGNFGPGPGSNFR 213 15 73.88 2 689.3168

ribonucleoproteins A2/B1 626.23 37429 82 16 33.14 GFGFVTFDDHDPVDK 153 15 122.17 3 565.9219

626.23 37429 82 16 33.14 GGNFGFGDSR 203 10 78.17 2 507.2257

626.23 37429 82 16 33.14 YHTINGHNAEVR 173 12 47.25 3 470.9

626.23 37429 82 16 33.14 LFIGGLSFETTEESLR 22 16 105.93 2 899.9674

626.23 37429 82 16 33.14 QEMQEVQSSR 190 10 15.56 2 611.2794

626.23 37429 82 16 33.14 TLETVPLER 3 9 47.97 2 529.2968

626.23 37429 82 16 33.14 LFVGGIK 113 7 42.4 2 367.2298

626.23 37429 82 16 33.14 Carbamidomethyl+C(4) LTDCVVMRDPASK 46 13 22.23 3 497.9115

626.23 37429 82 16 33.14 GFVTFDDHDPVDK 155 13 3.47 2 746.3425

626.23 37429 82 16 33.14 VTFDDHDPVDK 157 11 3.47 2 644.297

626.23 37429 82 16 33.14 FVTFDDHDPVDK 156 12 3.47 2 717.8379

626.23 37429 82 16 33.14 TIEIITDR 139 8 23.52 2 480.7701

626.23 37429 82 16 33.14 FGFVTFDDHDPVDK 154 14 3.47 3 546.9205

626.23 37429 82 16 33.14 GFGFVTFDDHDPVDK 153 15 3.47 3 559.9201

sp|Q6NXT2|H3C_HUMAN 145.7 15213 19 3 20 YRPGTVALR 40 9 93.94 2 516.7997

Histone H3.3C 145.7 15213 19 3 20 STELLIR 56 7 53.3 2 416.2465

145.7 15213 19 3 20 EIAQDFNTDLR 72 11 37.69 3 441.2212

sp|P62805|H4_HUMAN 297.46 11367 47 11 51.46 VFLENVIR 60 8 79.69 2 495.2894

Histone H4 297.46 11367 47 11 51.46 DAVTYTEHAK 68 10 35.66 2 567.7738

297.46 11367 47 11 51.46 ISGLIYEETR 46 10 93.04 2 590.8125

297.46 11367 47 11 51.46 TVTAMDVVYALKR 80 13 86.19 3 489.6007

297.46 11367 47 11 51.46 DNIQGITKPAIR 24 12 69.62 3 442.5835

297.46 11367 47 11 51.46 TVTAMDVVYALK 80 12 23.92 2 655.8575

297.46 11367 47 11 51.46 TVTAMDVVYALKR 80 13 22.23 2 773.8887

297.46 11367 47 11 51.46 TYTEHAK 71 7 1.73 2 425.2073

297.46 11367 47 11 51.46 TAMDVVYALKR 82 11 7.65 2 633.8481

297.46 11367 47 11 51.46 FLENVIR 61 7 10.17 2 445.7557

297.46 11367 47 11 51.46 ISGLIYEETR 46 10 1.73 2 581.8008

sp|P10809|CH60_HUMAN 771.85 61054 124 24 39.79 VGLQVVAVK 292 9 80.04 2 456.7942

60 kDa heat shock protein 771.85 61054 124 24 39.79 LSDGVAVLK 396 9 40.09 2 451.2692

771.85 61054 124 24 39.79 ISSIQSIVPALEIANAHR 250 18 43.59 3 640.3571

771.85 61054 124 24 39.79 TVIIEQSWGSPK 60 12 111.54 2 672.8616

771.85 61054 124 24 39.79 VTDALNATR 420 9 93.94 2 480.7571

771.85 61054 124 24 39.79 TLNDELEIIEGMK 205 13 36.35 2 752.8821

771.85 61054 124 24 39.79 IGIEIIK 462 7 39.39 2 393.2577

771.85 61054 124 24 39.79 NAGVEGSLIVEK 481 12 34.14 2 608.331

771.85 61054 124 24 39.79 VGGTSDVEVNEK 405 12 99.75 2 617.3024

771.85 61054 124 24 39.79 IQEIIEQLDVTTSEYEKEK 370 19 45.51 3 765.7251

771.85 61054 124 24 39.79 Carbamidomethyl+C(13) AAVEEGIVLGGGCALLR 429 17 99.08 2 842.9588

771.85 61054 124 24 39.79 GVMLAVDAVIAELKK 142 15 31.6 3 519.636

771.85 61054 124 24 39.79 LVQDVANNTNEEAGDGTTTATVLAR 96 25 13.3 3 854.0886

771.85 61054 124 24 39.79 ALMLQGVDLLADAVAVTMGPK 37 21 86.15 3 705.0486

771.85 61054 124 24 39.79 SIDLK 82 5 41.71 1 575.3367

771.85 61054 124 24 39.79 DDAMLLK 352 7 42.4 2 403.2124

771.85 61054 124 24 39.79 EIGNIISDAMK 180 11 29.02 2 595.8182

771.85 61054 124 24 39.79 FGADAR 31 6 14.25 1 636.3096

771.85 61054 124 24 39.79 KISSIQSIVPALEIANAHR 249 19 40.05 3 683.0536

771.85 61054 124 24 39.79 MLAVDAVIAELKK 144 13 15.71 2 700.9148

771.85 61054 124 24 39.79 DGVAVLK 398 7 9.27 1 701.4181

771.85 61054 124 24 39.79 AVDAVIAELKK 146 11 21.73 2 578.8558

771.85 61054 124 24 39.79 SDGVAVLK 397 8 9.27 1 788.4489

771.85 61054 124 24 39.79 LAVDAVIAELKK 145 12 7.37 2 635.3908

sp|P63104|1433Z_HUMAN 360.59 27745 47 10 34.29 NLLSVAYK 41 8 56.65 2 454.2613

14-3-3 protein zeta/delta 360.59 27745 47 10 34.29 FLIPNASQAESK 103 12 52.7 2 652.8465

360.59 27745 47 10 34.29 Carbamidomethyl+C(3) DICNDVLSLLEK 91 12 52.7 2 709.8655

360.59 27745 47 10 34.29 DSTLIMQLLR 212 10 105.18 2 595.3351

360.59 27745 47 10 34.29 GIVDQSQQAYQEAFEISK 139 18 26.81 2 1021.003

360.59 27745 47 10 34.29 YLAEVAAGDDKK 127 12 27.9 2 640.3346

360.59 27745 47 10 34.29 IETELR 85 6 14.25 2 380.7107

360.59 27745 47 10 34.29 NELVQK 3 6 14.25 1 730.4169

360.59 27745 47 10 34.29 YLAEVAAGDDK 127 11 26.01 2 576.2892

360.59 27745 47 10 34.29 PNASQAESK 106 9 7.88 2 466.229

sp|Q99497|PARK7_HUMAN 251.04 19891 39 13 57.67 Carbamidomethyl+C(14) VTVAGLAGKDPVQCSR 32 16 101.24 3 553.2909

Protein DJ-1 251.04 19891 39 13 57.67 Carbamidomethyl+C(5) DVVICPDASLEDAKK 48 15 27.69 3 553.9437

251.04 19891 39 13 57.67 APLVLKD 182 7 42.4 2 378.2341

251.04 19891 39 13 57.67 EGPYDVVVLPGGNLGAQNLSESAAVK 63 26 24.33 3 862.1141

251.04 19891 39 13 57.67 GAEEMETVIPVDVMR 12 15 45.86 2 838.4063

251.04 19891 39 13 57.67 Carbamidomethyl+C(7) GLIAAICAGPTALLAHEIGFGSK 99 23 14.06 3 756.4102

251.04 19891 39 13 57.67 ALVILAK 5 7 42.4 2 364.2561

251.04 19891 39 13 57.67 APLVLK 182 6 14.25 1 640.4393

251.04 19891 39 13 57.67 Carbamidomethyl+C(3) VICPDASLEDAKK 50 13 7.37 2 723.3718

251.04 19891 39 13 57.67 Carbamidomethyl+C(2) ICPDASLEDAKK 51 12 3.47 2 673.836

251.04 19891 39 13 57.67 Carbamidomethyl+C(10) GLAGKDPVQCSR 36 12 7.3 2 644.3327

251.04 19891 39 13 57.67 Carbamidomethyl+C(12) VAGLAGKDPVQCSR 34 14 7.3 2 729.3855

251.04 19891 39 13 57.67 Carbamidomethyl+C(7) GLIAAICAGPTALLAHEIG 99 19 6.24 2 924.5016

sp|P61981|1433G_HUMAN 336.92 28302 48 9 33.6 NLLSVAYK 42 8 56.65 2 454.2613

14-3-3 protein gamma 336.92 28302 48 9 33.6 DSTLIMQLLR 217 10 105.18 2 595.3351

336.92 28302 48 9 33.6 NVTELNEPLSNEER 28 14 40.74 2 822.3989

336.92 28302 48 9 33.6 YLAEVATGEK 132 10 35.66 2 540.7803

336.92 28302 48 9 33.6 Carbamidomethyl+C(6) ELEAVCQDVLSLLDNYLIK 91 19 85.75 3 745.7255

336.92 28302 48 9 33.6 MVDR 0 4 13.56 1 520.253

336.92 28302 48 9 33.6 AYSEAHEISK 152 10 43.44 2 567.7821

336.92 28302 48 9 33.6 IEMVR 78 5 27.81 1 647.3482

336.92 28302 48 9 33.6 IEMVRAYR 78 8 37.98 2 519.2765

sp|P05387|RLA2_HUMAN 135.33 11664 29 4 69.57 LASVPAGGAVAVSAAPGSAAPAAGSA 61 33 72.74 3 925.4839

60S acidic ribosomal protein P2 PAAAEEK

135.33 11664 29 4 69.57 YVASYLLAALGGNSSPSAK 2 19 48.94 2 934.9963

135.33 11664 29 4 69.57 NIEDVIAQGIGK 49 12 52.7 2 628.8482

135.33 11664 29 4 69.57 ILDSVGIEADDDRLNK 25 16 72.46 3 591.636

sp|P67936|TPM4_HUMAN 319.39 28521 48 8 31.05 IQLVEEELDR 55 10 106.94 2 622.328

Tropomyosin alpha-4 chain 319.39 28521 48 8 31.05 LVILEGELER 132 10 55.69 2 585.8377

319.39 28521 48 8 31.05 AEGDVAALNR 44 10 42.58 2 508.2647

319.39 28521 48 8 31.05 MEIQEMQLK 104 9 78.79 2 575.2887

319.39 28521 48 8 31.05 AQGLQRELDGER 27 12 18.47 2 686.36

319.39 28521 48 8 31.05 AGLNSLEAVK 1 10 28.67 2 501.2854

319.39 28521 48 8 31.05 KLVILEGELER 131 11 55.58 3 433.5896

319.39 28521 48 8 31.05 NVTNNLKSLEAASEK 162 15 27.69 2 809.4179

sp|P25398|RS12_HUMAN 123.51 14515 19 5 34.85 Carbamidomethyl+C(6) LVEALCAEHQINLIK 63 15 42.85 3 584.3181

40S ribosomal protein S12 123.51 14515 19 5 34.85 Carbamidomethyl+C(8) LGEWVGLCK 84 9 64.88 2 531.276

123.51 14515 19 5 34.85 AEEGIAAGGVMDVNTALQEVLK 1 22 14.51 3 739.0426

123.51 14515 19 5 34.85 Carbamidomethyl+C(4) EALCAEHQINLIK 65 13 7.37 2 769.9079

123.51 14515 19 5 34.85 Carbamidomethyl+C(5) VEALCAEHQINLIK 64 14 7.37 2 819.4313

sp|P27348|1433T_HUMAN 327.34 27764 45 7 35.1 NLLSVAYK 41 8 56.65 2 454.2613

14-3-3 protein theta 327.34 27764 45 7 35.1 Carbamidomethyl+C(3) SICTTVLELLDK 91 12 88.29 2 696.3766

327.34 27764 45 7 35.1 DSTLIMQLLR 212 10 105.18 2 595.3351

327.34 27764 45 7 35.1 YLIANATNPESK 103 12 23.92 2 660.844

327.34 27764 45 7 35.1 AVTEQGAELSNEER 27 14 20.82 3 511.5794

327.34 27764 45 7 35.1 Carbamidomethyl+C(7) YLAEVACGDDRK 127 12 27.9 3 466.2221

327.34 27764 45 7 35.1 QTIDNSQGAYQEAFDISK 139 18 25.8 3 672.3107

sp|P30101|PDIA3_HUMAN 445.94 56782 57 14 25.74 LAPEYEAAATR 62 11 71.6 2 596.3038

Protein disulfide-isomerase A3 445.94 56782 57 14 25.74 FVMQEEFSR 335 9 32.31 2 586.7795

445.94 56782 57 14 25.74 ELSDFISYLQR 471 11 59.04 2 685.8507

445.94 56782 57 14 25.74 TFSHELSDFGLESTAGEIPVVAIR 305 24 76.24 3 859.1074

445.94 56782 57 14 25.74 LNFAVASR 296 8 79.69 2 439.245

445.94 56782 57 14 25.74 FLQDYFDGNLK 351 11 37.77 2 680.3363

445.94 56782 57 14 25.74 GIVPLAK 75 7 53.3 2 349.2307

445.94 56782 57 14 25.74 DASIVGFFDDSFSEAHSEFLK 152 21 20.74 3 783.3676

445.94 56782 57 14 25.74 TADGIVSHLK 119 10 28.67 2 520.79

445.94 56782 57 14 25.74 GFPTIYFSPANK 448 12 23.92 3 447.8918

445.94 56782 57 14 25.74 AASNLR 173 6 14.25 1 631.3551

445.94 56782 57 14 25.74 PEYEAAATR 64 9 2.08 2 504.2429

445.94 56782 57 14 25.74 APEYEAAATR 63 10 2.08 2 539.7632

445.94 56782 57 14 25.74 ELSDFISYLQR 471 11 2.08 2 676.8462

sp|P31946|1433B_HUMAN 240.91 28082 32 6 21.14 NLLSVAYK 43 8 56.65 2 454.2613

14-3-3 protein beta/alpha 240.91 28082 32 6 21.14 DSTLIMQLLR 214 10 105.18 2 595.3351

240.91 28082 32 6 21.14 YLIPNATQPESK 105 12 38.5 2 680.8599

240.91 28082 32 6 21.14 YLSEVASGDNK 129 11 43.89 2 591.7859

240.91 28082 32 6 21.14 SELVQK 5 6 14.25 1 703.3933

240.91 28082 32 6 21.14 MTMDKSELVQK 0 11 26.01 3 437.2258

sp|P30086|PEBP1_HUMAN 175.33 21056 23 5 30.48 LYTLVLTDPDAPSR 62 14 77.1 2 780.914

Phosphatidylethanolamine- 175.33 21056 23 5 30.48 VLTPTQVK 39 8 42.75 2 443.2699

binding protein 1 175.33 21056 23 5 30.48 GNDISSGTVLSDYVGSGPPK 93 20 33.87 2 975.4861

175.33 21056 23 5 30.48 NRPTSISWDGLDSGK 47 15 75.53 2 816.9087

175.33 21056 23 5 30.48 TVLSDYVGSGPPK 100 13 5.2 2 660.3484

sp|P62701|RS4X_HUMAN 155.62 29597 16 4 9.51 LSNIFVIGK 221 9 47.97 2 495.802

40S ribosomal protein S4 155.62 29597 16 4 9.51 Carbamidomethyl+C(2) ECLPLIIFLR 39 10 76.12 2 637.3713

155.62 29597 16 4 9.51 HWMLDK 16 6 14.25 2 415.2012

155.62 29597 16 4 9.51 Oxidation+M(10) TDITYPAGFMDVISIDK 77 17 17.67 3 634.6468

sp|P27797|CALR_HUMAN 366.42 48141 58 12 36.21 IKDPDASKPEDWDER 207 15 38.87 3 600.9489

Calreticulin 366.42 48141 58 12 36.21 EQFLDGDGWTSR 24 12 85.85 2 705.824

366.42 48141 58 12 36.21 FYGDEEKDK 55 9 67.89 2 565.7562

366.42 48141 58 12 36.21 FVLSSGK 48 7 56.31 2 369.2112

366.42 48141 58 12 36.21 FYALSASFEPFSNK 73 14 29.79 2 804.3938

366.42 48141 58 12 36.21 Carbamidomethyl+C(7) HEQNIDCGGGYVK 98 13 88.44 3 492.8931

366.42 48141 58 12 36.21 IDNSQVESGSLEDDWDFLPPKK 185 22 14.51 3 840.4094

366.42 48141 58 12 36.21 KPEDWDEEMDGEWEPPVIQNPEYK 248 24 13.66 3 987.4424

366.42 48141 58 12 36.21 IDDPTDSKPEDWDKPEHIPDPDAK 224 24 38.73 5 552.8493

366.42 48141 58 12 36.21 GQTLVVQFTVK 87 11 26.01 3 407.2411

366.42 48141 58 12 36.21 Carbamidomethyl+C(5) QNIDCGGGYVK 100 11 2.77 2 605.7851

366.42 48141 58 12 36.21 Carbamidomethyl+C(1) CGGGYVK 104 7 7.65 1 740.3382

sp|P35268|RL22_HUMAN 103.26 14787 21 5 43.75 ITVTSEVPFSK 69 11 68.59 2 604.3321

60S ribosomal protein L22 103.26 14787 21 5 43.75 Carbamidomethyl+C(5) FTLDCTHPVEDGIMDAANFEQFLQER 20 26 23.65 3 1028.4757

103.26 14787 21 5 43.75 AGNLGGGVVTIER 52 13 22.23 2 621.8448

103.26 14787 21 5 43.75 ESYELR 107 6 14.25 1 796.3775

103.26 14787 21 5 43.75 ITVTSEVPFSKR 69 12 52.7 2 722.3684

sp|Q9BRA2|TXD17_HUMAN 114.47 13940 11 4 26.83 Carbamidomethyl+C(3) SWCPDCVQAEPVVR 40 14 28.6 2 851.8924

Thioredoxin domain-containing Carbamidomethyl+C(6)

protein 17 114.47 13940 11 4 26.83 VTAVPTLLK 89 9 57 2 471.3035

114.47 13940 11 4 26.83 TIFAYFTGSK 25 10 38.67 2 567.7956

114.47 13940 11 4 26.83 AVPTLLK 91 7 1.39 1 741.4838

sp|Q8NBS9|TXND5_HUMAN 216.8 47628 27 9 16.2 ALAPTWEQLALGLEHSETVK 221 20 59.76 3 731.7221

Thioredoxin domain-containing 216.8 47628 27 9 16.2 GYPTLLLFR 394 9 47.97 2 540.313

protein 5 216.8 47628 27 9 16.2 Carbamidomethyl+C(6) IAEVDCTAER 375 10 54.34 2 582.2738

216.8 47628 27 9 16.2 VDQYK 273 5 13.9 1 652.3295

216.8 47628 27 9 16.2 TLAPTWEELSK 354 11 33 2 637.8251

216.8 47628 27 9 16.2 EFPGLAGVK 366 9 32.31 2 459.2583

216.8 47628 27 9 16.2 DLESLR 281 6 14.25 1 732.3963

216.8 47628 27 9 16.2 PTLLLFR 396 7 1.39 2 430.2728

216.8 47628 27 9 16.2 PTWEQLALGLEHSETVK 224 17 7.34 3 646.6701

sp|P63244|GBLP_HUMAN 265.7 35076 38 9 39.43 Carbamidomethyl+C(8) LWNTLGVCK 130 9 32.31 2 545.7904

Guanine nucleotide-binding protein 265.7 35076 38 9 39.43 Carbamidomethyl+C(4) YWLCAATGPSIK 245 12 27.9 2 683.847

subunit beta-2-like 1 265.7 35076 38 9 39.43 LWDLTTGTTTR 88 11 86.39 2 632.8321

265.7 35076 38 9 39.43 Carbamidomethyl+C(15) HLYTLDGGDIINALCFSPNR 225 20 53.65 3 759.3736

265.7 35076 38 9 39.43 Carbamidomethyl+C(22) TNHIGHTGYLNTVTVSPDGSLCASGGK 185 27 12.69 4 686.5833

265.7 35076 38 9 39.43 QEVISTSSK 271 9 32.31 2 489.7529

265.7 35076 38 9 39.43 GHNGWVTQIATTPQFPDMILSASR 12 24 41.41 3 876.4354

265.7 35076 38 9 39.43 FVGHTK 100 6 14.25 2 344.6894

265.7 35076 38 9 39.43 IIVDELKQEVISTSSK 264 16 26.28 3 596.9978

sp|P38159|HNRPG_HUMAN 264 42331 42 10 23.79 VEQATKPSFESGR 80 13 43.62 3 479.2398

Heterogeneous nuclear 264 42331 42 10 23.79 LFIGGLNTETNEK 9 13 36.7 2 718.3778

ribonucleoprotein G 264 42331 42 10 23.79 GFAFVTFESPADAK 49 14 54.87 2 743.8625

264 42331 42 10 23.79 DVYLSPR 203 7 42.4 2 425.227

264 42331 42 10 23.79 VEADRPGK 1 8 37.98 2 436.2412

264 42331 42 10 23.79 ALEAVFGK 22 8 42.75 2 417.7367

264 42331 42 10 23.79 GFAFVTFESPADAKDAAR 49 18 24.28 3 633.9728

264 42331 42 10 23.79 GLPPSMERGYPPPR 347 14 62.23 2 777.3952

264 42331 42 10 23.79 GGSGGTRGPPSR 113 12 36.58 2 543.2707

264 42331 42 10 23.79 QATKPSFESGR 82 11 7.65 2 604.3063

sp|P49458|SRP09_HUMAN 81.93 10111 8 2 22.09 Carbamidomethyl+C(7) VTDDLVCLVYK 41 11 40.7 2 662.8445

Signal recognition particle 9 kDa protein 81.93 10111 8 2 22.09 LYLADPMK 16 8 42.75 2 475.7575

sp|P62318|SMD3_HUMAN 85.15 13916 9 2 15.08 VAQLEQVYIR 54 10 35.66 2 609.8457

Small nuclear ribonucleoprotein Sm D3 85.15 13916 9 2 15.08 FLILPDMLK 69 9 47.97 2 545.3232

sp|P61353|RL27_HUMAN 160.02 15797 22 4 36.03 NIDDGTSDRPYSHALVAGIDR 27 21 21.64 3 758.0335

60S ribosomal protein L27 160.02 15797 22 4 36.03 YSVDIPLDK 84 9 64.88 2 525.2803

160.02 15797 22 4 36.03 VVLVLAGR 9 8 79.69 2 413.7795

160.02 15797 22 4 36.03 VYNYNHLMPTR 73 11 26.01 3 469.8925

sp|Q16629|SRSF7_HUMAN 172.11 27366 26 9 31.93 NPPGFAFVEFEDPRDAEDAVR 44 21 24.65 3 793.371

Serine/arginine-rich splicing factor 7 172.11 27366 26 9 31.93 VELSTGMPR 78 9 47.97 2 495.2616

172.11 27366 26 9 31.93 AFSYYGPLR 29 9 57 2 537.2773

172.11 27366 26 9 31.93 VYVGNLGTGAGK 12 12 52.7 2 568.3028

172.11 27366 26 9 31.93 SPSPK 214 5 13.9 1 515.2783

172.11 27366 26 9 31.93 NPPGFAFVEFEDPR 44 14 20.82 2 811.3857

172.11 27366 26 9 31.93 TVWIAR 38 6 14.25 2 373.2248

172.11 27366 26 9 31.93 RPFDPNDR 97 8 34.97 3 339.4993

172.11 27366 26 9 31.93 SISRPR 201 6 14.25 2 358.2164

sp|P06748|NPM_HUMAN 283.17 32575 44 10 30.95 VDNDENEHQLSLR 32 13 97.09 3 523.5768

Nucleophosmin 283.17 32575 44 10 30.95 GPSSVEDIK 239 9 55.75 2 466.235

283.17 32575 44 10 30.95 MTDQEAIQDLWQWR 277 14 40.95 2 910.4283

283.17 32575 44 10 30.95 TVSLGAGAKDELHIVEAEAMNYEGSPIK 45 28 12.43 4 733.1159

283.17 32575 44 10 30.95 MSVQPTVSLGGFEITPPVVLR 80 21 80.37 3 743.0713

283.17 32575 44 10 30.95 FINYVK 267 6 42.06 2 392.2191

283.17 32575 44 10 30.95 DELHIVEAEAMNYEGSPIK 54 19 42.49 3 715.6741

283.17 32575 44 10 30.95 NDENEHQLSLR 34 11 7.65 2 677.8243

283.17 32575 44 10 30.95 DNDENEHQLSLR 33 12 2.77 2 735.336

283.17 32575 44 10 30.95 SSVEDIK 241 7 1.39 1 777.3973

sp|Q6S8J3|POTEE_HUMAN 723.89 121363 73 9 11.72 AGFAGDDAPR 718 10 120.85 2 488.7225

POTE ankyrin domain family member E 723.89 121363 73 9 11.72 SYELPDGQVITIGNER 938 16 192.16 2 895.9431

723.89 121363 73 9 11.72 Carbamidomethyl+C(2) LCYVALDFEQEMATAASSSSLEK 915 23 154.86 3 850.7308

723.89 121363 73 9 11.72 IWHHTFYNELR 784 11 43.89 3 505.9199

723.89 121363 73 9 11.72 DLIVMLRDTDVNK 155 13 34.58 2 766.4035

723.89 121363 73 9 11.72 EEIAMLRLELDTMK 652 14 51.33 2 846.4392

723.89 121363 73 9 11.72 Carbamidomethyl+C(4) EKLCYVALDFEQEMATAASSSSLEK 913 25 23.78 3 936.4435

723.89 121363 73 9 11.72 ANLNALDRYGR 295 11 26.01 2 631.8312

723.89 121363 73 9 11.72 Carbamidomethyl+C(19) TPENQQFPDNESEEYHRICELLSDYK 453 26 12.98 5 649.1005

sp|Q01105|SET_HUMAN 144.04 33488 18 3 12.76 VEVTEFEDIK 122 10 99.06 2 604.8079

Protein SET 144.04 33488 18 3 12.76 IDFYFDENPYFENK 136 14 20.82 2 920.9127

144.04 33488 18 3 12.76 EFHLNESGDPSSK 154 13 31.94 3 482.8858

sp|O43707|ACTN4_HUMAN 502.52 104854 59 16 20.09 VGWEQLLTTIAR 733 12 105.2 2 693.8914

Alpha-actinin-4 502.52 104854 59 16 20.09 LASDLLEWIR 300 10 64.26 2 608.34

502.52 104854 59 16 20.09 Carbamidomethyl+C(7) EGLLLWCQR 166 9 24.42 2 587.8084

502.52 104854 59 16 20.09 Carbamidomethyl+C(2) ACLISLGYDVENDR 791 14 11.84 2 812.8879

502.52 104854 59 16 20.09 TIPWLEDR 311 8 56.65 2 515.2712

502.52 104854 59 16 20.09 LSNRPAFMPSEGK 365 13 22.23 3 478.5762

502.52 104854 59 16 20.09 GYEEWLLNEIR 395 11 26.01 2 711.3539

502.52 104854 59 16 20.09 DGLAFNALIHR 193 11 26.01 3 409.5549

502.52 104854 59 16 20.09 QLEAIDQLHLEYAK 521 14 20.82 2 835.9378

502.52 104854 59 16 20.09 Carbamidomethyl+C(2) ICDQWDALGSLTHSR 497 15 27.69 3 586.9454

502.52 104854 59 16 20.09 MLDAEDIVNTARPDEK 239 16 30.44 3 606.3042

502.52 104854 59 16 20.09 EAMLK 432 5 13.9 1 591.3121

502.52 104854 59 16 20.09 VLAVNQENEHLMEDYEK 283 17 28.19 3 687.6674

502.52 104854 59 16 20.09 LEDFR 331 5 13.9 2 340.1766

502.52 104854 59 16 20.09 GISQEQMQEFR 760 11 26.01 3 451.5483

502.52 104854 59 16 20.09 MTLGMIWTIILR 140 12 31.13 3 483.279

sp|P60660|MYL6_HUMAN 164.63 16930 40 11 56.95 HVLVTLGEK 110 9 32.31 2 498.2963

Myosin light polypeptide 6 164.63 16930 40 11 56.95 ALGQNPTNAEVLK 37 13 36.35 2 677.8697

164.63 16930 40 11 56.95 VLDFEHFLPMLQTVAK 63 16 26.28 3 630.0035

164.63 16930 40 11 56.95 EAFQLFDR 13 8 51.88 2 513.2585

164.63 16930 40 11 56.95 VFDKEGNGTVMGAEIR 94 16 18.58 3 574.952

164.63 16930 40 11 56.95 DQGTYEDYVEGLR 81 13 50.25 3 515.5649

164.63 16930 40 11 56.95 VLGNPK 50 6 14.25 1 627.3829

164.63 16930 40 11 56.95 EAFQLFDRTGDGK 13 13 22.23 2 742.3647

164.63 16930 40 11 56.95 Oxidation+M(11) VFDKEGNGTVMGAEIR 94 16 38.22 2 869.9226

164.63 16930 40 11 56.95 PMLQTVAK 71 8 3.81 2 444.2539

164.63 16930 40 11 56.95 EAFQLFDR 13 8 1.04 2 504.2493

sp|P62263|RS14_HUMAN 126.42 16272 16 4 26.49 TPGPGAQSALR 106 11 59.04 2 527.7834

40S ribosomal protein S14 126.42 16272 16 4 26.49 IEDVTPIPSDSTR 128 13 48.49 2 715.3637

126.42 16272 16 4 26.49 ELGITALHIK 86 10 43.44 3 365.5532

126.42 16272 16 4 26.49 ATGGNR 98 6 14.25 1 575.2831

sp|P19338|NUCL_HUMAN 313.43 76614 46 10 15.21 EVFEDAAEIR 410 10 38.67 2 589.7903

Nucleolin 313.43 76614 46 10 15.21 NDLAVVDVR 333 9 63.12 2 500.7725

313.43 76614 46 10 15.21 ALELTGLK 362 8 68.8 2 422.7593

313.43 76614 46 10 15.21 SISLYYTGEK 457 10 57.35 2 580.7991

313.43 76614 46 10 15.21 TLVLSNLSYSATEETLQEVFEK 486 22 31.4 3 834.4307

313.43 76614 46 10 15.21 GFGFVDFNSEEDAK 610 14 26.26 2 781.3539

313.43 76614 46 10 15.21 GGRGGGGDHKPQGK 691 14 28.6 3 436.5522

313.43 76614 46 10 15.21 AGKNQGDPK 6 9 32.31 2 457.734

313.43 76614 46 10 15.21 NLPYKVTQDELK 398 12 34.14 3 483.2639

313.43 76614 46 10 15.21 SLYYTGEK 459 8 8.65 1 960.4698

sp|P62269|RS18_HUMAN 104.92 17718 24 7 39.47 IPDWFLNR 78 8 51.88 2 530.7825

40S ribosomal protein S18 104.92 17718 24 7 39.47 VLNTNIDGR 14 9 32.31 2 501.2769

104.92 17718 24 7 39.47 FQHILR 8 6 28.15 2 407.2429

104.92 17718 24 7 39.47 MSLVIPEK 0 8 34.97 2 458.762

104.92 17718 24 7 39.47 YSQVLANGLDNK 94 12 23.92 2 661.3414

104.92 17718 24 7 39.47 EDLER 108 5 13.9 1 661.3119

104.92 17718 24 7 39.47 RAGELTEDEVER 54 12 42.48 3 468.5688

sp|Q14195|DPYL3_HUMAN 250.81 61963 41 9 21.75 IFNLYPR 390 7 42.4 2 461.7611

Dihydropyrimidinase-related protein 3 250.81 61963 41 9 21.75 Carbamidomethyl+C(8) GAPLVVICQGK 440 11 39.12 2 571.3185

250.81 61963 41 9 21.75 Carbamidomethyl+C(4) FIPCSPFSDYVYK 467 13 29.71 2 811.8898

250.81 61963 41 9 21.75 MDENQFVAVTSTNAAK 374 16 25.11 2 863.4145

250.81 61963 41 9 21.75 GNVVFGEPITASLGIDGTHYWSK 270 23 44.73 3 816.7456

250.81 61963 41 9 21.75 GSPTRPNPPVR 520 11 26.01 2 589.328

250.81 61963 41 9 21.75 ISVGSDSDLVIWDPDAVK 400 18 26.81 2 958.4916

250.81 61963 41 9 21.75 Carbamidomethyl+C(10) AITIASQTNCPLYVTK 238 16 18.58 2 890.4709

250.81 61963 41 9 21.75 GGTPAGSAR 511 9 60.01 2 387.1972

sp|A5A3E0|POTEF_HUMAN 608.91 121445 51 7 7.63 AGFAGDDAPR 718 10 120.85 2 488.7225

POTE ankyrin domain family member F 608.91 121445 51 7 7.63 SYELPDGQVITIGNER 938 16 192.16 2 895.9431

608.91 121445 51 7 7.63 IWHHTFYNELR 784 11 43.89 3 505.9199

608.91 121445 51 7 7.63 DLIVMLRDTDVNK 155 13 34.58 2 766.4035

608.91 121445 51 7 7.63 DILHENSTLR 642 10 28.67 2 599.3186

608.91 121445 51 7 7.63 ILTEHGYR 891 8 37.98 2 494.759

608.91 121445 51 7 7.63 VVEVDSMPAASSVK 1 14 26.26 3 473.5792

sp|P46781|RS9_HUMAN 115.73 22591 9 3 10.31 LFEGNALLR 70 9 57 2 516.7958

40S ribosomal protein S9 115.73 22591 9 3 10.31 IEDFLER 101 7 42.4 2 461.238

115.73 22591 9 3 10.31 VLIR 127 4 13.56 1 500.3556

sp|Q07021|C1QBP_HUMAN 101.04 31362 22 7 16.67 AFVDFLSDEIKEER 80 14 46.97 3 566.6128

Complement component 1 Q 101.04 31362 22 7 16.67 Carbamidomethyl+C(6) ALVLDCHYPEDEVGQEDEAESDIFSIR 180 27 33.35 3 1046.1415

subcomponent-binding protein 101.04 31362 22 7 16.67 LPLLR 1 5 27.81 1 611.4273

101.04 31362 22 7 16.67 Carbamidomethyl+C(7) KALVLDCHYPEDEVGQEDEAESDIFSIR 179 28 18.44 5 653.7015

101.04 31362 22 7 16.67 Carbamidomethyl+C(6) ALVLDCHYPEDEVG 180 14 7.62 3 539.5813

101.04 31362 22 7 16.67 VDFLSDEIKEER 82 12 28.6 2 740.3726

101.04 31362 22 7 16.67 DFLSDEIKEER 83 11 7.48 2 690.8365

sp|P05388|RLA0_HUMAN 143.04 34273 26 4 20.19 IIQLLDDYPK 16 10 57.35 2 609.3392

60S acidic ribosomal protein P0 143.04 34273 26 4 20.19 TSFFQALGITTK 134 12 72.63 2 657.3541

143.04 34273 26 4 20.19 GHLENNPALEK 66 11 29.02 2 611.3247

143.04 34273 26 4 20.19 AFLADPSAFVAAAPVAAATTAAPAAAA 266 31 61.17 3 918.1602

APAK

sp|P63241|IF5A1_HUMAN 140.64 16832 19 4 24.68 IVEMSTSK 39 8 56.65 2 447.7308

Eukaryotic translation initiation factor 5A-1 140.64 16832 19 4 24.68 NGFVVLK 27 7 39.39 2 388.7354

140.64 16832 19 4 24.68 NDFQLIGIQDGYLSLLQDSGEVR 86 23 38.38 3 860.773

140.64 16832 19 4 24.68 VEMSTSK 40 7 1.04 1 781.3748

sp|P48643|TCPE_HUMAN 187.39 59671 22 6 12.2 ISDSVLVDIKDTEPLIQTAK 150 20 21.63 3 729.068

T-complex protein 1 subunit epsilon 187.39 59671 22 6 12.2 IADGYEQAAR 132 10 41.78 2 547.2706

187.39 59671 22 6 12.2 Carbamidomethyl+C(7) SLHDALCVIR 400 10 38.67 2 592.3158

187.39 59671 22 6 12.2 QQISLATQMVR 514 11 26.01 2 637.8478

187.39 59671 22 6 12.2 TSLGPNGLDK 49 10 28.67 2 501.2638

187.39 59671 22 6 12.2 TSLGPNGLDKMMVDK 49 15 19.61 3 535.9334

sp|P43243|MATR3_HUMAN 220.99 94623 38 9 14.17 IGPYQPNVPVGIDYVIPK 780 18 25.8 3 657.0388

Matrin-3 220.99 94623 38 9 14.17 ITPENLPQILLQLK 132 14 45.31 2 810.4945

220.99 94623 38 9 14.17 GPSLNPVLDYDHGSR 192 15 31.6 3 542.9384

220.99 94623 38 9 14.17 GDADQASNILASFGLSAR 102 18 28.57 2 896.9475

220.99 94623 38 9 14.17 GIDLLK 582 6 28.15 1 658.4081

220.99 94623 38 9 14.17 EWSQHINGASHSR 304 13 27 3 503.5733

220.99 94623 38 9 14.17 GNLGAGNGNLQGPR 373 14 20.82 2 662.8462

220.99 94623 38 9 14.17 Carbamidomethyl+C(8) ALWFQGRCVK 555 10 28.67 2 632.8304

220.99 94623 38 9 14.17 RTEEGPTLSYGR 148 12 39.59 2 683.3356

sp|P84103|SRSF3_HUMAN 108.38 19329 16 6 29.27 AFGYYGPLR 28 9 47.87 2 522.27

Serine/arginine-rich splicing factor 3 108.38 19329 16 6 29.27 NPPGFAFVEFEDPRDAADAVR 43 21 15 3 774.0369

108.38 19329 16 6 29.27 VYVGNLGNNGNK 11 12 39.59 2 624.821

108.38 19329 16 6 29.27 SVWVAR 37 6 14.25 2 359.2035

108.38 19329 16 6 29.27 GYYGPLR 30 7 1.39 1 825.4202

108.38 19329 16 6 29.27 PRDAADAVR 55 9 7.41 2 485.7541

**Mock_2**

**Protein score^b^ avg Matched Matched seq peptide seq^h^ seq seq score^m^ z^n^ mz^o^**

**Name^a^ Mass^c^ Products^d^ Peptides^e^ Cover(%)^f^  modification^g^ Start^i^ Length^l^**

sp|P07737|PROF1_HUMAN Profilin-1 646.15 15054 79 9 62.86 STGGAPTFNVTVTK 91 14 179.63 2 690.3596

646.15 15054 79 9 62.86 TFVNITPAEVGVLVGK 38 16 218.15 2 822.4719

646.15 15054 79 9 62.86 DSPSVWAAVPGK 26 12 87.02 2 607.3139

646.15 15054 79 9 62.86 TLVLLMGK 108 8 42.68 2 437.7717

646.15 15054 79 9 62.86 SSFYVNGLTLGGQK 56 14 72.79 2 735.8818

646.15 15054 79 9 62.86 EGVHGGLINK 116 10 28.6 2 512.2819

646.15 15054 79 9 62.86 DSLLQDGEFSMDLR 75 14 57.75 2 813.3816

646.15 15054 79 9 62.86 PSVWAAVPGK 28 10 31.06 2 506.2837

646.15 15054 79 9 62.86 PTFNVTVTK 96 9 16.42 1 1006.5628

sp|P60174|TPIS_HUMAN 871.45 26669 145 20 76.71 VVLAYEPVWAIGTGK 160 15 215.51 2 801.9489

Triosephosphate isomerase 871.45 26669 145 20 76.71 Carbamidomethyl+C(12) IIYGGSVTGATCK 206 13 113.8 2 663.8387

871.45 26669 145 20 76.71 Carbamidomethyl+C(2) DCGATWVVLGHSER 85 14 99.84 3 529.5794

871.45 26669 145 20 76.71 VVFEQTK 142 7 42.33 2 425.7344

871.45 26669 145 20 76.71 HVFGESDELIGQK 100 13 119 3 486.908

871.45 26669 145 20 76.71 QSLGELIGTLNAAK 19 14 76.47 2 707.9008

871.45 26669 145 20 76.71 VTNGAFTGEISPGMIK 69 16 66.3 2 811.4184

871.45 26669 145 20 76.71 Carbamidomethyl+C(14) VAHALAEGLGVIACIGEK 113 18 151.21 3 603.3248

871.45 26669 145 20 76.71 Carbamidomethyl+C(8) IAVAAQNCYK 59 10 28.6 3 379.8587

871.45 26669 145 20 76.71 Carbamidomethyl+C(9) VPADTEVVCAPPTAYIDFAR 33 20 138.48 3 731.3579

871.45 26669 145 20 76.71 ELASQPDVDGFLVGGASLKPEFVDIINAK 219 29 87.56 3 1010.5335

871.45 26669 145 20 76.71 FFVGGNWK 6 8 42.68 2 477.7386

871.45 26669 145 20 76.71 KQSLGELIGTLNAAK 18 15 63.98 3 514.9626

871.45 26669 145 20 76.71 RHVFGESDELIGQK 99 14 41.82 3 538.9432

871.45 26669 145 20 76.71 VIADNVKDWSK 149 11 55.91 2 637.8408

871.45 26669 145 20 76.71 KFFVGGNWK 5 9 32.23 2 541.7931

871.45 26669 145 20 76.71 GATWVVLGHSER 87 12 7.47 2 656.3468

871.45 26669 145 20 76.71 Carbamidomethyl+C(14) VAHALAEGLGVIACIGE 113 17 4.53 3 560.6274

871.45 26669 145 20 76.71 Carbamidomethyl+C(1) CGATWVVLGHSER 86 13 3.14 3 491.2446

871.45 26669 145 20 76.71 LAYEPVWAIGTGK 162 13 21.7 2 702.8807

sp|P62937|PPIA_HUMAN 769.72 18012 117 17 69.7 FEDENFILK 82 9 93.76 2 577.7858

Peptidyl-prolyl cis-trans isomerase A 769.72 18012 117 17 69.7 VSFELFADK 19 9 92.51 2 528.2704

769.72 18012 117 17 69.7 Carbamidomethyl+C(7) IIPGFMCQGGDFTR 55 14 114.15 2 799.8774

769.72 18012 117 17 69.7 EGMNIVEAMER 133 11 106.11 2 639.7955

769.72 18012 117 17 69.7 VNPTVFFDIAVDGEPLGR 1 18 179.33 2 973.5108

769.72 18012 117 17 69.7 Carbamidomethyl+C(24) HTGPGILSMANAGPNTNGSQFFICTAK 91 27 156.64 3 931.1125

769.72 18012 117 17 69.7 VKEGMNIVEAMER 131 13 64.59 2 753.3786

769.72 18012 117 17 69.7 Carbamidomethyl+C(7) KITIADCGQLE 154 11 32.93 2 624.3177

769.72 18012 117 17 69.7 SIYGEKFEDENFILK 76 15 58.96 3 611.3055

769.72 18012 117 17 69.7 HNGTGGKSIYGEK 69 13 26.94 2 674.3386

769.72 18012 117 17 69.7 MVNPTVFFDIAVDGEPLGRVSFELFADK 0 28 12.4 4 779.1544

769.72 18012 117 17 69.7 Oxidation+M(11) VKEGMNIVEAMER 131 13 26.94 3 507.9223

769.72 18012 117 17 69.7 FELFADK 21 7 24.38 1 869.4338

769.72 18012 117 17 69.7 Carbamidomethyl+C(5) PGFMCQGGDFTR 57 12 7.47 2 686.7963

769.72 18012 117 17 69.7 DENFILK 84 7 24.38 1 878.4562

769.72 18012 117 17 69.7 SFELFADK 20 8 40.01 1 956.475

769.72 18012 117 17 69.7 VSFELFADK 19 9 1.39 2 519.2685

sp|P22626|ROA2_HUMAN 839.13 37429 116 20 40.23 IDTIEIITDR 137 10 135.74 2 594.8257

Heterogeneous nuclear ribonucleoproteins A2/B1 839.13 37429 116 20 40.23 GGGGNFGPGPGSNFR 213 15 109.03 2 689.3189

839.13 37429 116 20 40.23 GFGFVTFDDHDPVDK 153 15 113.6 3 565.9231

839.13 37429 116 20 40.23 LFIGGLSFETTEESLR 22 16 181.57 2 899.9679

839.13 37429 116 20 40.23 YHTINGHNAEVR 173 12 37.08 3 470.8965

839.13 37429 116 20 40.23 NYYEQWGK 38 8 42.68 2 544.2478

839.13 37429 116 20 40.23 GGNFGFGDSR 203 10 54.24 2 507.2263

839.13 37429 116 20 40.23 LFVGGIK 113 7 56.2 2 367.2306

839.13 37429 116 20 40.23 QEMQEVQSSR 190 10 57.25 2 611.2798

839.13 37429 116 20 40.23 TLETVPLER 3 9 98.53 2 529.2972

839.13 37429 116 20 40.23 NMGGPYGGGNYGPGGSGGSGGYGGR 325 25 23.75 2 1095.466

839.13 37429 116 20 40.23 RGFGFVTFDDHDPVDK 152 16 18.52 3 617.9596

839.13 37429 116 20 40.23 ALSRQEMQEVQSSR 186 14 28.53 3 550.2745

839.13 37429 116 20 40.23 GFVTFDDHDPVDK 155 13 27.63 2 746.3441

839.13 37429 116 20 40.23 VTFDDHDPVDK 157 11 15.68 2 644.301

839.13 37429 116 20 40.23 TFDDHDPVDK 158 10 27.63 2 594.7665

839.13 37429 116 20 40.23 FVTFDDHDPVDK 156 12 3.49 2 717.8373

839.13 37429 116 20 40.23 TIEIITDR 139 8 8.63 2 480.7691

839.13 37429 116 20 40.23 FGFVTFDDHDPVDK 154 14 7.36 3 546.9247

839.13 37429 116 20 40.23 GFGFVTFDDHDPVDK 153 15 3.49 3 559.9225

sp|P23528|COF1_HUMAN Cofilin-1 463.57 18502 88 8 59.04 YALYDATYETK 81 11 133.87 2 669.315

463.57 18502 88 8 59.04 LGGSAVISLEGKPL 152 14 105.77 2 670.8929

463.57 18502 88 8 59.04 Carbamidomethyl+C(7) HELQANCYEEVKDR 132 14 92.69 3 597.6062

463.57 18502 88 8 59.04 Carbamidomethyl+C(7) MLPDKDCR 73 8 56.55 2 517.7419

463.57 18502 88 8 59.04 Carbamidomethyl+C(5) AVLFCLSEDK 34 10 30.36 3 394.5304

463.57 18502 88 8 59.04 EILVGDVGQTVDDPYATFVK 53 20 41.99 2 1083.5586

463.57 18502 88 8 59.04 MASGVAVSDGVIK 0 13 34.49 2 617.323

463.57 18502 88 8 59.04 NIILEEGKEILVGDVGQTVDDPYATFVK 45 28 349.1 3 1021.5367

sp|P62805|H4_HUMAN Histone H4 399.59 11367 52 13 51.46 ISGLIYEETR 46 10 129.64 2 590.8123

399.59 11367 52 13 51.46 VFLENVIR 60 8 79.53 2 495.2885

399.59 11367 52 13 51.46 DNIQGITKPAIR 24 12 69.47 3 442.5843

399.59 11367 52 13 51.46 DAVTYTEHAK 68 10 71.13 2 567.7748

399.59 11367 52 13 51.46 TVTAMDVVYALKR 80 13 61.38 3 489.6012

399.59 11367 52 13 51.46 TVTAMDVVYALK 80 12 31.06 2 655.8575

399.59 11367 52 13 51.46 DNIQGITKPAIRR 24 13 29.65 2 781.4138

399.59 11367 52 13 51.46 QGITKPAIR 27 9 2.44 2 492.3002

399.59 11367 52 13 51.46 TAMDVVYALKR 82 11 2.79 2 633.8481

399.59 11367 52 13 51.46 TYTEHAK 71 7 1.74 2 425.2074

399.59 11367 52 13 51.46 IQGITKPAIR 26 10 2.44 2 548.8464

399.59 11367 52 13 51.46 GITKPAIR 28 8 2.44 2 428.2726

399.59 11367 52 13 51.46 FLENVIR 61 7 10.15 2 445.7552

sp|P0C7M2|RA1L3_HUMAN 475.89 34223 57 8 22.19 IEVIEIMTDR 130 10 120.61 2 609.8217

Putative heterogeneous 475.89 34223 57 8 22.19 GFAFVTFDDHDSVDK 146 15 146.13 3 567.2547

nuclear ribonucleoprotein A1-like 3 475.89 34223 57 8 22.19 LFIGGLSFETTDESLR 15 16 110.53 2 892.9609

475.89 34223 57 8 22.19 GFGFVTYATVEEVDAAMNARPHK 55 23 68.15 4 628.3043

475.89 34223 57 8 22.19 IFVGGIK 106 7 73.09 2 367.2323

475.89 34223 57 8 22.19 Oxidation+M() GFGFVTYATVEEVDAAMNARPHK 55 23 23.05 4 632.3123

475.89 34223 57 8 22.19 VTFDDHDSVDK 150 11 7.36 2 639.2909

475.89 34223 57 8 22.19 VIEIMTDR 132 8 1.74 1 976.5039

sp|P62258|1433E_HUMAN 14-3-3 protein epsilon 436.23 29173 78 15 42.35 NLLSVAYK 42 8 79.53 2 454.2623

436.23 29173 78 15 42.35 Carbamidomethyl+C(3);

Carbamidomethyl+C(4) LICCDILDVLDK 94 12 131.2 2 738.8772

436.23 29173 78 15 42.35 YLAEFATGNDRK 130 12 85.7 3 462.2301

436.23 29173 78 15 42.35 DSTLIMQLLR 215 10 92.86 2 595.3359

436.23 29173 78 15 42.35 AAFDDAIAELDTLSEESYK 196 19 16.13 2 1044.4928

436.23 29173 78 15 42.35 YLAEFATGNDR 130 11 40.61 2 628.7917

436.23 29173 78 15 42.35 VAGMDVELTVEER 29 13 36.25 2 724.3699

436.23 29173 78 15 42.35 VFYYK 118 5 27.75 2 360.1893

436.23 29173 78 15 42.35 HLIPAANTGESK 106 12 69.47 3 413.216

436.23 29173 78 15 42.35 NVIGAR 50 6 31.11 1 629.3727

436.23 29173 78 15 42.35 EAAENSLVAYK 142 11 39.02 2 597.8138

436.23 29173 78 15 42.35 Carbamidomethyl+C(3);

Carbamidomethyl+C(4) LICCDILDVLDKHLIPAANTGESK 94 24 44.54 4 674.5951

436.23 29173 78 15 42.35 YLAEFATGNDRK 130 12 23.86 2 692.8507

436.23 29173 78 15 42.35 AEFATGNDRK 132 10 18.43 2 554.7745

436.23 29173 78 15 42.35 LLSVAYK 43 7 1.05 2 397.2427

sp|P16949|STMN1_HUMAN Stathmin 277.63 17302 38 9 39.6 ASGQAFELILSPR 14 13 123.77 2 694.8774

277.63 17302 38 9 39.6 AIEENNNFSK 85 10 71.13 2 583.2773

277.63 17302 38 9 39.6 DLSLEEIQK 43 9 40.01 2 537.7875

277.63 17302 38 9 39.6 SKESVPEFPLSPPK 27 14 29.33 3 514.6095

277.63 17302 38 9 39.6 QLAEK 70 5 13.88 1 588.3384

277.63 17302 38 9 39.6 LEAAEERR 53 8 56.55 2 487.2519

277.63 17302 38 9 39.6 PEFPLSPPK 32 9 16.42 2 506.2789

277.63 17302 38 9 39.6 SLEEIQK 45 7 24.38 1 846.4504

277.63 17302 38 9 39.6 EENNNFSK 87 8 1.74 1 981.4264

sp|P63104|1433Z_HUMAN 14-3-3 457.82 27745 54 10 32.24 NLLSVAYK 41 8 79.53 2 454.2623

protein zeta/delta 457.82 27745 54 10 32.24 Carbamidomethyl+C(3) DICNDVLSLLEK 91 12 88.11 2 709.8665

457.82 27745 54 10 32.24 FLIPNASQAESK 103 12 72.48 2 652.8467

457.82 27745 54 10 32.24 YLAEVAAGDDKK 127 12 50.16 3 427.2195

457.82 27745 54 10 32.24 DSTLIMQLLR 212 10 92.86 2 595.3359

457.82 27745 54 10 32.24 SVTEQGAELSNEER 27 14 36.32 2 774.8648

457.82 27745 54 10 32.24 VFYLK 115 5 27.75 2 335.1986

457.82 27745 54 10 32.24 IETELR 85 6 28.1 2 380.7096

457.82 27745 54 10 32.24 YLAEVAAGDDK 127 11 28.95 3 384.5242

457.82 27745 54 10 32.24 PNASQAESK 106 9 7.87 1 931.4518

sp|P30086|PEBP1_HUMAN 250.66 21056 22 3 19.79 LYTLVLTDPDAPSR 62 14 71.03 2 780.9133

Phosphatidylethanolamine-binding protein 1 250.66 21056 22 3 19.79 VLTPTQVK 39 8 56.55 2 443.2705

250.66 21056 22 3 19.79 NRPTSISWDGLDSGK 47 15 106.33 3 544.9397

sp|P67936|TPM4_HUMAN 323.49 28521 49 8 29.44 IQLVEEELDR 55 10 118.85 2 622.3266

Tropomyosin alpha-4 chain 323.49 28521 49 8 29.44 LVILEGELER 132 10 61.13 2 585.8384

323.49 28521 49 8 29.44 IQALQQQADEAEDR 13 14 32.87 2 807.8946

323.49 28521 49 8 29.44 MEIQEMQLK 104 9 47.87 2 575.2898

323.49 28521 49 8 29.44 AEGDVAALNR 44 10 42.48 2 508.2641

323.49 28521 49 8 29.44 AGLNSLEAVK 1 10 50.26 2 501.2797

323.49 28521 49 8 29.44 TIDDLEEK 215 8 34.89 2 481.7359

323.49 28521 49 8 29.44 EKAEGDVAALNR 42 12 36.49 2 636.8296

sp|P25398|RS12_HUMAN 188.16 14515 36 9 54.55 Carbamidomethyl+C(6) LVEALCAEHQINLIK 63 15 43.55 3 584.3178

40S ribosomal protein S12 188.16 14515 36 9 54.55 Carbamidomethyl+C(8) LGEWVGLCK 84 9 79.88 2 531.2763

188.16 14515 36 9 54.55 TALIHDGLAR 23 10 54.24 2 533.7983

188.16 14515 36 9 54.55 DVIEEYFK 121 8 51.78 2 521.7585

188.16 14515 36 9 54.55 Carbamidomethyl+C(4);

Carbamidomethyl+C(6) VVGCSCVVVK 102 10 28.6 2 553.7878

188.16 14515 36 9 54.55 Carbamidomethyl+C(5);

Carbamidomethyl+C(11) QAHLCVLASNCDEPMYVK 45 18 34.48 3 712.3371

188.16 14515 36 9 54.55 Carbamidomethyl+C(9) DVIEEYFKCK 121 10 30.36 3 444.2153

188.16 14515 36 9 54.55 Carbamidomethyl+C(4) EALCAEHQINLIK 65 13 15.68 2 769.9062

188.16 14515 36 9 54.55 Carbamidomethyl+C(5) VEALCAEHQINLIK 64 14 3.49 2 819.4337

sp|P10809|CH60_HUMAN 766.05 61054 115 24 39.44 VGLQVVAVK 292 9 93.76 2 456.7949

60 kDa heat shock protein 766.05 61054 115 24 39.44 LSDGVAVLK 396 9 56.9 2 451.2695

766.05 61054 115 24 39.44 VGEVIVTK 344 8 56.55 2 422.7584

766.05 61054 115 24 39.44 TVIIEQSWGSPK 60 12 52.58 2 672.8634

766.05 61054 115 24 39.44 ISSIQSIVPALEIANAHR 250 18 43.51 3 640.3586

766.05 61054 115 24 39.44 VTDALNATR 420 9 59.91 2 480.7576

766.05 61054 115 24 39.44 VGGTSDVEVNEK 405 12 102.59 2 617.3014

766.05 61054 115 24 39.44 TLNDELEIIEGMK 205 13 58.05 2 752.886

766.05 61054 115 24 39.44 IGIEIIK 462 7 39.32 2 393.2566

766.05 61054 115 24 39.44 IQEIIEQLDVTTSEYEKEK 370 19 16.13 3 765.7231

766.05 61054 115 24 39.44 GVMLAVDAVIAELKK 142 15 49.65 3 519.6372

766.05 61054 115 24 39.44 Carbamidomethyl+C(13) AAVEEGIVLGGGCALLR 429 17 72.61 2 842.9568

766.05 61054 115 24 39.44 GIIDPTK 516 7 42.33 1 743.4379

766.05 61054 115 24 39.44 LVQDVANNTNEEAGDGTTTATVLAR 96 25 13.26 3 854.091

766.05 61054 115 24 39.44 ALMLQGVDLLADAVAVTMGPK 37 21 85.01 3 705.0453

766.05 61054 115 24 39.44 NAGVEGSLIVEK 481 12 34.07 2 608.3326

766.05 61054 115 24 39.44 SIDLK 82 5 27.75 1 575.3377

766.05 61054 115 24 39.44 APGFGDNR 301 8 56.55 2 417.2046

766.05 61054 115 24 39.44 MLAVDAVIAELKK 144 13 7.36 2 700.9129

766.05 61054 115 24 39.44 GEVIVTK 345 7 1.05 1 745.4421

766.05 61054 115 24 39.44 SDGVAVLK 397 8 1.39 1 788.4504

766.05 61054 115 24 39.44 GLQVVAVK 293 8 9.25 1 813.5217

766.05 61054 115 24 39.44 LAVDAVIAELKK 145 12 7.36 2 635.3922

766.05 61054 115 24 39.44 AVDAVIAELKK 146 11 21.7 2 578.8541

sp|P27348|1433T_HUMAN 14-3-3 protein theta 353.85 27764 47 8 35.51 NLLSVAYK 41 8 79.53 2 454.2623

353.85 27764 47 8 35.51 Carbamidomethyl+C(3) SICTTVLELLDK 91 12 102.66 2 696.3772

353.85 27764 47 8 35.51 DSTLIMQLLR 212 10 92.86 2 595.3359

353.85 27764 47 8 35.51 YLIANATNPESK 103 12 53.07 2 660.8444

353.85 27764 47 8 35.51 Carbamidomethyl+C(7) YLAEVACGDDRK 127 12 23.86 3 466.2225

353.85 27764 47 8 35.51 AVTEQGAELSNEER 27 14 50.86 2 766.8656

353.85 27764 47 8 35.51 TAFDEAIAELDTLNEDSYK 193 19 25.5 3 715.6762

353.85 27764 47 8 35.51 NLLSVAYK 41 8 1.05 2 445.7545

sp|P11021|GRP78_HUMAN 652.93 72333 113 23 35.47 VEIIANDQGNR 49 11 116.98 2 614.8165

78 kDa glucose-regulated protein 652.93 72333 113 23 35.47 IINEPTAAAIAYGLDK 197 16 90.08 2 830.4546

652.93 72333 113 23 35.47 VTHAVVTVPAYFNDAQR 164 17 81.56 3 629.9946

652.93 72333 113 23 35.47 IEWLESHQDADIEDFK 601 16 55.22 3 658.9728

652.93 72333 113 23 35.47 NELESYAYSLK 562 11 39.02 2 658.8279

652.93 72333 113 23 35.47 NQLTSNPENTVFDAK 81 15 58.96 2 839.4104

652.93 72333 113 23 35.47 TFAPEEISAMVLTK 138 14 45.2 2 768.9046

652.93 72333 113 23 35.47 ELEEIVQPIISK 621 12 39.5 2 699.3996

652.93 72333 113 23 35.47 ITPSYVAFTPEGER 60 14 46.88 2 783.9004

652.93 72333 113 23 35.47 VMEHFIK 261 7 39.32 2 452.2387

652.93 72333 113 23 35.47 TKPYIQVDIGGGQTK 123 15 27.87 3 535.6227

652.93 72333 113 23 35.47 TWNDPSVQQDIK 101 12 31.06 3 477.5669

652.93 72333 113 23 35.47 VYEGERPLTK 464 10 38.6 3 397.877

652.93 72333 113 23 35.47 ITITNDQNR 523 9 40.01 2 537.7776

652.93 72333 113 23 35.47 LSSEDK 585 6 14.23 1 678.3376

652.93 72333 113 23 35.47 VTAEDK 510 6 14.23 1 662.3298

652.93 72333 113 23 35.47 VVEK 118 4 13.53 1 474.2881

652.93 72333 113 23 35.47 DAGTIAGLNVMR 185 12 38.4 2 609.3182

652.93 72333 113 23 35.47 FLPFK 113 5 13.88 2 326.1947

652.93 72333 113 23 35.47 ITPSYVAFTPEGERLIGDAAK 60 21 14.95 3 745.7234

652.93 72333 113 23 35.47 AKFEELNMDLFR 324 12 75.77 3 504.9206

652.93 72333 113 23 35.47 NQLTSNPENTVFDAKR 81 16 25.05 2 917.4562

652.93 72333 113 23 35.47 PAYFNDAQR 172 9 4.18 2 541.2602

sp|P35268|RL22_HUMAN 148.3 14787 20 6 31.25 ITVTSEVPFSK 69 11 74.56 2 604.3329

60S ribosomal protein L22 148.3 14787 20 6 31.25 AGNLGGGVVTIER 52 13 36.25 2 621.8452

148.3 14787 20 6 31.25 VVANSKESYELR 101 12 36.49 3 465.585

148.3 14787 20 6 31.25 QVLK 16 4 13.53 1 487.3254

148.3 14787 20 6 31.25 VVANSK 101 6 28.1 1 617.3642

148.3 14787 20 6 31.25 ESYELR 107 6 14.23 1 796.3774

sp|P61981|1433G_HUMAN 288.18 28302 45 8 32.39 NLLSVAYK 42 8 79.53 2 454.2623

14-3-3 protein gamma 288.18 28302 45 8 32.39 YLAEVATGEK 132 10 35.59 2 540.7824

288.18 28302 45 8 32.39 AYSEAHEISK 152 10 38.6 2 567.7787

288.18 28302 45 8 32.39 DSTLIMQLLR 217 10 92.86 2 595.3359

288.18 28302 45 8 32.39 NVTELNEPLSNEER 28 14 43.87 2 822.4012

288.18 28302 45 8 32.39 ATVVESSEK 143 9 32.23 2 475.2439

288.18 28302 45 8 32.39 Carbamidomethyl+C(6) ELEAVCQDVLSLLDNYLIK 91 19 79.54 3 745.7197

288.18 28302 45 8 32.39 SEAHEISK 154 8 1.74 2 450.7239

sp|P09936|UCHL1_HUMAN 350.61 24824 51 11 33.63 LGFEDGSVLK 105 10 71.13 2 532.7831

Ubiquitin carboxyl-terminal hydrolase isozyme L1 350.61 24824 51 11 33.63 Carbamidomethyl+C(17) NEAIQAAHDAVAQEGQCR 135 18 144.22 3 656.6356

350.61 24824 51 11 33.63 LGVAGQWR 19 8 34.89 2 443.7517

350.61 24824 51 11 33.63 Carbamidomethyl+C(7) FSAVALCK 213 8 70.43 2 448.2382

350.61 24824 51 11 33.63 MQLKPMEINPEMLNK 0 15 25.57 3 605.976

350.61 24824 51 11 33.63 QLKPMEINPEMLNK 1 14 26.19 2 842.9361

350.61 24824 51 11 33.63 QIEELK 65 6 28.1 1 759.4189

350.61 24824 51 11 33.63 VYFMK 78 5 13.88 2 344.1792

350.61 24824 51 11 33.63 EFTER 202 5 13.88 2 341.1619

350.61 24824 51 11 33.63 Carbamidomethyl+C(15) AIQAAHDAVAQEGQCR 137 16 4.53 2 862.912

350.61 24824 51 11 33.63 Carbamidomethyl+C(7) FSAVALCK 213 8 1.05 2 439.2333

sp|P31946|1433B_HUMAN 240.96 28082 29 4 16.67 NLLSVAYK 43 8 79.53 2 454.2623

14-3-3 protein beta/alpha 240.96 28082 29 4 16.67 DSTLIMQLLR 214 10 92.86 2 595.3359

240.96 28082 29 4 16.67 YLIPNATQPESK 105 12 34.42 2 680.8633

240.96 28082 29 4 16.67 YLSEVASGDNK 129 11 37.6 2 591.7892

sp|Q99497|PARK7_HUMAN Protein DJ-1 176.96 19891 30 11 61.38 Carbamidomethyl+C(5) DVVICPDASLEDAKK 48 15 79.76 3 553.9444

176.96 19891 30 11 61.38 Carbamidomethyl+C(14) VTVAGLAGKDPVQCSR 32 16 26.22 3 553.2929

176.96 19891 30 11 61.38 EGPYDVVVLPGGNLGAQNLSESAAVK 63 26 19.09 3 862.1129

176.96 19891 30 11 61.38 Carbamidomethyl+C(7) GLIAAICAGPTALLAHEIGFGSK 99 23 39.55 3 756.4054

176.96 19891 30 11 61.38 EILK 89 4 13.53 1 502.3224

176.96 19891 30 11 61.38 APLVLK 182 6 14.23 2 320.7209

176.96 19891 30 11 61.38 ALVILAK 5 7 42.33 2 364.2556

176.96 19891 30 11 61.38 GAEEMETVIPVDVMR 12 15 36.09 2 838.4163

176.96 19891 30 11 61.38 AGIK 28 4 13.53 1 388.2529

176.96 19891 30 11 61.38 Carbamidomethyl+C(3) VICPDASLEDAKK 50 13 3.49 2 723.3705

176.96 19891 30 11 61.38 Carbamidomethyl+C(2) ICPDASLEDAKK 51 12 3.49 2 673.8391

sp|P05387|RLA2_HUMAN 60S acidic 125.29 11664 25 4 69.57 LASVPAGGAVAVSAAPGSAAPAAGSAPAAAEEK 61 33 42.8 3 925.4831 ribosomal protein P2 125.29 11664 25 4 69.57 YVASYLLAALGGNSSPSAK 2 19 57.14 2 934.9988

125.29 11664 25 4 69.57 NIEDVIAQGIGK 49 12 50.06 2 628.8489

125.29 11664 25 4 69.57 ILDSVGIEADDDRLNK 25 16 56.98 3 591.6364

sp|Q6NXT2|H3C_HUMAN Histone H3.3C 116.87 15213 23 6 31.85 YRPGTVALR 40 9 63 2 516.8011

116.87 15213 23 6 31.85 STELLIR 56 7 53.19 2 416.2466

116.87 15213 23 6 31.85 DIQLAR 122 6 41.98 2 358.2065

116.87 15213 23 6 31.85 LPFQR 64 5 13.88 1 660.3838

116.87 15213 23 6 31.85 EIAQDFNTDLR 72 11 28.95 3 441.22

116.87 15213 23 6 31.85 QLATK 19 5 13.88 1 560.3338

sp|P30101|PDIA3_HUMAN 499.23 56782 97 23 40.99 YGVSGYPTLK 94 10 64.14 2 542.7928

Protein disulfide-isomerase A3 499.23 56782 97 23 40.99 LAPEYEAAATR 62 11 52.83 2 596.3058

499.23 56782 97 23 40.99 ELSDFISYLQR 471 11 39.02 2 685.8537

499.23 56782 97 23 40.99 FVMQEEFSR 335 9 40.01 2 586.7804

499.23 56782 97 23 40.99 LNFAVASR 296 8 70.43 2 439.2462

499.23 56782 97 23 40.99 EATNPPVIQEEKPK 482 14 29.33 3 527.2785

499.23 56782 97 23 40.99 TFSHELSDFGLESTAGEIPVVAIR 305 24 98.99 3 859.1097

499.23 56782 97 23 40.99 TADGIVSHLK 119 10 28.6 2 520.7891

499.23 56782 97 23 40.99 GFPTIYFSPANK 448 12 23.86 2 671.3495

499.23 56782 97 23 40.99 GIVPLAK 75 7 42.33 2 349.2326

499.23 56782 97 23 40.99 DASIVGFFDDSFSEAHSEFLK 152 21 32.15 3 783.3646

499.23 56782 97 23 40.99 SEPIPESNDGPVK 366 13 45.94 2 684.8387

499.23 56782 97 23 40.99 ALER 347 4 27.4 1 488.278

499.23 56782 97 23 40.99 DLLIAYYDVDYEK 258 13 29.65 2 810.3954

499.23 56782 97 23 40.99 LAAASDVLELTDDNFESR 20 18 16.83 2 983.4849

499.23 56782 97 23 40.99 TVAYTEQK 218 8 53.54 2 470.249

499.23 56782 97 23 40.99 TVAYTEQKMTSGK 218 13 22.17 3 481.9134

499.23 56782 97 23 40.99 NRVMMVAK 280 8 42.68 2 474.7596

499.23 56782 97 23 40.99 KTFSHELSDFGLESTAGEIPVVAIR 304 25 20.35 3 901.7959

499.23 56782 97 23 40.99 PEYEAAATR 64 9 8.19 2 504.2463

499.23 56782 97 23 40.99 PPVIQEEKPK 486 10 21.86 2 582.8334

499.23 56782 97 23 40.99 APEYEAAATR 63 10 8.19 2 539.764

499.23 56782 97 23 40.99 ELSDFISYLQR 471 11 2.09 2 676.8435

sp|P60660|MYL6_HUMAN 184.58 16930 36 8 44.37 HVLVTLGEK 110 9 47.87 2 498.2975

Myosin light polypeptide 6 184.58 16930 36 8 44.37 ALGQNPTNAEVLK 37 13 43.52 2 677.8705

184.58 16930 36 8 44.37 EAFQLFDR 13 8 56.55 2 513.256

184.58 16930 36 8 44.37 VLDFEHFLPMLQTVAK 63 16 44.35 3 630.0027

184.58 16930 36 8 44.37 VFDKEGNGTVMGAEIR 94 16 10.76 3 574.9507

184.58 16930 36 8 44.37 EAFQLFDRTGDGK 13 13 36.25 3 495.2454

184.58 16930 36 8 44.37 Oxidation+M(11) VFDKEGNGTVMGAEIR 94 16 26.28 2 869.9178

184.58 16930 36 8 44.37 PMLQTVAK 71 8 3.84 2 444.2531

sp|P49458|SRP09_HUMAN 87.42 10111 13 4 36.05 Carbamidomethyl+C(7) VTDDLVCLVYK 41 11 39.02 2 662.8467

Signal recognition particle 9 kDa protein 87.42 10111 13 4 36.05 LYLADPMK 16 8 42.68 2 475.7554

87.42 10111 13 4 36.05 LMVAK 71 5 13.88 1 561.3389

87.42 10111 13 4 36.05 FHSQLMR 64 7 42.33 2 459.7359

sp|Q8NBS9|TXND5_HUMAN 237.73 47628 27 9 17.36 ALAPTWEQLALGLEHSETVK 221 20 49.78 3 731.7222

Thioredoxin domain-containing protein 5 237.73 47628 27 9 17.36 TLAPTWEELSK 354 11 37.7 2 637.8357

237.73 47628 27 9 17.36 Carbamidomethyl+C(6) IAEVDCTAER 375 10 35.59 2 582.2725

237.73 47628 27 9 17.36 GYPTLLLFR 394 9 56.9 2 540.318

237.73 47628 27 9 17.36 EFPGLAGVK 366 9 32.23 2 459.2577

237.73 47628 27 9 17.36 VDQYK 273 5 13.88 1 652.3255

237.73 47628 27 9 17.36 YQGPR 150 5 13.88 1 620.3121

237.73 47628 27 9 17.36 DLESLR 281 6 14.23 1 732.3861

237.73 47628 27 9 17.36 PTLLLFR 396 7 1.39 2 430.2726

sp|P06748|NPM_HUMAN Nucleophosmin 288.16 32575 51 15 36.39 VDNDENEHQLSLR 32 13 99.92 3 523.5769

288.16 32575 51 15 36.39 GPSSVEDIK 239 9 55.65 2 466.238

288.16 32575 51 15 36.39 MTDQEAIQDLWQWR 277 14 36.32 2 910.4324

288.16 32575 51 15 36.39 TVSLGAGAKDELHIVEAEAMNYEGSPIK 45 28 16.25 4 733.1151

288.16 32575 51 15 36.39 MSVQPTVSLGGFEITPPVVLR 80 21 83.92 3 743.0696

288.16 32575 51 15 36.39 DELHIVEAEAMNYEGSPIK 54 19 16.13 4 537.0118

288.16 32575 51 15 36.39 FINYVK 267 6 28.1 2 392.2194

288.16 32575 51 15 36.39 LLSISGK 134 7 42.33 2 359.2256

288.16 32575 51 15 36.39 DYHFK 27 5 13.88 2 355.1703

288.16 32575 51 15 36.39 Carbamidomethyl+C(8) FINYVKNCFR 267 10 30.36 2 680.8466

288.16 32575 51 15 36.39 NDENEHQLSLR 34 11 2.79 2 677.8252

288.16 32575 51 15 36.39 DNDENEHQLSLR 33 12 7.63 2 735.3365

288.16 32575 51 15 36.39 PSSVEDIK 240 8 1.39 2 437.7317

288.16 32575 51 15 36.39 SSVEDIK 241 7 1.39 1 777.3959

288.16 32575 51 15 36.39 VDNDENEHQLSLR 32 13 2.79 3 517.9077

sp|Q9BRA2|TXD17_HUMAN 17 139.22 13940 14 3 26.83 VTAVPTLLK 89 9 40.01 2 471.3041

Thioredoxin domain-containing protein 139.22 13940 14 3 26.83 TIFAYFTGSK 25 10 57.25 2 567.7943

139.22 13940 14 3 26.83 Carbamidomethyl+C(3) SWCPDCVQAEPVVR 40 14 28.53 2 851.8937

Carbamidomethyl+C(6)

sp|P27797|CALR_HUMAN Calreticulin 334.07 48141 48 9 23.26 IKDPDASKPEDWDER 207 15 38.78 3 600.9527

334.07 48141 48 9 23.26 EQFLDGDGWTSR 24 12 64.04 2 705.8207

334.07 48141 48 9 23.26 FYALSASFEPFSNK 73 14 54.14 2 804.3943

334.07 48141 48 9 23.26 QIDNPDYK 278 8 37.9 2 496.739

334.07 48141 48 9 23.26 FVLSSGK 48 7 56.2 2 369.2115

334.07 48141 48 9 23.26 VHVIFNYK 143 8 37.9 2 510.2862

334.07 48141 48 9 23.26 FYGDEEKDK 55 9 40.01 2 565.7536

334.07 48141 48 9 23.26 IDDPTDSKPEDWDKPEHIPDPDAK 224 24 30.62 5 552.8531

334.07 48141 48 9 23.26 GDEEKDK 57 7 1.39 2 410.6908

sp|O43707|ACTN4_HUMAN Alpha-actinin-4 468.17 104854 45 9 9.55 VGWEQLLTTIAR 733 12 105 2 693.8933

468.17 104854 45 9 9.55 LASDLLEWIR 300 10 91.89 2 608.3423

468.17 104854 45 9 9.55 MLDAEDIVNTARPDEK 239 16 26.28 3 606.3028

468.17 104854 45 9 9.55 LSNRPAFMPSEGK 365 13 22.17 3 478.5766

468.17 104854 45 9 9.55 Carbamidomethyl+C(10) ELPPDQAEYCIAR 869 13 26.94 2 781.3685

468.17 104854 45 9 9.55 QQSNEHLR 643 8 56.55 2 506.2561

468.17 104854 45 9 9.55 Carbamidomethyl+C(2) ICDQWDALGSLTHSR 497 15 27.63 3 586.9444

468.17 104854 45 9 9.55 Oxidation+M(8) LSNRPAFMPSEGK 365 13 48.95 2 725.3635

468.17 104854 45 9 9.55 TARPDEK 248 7 3.84 2 408.7174

sp|P19338|NUCL_HUMAN Nucleolin 329.68 76614 43 10 14.37 EVFEDAAEIR 410 10 54.24 2 589.79

329.68 76614 43 10 14.37 NDLAVVDVR 333 9 63 2 500.7731

329.68 76614 43 10 14.37 ALELTGLK 362 8 68.67 2 422.7587

329.68 76614 43 10 14.37 LELQGPR 554 7 42.33 2 406.7335

329.68 76614 43 10 14.37 GIAYIEFK 429 8 37.9 2 470.7596

329.68 76614 43 10 14.37 SISLYYTGEK 457 10 28.6 3 387.5336

329.68 76614 43 10 14.37 IGMTR 342 5 13.88 1 577.3073

329.68 76614 43 10 14.37 VEGTEPTTAFNLFVGNLNFNK 297 21 14.95 3 771.3922

329.68 76614 43 10 14.37 TEADAEKTFEEK 437 12 23.86 3 466.5481

329.68 76614 43 10 14.37 GGGGDHKPQGKK 694 12 34.07 2 583.3022

sp|Q6S8J3|POTEE_HUMAN POTE 663.17 121363 61 9 9.86 SYELPDGQVITIGNER 938 16 170.11 2 895.9453

ankyrin domain family member E 663.17 121363 61 9 9.86 AGFAGDDAPR 718 10 135.74 2 488.7222

663.17 121363 61 9 9.86 Carbamidomethyl+C(2) LCYVALDFEQEMATAASSSSLEK 915 23 111.02 3 850.7286

663.17 121363 61 9 9.86 IWHHTFYNELR 784 11 28.95 2 758.3808

663.17 121363 61 9 9.86 EEIAMLRLELDTMK 652 14 29.33 2 846.4437

663.17 121363 61 9 9.86 RSQEPEINK 518 9 40.01 2 550.786

663.17 121363 61 9 9.86 Carbamidomethyl+C(4) EKLCYVALDFEQEMATAASSSSLEK 913 25 19.49 3 936.4493

663.17 121363 61 9 9.86 GYRFTTMAER 896 10 28.6 2 616.2953

663.17 121363 61 9 9.86 ESYVGKEAQSK 750 11 25.94 2 613.3108

sp|P62701|RS4X_HUMAN 142.2 29597 22 6 23.57 Carbamidomethyl+C(2) ECLPLIIFLR 39 10 28.6 2 637.3726

40S ribosomal protein S4 142.2 29597 22 6 23.57 LSNIFVIGK 221 9 67.77 2 495.8024

142.2 29597 22 6 23.57 YALTGDEVK 53 9 32.23 2 498.2565

142.2 29597 22 6 23.57 IFVGTK 128 6 14.23 2 332.7018

142.2 29597 22 6 23.57 TDITYPAGFMDVISIDK 77 17 17.62 2 943.4714

142.2 29597 22 6 23.57 HPGSFDVVHVK 200 11 39.02 2 611.3269

sp|P84103|SRSF3_HUMAN 152.81 19329 28 7 29.27 AFGYYGPLR 28 9 56.9 2 522.2687

Serine/arginine-rich splicing factor 3 152.81 19329 28 7 29.27 NPPGFAFVEFEDPRDAADAVR 43 21 40.63 3 774.0375

152.81 19329 28 7 29.27 VYVGNLGNNGNK 11 12 47.05 2 624.8233

152.81 19329 28 7 29.27 NPPGFAFVEFEDPR 43 14 20.75 2 811.3868

152.81 19329 28 7 29.27 SVWVAR 37 6 14.23 1 717.4119

152.81 19329 28 7 29.27 SVWVARNPPGFAFVEFEDPR 37 20 15.51 3 774.0595

152.81 19329 28 7 29.27 PRDAADAVR 55 9 7.41 2 485.7553

sp|P48643|TCPE_HUMAN 296.76 59671 48 12 22.92 LGFAGLVQEISFGTTK 352 16 43.97 2 834.4573

T-complex protein 1 subunit epsilon 296.76 59671 48 12 22.92 IADGYEQAAR 132 10 61.13 2 547.2705

296.76 59671 48 12 22.92 Carbamidomethyl+C(7) SLHDALCVIR 400 10 58.57 2 592.3159

296.76 59671 48 12 22.92 LMGLEALK 27 8 34.89 2 437.7521

296.76 59671 48 12 22.92 GVIVDKDFSHPQMPK 226 15 15.68 3 566.6338

296.76 59671 48 12 22.92 Carbamidomethyl+C(10) EMNPALGIDCLHK 483 13 29.65 3 499.9075

296.76 59671 48 12 22.92 Carbamidomethyl+C(6) IAILTCPFEPPKPK 247 14 35.14 3 537.63

296.76 59671 48 12 22.92 Carbamidomethyl+C(7) MLVIEQCK 370 8 34.89 2 510.7718

296.76 59671 48 12 22.92 FSELTAEK 344 8 34.89 2 462.7335

296.76 59671 48 12 22.92 TSLGPNGLDKMMVDK 49 15 19.55 3 535.9332

296.76 59671 48 12 22.92 AVANTMRTSLGPNGLDK 42 17 28.11 2 872.9586

296.76 59671 48 12 22.92 Oxidation+M(2) LMVELSK 89 7 42.33 2 418.2337

sp|P62917|RL8_HUMAN 128.35 28024 24 9 25.29 ASGNYATVISHNPETK 128 16 26.22 3 563.6116

60S ribosomal protein L8 128.35 28024 24 9 25.29 AVVGVVAGGGR 163 11 28.95 2 471.2801

128.35 28024 24 9 25.29 DIIHDPGR 46 8 34.89 2 461.739

128.35 28024 24 9 25.29 VVFRDPYR 60 8 37.9 2 526.2822

128.35 28024 24 9 25.29 GAPLAK 54 6 31.11 1 556.3404

128.35 28024 24 9 25.29 TVQEK 250 5 13.88 1 604.3272

128.35 28024 24 9 25.29 GAGSVFRAHVK 10 11 32.93 2 564.8108

128.35 28024 24 9 25.29 ASGNYATV 128 8 3.84 1 782.3706

128.35 28024 24 9 25.29 GNYATVISHNPETK 130 14 7.3 2 765.8797

sp|A5A3E0|POTEF_HUMAN POTE 565.68 121445 52 9 10.33 SYELPDGQVITIGNER 938 16 170.11 2 895.9453

ankyrin domain family member F 565.68 121445 52 9 10.33 AGFAGDDAPR 718 10 135.74 2 488.7222

565.68 121445 52 9 10.33 IWHHTFYNELR 784 11 28.95 2 758.3808

565.68 121445 52 9 10.33 EKDILHENSTLR 640 12 23.86 3 485.5904

565.68 121445 52 9 10.33 SNVGTSGDHDDSAMK 40 15 27.87 3 507.5401

565.68 121445 52 9 10.33 VVEVDSMPAASSVK 1 14 20.75 2 709.868

565.68 121445 52 9 10.33 YLEDIESVK 675 9 32.23 2 548.2811

565.68 121445 52 9 10.33 TALHLASANGNSEVVK 174 16 18.52 2 805.9244

565.68 121445 52 9 10.33 ILTEHGYR 891 8 34.89 2 494.7583

sp|Q01105|SET_HUMAN Protein SET 162.18 33488 24 5 20 VEVTEFEDIK 122 10 88.01 2 604.8054

162.18 33488 24 5 20 EFHLNESGDPSSK 154 13 29.65 3 482.8854

162.18 33488 24 5 20 IDFYFDENPYFENK 136 14 46.88 2 920.9144

162.18 33488 24 5 20 LNEQASEEILK 57 11 32.93 2 637.3279

162.18 33488 24 5 20 SSQTQNKASR 182 10 28.6 3 369.5226

sp|P62269|RS18_HUMAN 105.92 17718 13 4 19.08 IPDWFLNR 78 8 51.78 2 530.7812

40S ribosomal protein S18 105.92 17718 13 4 19.08 YSQVLANGLDNK 94 12 27.84 2 661.3393

105.92 17718 13 4 19.08 VLNTNIDGR 14 9 40.01 2 501.2727

105.92 17718 13 4 19.08 PDWFLNR 79 7 1.05 2 474.2398

sp|P46781|RS9_HUMAN 100.16 22591 14 5 15.98 LFEGNALLR 70 9 47.87 2 516.7954

40S ribosomal protein S9 100.16 22591 14 5 15.98 IEDFLER 101 7 42.33 2 461.2373

100.16 22591 14 5 15.98 MPVAR 0 5 13.88 1 573.3194

100.16 22591 14 5 15.98 VLIR 127 4 13.53 1 500.3531

100.16 22591 14 5 15.98 SIHHAR 121 6 14.23 2 360.7003

sp|P38159|HNRPG_HUMAN 282.99 42331 50 10 23.79 LFIGGLNTETNEK 9 13 48.37 2 718.3771

Heterogeneous nuclear ribonucleoprotein G 282.99 42331 50 10 23.79 IVEVLLMK 33 8 37.9 2 472.7951

282.99 42331 50 10 23.79 GFAFVTFESPADAK 49 14 63.84 2 743.8667

282.99 42331 50 10 23.79 GPPPSYGGSSR 298 11 28.95 2 531.2589

282.99 42331 50 10 23.79 DRDYSDHPSGGSYR 268 14 29.33 3 537.8991

282.99 42331 50 10 23.79 ALEAVFGK 22 8 42.68 2 417.7382

282.99 42331 50 10 23.79 VEQATKPSFESGR 80 13 118.65 3 479.2403

282.99 42331 50 10 23.79 DVYLSPR 203 7 53.19 2 425.2292

282.99 42331 50 10 23.79 ALEAVFGKYGR 22 11 25.94 3 404.2205

282.99 42331 50 10 23.79 SRGFAFVTFESPADAK 47 16 18.52 3 577.2849

sp|Q16629|SRSF7_HUMAN 104.15 27366 14 5 19.75 VYVGNLGTGAGK 12 12 31.06 2 568.3122

Serine/arginine-rich splicing factor 7 104.15 27366 14 5 19.75 AFSYYGPLR 29 9 40.01 2 537.2773

104.15 27366 14 5 19.75 NPPGFAFVEFEDPR 44 14 20.75 2 811.3868

104.15 27366 14 5 19.75 Carbamidomethyl+C(3) VICGSR 70 6 14.23 1 691.3575

104.15 27366 14 5 19.75 TVWIAR 38 6 14.23 2 373.2234

sp|P63241|IF5A1_HUMAN 116.56 16832 33 6 44.81 NGFVVLK 27 7 42.33 2 388.7346

Eukaryotic translation initiation factor 5A-1 116.56 16832 33 6 44.81 NDFQLIGIQDGYLSLLQDSGEVR 86 23 56.39 3 860.7684

116.56 16832 33 6 44.81 VHLVGIDIFTGK 55 12 111.77 3 433.5804

116.56 16832 33 6 44.81 IVEMSTSK 39 8 37.9 2 447.7406

116.56 16832 33 6 44.81 Carbamidomethyl+C(6) KYEDICPSTHNMDVPNIK 67 18 40.95 3 721.0098

116.56 16832 33 6 44.81 RNDFQLIGIQDGYLSLLQDSGEVR 85 24 21.02 3 912.8029

sp|P62263|RS14_HUMAN 108.42 16272 11 2 15.89 TPGPGAQSALR 106 11 37.7 2 527.7837

40S ribosomal protein S14 108.42 16272 11 2 15.89 IEDVTPIPSDSTR 128 13 48.37 2 715.3644

sp|P08758|ANXA5_HUMAN Annexin A5 150.04 35936 22 6 15 SEIDLFNIR 276 9 40.01 2 553.7975

150.04 35936 22 6 15 GLGTDEESILTLLTSR 29 16 68.23 2 852.959

150.04 35936 22 6 15 SNAQR 45 5 13.88 1 575.2859

150.04 35936 22 6 15 MAQVLR 0 6 14.23 2 359.2037

150.04 35936 22 6 15 GTVTDFPGFDER 6 12 36.49 2 670.8179

150.04 35936 22 6 15 GLGTDEESILTLLTSRSNAQR 29 21 20.66 3 754.4026

sp|Q14195|DPYL3_HUMAN 232.79 61963 54 13 27.89 Carbamidomethyl+C(8) GAPLVVICQGK 440 11 25.94 2 571.3223

Dihydropyrimidinase-related protein 3 232.79 61963 54 13 27.89 IFNLYPR 390 7 56.2 2 461.7604

232.79 61963 54 13 27.89 Carbamidomethyl+C(4) FIPCSPFSDYVYK 467 13 34.49 2 811.8891

232.79 61963 54 13 27.89 NLHQSGFSLSGTQVDEGVR 531 19 25.5 3 677.6686

232.79 61963 54 13 27.89 IVAPPGGR 555 8 56.55 2 383.7308

232.79 61963 54 13 27.89 LLIK 16 4 27.4 1 486.3639

232.79 61963 54 13 27.89 ISVGSDSDLVIWDPDAVK 400 18 16.83 2 958.4908

232.79 61963 54 13 27.89 Carbamidomethyl+C(10) AITIASQTNCPLYVTK 238 16 29.68 2 890.472

232.79 61963 54 13 27.89 DNFTAIPEGTNGVEER 345 16 80.04 2 874.9146

232.79 61963 54 13 27.89 GNVVFGEPITASLGIDGTHYWSK 270 23 23.05 3 816.7457

232.79 61963 54 13 27.89 GMYDGPVFDLTTTPK 496 15 27.63 2 821.4052

232.79 61963 54 13 27.89 GGTPAGSAR 511 9 32.23 2 387.1983

232.79 61963 54 13 27.89 NLHQSGFSLSGTQVDEGVR 531 19 4.88 3 671.6585

sp|P61353|RL27_HUMAN 110.36 15797 15 3 27.94 NIDDGTSDRPYSHALVAGIDR 27 21 14.95 3 758.0372

60S ribosomal protein L27 110.36 15797 15 3 27.94 YSVDIPLDK 84 9 32.23 2 525.2773

110.36 15797 15 3 27.94 VVLVLAGR 9 8 70.43 2 413.7792

sp|P52272|HNRPM_HUMAN 342.46 77515 71 19 29.32 INEILSNALK 371 10 69.44 2 557.8249

Heterogeneous nuclear ribonucleoprotein M 342.46 77515 71 19 29.32 AFITNIPFDVK 72 11 37.7 2 632.8515

342.46 77515 71 19 29.32 LGSTVFVANLDYK 201 13 36.64 2 713.8839

342.46 77515 71 19 29.32 MGPLGLDHMASSIER 456 15 48.27 3 538.5973

342.46 77515 71 19 29.32 ADILEDKDGK 232 10 30.36 3 368.5214

342.46 77515 71 19 29.32 Carbamidomethyl+C(4) FNECGHVLYADIK 672 13 22.17 2 783.3612

342.46 77515 71 19 29.32 FESPEVAER 698 9 40.01 2 532.2632

342.46 77515 71 19 29.32 GNFGGSFAGSFGGAGGHAPGVAR 627 23 51.69 3 678.988

342.46 77515 71 19 29.32 GEGERPAQNEK 37 11 25.94 3 405.5315

342.46 77515 71 19 29.32 MAAPIDR 496 7 42.33 2 387.204

342.46 77515 71 19 29.32 VGEVTYVELLMDAEGK 94 16 25.05 2 876.9447

342.46 77515 71 19 29.32 AAGVEAAAEVAATEIK 1 16 30.35 2 750.8906

342.46 77515 71 19 29.32 MGLAMGGGGGASFDR 606 15 31.51 2 692.302

342.46 77515 71 19 29.32 ALPK 281 4 13.53 1 428.2866

342.46 77515 71 19 29.32 MGPVMDRMATGLER 543 14 20.75 2 782.3829

342.46 77515 71 19 29.32 MGSGVERMGPAIER 510 14 20.75 3 497.251

342.46 77515 71 19 29.32 MGSVERMGSGIER 443 13 36.25 2 704.839

342.46 77515 71 19 29.32 Oxidation+M(1) MVPAGMGAGLER 531 12 23.86 2 602.7997

342.46 77515 71 19 29.32 Oxidation+M(11) VGEVTYVELLMDAEGK 94 16 18.52 3 590.2922

sp|P05388|RLA0_HUMAN 120.3 34273 25 6 27.76 IIQLLDDYPK 16 10 61.13 2 609.338

60S acidic ribosomal protein P0 120.3 34273 25 6 27.76 TSFFQALGITTK 134 12 31.06 2 657.3554

120.3 34273 25 6 27.76 AFLADPSAFVAAAPVAAATTAAPAAAAAPAK 266 31 20.61 3 918.1608

120.3 34273 25 6 27.76 EDLTEIR 92 7 42.33 2 438.228

120.3 34273 25 6 27.76 Carbamidomethyl+C(7) AGAIAPCEVTVPAQNTGLGPEK 112 22 23.21 3 727.3694

120.3 34273 25 6 27.76 FLEGVR 214 6 14.23 2 360.7043

**Mock_3**

**Protein score^b^ avg Matched Matched seq peptide seq^h^ seq seq score^m^ z^n^ mz^o^**

**Name^a^ Mass^c^ Products^d^ Peptides^e^ Cover(%)^f^  modification^g^ Start^i^ Length^l^**

sp|P60174|TPIS_HUMAN 994.78 26669 167 23 88.35 VVLAYEPVWAIGTGK 160 15 217.05 2 801.946

Triosephosphate isomerase 994.78 26669 167 23 88.35 Carbamidomethyl+C(2) DCGATWVVLGHSER 85 14 95.07 3 529.5797

994.78 26669 167 23 88.35 Carbamidomethyl+C(12) IIYGGSVTGATCK 206 13 83.76 2 663.8397

994.78 26669 167 23 88.35 SNVSDAVAQSTR 194 12 114.21 2 617.808

994.78 26669 167 23 88.35 VVFEQTK 142 7 53.58 2 425.7339

994.78 26669 167 23 88.35 HVFGESDELIGQK 100 13 138.7 3 486.9085 994.78 26669 167 23 88.35 QSLGELIGTLNAAK 19 14 93.43 2 707.9004

994.78 26669 167 23 88.35 Carbamidomethyl+C(14) VAHALAEGLGVIACIGEK 113 18 128.45 3 603.3257

994.78 26669 167 23 88.35 VTNGAFTGEISPGMIK 69 16 42.18 2 811.4199

994.78 26669 167 23 88.35 ELASQPDVDGFLVGGASLKPEFVDIINAK 219 29 79.02 3 1010.5339

994.78 26669 167 23 88.35 Carbamidomethyl+C(8) IAVAAQNCYK 59 10 30.63 2 569.2851

994.78 26669 167 23 88.35 Carbamidomethyl+C(9) VPADTEVVCAPPTAYIDFAR 33 20 184.53 3 731.358

994.78 26669 167 23 88.35 EAGITEK 135 7 39.6 1 747.38

994.78 26669 167 23 88.35 KQSLGELIGTLNAAK 18 15 46.27 3 514.9636

994.78 26669 167 23 88.35 RHVFGESDELIGQK 99 14 101.84 3 538.943

994.78 26669 167 23 88.35 Carbamidomethyl+C(9) VPADTEVVCAPPTAYIDFARQK 33 22 14.63 3 816.7482

994.78 26669 167 23 88.35 GWLKSNVSDAVAQSTR 190 16 42.18 2 859.9496

994.78 26669 167 23 88.35 KFFVGGNWK 5 9 32.5 2 541.7896

994.78 26669 167 23 88.35 TATPQQAQEVHEKLR 175 15 25.8 2 868.4579

994.78 26669 167 23 88.35 Oxidation+M(14) VTNGAFTGEISPGMIK 69 16 27.11 3 546.6055

994.78 26669 167 23 88.35 GATWVVLGHSER 87 12 7.5 2 656.346

994.78 26669 167 23 88.35 Carbamidomethyl+C(1) CGATWVVLGHSER 86 13 3.07 3 491.2461

994.78 26669 167 23 88.35 VTNGAFTGEISPGMI 69 15 3.75 2 747.3607

sp|P07737|PROF1_HUMAN Profilin-1 482.52 15054 63 9 62.14 TFVNITPAEVGVLVGK 38 16 187.4 2 822.4713

482.52 15054 63 9 62.14 STGGAPTFNVTVTK 91 14 77.11 2 690.3585

482.52 15054 63 9 62.14 DSPSVWAAVPGK 26 12 87.68 2 607.3152

482.52 15054 63 9 62.14 TLVLLMGK 108 8 42.96 2 437.7736

482.52 15054 63 9 62.14 SSFYVNGLTLGGQK 56 14 72.92 2 735.8848

482.52 15054 63 9 62.14 DSLLQDGEFSMDLR 75 14 36.55 2 813.3813

482.52 15054 63 9 62.14 Carbamidomethyl+C(1) CYEMASHLR 127 9 40.29 2 583.7618

482.52 15054 63 9 62.14 Carbamidomethyl+C(16);

Oxidation+M(11) AGWNAYIDNLMADGTCQDAAIVGYK 1 25 23.89 3 911.7401

482.52 15054 63 9 62.14 PSVWAAVPGK 28 10 22.56 2 506.2841

sp|P62937|PPIA_HUMAN 827.01 18012 109 13 62.42 FEDENFILK 82 9 94.44 2 577.7878

Peptidyl-prolyl cis-trans isomerase A 827.01 18012 109 13 62.42 TEWLDGK 118 7 42.61 2 424.7073

827.01 18012 109 13 62.42 VSFELFADK 19 9 108.41 2 528.2705

827.01 18012 109 13 62.42 Carbamidomethyl+C(7) IIPGFMCQGGDFTR 55 14 114.89 2 799.8771

827.01 18012 109 13 62.42 EGMNIVEAMER 133 11 92.11 2 639.7973

827.01 18012 109 13 62.42 Carbamidomethyl+C(24) HTGPGILSMANAGPNTNGSQFFICTAK 91 27 154.23 3 931.1135

827.01 18012 109 13 62.42 VNPTVFFDIAVDGEPLGR 1 18 154.93 2 973.5097

827.01 18012 109 13 62.42 VKEGMNIVEAMER 131 13 112.96 2 753.3797

827.01 18012 109 13 62.42 SIYGEKFEDENFILK 76 15 59.49 3 611.3056

827.01 18012 109 13 62.42 Carbamidomethyl+C(24);

Oxidation+M(9) HTGPGILSMANAGPNTNGSQFFICTAK 91 27 12.78 3 936.4445

827.01 18012 109 13 62.42 FELFADK 21 7 24.55 2 435.2229

827.01 18012 109 13 62.42 Carbamidomethyl+C(5) PGFMCQGGDFTR 57 12 7.5 2 686.7989

827.01 18012 109 13 62.42 DENFILK 84 7 57.27 1 878.4587

sp|P23528|COF1_HUMAN Cofilin-1 455.37 18502 81 8 51.2 YALYDATYETK 81 11 145.01 2 669.3147

455.37 18502 81 8 51.2 LGGSAVISLEGKPL 152 14 33.99 2 670.8951

455.37 18502 81 8 51.2 Carbamidomethyl+C(7) HELQANCYEEVKDR 132 14 129.62 3 597.6054

455.37 18502 81 8 51.2 Carbamidomethyl+C(7) MLPDKDCR 73 8 66.14 2 517.7414

455.37 18502 81 8 51.2 EILVGDVGQTVDDPYATFVK 53 20 59.74 2 1083.5582

455.37 18502 81 8 51.2 Carbamidomethyl+C(5) AVLFCLSEDK 34 10 88.58 2 591.2882

455.37 18502 81 8 51.2 NIILEEGKEILVGDVGQTVDDPYATFVK 45 28 265.33 3 1021.5383

455.37 18502 81 8 51.2 ASGVAVSDGVIK 1 12 53.52 3 394.8575

sp|P16949|STMN1_HUMAN Stathmin 382.73 17302 43 7 30.2 ASGQAFELILSPR 14 13 160.46 2 694.8783

382.73 17302 43 7 30.2 AIEENNNFSK 85 10 54.6 2 583.2777

382.73 17302 43 7 30.2 DLSLEEIQK 43 9 96.2 2 537.7882

382.73 17302 43 7 30.2 ESVPEFPLSPPK 29 12 67.68 2 663.8526

382.73 17302 43 7 30.2 ESVPEFPLSPPKK 29 13 27.18 2 727.9032

382.73 17302 43 7 30.2 SLEEIQK 45 7 9.32 1 846.4557

382.73 17302 43 7 30.2 EENNNFSK 87 8 1.7 1 981.4237

sp|P25398|RS12_HUMAN 265.5 14515 33 6 33.33 Carbamidomethyl+C(8) LGEWVGLCK 84 9 110.17 2 531.2747

40S ribosomal protein S12 265.5 14515 33 6 33.33 Carbamidomethyl+C(6) LVEALCAEHQINLIK 63 15 62.5 3 584.3185

265.5 14515 33 6 33.33 TALIHDGLAR 23 10 71.59 2 533.7983

265.5 14515 33 6 33.33 Carbamidomethyl+C(4);

Carbamidomethyl+C(6) VVGCSCVVVK 102 10 30.63 3 369.5242

265.5 14515 33 6 33.33 Carbamidomethyl+C(4) EALCAEHQINLIK 65 13 36.31 2 769.9076

265.5 14515 33 6 33.33 Carbamidomethyl+C(5) VEALCAEHQINLIK 64 14 3.41 2 819.4294

sp|P62805|H4_HUMAN Histone H4 326.41 11367 47 9 51.46 VFLENVIR 60 8 80.12 2 495.2899

326.41 11367 47 9 51.46 ISGLIYEETR 46 10 107.51 2 590.8121

326.41 11367 47 9 51.46 DAVTYTEHAK 68 10 78.58 2 567.7757

326.41 11367 47 9 51.46 TVTAMDVVYALKR 80 13 61.82 3 489.6019

326.41 11367 47 9 51.46 DNIQGITKPAIR 24 12 50.51 3 442.5843

326.41 11367 47 9 51.46 TVTAMDVVYALK 80 12 31.31 2 655.8545

326.41 11367 47 9 51.46 TYTEHAK 71 7 1.7 2 425.2071

326.41 11367 47 9 51.46 TAMDVVYALKR 82 11 7.67 2 633.8493

326.41 11367 47 9 51.46 FLENVIR 61 7 10.23 2 445.7563

sp|P22626|ROA2_HUMAN 768.5 37429 112 20 39.09 GGGGNFGPGPGSNFR 213 15 98.72 2 689.3194

Heterogeneous nuclear ribonucleoproteins A2/B1 768.5 37429 112 20 39.09 IDTIEIITDR 137 10 121.48 2 594.8254

768.5 37429 112 20 39.09 GFGFVTFDDHDPVDK 153 15 95.23 3 565.9222

768.5 37429 112 20 39.09 GGNFGFGDSR 203 10 78.58 2 507.2275

768.5 37429 112 20 39.09 Carbamidomethyl+C(4) LTDCVVMR 46 8 66.14 2 497.2456

768.5 37429 112 20 39.09 LFIGGLSFETTEESLR 22 16 189.89 2 899.9686

768.5 37429 112 20 39.09 QEMQEVQSSR 190 10 21.88 2 611.2806

768.5 37429 112 20 39.09 LFVGGIK 113 7 53.58 2 367.231

768.5 37429 112 20 39.09 DYFEEYGK 129 8 42.96 2 525.7256

768.5 37429 112 20 39.09 TLETVPLER 3 9 63.47 2 529.2979

768.5 37429 112 20 39.09 NMGGPYGGGNYGPGGSGGSGGYGGR 325 25 23.89 3 730.6457

768.5 37429 112 20 39.09 DPASK 54 5 13.98 1 517.2623

768.5 37429 112 20 39.09 Carbamidomethyl+C(4) LTDCVVMRDPASK 46 13 48.81 2 746.3681

768.5 37429 112 20 39.09 GFVTFDDHDPVDK 155 13 39.11 2 746.3441

768.5 37429 112 20 39.09 VTFDDHDPVDK 157 11 15.8 2 644.3001

768.5 37429 112 20 39.09 TFDDHDPVDK 158 10 15.8 2 594.7629

768.5 37429 112 20 39.09 TIEIITDR 139 8 23.64 2 480.7677

768.5 37429 112 20 39.09 FGFVTFDDHDPVDK 154 14 3.41 3 546.9158

768.5 37429 112 20 39.09 Carbamidomethyl+C(3) TDCVVMR 47 7 10.23 1 880.4021

768.5 37429 112 20 39.09 GFGFVTFDDHDPVDK 153 15 3.41 3 559.9218

sp|P30086|PEBP1_HUMAN 288.71 21056 30 4 30.48 LYTLVLTDPDAPSR 62 14 77.57 2 780.9146

Phosphatidylethanolamine-binding protein 1 288.71 21056 30 4 30.48 VLTPTQVK 39 8 42.96 2 443.2702

288.71 21056 30 4 30.48 NRPTSISWDGLDSGK 47 15 113.19 3 544.9405

288.71 21056 30 4 30.48 GNDISSGTVLSDYVGSGPPK 93 20 54.59 2 975.4836

sp|P62258|1433E_HUMAN 14-3-3 protein epsilon 377.04 29173 60 10 30.59 NLLSVAYK 42 8 80.12 2 454.2615

377.04 29173 60 10 30.59 Carbamidomethyl+C(3);

Carbamidomethyl+C(4) LICCDILDVLDK 94 12 130.4 2 738.8782

377.04 29173 60 10 30.59 HLIPAANTGESK 106 12 53.03 2 619.3301

377.04 29173 60 10 30.59 DSTLIMQLLR 215 10 93.53 2 595.3359

377.04 29173 60 10 30.59 VAGMDVELTVEER 29 13 31.65 2 724.3706

377.04 29173 60 10 30.59 NVIGAR 50 6 28.3 1 629.3715

377.04 29173 60 10 30.59 YLAEFATGNDR 130 11 33.19 3 419.5298

377.04 29173 60 10 30.59 YLAEFATGNDRK 130 12 47.5 3 462.2301

377.04 29173 60 10 30.59 Carbamidomethyl+C(3);

Carbamidomethyl+C(4) LICCDILDVLDKHLIPAANTGESK 94 24 46.2 4 674.5962

377.04 29173 60 10 30.59 HLIPAANTGESKVFYYK 106 17 17.83 3 646.6775

sp|P11021|GRP78_HUMAN 795.16 72333 133 27 40.67 VEIIANDQGNR 49 11 106.08 2 614.8165

78 kDa glucose-regulated protein 795.16 72333 133 27 40.67 IINEPTAAAIAYGLDKR 197 17 79.38 3 605.9987

795.16 72333 133 27 40.67 IINEPTAAAIAYGLDK 197 16 104.78 2 830.4563

795.16 72333 133 27 40.67 NELESYAYSLK 562 11 75.12 2 658.8246

795.16 72333 133 27 40.67 NQLTSNPENTVFDAK 81 15 67.91 2 839.4136

795.16 72333 133 27 40.67 VLEDSDLK 344 8 42.96 2 459.7429

795.16 72333 133 27 40.67 TFAPEEISAMVLTK 138 14 71.67 2 768.9015

795.16 72333 133 27 40.67 VTHAVVTVPAYFNDAQR 164 17 82.28 3 629.9942

795.16 72333 133 27 40.67 ELEEIVQPIISK 621 12 53.03 2 699.4007

795.16 72333 133 27 40.67 ITPSYVAFTPEGER 60 14 62.62 2 783.9015

795.16 72333 133 27 40.67 VMEHFIK 261 7 39.6 2 452.2403

795.16 72333 133 27 40.67 DAGTIAGLNVMR 185 12 24.11 2 609.3187

795.16 72333 133 27 40.67 TKPYIQVDIGGGQTK 123 15 40.25 3 535.6209

795.16 72333 133 27 40.67 VYEGERPLTK 464 10 45.85 3 397.878

795.16 72333 133 27 40.67 IEWLESHQDADIEDFK 601 16 18.74 3 658.9726

795.16 72333 133 27 40.67 ITITNDQNR 523 9 40.29 2 537.7798

795.16 72333 133 27 40.67 LSSEDK 585 6 14.32 1 678.3368

795.16 72333 133 27 40.67 ETAEAYLGK 154 9 40.29 2 491.2526

795.16 72333 133 27 40.67 VVEK 118 4 13.64 1 474.2898

795.16 72333 133 27 40.67 FLPFK 113 5 13.98 2 326.1972

795.16 72333 133 27 40.67 LIGRTWNDPSVQQDIK 97 16 18.74 3 623.9954

795.16 72333 133 27 40.67 AKFEELNMDLFR 324 12 36.84 3 504.9203

795.16 72333 133 27 40.67 ITPSYVAFTPEGERLIGDAAK 60 21 15.13 3 745.7261

795.16 72333 133 27 40.67 KSDIDEIVLVGGSTR 352 15 19.78 3 530.2947

795.16 72333 133 27 40.67 LGGKLSSEDK 581 10 28.87 2 517.2845

795.16 72333 133 27 40.67 PAYFNDAQR 172 9 4.09 2 541.2614

795.16 72333 133 27 40.67 NEPTAAAIAYGLDKR 199 15 7.28 2 795.4204

sp|P0C7M2|RA1L3_HUMAN 420.62 34223 64 9 31.25 IEVIEIMTDR 130 10 119.72 2 609.8224

Putative heterogeneous nuclear 420.62 34223 64 9 31.25 EDSQRPGAHLTVK 92 13 57.05 3 479.915

ribonucleoprotein A1-like 3 420.62 34223 64 9 31.25 GFAFVTFDDHDSVDK 146 15 114.44 3 567.2557

420.62 34223 64 9 31.25 LFIGGLSFETTDESLR 15 16 87.38 2 892.9628

420.62 34223 64 9 31.25 IFVGGIK 106 7 42.61 2 367.2299

420.62 34223 64 9 31.25 GFGFVTYATVEEVDAAMNARPHK 55 23 96.43 4 628.3023

420.62 34223 64 9 31.25 NSGPYGGGGQYFAKPR 284 16 18.74 2 828.4062

420.62 34223 64 9 31.25 AFVTFDDHDSVDK 148 13 7.39 2 748.3409

420.62 34223 64 9 31.25 VTFDDHDSVDK 150 11 7.39 2 639.2914

sp|P63104|1433Z_HUMAN 439.37 27745 63 12 42.86 NLLSVAYK 41 8 80.12 2 454.2615

14-3-3 protein zeta/delta 439.37 27745 63 12 42.86 FLIPNASQAESK 103 12 63.7 2 652.847

439.37 27745 63 12 42.86 Carbamidomethyl+C(3) DICNDVLSLLEK 91 12 99.44 2 709.8655

439.37 27745 63 12 42.86 YLAEVAAGDDKK 127 12 18.58 3 427.2195

439.37 27745 63 12 42.86 DSTLIMQLLR 212 10 93.53 2 595.3359

439.37 27745 63 12 42.86 SVTEQGAELSNEER 27 14 40.98 2 774.8664

439.37 27745 63 12 42.86 TAFDEAIAELDTLSEESYK 193 19 35.91 3 711.3387

439.37 27745 63 12 42.86 YLAEVAAGDDK 127 11 29.21 2 576.2854

439.37 27745 63 12 42.86 GIVDQSQQAYQEAFEISK 139 18 17.03 2 1021.0099

439.37 27745 63 12 42.86 VVSSIEQK 60 8 38.18 2 485.2345

439.37 27745 63 12 42.86 PNASQAESK 106 9 2.39 2 466.2329

439.37 27745 63 12 42.86 AAGDDKK 132 7 2.39 1 704.354

sp|P09936|UCHL1_HUMAN 275.91 24824 39 10 21.08 Carbamidomethyl+C(17) NEAIQAAHDAVAQEGQCR 135 18 140.67 3 656.6366

Ubiquitin carboxyl-terminal hydrolase 275.91 24824 39 10 21.08 LGVAGQWR 19 8 66.14 2 443.7468

isozyme L1 275.91 24824 39 10 21.08 Carbamidomethyl+C(7) FSAVALCK 213 8 56.93 2 448.2381

275.91 24824 39 10 21.08 QIEELK 65 6 14.32 1 759.417

275.91 24824 39 10 21.08 EFTER 202 5 13.98 2 341.1608

275.91 24824 39 10 21.08 Carbamidomethyl+C(7) FSAVALCKAA 213 10 43.64 2 519.277

275.91 24824 39 10 21.08 NEAIQAAHDAV 135 11 4.43 2 569.7776

275.91 24824 39 10 21.08 Carbamidomethyl+C(15) AIQAAHDAVAQEGQCR 137 16 7.27 2 862.9101

275.91 24824 39 10 21.08 GVAGQWR 20 7 1.02 1 773.3986

275.91 24824 39 10 21.08 Carbamidomethyl+C(7) FSAVALCK 213 8 1.02 2 439.2349

sp|P10809|CH60_HUMAN 653.73 61054 106 22 34.55 VGLQVVAVK 292 9 94.44 2 456.7946

60 kDa heat shock protein 653.73 61054 106 22 34.55 ISSIQSIVPALEIANAHR 250 18 34.78 3 640.3571

653.73 61054 106 22 34.55 VGEVIVTK 344 8 42.96 2 422.7574

653.73 61054 106 22 34.55 VTDALNATR 420 9 68.24 2 480.7569

653.73 61054 106 22 34.55 LSDGVAVLK 396 9 40.29 2 451.2699

653.73 61054 106 22 34.55 TVIIEQSWGSPK 60 12 64.49 2 672.8625

653.73 61054 106 22 34.55 TLNDELEIIEGMK 205 13 66.77 2 752.8839

653.73 61054 106 22 34.55 IGIEIIK 462 7 39.6 2 393.2575

653.73 61054 106 22 34.55 VGGTSDVEVNEK 405 12 58.3 2 617.3044

653.73 61054 106 22 34.55 Carbamidomethyl+C(13) AAVEEGIVLGGGCALLR 429 17 37.03 2 842.957

653.73 61054 106 22 34.55 IQEIIEQLDVTTSEYEKEK 370 19 23.28 3 765.7264

653.73 61054 106 22 34.55 GIIDPTK 516 7 42.61 1 743.4368

653.73 61054 106 22 34.55 GVMLAVDAVIAELKK 142 15 47.86 3 519.6364

653.73 61054 106 22 34.55 Carbamidomethyl+C(1) CEFQDAYVLLSEK 236 13 51.65 2 801.3832

653.73 61054 106 22 34.55 Carbamidomethyl+C(1) CIPALDSLTPANEDQK 446 16 25.27 3 591.2895

653.73 61054 106 22 34.55 IPAMTIAK 473 8 81.88 2 422.746

653.73 61054 106 22 34.55 SIDLK 82 5 27.96 1 575.3341

653.73 61054 106 22 34.55 VGGTSDVEVNEKK 405 13 22.41 2 681.352

653.73 61054 106 22 34.55 Oxidation+M(20) IMQSSSEVGYDAMAGDFVNMVEK 493 23 28.41 4 631.7882

653.73 61054 106 22 34.55 MLAVDAVIAELKK 144 13 3.41 2 700.9131

653.73 61054 106 22 34.55 SDGVAVLK 397 8 9.32 1 788.4446

653.73 61054 106 22 34.55 LAVDAVIAELKK 145 12 7.39 2 635.3931

sp|P67936|TPM4_HUMAN 404.04 28521 70 13 47.98 IQLVEEELDR 55 10 119.72 2 622.3269

Tropomyosin alpha-4 chain 404.04 28521 70 13 47.98 TIDDLEEK 215 8 52.16 2 481.7365

404.04 28521 70 13 47.98 LVILEGELER 132 10 61.59 2 585.8376

404.04 28521 70 13 47.98 IQALQQQADEAEDR 13 14 67.69 2 807.8932

404.04 28521 70 13 47.98 AEGDVAALNR 44 10 54.6 2 508.2645

404.04 28521 70 13 47.98 MAGLNSLEAVK 0 11 29.21 3 378.2053

404.04 28521 70 13 47.98 Carbamidomethyl+C(19) EENVGLHQTLDQTLNELNCI 228 20 27.33 3 780.711

404.04 28521 70 13 47.98 AQGLQRELDGER 27 12 24.11 2 686.3462

404.04 28521 70 13 47.98 AGLNSLEAVK 1 10 28.87 2 501.2792

404.04 28521 70 13 47.98 MEIQEMQLK 104 9 47.73 2 575.2896

404.04 28521 70 13 47.98 RIQLVEEELDR 54 11 29.21 3 467.251

404.04 28521 70 13 47.98 SLEAASEKYSEK 169 12 34.77 3 447.8919

404.04 28521 70 13 47.98 EKAEGDVAALNR 42 12 56.04 3 424.8855

sp|P27348|1433T_HUMAN 14-3-3 protein theta 388.35 27764 48 7 30.61 NLLSVAYK 41 8 80.12 2 454.2615

388.35 27764 48 7 30.61 Carbamidomethyl+C(3) SICTTVLELLDK 91 12 105.76 2 696.3758

388.35 27764 48 7 30.61 YLIANATNPESK 103 12 53.03 2 660.8446

388.35 27764 48 7 30.61 DSTLIMQLLR 212 10 93.53 2 595.3359

388.35 27764 48 7 30.61 AVTEQGAELSNEER 27 14 55.63 2 766.8664

388.35 27764 48 7 30.61 KQTIDNSQGAYQEAFDISK 138 19 24.1 3 715.0171

388.35 27764 48 7 30.61 LLSVAYK 42 7 10.23 2 397.2425

sp|Q6NXT2|H3C_HUMAN Histone H3.3C 128.93 15213 19 4 20 YRPGTVALR 40 9 79.21 2 516.8002

128.93 15213 19 4 20 STELLIR 56 7 53.58 2 416.2471

128.93 15213 19 4 20 DIQLAR 122 6 42.27 2 358.2068

128.93 15213 19 4 20 LPFQR 64 5 13.98 1 660.3828

sp|P61981|1433G_HUMAN 302.16 28302 38 5 24.7 NLLSVAYK 42 8 80.12 2 454.2615

14-3-3 protein gamma 302.16 28302 38 5 24.7 DSTLIMQLLR 217 10 93.53 2 595.3359

302.16 28302 38 5 24.7 NVTELNEPLSNEER 28 14 59.95 2 822.4028

302.16 28302 38 5 24.7 Carbamidomethyl+C(6) ELEAVCQDVLSLLDNYLIK 91 19 65.91 3 745.7199

302.16 28302 38 5 24.7 AYSEAHEISK 152 10 28.87 2 567.7776

sp|P30101|PDIA3_HUMAN 528 56782 83 24 39.6 LAPEYEAAATR 62 11 68.92 2 596.3053

Protein disulfide-isomerase A3 528 56782 83 24 39.6 FVMQEEFSR 335 9 40.29 2 586.7787

528 56782 83 24 39.6 FLQDYFDGNLKR 351 12 31.31 3 505.9208

528 56782 83 24 39.6 ELSDFISYLQR 471 11 81.32 2 685.8528

528 56782 83 24 39.6 LNFAVASR 296 8 56.93 2 439.2461

528 56782 83 24 39.6 TFSHELSDFGLESTAGEIPVVAIR 305 24 68.93 3 859.1069

528 56782 83 24 39.6 TADGIVSHLK 119 10 35.86 2 520.7894

528 56782 83 24 39.6 GFPTIYFSPANK 448 12 42.73 2 671.3481

528 56782 83 24 39.6 GIVPLAK 75 7 42.61 2 349.233

528 56782 83 24 39.6 DASIVGFFDDSFSEAHSEFLK 152 21 23.58 3 783.3695

528 56782 83 24 39.6 GFPTIYFSPANKK 448 13 22.41 3 490.5945

528 56782 83 24 39.6 SEPIPESNDGPVK 366 13 34.84 2 684.8354

528 56782 83 24 39.6 YGVSGYPTLK 94 10 85.57 2 542.7937

528 56782 83 24 39.6 EATNPPVIQEEKPK 482 14 29.56 3 527.2787

528 56782 83 24 39.6 FLQDYFDGNLK 351 11 33.19 2 680.3379

528 56782 83 24 39.6 DLIQGK 252 6 14.32 1 673.3826

528 56782 83 24 39.6 FISDK 147 5 13.98 1 609.3226

528 56782 83 24 39.6 GSNYWR 274 6 14.32 1 782.3607

528 56782 83 24 39.6 DLLIAYYDVDYEK 258 13 22.41 2 810.4069

528 56782 83 24 39.6 FLDAGHKLNFAVASR 289 15 31.84 2 823.4445

528 56782 83 24 39.6 PEYEAAATR 64 9 2.04 2 504.2457

528 56782 83 24 39.6 APEYEAAATR 63 10 2.04 2 539.7633

528 56782 83 24 39.6 PTIYFSPANKK 450 11 2.73 2 633.3443

528 56782 83 24 39.6 ELSDFISYLQR 471 11 2.04 2 676.8437

sp|P31946|1433B_HUMAN 252.69 28082 34 6 24.39 NLLSVAYK 43 8 80.12 2 454.2615

14-3-3 protein beta/alpha 252.69 28082 34 6 24.39 DSTLIMQLLR 214 10 93.53 2 595.3359

252.69 28082 34 6 24.39 YLIPNATQPESK 105 12 36.84 2 680.8614

252.69 28082 34 6 24.39 YLSEVASGDNK 129 11 26.2 2 591.7837

252.69 28082 34 6 24.39 AVTEQGHELSNEER 29 14 26.43 3 533.5921

252.69 28082 34 6 24.39 GDYFRYLSEVASGDNK 124 16 25.27 3 607.6191

sp|Q01105|SET_HUMAN Protein SET 187.07 33488 27 6 19.66 VEVTEFEDIK 122 10 85.57 2 604.8069

187.07 33488 27 6 19.66 EFHLNESGDPSSK 154 13 68.14 3 482.8862

187.07 33488 27 6 19.66 IDFYFDENPYFENK 136 14 45.63 2 920.9121

187.07 33488 27 6 19.66 QPFFQK 77 6 14.32 2 397.7134

187.07 33488 27 6 19.66 DLTK 177 4 13.64 1 476.268

187.07 33488 27 6 19.66 SSQTQNKASR 182 10 35.86 3 369.5226

sp|P05387|RLA2_HUMAN 87.81 11664 24 4 69.57 LASVPAGGAVAVSAAPGSAAPAAGSAPAAAEEK 61 33 51.52 3 925.4862

60S acidic ribosomal protein P2 87.81 11664 24 4 69.57 YVASYLLAALGGNSSPSAK 2 19 33.74 2 934.9958

87.81 11664 24 4 69.57 NIEDVIAQGIGK 49 12 50.51 2 628.8469

87.81 11664 24 4 69.57 ILDSVGIEADDDRLNK 25 16 57.4 3 591.6361

sp|Q99497|PARK7_HUMAN Protein DJ-1 165.19 19891 26 9 45.5 Carbamidomethyl+C(14) VTVAGLAGKDPVQCSR 32 16 66.82 3 553.2915

165.19 19891 26 9 45.5 Carbamidomethyl+C(5) DVVICPDASLEDAKK 48 15 31.84 3 553.9429

165.19 19891 26 9 45.5 GAEEMETVIPVDVMR 12 15 27.86 2 838.4087

165.19 19891 26 9 45.5 EILK 89 4 13.64 1 502.321

165.19 19891 26 9 45.5 ALVILAK 5 7 42.61 2 364.2561

165.19 19891 26 9 45.5 Carbamidomethyl+C(7) GLIAAICAGPTALLAHEIGFGSK 99 23 23.21 3 756.4094

165.19 19891 26 9 45.5 APLVLK 182 6 14.32 1 640.4405

165.19 19891 26 9 45.5 Carbamidomethyl+C(3) VICPDASLEDAKK 50 13 3.41 2 723.3734

165.19 19891 26 9 45.5 Carbamidomethyl+C(12) VAGLAGKDPVQCSR 34 14 7.31 2 729.3786

sp|P27797|CALR_HUMAN Calreticulin 324.25 48141 59 10 35.25 IKDPDASKPEDWDER 207 15 27.86 3 600.9507

324.25 48141 59 10 35.25 EQFLDGDGWTSR 24 12 86.25 2 705.8229

324.25 48141 59 10 35.25 FVLSSGK 48 7 67.56 2 369.2112

324.25 48141 59 10 35.25 VHVIFNYK 143 8 56.93 2 510.2859

324.25 48141 59 10 35.25 FYALSASFEPFSNK 73 14 89.63 2 804.3924

324.25 48141 59 10 35.25 IDDPTDSKPEDWDKPEHIPDPDAK 224 24 20.08 5 552.8499

324.25 48141 59 10 35.25 Carbamidomethyl+C(7) HEQNIDCGGGYVK 98 13 107.73 3 492.8951

324.25 48141 59 10 35.25 KPEDWDEEMDGEWEPPVIQNPEYK 248 24 13.77 3 987.4349

324.25 48141 59 10 35.25 GLQTSQDAR 64 9 32.5 2 488.2532

324.25 48141 59 10 35.25 IDNSQVESGSLEDDWDFLPPK 185 21 24.79 4 598.5265

sp|P06748|NPM_HUMAN Nucleophosmin 353.87 32575 54 13 32.31 VDNDENEHQLSLR 32 13 84.83 3 523.5771

353.87 32575 54 13 32.31 GPSSVEDIK 239 9 71.25 2 466.2355

353.87 32575 54 13 32.31 MTDQEAIQDLWQWR 277 14 84.68 2 910.4341

353.87 32575 54 13 32.31 MSVQPTVSLGGFEITPPVVLR 80 21 95.1 3 743.0695

353.87 32575 54 13 32.31 FINYVK 267 6 28.3 2 392.2197

353.87 32575 54 13 32.31 VTLATLK 73 7 42.61 2 373.2389

353.87 32575 54 13 32.31 LLSISGK 134 7 56.59 2 359.2254

353.87 32575 54 13 32.31 ADKDYHFK 24 8 42.96 2 512.2463

353.87 32575 54 13 32.31 NAQKSNQNGK 202 10 28.87 2 544.7738

353.87 32575 54 13 32.31 NDENEHQLSLR 34 11 2.73 2 677.8243

353.87 32575 54 13 32.31 DNDENEHQLSLR 33 12 7.67 2 735.339

353.87 32575 54 13 32.31 SSVEDIK 241 7 1.36 1 777.394

353.87 32575 54 13 32.31 VDNDENEHQLSLR 32 13 2.73 3 517.908

sp|P84103|SRSF3_HUMAN 197.75 19329 22 5 25 AFGYYGPLR 28 9 63.47 2 522.269

Serine/arginine-rich splicing factor 3 197.75 19329 22 5 25 NPPGFAFVEFEDPR 43 14 55.26 2 811.3896

197.75 19329 22 5 25 VYVGNLGNNGNK 11 12 73.03 2 624.824

197.75 19329 22 5 25 SVWVAR 37 6 14.32 2 359.2046

197.75 19329 22 5 25 GYYGPLR 30 7 1.36 1 825.4208

sp|Q8NBS9|TXND5_HUMAN 217.09 47628 32 11 18.29 ALAPTWEQLALGLEHSETVK 221 20 71.16 3 731.7255

Thioredoxin domain-containing protein 5 217.09 47628 32 11 18.29 TLAPTWEELSK 354 11 37.96 2 637.8354

217.09 47628 32 11 18.29 GYPTLLLFR 394 9 40.29 2 540.3141

217.09 47628 32 11 18.29 GYPTLLWFR 260 9 40.29 2 576.8178

217.09 47628 32 11 18.29 VDQYK 273 5 27.96 1 652.3254

217.09 47628 32 11 18.29 Carbamidomethyl+C(6) IAEVDCTAER 375 10 38.87 2 582.2741

217.09 47628 32 11 18.29 DLESLR 281 6 14.32 2 366.6993

217.09 47628 32 11 18.29 EFPGLAGVK 366 9 40.29 2 459.2587

217.09 47628 32 11 18.29 PTLLLFR 396 7 1.36 2 430.2714

217.09 47628 32 11 18.29 PTLLWFR 262 7 9.32 2 466.7717

217.09 47628 32 11 18.29 PTWEQLALGLEHSETVK 224 17 7.33 3 646.6698

sp|P62269|RS18_HUMAN 114.73 17718 22 9 38.16 IPDWFLNR 78 8 59.94 2 530.7824

40S ribosomal protein S18 114.73 17718 22 9 38.16 VLNTNIDGR 14 9 40.29 2 501.275

114.73 17718 22 9 38.16 FQHILR 8 6 14.32 2 407.2434

114.73 17718 22 9 38.16 YSQVLANGLDNK 94 12 24.11 2 661.346

114.73 17718 22 9 38.16 HFWGLR 124 6 14.32 1 815.4379

114.73 17718 22 9 38.16 EDLER 108 5 13.98 1 661.3156

114.73 17718 22 9 38.16 RAGELTEDEVER 54 12 24.11 3 468.563

114.73 17718 22 9 38.16 Oxidation+M(5) VITIMQNPR 66 9 32.5 2 544.3018

114.73 17718 22 9 38.16 PDWFLNR 79 7 1.02 2 474.2383

sp|P35268|RL22_HUMAN 99.59 14787 14 3 26.56 AGNLGGGVVTIER 52 13 56.59 2 621.846

60S ribosomal protein L22 99.59 14787 14 3 26.56 ITVTSEVPFSK 69 11 33.19 2 604.3308

99.59 14787 14 3 26.56 DWLRVVANSK 97 10 28.87 2 594.324

sp|Q9BRA2|TXD17_HUMAN 113.06 13940 14 4 26.83 VTAVPTLLK 89 9 57.27 2 471.3031

Thioredoxin domain-containing protein 17 113.06 13940 14 4 26.83 TIFAYFTGSK 25 10 38.87 2 567.7929

113.06 13940 14 4 26.83 Carbamidomethyl+C(3);

Carbamidomethyl+C(6) SWCPDCVQAEPVVR 40 14 20.99 2 851.891

113.06 13940 14 4 26.83 VTAVPTLLK 89 9 40.29 2 511.2778

sp|P38159|HNRPG_HUMAN 350.15 42331 51 11 27.37 LFIGGLNTETNEK 9 13 71.54 2 718.3773

Heterogeneous nuclear ribonucleoprotein G 350.15 42331 51 11 27.37 VEQATKPSFESGR 80 13 76.56 3 479.2401

350.15 42331 51 11 27.37 GFAFVTFESPADAK 49 14 54.68 2 743.8639

350.15 42331 51 11 27.37 IVEVLLMK 33 8 38.18 2 472.7964

350.15 42331 51 11 27.37 ALEAVFGK 22 8 42.96 2 417.738

350.15 42331 51 11 27.37 DVYLSPRDDGYSTK 203 14 26.43 3 539.2612

350.15 42331 51 11 27.37 DVYLSPR 203 7 39.6 1 849.4441

350.15 42331 51 11 27.37 QERGLPPSMER 344 11 26.2 3 433.8842

350.15 42331 51 11 27.37 GGSRSDR 377 7 39.6 1 734.3467

350.15 42331 51 11 27.37 GPPPSYGGSSRYDDYSSSR 298 19 22.84 4 509.4694

350.15 42331 51 11 27.37 QATKPSFESGR 82 11 2.73 2 604.3069

sp|P63241|IF5A1_HUMAN 126.61 16832 42 8 50.65 NGFVVLK 27 7 53.58 2 388.7349

Eukaryotic translation initiation factor 5A-1 126.61 16832 42 8 50.65 NDFQLIGIQDGYLSLLQDSGEVR 86 23 60.04 3 860.7754

126.61 16832 42 8 50.65 VHLVGIDIFTGK 55 12 132.62 3 433.5807

126.61 16832 42 8 50.65 Carbamidomethyl+C(4) GRPCK 34 5 13.98 1 617.3184

126.61 16832 42 8 50.65 IVEMSTSK 39 8 42.96 2 447.7304

126.61 16832 42 8 50.65 Carbamidomethyl+C(6) KYEDICPSTHNMDVPNIK 67 18 64.46 3 721.0046

126.61 16832 42 8 50.65 NDFQLIGIQDGYLSLLQDSGEVREDLR 86 27 12.78 4 774.1506

126.61 16832 42 8 50.65 RNDFQLIGIQDGYLSLLQDSGEVR 85 24 37.29 3 912.8048

sp|P52272|HNRPM_HUMAN 270.04 77515 57 17 26.44 AFITNIPFDVK 72 11 59.38 2 632.8511

Heterogeneous nuclear ribonucleoprotein M 270.04 77515 57 17 26.44 GNFGGSFAGSFGGAGGHAPGVAR 627 23 24.27 3 678.9899

270.04 77515 57 17 26.44 MGPLGLDHMASSIER 456 15 27.86 3 538.5955

270.04 77515 57 17 26.44 FEPYANPTKR 60 10 28.87 3 408.2081

270.04 77515 57 17 26.44 LGSTVFVANLDYK 201 13 45.63 2 713.8836

270.04 77515 57 17 26.44 VGEVTYVELLMDAEGK 94 16 18.74 3 584.9549

270.04 77515 57 17 26.44 WQSLK 83 5 13.98 1 661.3709

270.04 77515 57 17 26.44 QGGGGGGGSVPGIER 388 15 27.86 2 642.8224

270.04 77515 57 17 26.44 MAAPIDR 496 7 42.61 2 387.2026

270.04 77515 57 17 26.44 AAGVEAAAEVAATEIK 1 16 25.27 2 750.8913

270.04 77515 57 17 26.44 FEPYANPTK 60 9 32.5 2 533.7715

270.04 77515 57 17 26.44 ALPK 281 4 13.64 1 428.2839

270.04 77515 57 17 26.44 Carbamidomethyl+C(3) KACQIFVR 650 8 42.96 2 511.2836

270.04 77515 57 17 26.44 MGQTMERIGSGVER 471 14 20.99 2 775.8754

270.04 77515 57 17 26.44 QGGGGGGGSVPGIERMGPGIDR 388 22 24.5 3 670.9925

270.04 77515 57 17 26.44 Carbamidomethyl+C(6) DKFNECGHVLYADIK 670 15 19.78 3 603.6281

270.04 77515 57 17 26.44 VGSEIERMGLVMDR 429 14 33.2 3 531.2632

sp|O43707|ACTN4_HUMAN Alpha-actinin-4 414.2 104854 51 12 13.94 VGWEQLLTTIAR 733 12 80.69 2 693.8923

414.2 104854 51 12 13.94 LASDLLEWIR 300 10 81.59 2 608.3442

414.2 104854 51 12 13.94 MLDAEDIVNTARPDEK 239 16 39.7 3 606.3014

414.2 104854 51 12 13.94 TAPYK 176 5 13.98 1 579.3087

414.2 104854 51 12 13.94 Carbamidomethyl+C(2) ICDQWDALGSLTHSR 497 15 19.78 3 586.9447

414.2 104854 51 12 13.94 SFSTALYGESDL 899 12 24.11 2 645.3033

414.2 104854 51 12 13.94 HTNYTMEHIR 723 10 38.87 2 651.3122

414.2 104854 51 12 13.94 DGLAFNALIHR 193 11 33.19 3 409.5532

414.2 104854 51 12 13.94 DAKGISQEQMQEFR 757 14 20.99 3 556.2714

414.2 104854 51 12 13.94 VPQKTIQEMQQK 319 12 37.33 3 486.5922

414.2 104854 51 12 13.94 STLPDADRER 574 10 28.87 2 580.2864

414.2 104854 51 12 13.94 Oxidation+M(8) LSNRPAFMPSEGK 365 13 58.81 3 483.9124

sp|P62318|SMD3_HUMAN 109.36 13916 17 3 31.75 VAQLEQVYIR 54 10 54.6 2 609.8469

Small nuclear ribonucleoprotein Sm D3 109.36 13916 17 3 31.75 FLILPDMLK 69 9 48.24 2 545.3244

109.36 13916 17 3 31.75 Carbamidomethyl+C(12) VLHEAEGHIVTCETNTGEVYR 8 21 15.13 3 805.3903

sp|Q6S8J3|POTEE_HUMAN 659.49 121363 49 4 5.86 AGFAGDDAPR 718 10 136.71 2 488.7229

POTE ankyrin domain family member E 659.49 121363 49 4 5.86 SYELPDGQVITIGNER 938 16 171.44 2 895.9455

659.49 121363 49 4 5.86 Carbamidomethyl+C(2) LCYVALDFEQEMATAASSSSLEK 915 23 137.05 3 850.7315

659.49 121363 49 4 5.86 VVEVDSMPAASSVK 1 14 30.03 2 709.8716

sp|P19338|NUCL_HUMAN Nucleolin 280.71 76614 38 9 12.82 EVFEDAAEIR 410 10 61.59 2 589.7892

280.71 76614 38 9 12.82 NDLAVVDVR 333 9 63.47 2 500.7736

280.71 76614 38 9 12.82 ALELTGLK 362 8 59.94 2 422.7589

280.71 76614 38 9 12.82 SISLYYTGEK 457 10 28.87 2 580.7956

280.71 76614 38 9 12.82 TLVLSNLSYSATEETLQEVFEK 486 22 23.38 3 834.4291

280.71 76614 38 9 12.82 GGGGDHKPQGK 694 11 26.2 2 519.2531

280.71 76614 38 9 12.82 SAPELK 318 6 14.32 1 644.363

280.71 76614 38 9 12.82 AAKEAMEDGEIDGNK 624 15 31.84 3 526.5802

280.71 76614 38 9 12.82 LELTGLK 363 7 10.23 2 387.2445

sp|P46781|RS9_HUMAN 138.57 22591 19 5 17.53 LFEGNALLR 70 9 83.47 2 516.794

40S ribosomal protein S9 138.57 22591 19 5 17.53 IEDFLER 101 7 42.61 2 461.2367

138.57 22591 19 5 17.53 VLIR 127 4 13.64 1 500.3544

138.57 22591 19 5 17.53 SIHHAR 121 6 28.3 2 360.7005

138.57 22591 19 5 17.53 LIGEYGLR 30 8 53.92 2 460.7645

sp|P62917|RL8_HUMAN 102.92 28024 17 4 15.56 ASGNYATVISHNPETK 128 16 49.8 3 563.6095

60S ribosomal protein L8 102.92 28024 17 4 15.56 AVVGVVAGGGR 163 11 39.38 2 471.2805

102.92 28024 17 4 15.56 GAPLAK 54 6 45.28 1 556.3414

102.92 28024 17 4 15.56 GAGSVFR 10 7 39.6 2 347.1864

sp|Q16629|SRSF7_HUMAN 171.22 27366 37 10 30.67 NPPGFAFVEFEDPRDAEDAVR 44 21 21.77 3 793.3715

Serine/arginine-rich splicing factor 7 171.22 27366 37 10 30.67 AFSYYGPLR 29 9 71.25 2 537.2789

171.22 27366 37 10 30.67 NPPGFAFVEFEDPR 44 14 55.26 2 811.3896

171.22 27366 37 10 30.67 VYVGNLGTGAGK 12 12 36.84 2 568.3047

171.22 27366 37 10 30.67 SPSPK 214 5 41.93 1 515.2766

171.22 27366 37 10 30.67 Carbamidomethyl+C(3) VICGSR 70 6 14.32 1 691.3555

171.22 27366 37 10 30.67 TVWIAR 38 6 14.32 2 373.2239

171.22 27366 37 10 30.67 RPFDPNDR 97 8 52.16 3 339.4971

171.22 27366 37 10 30.67 YGGETKVYVGNLGTGAGK 6 18 17.03 3 590.9686

171.22 27366 37 10 30.67 AFSYYGPLR 29 9 1.36 2 528.2727

sp|P63244|GBLP_HUMAN 172.33 35076 33 9 40.38 Carbamidomethyl+C(4) YWLCAATGPSIK 245 12 28.09 2 683.8475

Guanine nucleotide-binding 172.33 35076 33 9 40.38 LWDLTTGTTTR 88 11 39.38 2 632.8309

protein subunit beta-2-like 1 172.33 35076 33 9 40.38 Carbamidomethyl+C(22) TNHIGHTGYLNTVTVSPDGSLCASGGK 185 27 23.63 4 686.5819

172.33 35076 33 9 40.38 GHNGWVTQIATTPQFPDMILSASR 12 24 13.77 3 876.4397

172.33 35076 33 9 40.38 Carbamidomethyl+C(13) FSPNSSNPIIVSCGWDK 155 17 35.78 3 636.6401

172.33 35076 33 9 40.38 Carbamidomethyl+C(8) LWNTLGVCK 130 9 47.73 2 545.7897

172.33 35076 33 9 40.38 FVGHTK 100 6 14.32 1 688.3824

172.33 35076 33 9 40.38 VWQVTIGTR 308 9 32.5 2 530.299

172.33 35076 33 9 40.38 LTRDETNYGIPQR 44 13 27.18 2 781.9062

sp|Q07021|C1QBP_HUMAN 108.43 31362 23 7 23.4 AFVDFLSDEIKEER 80 14 60.12 3 566.6133

Complement component 1 Q 108.43 31362 23 7 23.4 VEEQEPELTSTPNFVVEVIK 154 20 15.69 3 763.0593

subcomponent-binding protein 108.43 31362 23 7 23.4 Carbamidomethyl+C(6) ALVLDCHYPEDEVGQEDEAESDIFSIR 180 27 23.63 3 1046.145

108.43 31362 23 7 23.4 LPLLR 1 5 13.98 1 611.4244

108.43 31362 23 7 23.4 Carbamidomethyl+C(6) ALVLDCHYPEDEVG 180 14 7.5 3 539.581

108.43 31362 23 7 23.4 VDFLSDEIKEER 82 12 16.55 2 740.368

108.43 31362 23 7 23.4 DFLSDEIKEER 83 11 7.5 2 690.834

sp|A5A3E0|POTEF_HUMAN 542.23 121445 39 5 5.58 AGFAGDDAPR 718 10 136.71 2 488.7229

POTE ankyrin domain family member F 542.23 121445 39 5 5.58 SYELPDGQVITIGNER 938 16 171.44 2 895.9455

542.23 121445 39 5 5.58 VVEVDSMPAASSVK 1 14 30.03 2 709.8716

542.23 121445 39 5 5.58 LTSEEESQRFK 380 11 29.21 2 677.339

542.23 121445 39 5 5.58 GEDLDKLHR 136 9 32.5 2 541.7796

sp|P60660|MYL6_HUMAN 129.45 16930 30 7 33.77 HVLVTLGEK 110 9 32.5 2 498.2962

Myosin light polypeptide 6 129.45 16930 30 7 33.77 ALGQNPTNAEVLK 37 13 22.41 2 677.8718

129.45 16930 30 7 33.77 EAFQLFDR 13 8 56.93 2 513.2569

129.45 16930 30 7 33.77 VLDFEHFLPMLQTVAK 63 16 18.74 3 630.0028

129.45 16930 30 7 33.77 EAFQLFDRTGDGK 13 13 22.41 2 742.3667

129.45 16930 30 7 33.77 Oxidation+M(11) VFDKEGNGTVMGAEIR 94 16 37.86 2 869.9214

129.45 16930 30 7 33.77 EAFQLFDR 13 8 1.02 2 504.2468

sp|Q14195|DPYL3_HUMAN 253.49 61963 29 8 14.56 IVAPPGGR 555 8 42.96 2 383.7281

Dihydropyrimidinase-related protein 3 253.49 61963 29 8 14.56 Carbamidomethyl+C(8) GAPLVVICQGK 440 11 29.21 2 571.3221

253.49 61963 29 8 14.56 IFNLYPR 390 7 53.58 2 461.7589

253.49 61963 29 8 14.56 Carbamidomethyl+C(4) FIPCSPFSDYVYK 467 13 31.65 2 811.8886

253.49 61963 29 8 14.56 GSPTRPNPPVR 520 11 26.2 2 589.3266

253.49 61963 29 8 14.56 DNFTAIPEGTNGVEER 345 16 40.97 2 874.9146

253.49 61963 29 8 14.56 Carbamidomethyl+C(10) AITIASQTNCPLYVTK 238 16 26.44 2 890.4761

253.49 61963 29 8 14.56 Carbamidomethyl+C(4) FIPCSPFSDYVYKR 467 14 30.03 2 889.9221

sp|P43243|MATR3_HUMAN Matrin-3 183.2 94623 32 9 14.52 ITPENLPQILLQLK 132 14 33.99 2 810.4961

183.2 94623 32 9 14.52 GPSLNPVLDYDHGSR 192 15 31.84 3 542.9364

183.2 94623 32 9 14.52 Carbamidomethyl+C(6);

Carbamidomethyl+C(9) GYPHLCSICDLPVHSNK 287 17 24.82 3 666.3138

183.2 94623 32 9 14.52 IGPYQPNVPVGIDYVIPK 780 18 17.03 2 985.0478

183.2 94623 32 9 14.52 EWSQHINGASHSR 304 13 22.41 3 503.5768

183.2 94623 32 9 14.52 SFQQSSLSR 3 9 47.73 2 520.2657

183.2 94623 32 9 14.52 MKSQAFIEMETR 530 12 24.11 2 735.8618

183.2 94623 32 9 14.52 GPGPLQERSLFEK 255 13 22.41 2 729.3932

183.2 94623 32 9 14.52 RTEEGPTLSYGR 148 12 24.11 2 683.3481

sp|P08758|ANXA5_HUMAN Annexin A5 155.37 35936 11 2 7.81 SEIDLFNIR 276 9 48.24 2 553.7979

155.37 35936 11 2 7.81 GLGTDEESILTLLTSR 29 16 65.19 2 852.9611

***LMNA*-KD_1**

**Protein score^b^ avg Matched Matched seq peptide seq^h^ seq seq score^m^ z^n^ mz^o^**

**Name^a^ Mass^c^ Products^d^ Peptides^e^ Cover(%)^f^  modification^g^ Start^i^ Length^l^**

sp|P60174|TPIS_HUMAN 1303.3 26669 174 28 75.1 VVLAYEPVWAIGTGK 160 15 232.39 2 801.9468

Triosephosphate isomerase 1303.3 26669 174 28 75.1 Carbamidomethyl+C(12) IIYGGSVTGATCK 206 13 103.48 2 663.8366

1303.3 26669 174 28 75.1 Carbamidomethyl+C(2) DCGATWVVLGHSER 85 14 163.85 3 529.5795

1303.3 26669 174 28 75.1 SNVSDAVAQSTR 194 12 106.74 2 617.807

1303.3 26669 174 28 75.1 VVFEQTK 142 7 31.89 2 425.7347

1303.3 26669 174 28 75.1 ELASQPDVDGFLVGGASLKPEFVDIINAK 219 29 148.12 3 1010.5329

1303.3 26669 174 28 75.1 Carbamidomethyl+C(9) VPADTEVVCAPPTAYIDFAR 33 20 174.89 2 1096.5459

1303.3 26669 174 28 75.1 HVFGESDELIGQK 100 13 105.83 2 729.8662

1303.3 26669 174 28 75.1 Carbamidomethyl+C(14) VAHALAEGLGVIACIGEK 113 18 160.17 3 603.3249

1303.3 26669 174 28 75.1 VTNGAFTGEISPGMIK 69 16 61.65 2 811.4199

1303.3 26669 174 28 75.1 QSLGELIGTLNAAK 19 14 93.02 2 707.8987

1303.3 26669 174 28 75.1 Carbamidomethyl+C(8) IAVAAQNCYK 59 10 29.21 2 569.2846

1303.3 26669 174 28 75.1 KQSLGELIGTLNAAK 18 15 74.75 3 514.9632

1303.3 26669 174 28 75.1 RHVFGESDELIGQK 99 14 30.47 3 538.9445

1303.3 26669 174 28 75.1 GWLKSNVSDAVAQSTR 190 16 25.55 3 573.627

1303.3 26669 174 28 75.1 Carbamidomethyl+C(8) IAVAAQNCYKVTNGAFTGEISPGMIK 59 26 37.38 3 914.136

1303.3 26669 174 28 75.1 GATWVVLGHSER 87 12 7.54 2 656.3449

1303.3 26669 174 28 75.1 PPTAYIDFAR 43 10 4.96 2 575.8023

1303.3 26669 174 28 75.1 Carbamidomethyl+C(3) IACIGEK 124 7 7.27 2 395.7078

1303.3 26669 174 28 75.1 Carbamidomethyl+C(14) VAHALAEGLGVIACIGE 113 17 4.3 3 560.628

1303.3 26669 174 28 75.1 ATWVVLGHSER 88 11 2.98 2 627.8301

1303.3 26669 174 28 75.1 SDAVAQSTR 197 9 2.32 1 934.4623

1303.3 26669 174 28 75.1 TWVVLGHSER 89 10 2.98 2 592.3158

1303.3 26669 174 28 75.1 Carbamidomethyl+C(1) CGATWVVLGHSER 86 13 16.73 3 491.2428

1303.3 26669 174 28 75.1 Carbamidomethyl+C(5) GVIACIGEK 122 9 14.32 2 473.7542

1303.3 26669 174 28 75.1 PVWAIGTGK 166 9 7.42 2 464.765

1303.3 26669 174 28 75.1 LAYEPVWAIGTGK 162 13 21.99 2 702.8811

1303.3 26669 174 28 75.1 Carbamidomethyl+C(9) VPADTEVVCAPPTAYIDFAR 33 20 4.96 3 725.3561

sp|P62937|PPIA_HUMAN 958.34 18012 129 17 68.48 FEDENFILK 82 9 95.32 2 577.7865

Peptidyl-prolyl cis-trans isomerase A 958.34 18012 129 17 68.48 VSFELFADK 19 9 109.43 2 528.2693

958.34 18012 129 17 68.48 Carbamidomethyl+C(7) IIPGFMCQGGDFTR 55 14 112.85 2 799.8747

958.34 18012 129 17 68.48 VNPTVFFDIAVDGEPLGR 1 18 265.04 2 973.5082

958.34 18012 129 17 68.48 EGMNIVEAMER 133 11 107.87 2 639.7958

958.34 18012 129 17 68.48 Carbamidomethyl+C(24) HTGPGILSMANAGPNTNGSQFFICTAK 91 27 192.85 3 931.115

958.34 18012 129 17 68.48 VKEGMNIVEAMER 131 13 105.83 2 753.3793

958.34 18012 129 17 68.48 TAENFR 31 6 14.44 1 737.3658

958.34 18012 129 17 68.48 Carbamidomethyl+C(7) KITIADCGQLE 154 11 59.98 2 624.3177

958.34 18012 129 17 68.48 SIYGEKFEDENFILK 76 15 74.3 3 611.3055

958.34 18012 129 17 68.48 Carbamidomethyl+C(24);

Phosphoryl STY(16) HTGPGILSMANAGPNTNGSQFFICTAK 91 27 24.04 3 957.7739

958.34 18012 129 17 68.48 FELFADK 21 7 40.64 1 869.4401

958.34 18012 129 17 68.48 Carbamidomethyl+C(5) PGFMCQGGDFTR 57 12 7.54 2 686.7983

958.34 18012 129 17 68.48 DENFILK 84 7 24.77 1 878.46

958.34 18012 129 17 68.48 SFELFADK 20 8 24.77 1 956.4746

958.34 18012 129 17 68.48 PTVFFDIAVDGEPLGR 3 16 24.45 2 866.9528

958.34 18012 129 17 68.48 DGEPLGR 12 7 4.3 1 743.3643

sp|P07737|PROF1_HUMAN Profilin-1 716.14 15054 92 15 71.43 TFVNITPAEVGVLVGK 38 16 196.04 2 822.4667

716.14 15054 92 15 71.43 STGGAPTFNVTVTK 91 14 168.62 2 690.358

716.14 15054 92 15 71.43 DSPSVWAAVPGK 26 12 104.4 2 607.3142

716.14 15054 92 15 71.43 TLVLLMGK 108 8 82.64 2 437.7718

716.14 15054 92 15 71.43 SSFYVNGLTLGGQK 56 14 86.61 2 735.883

716.14 15054 92 15 71.43 EGVHGGLINK 116 10 29.21 2 512.2829

716.14 15054 92 15 71.43 DSLLQDGEFSMDLR 75 14 48.26 2 813.3807

716.14 15054 92 15 71.43 Carbamidomethyl+C(1) CYEMASHLR 127 9 57.76 2 583.764

716.14 15054 92 15 71.43 STGGAPTFNVTVTKTDK 91 17 36.4 3 575.2949

716.14 15054 92 15 71.43 Carbamidomethyl+C(16);

Oxidation+M(11) AGWNAYIDNLMADGTCQDAAIVGYK 1 25 19.83 3 911.7525

716.14 15054 92 15 71.43 PSVWAAVPGK 28 10 7.97 2 506.2835

716.14 15054 92 15 71.43 PTFNVTVTK 96 9 29.07 1 1006.5623

716.14 15054 92 15 71.43 LVLLMGK 109 7 0.99 2 387.248

716.14 15054 92 15 71.43 EGVHGGLINK 116 10 1.65 2 503.2781

716.14 15054 92 15 71.43 DSPSVWAAVPGK 26 12 2.32 2 598.3081

sp|P23528|COF1_HUMAN Cofilin-1 1134.74 18502 141 18 63.25 YALYDATYETK 81 11 132.29 2 669.3141

1134.74 18502 141 18 63.25 LGGSAVISLEGKPL 152 14 134.38 2 670.8932

1134.74 18502 141 18 63.25 NIILEEGKEILVGDVGQTVDDPYATFVK 45 28 374.85 3 1021.5329

1134.74 18502 141 18 63.25 Carbamidomethyl+C(7) HELQANCYEEVKDR 132 14 87.58 3 597.606

1134.74 18502 141 18 63.25 Carbamidomethyl+C(7) MLPDKDCR 73 8 99.76 2 517.7417

1134.74 18502 141 18 63.25 EILVGDVGQTVDDPYATFVK 53 20 135.21 2 1083.5611

1134.74 18502 141 18 63.25 KEDLVFIFWAPESAPLK 95 17 153.38 3 664.0254

1134.74 18502 141 18 63.25 YALYDATYETKESK 81 14 21.29 3 561.2687

1134.74 18502 141 18 63.25 Carbamidomethyl+C(5) AVLFCLSEDK 34 10 44.29 2 591.2984

1134.74 18502 141 18 63.25 Carbamidomethyl+C(7) HELQANCYEEVK 132 12 24.43 2 760.3535

1134.74 18502 141 18 63.25 ASGVAVSDGVIK 1 12 43.19 3 394.8571

1134.74 18502 141 18 63.25 NIILEEGKEILVGDVGQTVDDPYATFVK 45 28 58.38 4 786.387

1134.74 18502 141 18 63.25 PESAPLK 105 7 21.78 1 741.4085

1134.74 18502 141 18 63.25 PYATFVK 66 7 23.71 2 413.2282

1134.74 18502 141 18 63.25 WAPESAPLK 103 9 3.97 2 499.7656

1134.74 18502 141 18 63.25 FWAPESAPLK 102 10 3.97 2 573.3049

1134.74 18502 141 18 63.25 APESAPLK 104 8 3.97 2 406.7281

1134.74 18502 141 18 63.25 YDATYETK 84 8 1.98 1 990.4445

sp|P62258|1433E_HUMAN 14-3-3 protein epsilon 621.63 29173 91 14 42.35 NLLSVAYK 42 8 66.77 2 454.2613

621.63 29173 91 14 42.35 Carbamidomethyl+C(3);

Carbamidomethyl+C(4) LICCDILDVLDK 94 12 158.62 2 738.8738

621.63 29173 91 14 42.35 HLIPAANTGESK 106 12 100.42 2 619.3315

621.63 29173 91 14 42.35 DSTLIMQLLR 215 10 123.87 2 595.3354

621.63 29173 91 14 42.35 EAAENSLVAYK 142 11 26.53 2 597.8063

621.63 29173 91 14 42.35 YLAEFATGNDRK 130 12 101.09 3 462.2309

621.63 29173 91 14 42.35 YLAEFATGNDR 130 11 33.52 2 628.8028

621.63 29173 91 14 42.35 AAFDDAIAELDTLSEESYK 196 19 35.05 2 1044.4984

621.63 29173 91 14 42.35 VAGMDVELTVEER 29 13 22.72 2 724.3668

621.63 29173 91 14 42.35 VFYYK 118 5 28.22 2 360.1908

621.63 29173 91 14 42.35 NVIGAR 50 6 31.56 1 629.3752

621.63 29173 91 14 42.35 Carbamidomethyl+C(3);

Carbamidomethyl+C(4) LICCDILDVLDKHLIPAANTGESK 94 24 75.51 4 674.5944

621.63 29173 91 14 42.35 AEFATGNDRK 132 10 2.32 2 554.7743

621.63 29173 91 14 42.35 LAEFATGNDRK 131 11 2.32 2 611.3086

sp|P16949|STMN1_HUMAN Stathmin 352.79 17302 50 10 45.64 ASGQAFELILSPR 14 13 211.29 2 694.8778

352.79 17302 50 10 45.64 AIEENNNFSK 85 10 72.2 2 583.2763

352.79 17302 50 10 45.64 DLSLEEIQK 43 9 48.73 2 537.788

352.79 17302 50 10 45.64 ESVPEFPLSPPK 29 12 73.75 2 663.8536

352.79 17302 50 10 45.64 QLAEK 70 5 14.11 1 588.3418

352.79 17302 50 10 45.64 ASSDIQVK 1 8 57.43 2 424.2366

352.79 17302 50 10 45.64 SKESVPEFPLSPPK 27 14 42.82 3 514.6089

352.79 17302 50 10 45.64 EHEKEVLQK 76 9 40.64 2 570.3013

352.79 17302 50 10 45.64 SLEEIQK 45 7 9.41 1 846.4555

352.79 17302 50 10 45.64 EENNNFSK 87 8 1.65 1 981.4294

sp|P0C7M2|RA1L3_HUMAN 673.3 34223 102 20 38.13 IEVIEIMTDR 130 10 137.98 2 609.8201

Putative heterogeneous 673.3 34223 102 20 38.13 GFAFVTFDDHDSVDK 146 15 152.13 3 567.2551

nuclear ribonucleoprotein A1-like 3 673.3 34223 102 20 38.13 LFIGGLSFETTDESLR 15 16 159.95 2 892.96

673.3 34223 102 20 38.13 DYFEQYGK 122 8 43.32 2 525.2339

673.3 34223 102 20 38.13 GFGFVTYATVEEVDAAMNARPHK 55 23 146.26 4 628.3018

673.3 34223 102 20 38.13 EDSQRPGAHLTVK 92 13 96.95 3 479.9129

673.3 34223 102 20 38.13 IFVGGIK 106 7 42.99 2 367.2298

673.3 34223 102 20 38.13 Carbamidomethyl+C(9) YHTVNGHNCEVR 166 12 37.29 2 743.3415

673.3 34223 102 20 38.13 NSGPYGGGGQYFAKPR 284 16 19.02 2 828.4081

673.3 34223 102 20 38.13 KIFVGGIK 105 8 52.66 2 431.2812

673.3 34223 102 20 38.13 KLFIGGLSFETTDESLR 14 17 47.12 3 638.334

673.3 34223 102 20 38.13 AFVTFDDHDSVDK 148 13 21.99 2 748.3409

673.3 34223 102 20 38.13 VTFDDHDSVDK 150 11 21.99 2 639.2906

673.3 34223 102 20 38.13 FVTFDDHDSVDK 149 12 7.42 2 712.8249

673.3 34223 102 20 38.13 VIEIMTDR 132 8 1.65 1 976.5103

673.3 34223 102 20 38.13 EEVDAAMNARPHK 65 13 7.51 2 734.3537

673.3 34223 102 20 38.13 IEIMTDR 133 7 8.77 1 877.4427

673.3 34223 102 20 38.13 DAAMNARPHK 68 10 7.51 2 555.7801

673.3 34223 102 20 38.13 FDDHDSVDK 152 9 3.31 2 539.2312

673.3 34223 102 20 38.13 VEEVDAAMNARPHK 64 14 5.95 2 783.8973

sp|P22626|ROA2_HUMAN 1091.29 37429 157 29 47.88 IDTIEIITDR 137 10 137.98 2 594.8246

Heterogeneous nuclear ribonucleoproteins A2/B1 1091.29 37429 157 29 47.88 GGGGNFGPGPGSNFR 213 15 147.64 2 689.3189

1091.29 37429 157 29 47.88 GFGFVTFDDHDPVDK 153 15 136.26 3 565.9222

1091.29 37429 157 29 47.88 LFVGGIKEDTEEHHLR 113 16 105.73 4 470.7419

1091.29 37429 157 29 47.88 LFIGGLSFETTEESLR 22 16 206.26 2 899.9665

1091.29 37429 157 29 47.88 NYYEQWGK 38 8 43.32 2 544.2458

1091.29 37429 157 29 47.88 Carbamidomethyl+C(4) LTDCVVMR 46 8 43.32 2 497.2447

1091.29 37429 157 29 47.88 GGNFGFGDSR 203 10 62.2 2 507.2268

1091.29 37429 157 29 47.88 DYFEEYGK 129 8 35.54 2 525.7258

1091.29 37429 157 29 47.88 TLETVPLER 3 9 97.08 2 529.2981

1091.29 37429 157 29 47.88 QEMQEVQSSR 190 10 72.2 2 611.2776

1091.29 37429 157 29 47.88 LFVGGIK 113 7 57.1 2 367.2313

1091.29 37429 157 29 47.88 Carbamidomethyl+C(4) LTDCVVMRDPASK 46 13 49.39 2 746.3686

1091.29 37429 157 29 47.88 YHTINGHNAEVR 173 12 68.4 3 470.8934

1091.29 37429 157 29 47.88 NMGGPYGGGNYGPGGSGGSGGYGGR 325 25 20.93 2 1095.4697

1091.29 37429 157 29 47.88 RGFGFVTFDDHDPVDK 152 16 49.51 3 617.9601

1091.29 37429 157 29 47.88 YHTINGHNAEVRK 173 13 22.72 2 769.8973

1091.29 37429 157 29 47.88 GFVTFDDHDPVDK 155 13 28.16 2 746.3429

1091.29 37429 157 29 47.88 VTFDDHDPVDK 157 11 3.31 2 644.3011

1091.29 37429 157 29 47.88 Carbamidomethyl+C(3) TDCVVMRDPASK 47 12 7.73 2 689.8332

1091.29 37429 157 29 47.88 FVTFDDHDPVDK 156 12 3.31 2 717.8351

1091.29 37429 157 29 47.88 TFDDHDPVDK 158 10 28.16 2 594.7651

1091.29 37429 157 29 47.88 TIEIITDR 139 8 8.77 1 960.5379

1091.29 37429 157 29 47.88 TEEHHLR 122 7 3.64 2 461.233

1091.29 37429 157 29 47.88 Carbamidomethyl+C(3) TDCVVMR 47 7 10.33 1 880.4079

1091.29 37429 157 29 47.88 FGFVTFDDHDPVDK 154 14 3.31 3 546.9189

1091.29 37429 157 29 47.88 ETVPLER 5 7 1.32 1 843.4553

1091.29 37429 157 29 47.88 GFGFVTFDDHDPVDK 153 15 3.31 3 559.9228

1091.29 37429 157 29 47.88 Carbamidomethyl+C(4) LTDCVVMR 46 8 0.99 2 488.2418

sp|P63104|1433Z_HUMAN 566.66 27745 68 15 46.12 NLLSVAYK 41 8 66.77 2 454.2613

14-3-3 protein zeta/delta 566.66 27745 68 15 46.12 FLIPNASQAESK 103 12 100.42 2 652.8464

566.66 27745 68 15 46.12 DSTLIMQLLR 212 10 123.87 2 595.3354

566.66 27745 68 15 46.12 Carbamidomethyl+C(3) DICNDVLSLLEK 91 12 106.74 2 709.863

566.66 27745 68 15 46.12 YLAEVAAGDDKK 127 12 68.09 3 427.2214

566.66 27745 68 15 46.12 SVTEQGAELSNEER 27 14 51.64 2 774.8676

566.66 27745 68 15 46.12 GIVDQSQQAYQEAFEISK 139 18 27.43 2 1021.0008

566.66 27745 68 15 46.12 IETELR 85 6 28.55 2 380.7111

566.66 27745 68 15 46.12 TAFDEAIAELDTLSEESYK 193 19 23.54 2 1066.5128

566.66 27745 68 15 46.12 EKIETELR 83 8 51.1 2 509.2797

566.66 27745 68 15 46.12 PNASQAESK 106 9 22.75 1 931.4496

566.66 27745 68 15 46.12 LLSVAYK 42 7 0.99 2 397.2424

566.66 27745 68 15 46.12 Carbamidomethyl+C(1) CNDVLSLLEK 93 10 2.32 2 595.8058

566.66 27745 68 15 46.12 TLIMQLLR 214 8 1.65 2 494.3058

566.66 27745 68 15 46.12 DSTLIMQLLR 212 10 1.65 2 586.3283

sp|P27797|CALR_HUMAN Calreticulin 570.17 48141 77 13 33.33 IKDPDASKPEDWDER 207 15 64.38 3 600.9528

570.17 48141 77 13 33.33 EQFLDGDGWTSR 24 12 86.98 2 705.8203

570.17 48141 77 13 33.33 FYALSASFEPFSNK 73 14 156.06 2 804.3946

570.17 48141 77 13 33.33 Carbamidomethyl+C(7) HEQNIDCGGGYVK 98 13 121.19 2 738.8367

570.17 48141 77 13 33.33 FVLSSGK 48 7 57.1 2 369.2123

570.17 48141 77 13 33.33 VHVIFNYK 143 8 68.53 2 510.287

570.17 48141 77 13 33.33 IDNSQVESGSLEDDWDFLPPKK 185 22 24.73 3 840.4045

570.17 48141 77 13 33.33 KPEDWDEEMDGEWEPPVIQNPEYK 248 24 24.27 3 987.4398

570.17 48141 77 13 33.33 IDDPTDSKPEDWDKPEHIPDPDAK 224 24 59.53 3 920.7638

570.17 48141 77 13 33.33 IDNSQVESGSLEDDWDFLPPK 185 21 38.21 4 598.5304

570.17 48141 77 13 33.33 Carbamidomethyl+C(5) QNIDCGGGYVK 100 11 2.65 2 605.7867

570.17 48141 77 13 33.33 LEDDWDFLPPKK 195 12 5.62 2 751.883

570.17 48141 77 13 33.33 Carbamidomethyl+C(2) DCGGGYVK 103 8 2.65 1 855.3758

sp|P10809|CH60_HUMAN 1271.54 61054 190 33 52.18 ISSIQSIVPALEIANAHR 250 18 63.7 3 640.3587

60 kDa heat shock protein 1271.54 61054 190 33 52.18 VGLQVVAVK 292 9 125.3 2 456.7946

1271.54 61054 190 33 52.18 VGEVIVTK 344 8 66.77 2 422.7581

1271.54 61054 190 33 52.18 VTDALNATR 420 9 79.96 2 480.7574

1271.54 61054 190 33 52.18 LSDGVAVLK 396 9 57.76 2 451.2683

1271.54 61054 190 33 52.18 TLNDELEIIEGMK 205 13 84.6 2 752.8854

1271.54 61054 190 33 52.18 TVIIEQSWGSPK 60 12 149.74 2 672.8634

1271.54 61054 190 33 52.18 IGIEIIK 462 7 39.98 2 393.257

1271.54 61054 190 33 52.18 GYISPYFINTSK 221 12 52.07 2 695.3592

1271.54 61054 190 33 52.18 VGGTSDVEVNEK 405 12 73.75 2 617.3036

1271.54 61054 190 33 52.18 GANPVEIR 133 8 35.54 2 428.239

1271.54 61054 190 33 52.18 Carbamidomethyl+C(13) AAVEEGIVLGGGCALLR 429 17 117.29 2 842.9591

1271.54 61054 190 33 52.18 LVQDVANNTNEEAGDGTTTATVLAR 96 25 29.98 3 854.0905

1271.54 61054 190 33 52.18 GVMLAVDAVIAELKK 142 15 112.52 3 519.637

1271.54 61054 190 33 52.18 ALMLQGVDLLADAVAVTMGPK 37 21 187.5 3 705.0482

1271.54 61054 190 33 52.18 IQEIIEQLDVTTSEYEKEK 370 19 38.79 3 765.726

1271.54 61054 190 33 52.18 GIIDPTK 516 7 39.98 2 372.2165

1271.54 61054 190 33 52.18 Carbamidomethyl+C(1) CIPALDSLTPANEDQK 446 16 40.12 2 886.4366

1271.54 61054 190 33 52.18 IQEIIEQLDVTTSEYEK 370 17 25.09 2 1019.516

1271.54 61054 190 33 52.18 IPAMTIAK 473 8 66.77 2 422.7456

1271.54 61054 190 33 52.18 KPLVIIAEDVDGEALSTLVLNR 268 22 21.37 3 789.1145

1271.54 61054 190 33 52.18 KISSIQSIVPALEIANAHR 249 19 34.12 3 683.0567

1271.54 61054 190 33 52.18 DGKTLNDELEIIEGMK 202 16 30.42 2 902.9506

1271.54 61054 190 33 52.18 TALLDAAGVASLLTTAEVVVTEIPKEEK 526 28 99.07 3 956.8669

1271.54 61054 190 33 52.18 GEVIVTK 345 7 0.99 1 745.4426

1271.54 61054 190 33 52.18 MLAVDAVIAELKK 144 13 7.42 2 700.9132

1271.54 61054 190 33 52.18 DALNATR 422 7 1.32 1 760.3954

1271.54 61054 190 33 52.18 AVDAVIAELKK 146 11 21.99 2 578.8567

1271.54 61054 190 33 52.18 LAVDAVIAELKK 145 12 7.42 2 635.3948

1271.54 61054 190 33 52.18 SDGVAVLK 397 8 40.64 1 788.4494

1271.54 61054 190 33 52.18 GLQVVAVK 293 8 9.41 1 813.521

1271.54 61054 190 33 52.18 IIEQLDVTTSEYEKEK 373 16 4.63 2 962.9893

1271.54 61054 190 33 52.18 LVQDVANNTNEEAGDGTTTATVLAR 96 25 6.62 3 848.0881

sp|P11021|GRP78_HUMAN 1227.7 72333 196 29 42.81 VEIIANDQGNR 49 11 137.06 2 614.8167

78 kDa glucose-regulated protein 1227.7 72333 196 29 42.81 IINEPTAAAIAYGLDK 197 16 84.72 2 830.4538

1227.7 72333 196 29 42.81 IINEPTAAAIAYGLDKR 197 17 102.08 3 605.9975

1227.7 72333 196 29 42.81 ELEEIVQPIISK 621 12 116.29 2 699.3995

1227.7 72333 196 29 42.81 TFAPEEISAMVLTK 138 14 129.6 2 768.904

1227.7 72333 196 29 42.81 IEWLESHQDADIEDFK 601 16 42.6 3 658.9735

1227.7 72333 196 29 42.81 NQLTSNPENTVFDAK 81 15 95.4 2 839.411

1227.7 72333 196 29 42.81 ITITNDQNR 523 9 40.64 2 537.7803

1227.7 72333 196 29 42.81 NELESYAYSLK 562 11 74.88 2 658.8254

1227.7 72333 196 29 42.81 TWNDPSVQQDIK 101 12 24.43 2 715.8512

1227.7 72333 196 29 42.81 VLEDSDLK 344 8 52.66 2 459.7432

1227.7 72333 196 29 42.81 LTPEEIER 532 8 52.66 2 493.7633

1227.7 72333 196 29 42.81 ITPSYVAFTPEGER 60 14 84.42 2 783.8967

1227.7 72333 196 29 42.81 VTHAVVTVPAYFNDAQR 164 17 133.25 3 629.9921

1227.7 72333 196 29 42.81 SQIFSTASDNQPTVTIK 447 17 55.66 2 918.9787

1227.7 72333 196 29 42.81 TKPYIQVDIGGGQTK 123 15 50.64 3 535.6249

1227.7 72333 196 29 42.81 FEELNMDLFR 326 10 42.53 2 657.3166

1227.7 72333 196 29 42.81 VYEGERPLTK 464 10 46.33 3 397.8783

1227.7 72333 196 29 42.81 DAGTIAGLNVMR 185 12 100.42 2 609.3216

1227.7 72333 196 29 42.81 SDIDEIVLVGGSTR 353 14 26.73 3 487.5967

1227.7 72333 196 29 42.81 Carbamidomethyl+C(17) EDVGTVVGIDLGTTYSCVGVFK 24 22 26.54 3 772.7068

1227.7 72333 196 29 42.81 FLPFK 113 5 14.11 2 326.1982

1227.7 72333 196 29 42.81 AKFEELNMDLFR 324 12 39.21 2 756.883

1227.7 72333 196 29 42.81 KSDIDEIVLVGGSTR 352 15 69.91 3 530.2841

1227.7 72333 196 29 42.81 STMKPVQKVLEDSDLK 336 16 19.02 3 606.6635

1227.7 72333 196 29 42.81 ITITNDQNRLTPEEIER 523 17 18.1 3 681.3541

1227.7 72333 196 29 42.81 PAYFNDAQR 172 9 3.97 2 541.259

1227.7 72333 196 29 42.81 NEPTAAAIAYGLDKR 199 15 7.29 2 795.4194

1227.7 72333 196 29 42.81 LEDSDLK 345 7 0.99 1 819.4066

sp|Q99497|PARK7_HUMAN Protein DJ-1 378.64 19891 63 15 60.85 Carbamidomethyl+C(14) VTVAGLAGKDPVQCSR 32 16 60.97 3 553.2915

378.64 19891 63 15 60.85 Carbamidomethyl+C(5) DVVICPDASLEDAKK 48 15 43.65 3 553.9438

378.64 19891 63 15 60.85 DGLILTSR 148 8 52.66 2 437.7524

378.64 19891 63 15 60.85 Carbamidomethyl+C(7) GLIAAICAGPTALLAHEIGFGSK 99 23 93.44 3 756.4104

378.64 19891 63 15 60.85 ALVILAK 5 7 85.32 2 364.2557

378.64 19891 63 15 60.85 EGPYDVVVLPGGNLGAQNLSESAAVK 63 26 24.85 3 862.1133

378.64 19891 63 15 60.85 Carbamidomethyl+C(5) DVVICPDASLEDAK 48 14 33.64 2 766.3733

378.64 19891 63 15 60.85 EILK 89 4 13.78 1 502.3239

378.64 19891 63 15 60.85 GAEEMETVIPVDVMR 12 15 28.16 2 838.4162

378.64 19891 63 15 60.85 Carbamidomethyl+C(8) KGLIAAICAGPTALLAHEIGFGSK 98 24 28.32 3 799.1084

378.64 19891 63 15 60.85 Carbamidomethyl+C(5) DVVICPDASLEDAKK 48 15 20.07 2 830.4091

378.64 19891 63 15 60.85 Oxidation+M(14) GAEEMETVIPVDVMRR 12 16 26.73 3 616.6338

378.64 19891 63 15 60.85 Carbamidomethyl+C(3) VICPDASLEDAKK 50 13 7.42 2 723.3749

378.64 19891 63 15 60.85 Carbamidomethyl+C(2) ICPDASLEDAKK 51 12 3.31 2 673.8384

378.64 19891 63 15 60.85 Carbamidomethyl+C(12) VAGLAGKDPVQCSR 34 14 3.64 2 729.3835

sp|P30086|PEBP1_HUMAN 312.38 21056 51 10 62.03 LYTLVLTDPDAPSR 62 14 95.36 2 780.9163

Phosphatidylethanolamine-binding protein 1 312.38 21056 51 10 62.03 WSGPLSLQEVDEQPQHPLHVTYAGAAVDELGK 7 32 17.64 4 868.6836

312.38 21056 51 10 62.03 VLTPTQVK 39 8 66.77 2 443.272

312.38 21056 51 10 62.03 NRPTSISWDGLDSGK 47 15 92.39 3 544.9382

312.38 21056 51 10 62.03 GNDISSGTVLSDYVGSGPPK 93 20 35.55 2 975.4845

312.38 21056 51 10 62.03 YVWLVYEQDRPLK 119 13 30.2 3 570.3045

312.38 21056 51 10 62.03 LYEQLSGK 179 8 43.32 2 469.256

312.38 21056 51 10 62.03 GNDISSGTVLSDYVGSGPPKGTGLHR 93 26 13.27 3 857.7604

312.38 21056 51 10 62.03 WLVYEQDRPLK 121 11 7.73 2 723.887

312.38 21056 51 10 62.03 WDGLDSGK 54 8 3.31 1 877.4099

sp|P62805|H4_HUMAN Histone H4 313.89 11367 32 8 42.72 VFLENVIR 60 8 80.88 2 495.2911

313.89 11367 32 8 42.72 ISGLIYEETR 46 10 131.65 2 590.8103

313.89 11367 32 8 42.72 DNIQGITKPAIR 24 12 47.96 3 442.5852

313.89 11367 32 8 42.72 DAVTYTEHAK 68 10 55.08 2 567.7761

313.89 11367 32 8 42.72 GGVK 41 4 13.78 1 360.2228

313.89 11367 32 8 42.72 QGITKPAIR 27 9 2.32 2 492.299

313.89 11367 32 8 42.72 IQGITKPAIR 26 10 2.32 2 548.8476

313.89 11367 32 8 42.72 GITKPAIR 28 8 2.32 2 428.2735

sp|P05387|RLA2_HUMAN 170.29 11664 35 5 69.57 YVASYLLAALGGNSSPSAK 2 19 87.13 2 934.9948

60S acidic ribosomal protein P2 170.29 11664 35 5 69.57 LASVPAGGAVAVSAAPGSAAPAAGSAPAAAEEK 61 33 66.24 3 925.4857

170.29 11664 35 5 69.57 ILDSVGIEADDDR 25 13 12.8 2 709.3484

170.29 11664 35 5 69.57 NIEDVIAQGIGK 49 12 31.63 2 628.8507

170.29 11664 35 5 69.57 ILDSVGIEADDDRLNK 25 16 73.53 3 591.6382

sp|P25398|RS12_HUMAN 230.47 14515 38 9 43.18 Carbamidomethyl+C(6) LVEALCAEHQINLIK 63 15 87.61 3 584.3193

40S ribosomal protein S12 230.47 14515 38 9 43.18 TALIHDGLAR 23 10 55.08 2 533.8038

230.47 14515 38 9 43.18 Carbamidomethyl+C(8) LGEWVGLCK 84 9 81.21 2 531.2754

230.47 14515 38 9 43.18 Carbamidomethyl+C(4);

Carbamidomethyl+C(6) VVGCSCVVVK 102 10 30.97 2 553.792

230.47 14515 38 9 43.18 ESQAKDVIEEYFK 116 13 27.49 2 793.3959

230.47 14515 38 9 43.18 Oxidation+M(11) AEEGIAAGGVMDVNTALQEVLK 1 22 38 3 744.3719

230.47 14515 38 9 43.18 Carbamidomethyl+C(4) EALCAEHQINLIK 65 13 15.96 2 769.9083

230.47 14515 38 9 43.18 Carbamidomethyl+C(7) GEWVGLCK 85 8 1.32 1 948.4541

230.47 14515 38 9 43.18 Carbamidomethyl+C(3) ALCAEHQINLIK 66 12 3.31 2 705.3822

sp|P09936|UCHL1_HUMAN 414.38 24824 73 17 56.05 MPFPVNHGASSEDTLLK 178 17 28.83 3 614.9705

Ubiquitin carboxyl-terminal 414.38 24824 73 17 56.05 Carbamidomethyl+C(17) NEAIQAAHDAVAQEGQCR 135 18 168.49 3 656.6363

hydrolase isozyme L1 414.38 24824 73 17 56.05 Carbamidomethyl+C(7) FSAVALCK 213 8 80.88 2 448.2371

414.38 24824 73 17 56.05 LGVAGQWR 19 8 66.77 2 443.7461

414.38 24824 73 17 56.05 MQLKPMEINPEMLNK 0 15 60.19 3 605.976

414.38 24824 73 17 56.05 Carbamidomethyl+C(7) QTIGNSCGTIGLIHAVANNQDK 83 22 26.54 3 771.0537

414.38 24824 73 17 56.05 VYFMK 78 5 14.11 2 344.18

414.38 24824 73 17 56.05 EFTER 202 5 14.11 2 341.1609

414.38 24824 73 17 56.05 QFLSETEKMSPEDR 115 14 21.29 3 566.2679

414.38 24824 73 17 56.05 QIEELKGQEVSPK 65 13 22.72 3 495.6079

414.38 24824 73 17 56.05 Oxidation+M(1) MPFPVNHGASSEDTLLK 178 17 18.1 3 620.3081

414.38 24824 73 17 56.05 Oxidation+M(12) MQLKPMEINPEMLNK 0 15 20.07 3 611.315

414.38 24824 73 17 56.05 Carbamidomethyl+C(6) SAVALCK 214 7 26.2 1 748.4028

414.38 24824 73 17 56.05 GVAGQWR 20 7 10.33 1 773.4024

414.38 24824 73 17 56.05 Carbamidomethyl+C(13) QAAHDAVAQEGQCR 139 14 7.27 2 770.8584

414.38 24824 73 17 56.05 MQLKPMEINPE 0 11 3.31 2 665.3381

414.38 24824 73 17 56.05 Carbamidomethyl+C(7) FSAVALCK 213 8 0.99 2 439.2346

sp|P61981|1433G_HUMAN 450.61 28302 73 10 46.96 NLLSVAYK 42 8 66.77 2 454.2613

14-3-3 protein gamma 450.61 28302 73 10 46.96 DSTLIMQLLR 217 10 123.87 2 595.3354

450.61 28302 73 10 46.96 NVTELNEPLSNEER 28 14 76.84 2 822.402

450.61 28302 73 10 46.96 AYSEAHEISK 152 10 39.21 2 567.7773

450.61 28302 73 10 46.96 YLAEVATGEK 132 10 55.08 2 540.7828

450.61 28302 73 10 46.96 Carbamidomethyl+C(6) ELEAVCQDVLSLLDNYLIK 91 19 100.62 3 745.72

450.61 28302 73 10 46.96 EHMQPTHPIR 162 10 29.21 3 415.8775

450.61 28302 73 10 46.96 Carbamidomethyl+C(22) LGLALNYSVFYYEIQNAPEQACHLAK 172 26 95.16 3 1004.838

450.61 28302 73 10 46.96 ATVVESSEK 143 9 32.86 2 475.2416

450.61 28302 73 10 46.96 SEAHEISK 154 8 8.77 2 450.725

sp|P67936|TPM4_HUMAN 334.78 28521 70 14 36.29 IQLVEEELDR 55 10 120.86 2 622.3265

Tropomyosin alpha-4 chain 334.78 28521 70 14 36.29 LVILEGELER 132 10 77.28 2 585.8387

334.78 28521 70 14 36.29 IQALQQQADEAEDR 13 14 56.2 2 807.8946

334.78 28521 70 14 36.29 MEIQEMQLK 104 9 48.73 2 575.2886

334.78 28521 70 14 36.29 AEGDVAALNR 44 10 29.21 2 508.2628

334.78 28521 70 14 36.29 MAGLNSLEAVK 0 11 41.43 2 566.8146

334.78 28521 70 14 36.29 LLSDK 190 5 14.11 1 575.3369

334.78 28521 70 14 36.29 RIQLVEEELDR 54 11 29.54 2 700.3789

334.78 28521 70 14 36.29 KLVILEGELER 131 11 74.6 2 649.8876

334.78 28521 70 14 36.29 EKAEGDVAALNR 42 12 81.54 2 636.8332

334.78 28521 70 14 36.29 KIQALQQQADEAEDR 12 15 28.16 3 581.6281

334.78 28521 70 14 36.29 Carbamidomethyl+C(1) CGDLEEELKNVTNNLK 153 16 25.55 3 625.9748

334.78 28521 70 14 36.29 AGLNSLEAVKR 1 11 26.53 2 619.311

334.78 28521 70 14 36.29 GDVAALNR 46 8 1.65 1 815.4339

sp|P63241|IF5A1_HUMAN 327.99 16832 53 7 52.6 VHLVGIDIFTGK 55 12 130.86 2 649.876

Eukaryotic translation initiation factor 5A-1 327.99 16832 53 7 52.6 IVEMSTSK 39 8 57.43 2 447.7337

327.99 16832 53 7 52.6 NGFVVLK 27 7 54.09 2 388.7349

327.99 16832 53 7 52.6 NDFQLIGIQDGYLSLLQDSGEVR 86 23 86.37 3 860.7686

327.99 16832 53 7 52.6 EDLRLPEGDLGK 109 12 35.23 3 447.9006

327.99 16832 53 7 52.6 Carbamidomethyl+C(6) KYEDICPSTHNMDVPNIK 67 18 56.37 3 721.0087

327.99 16832 53 7 52.6 RNDFQLIGIQDGYLSLLQDSGEVR 85 24 26.66 3 912.8074

sp|P06748|NPM_HUMAN Nucleophosmin 389.76 32575 68 15 38.78 VDNDENEHQLSLR 32 13 115.53 3 523.5783

389.76 32575 68 15 38.78 GPSSVEDIK 239 9 56.51 2 466.2388

389.76 32575 68 15 38.78 MTDQEAIQDLWQWR 277 14 93.97 2 910.4282

389.76 32575 68 15 38.78 MSVQPTVSLGGFEITPPVVLR 80 21 104.25 2 1114.1216

389.76 32575 68 15 38.78 TVSLGAGAKDELHIVEAEAMNYEGSPIK 45 28 16.78 4 733.1177

389.76 32575 68 15 38.78 DELHIVEAEAMNYEGSPIK 54 19 24.36 3 715.6761

389.76 32575 68 15 38.78 FINYVK 267 6 42.66 2 392.2203

389.76 32575 68 15 38.78 VTLATLK 73 7 42.99 2 373.2393

389.76 32575 68 15 38.78 LLSISGK 134 7 42.99 2 359.2262

389.76 32575 68 15 38.78 SNQNGK 206 6 14.44 1 647.3128

389.76 32575 68 15 38.78 TPKGPSSVEDIK 236 12 24.43 2 629.3422

389.76 32575 68 15 38.78 NDENEHQLSLR 34 11 2.65 2 677.8279

389.76 32575 68 15 38.78 DNDENEHQLSLR 33 12 7.73 2 735.3419

389.76 32575 68 15 38.78 SSVEDIK 241 7 1.32 1 777.4015

389.76 32575 68 15 38.78 VDNDENEHQLSLR 32 13 2.65 3 517.9095

sp|P27348|1433T_HUMAN 14-3-3 protein theta 398.69 27764 50 8 29.8 NLLSVAYK 41 8 66.77 2 454.2613

398.69 27764 50 8 29.8 DSTLIMQLLR 212 10 123.87 2 595.3354

398.69 27764 50 8 29.8 Carbamidomethyl+C(3) SICTTVLELLDK 91 12 100.42 2 696.3769

398.69 27764 50 8 29.8 YLIANATNPESK 103 12 28.41 2 660.8444

398.69 27764 50 8 29.8 AVTEQGAELSNEER 27 14 66.54 2 766.8672

398.69 27764 50 8 29.8 Carbamidomethyl+C(7) YLAEVACGDDRK 127 12 24.43 3 466.2236

398.69 27764 50 8 29.8 LQLIK 75 5 14.11 2 307.7127

398.69 27764 50 8 29.8 Carbamidomethyl+C(3) SICTTVLELLDKYLIANATNPESK 91 24 24.27 3 898.4722

sp|Q07021|C1QBP_HUMAN 227.9 31362 35 8 21.63 AFVDFLSDEIKEER 80 14 102.94 3 566.6124

Complement component 1 Q 227.9 31362 35 8 21.63 Carbamidomethyl+C(6) ALVLDCHYPEDEVGQEDEAESDIFSIR 180 27 57.97 3 1046.149

subcomponent-binding protein 227.9 31362 35 8 21.63 VEEQEPELTSTPNFVVEVIK 154 20 41.84 2 1144.0975

227.9 31362 35 8 21.63 AFVDFLSDEIKEER 80 14 21.29 3 566.6156

227.9 31362 35 8 21.63 VDFLSDEIKEER 82 12 29.07 2 740.3749

227.9 31362 35 8 21.63 Carbamidomethyl+C(6) ALVLDCHYPEDEVG 180 14 7.28 3 539.5837

227.9 31362 35 8 21.63 DFLSDEIKEER 83 11 7.54 2 690.8399

227.9 31362 35 8 21.63 Carbamidomethyl+C(6) ALVLDCHYPEDEVGQEDEAESDIFSIR 180 27 7.28 3 1040.4576

sp|P31946|1433B_HUMAN 279.76 28082 39 6 32.93 NLLSVAYK 43 8 66.77 2 454.2613

14-3-3 protein beta/alpha 279.76 28082 39 6 32.93 DSTLIMQLLR 214 10 123.87 2 595.3354

279.76 28082 39 6 32.93 YLIPNATQPESK 105 12 24.43 2 680.8628

279.76 28082 39 6 32.93 Carbamidomethyl+C(9) IEAELQDICNDVLELLDK 87 18 31.75 3 710.6915

279.76 28082 39 6 32.93 QTTVSNSQQAYQEAFEISK 140 19 35.05 3 720.3471

279.76 28082 39 6 32.93 AVTEQGHELSNEER 29 14 34.43 3 533.5837

sp|P60660|MYL6_HUMAN 231.41 16930 50 10 45.7 ALGQNPTNAEVLK 37 13 59.31 2 677.8705

Myosin light polypeptide 6 231.41 16930 50 10 45.7 HVLVTLGEK 110 9 48.73 2 498.2969

231.41 16930 50 10 45.7 VLDFEHFLPMLQTVAK 63 16 80.44 3 630.0021

231.41 16930 50 10 45.7 EAFQLFDR 13 8 43.32 2 513.2573

231.41 16930 50 10 45.7 DQGTYEDYVEGLR 81 13 44.31 2 772.8573

231.41 16930 50 10 45.7 HILSG 146 5 14.11 1 526.2938

231.41 16930 50 10 45.7 EAFQLFDRTGDGK 13 13 46.96 3 495.2445

231.41 16930 50 10 45.7 Oxidation+M(11) VFDKEGNGTVMGAEIR 94 16 27.01 3 580.2857

231.41 16930 50 10 45.7 PMLQTVAK 71 8 7.33 2 444.2526

231.41 16930 50 10 45.7 DFEHFLPMLQTVAK 65 14 15.33 2 838.4322

sp|P35268|RL22_HUMAN 163.19 14787 21 4 48.44 AGNLGGGVVTIER 52 13 30.2 2 621.8467

60S ribosomal protein L22 163.19 14787 21 4 48.44 ITVTSEVPFSK 69 11 86.64 2 604.3339

163.19 14787 21 4 48.44 Carbamidomethyl+C(5) FTLDCTHPVEDGIMDAANFEQFLQER 20 26 28.53 3 1028.4747

163.19 14787 21 4 48.44 VVANSKESYELR 101 12 24.43 3 465.5774

sp|P30101|PDIA3_HUMAN 716.48 56782 108 24 42.77 YGVSGYPTLK 94 10 62.2 2 542.7918

Protein disulfide-isomerase A3 716.48 56782 108 24 42.77 ELSDFISYLQR 471 11 87.74 2 685.852

716.48 56782 108 24 42.77 LAPEYEAAATR 62 11 53.65 2 596.3069

716.48 56782 108 24 42.77 FVMQEEFSR 335 9 40.64 2 586.7777

716.48 56782 108 24 42.77 TFSHELSDFGLESTAGEIPVVAIR 305 24 99.76 3 859.1063

716.48 56782 108 24 42.77 LNFAVASR 296 8 71.54 2 439.2463

716.48 56782 108 24 42.77 EATNPPVIQEEKPK 482 14 52.22 3 527.2811

716.48 56782 108 24 42.77 FLQDYFDGNLK 351 11 26.53 2 680.3351

716.48 56782 108 24 42.77 FISDKDASIVGFFDDSFSEAHSEFLK 147 26 39.15 4 735.3434

716.48 56782 108 24 42.77 LSKDPNIVIAK 422 11 53.65 2 599.3653

716.48 56782 108 24 42.77 GFPTIYFSPANKK 448 13 32.64 3 490.5964

716.48 56782 108 24 42.77 GIVPLAK 75 7 42.99 2 349.2328

716.48 56782 108 24 42.77 QAGPASVPLR 130 10 36.2 2 498.2873

716.48 56782 108 24 42.77 DASIVGFFDDSFSEAHSEFLK 152 21 38.23 3 783.3635

716.48 56782 108 24 42.77 FAHTNVESLVNEYDDNGEGIILFRPSHLTNK 183 31 42.46 4 883.1824

716.48 56782 108 24 42.77 DLIQGK 252 6 14.44 1 673.3842

716.48 56782 108 24 42.77 FISDK 147 5 14.11 1 609.324

716.48 56782 108 24 42.77 DLLIAYYDVDYEK 258 13 22.72 3 540.6061

716.48 56782 108 24 42.77 KTFSHELSDFGLESTAGEIPVVAIR 304 25 13.6 3 901.7996

716.48 56782 108 24 42.77 PPVIQEEKPK 486 10 16.73 2 582.8356

716.48 56782 108 24 42.77 PTIYFSPANKK 450 11 17.64 2 633.3511

716.48 56782 108 24 42.77 GFFDDSFSEAHSEFLK 157 16 6.95 2 931.9236

716.48 56782 108 24 42.77 TNPPVIQEEKPK 484 12 2.98 3 460.5864

716.48 56782 108 24 42.77 ELSDFISYLQR 471 11 1.98 2 676.8457

sp|P49458|SRP09_HUMAN 123.84 10111 20 6 45.35 Carbamidomethyl+C(7) VTDDLVCLVYK 41 11 74.88 2 662.8453

Signal recognition particle 9 kDa protein 123.84 10111 20 6 45.35 LYLADPMK 16 8 43.32 2 475.7539

123.84 10111 20 6 45.35 FHSQLMR 64 7 42.99 2 459.7368

123.84 10111 20 6 45.35 LMVAK 71 5 14.11 1 561.337

123.84 10111 20 6 45.35 Carbamidomethyl+C(7) VTDDLVCLVYKTDQAQDVK 41 19 16.58 3 737.3684

123.84 10111 20 6 45.35 YLADPMK 17 7 0.99 1 837.4188

sp|P62318|SMD3_HUMAN 143.08 13916 24 5 35.71 VAQLEQVYIR 54 10 65.21 2 609.8459

Small nuclear ribonucleoprotein Sm D3 143.08 13916 24 5 35.71 FLILPDMLK 69 9 65.85 2 545.3222

143.08 13916 24 5 35.71 Carbamidomethyl+C(12) VLHEAEGHIVTCETNTGEVYR 8 21 21.31 3 805.3902

143.08 13916 24 5 35.71 AAILK 99 5 28.22 1 515.3564

143.08 13916 24 5 35.71 ILPDMLK 71 7 24.77 1 829.481

sp|P38159|HNRPG_HUMAN 399.3 42331 58 13 31.2 LFIGGLNTETNEK 9 13 44.31 2 718.3764

Heterogeneous nuclear ribonucleoprotein G 399.3 42331 58 13 31.2 VEQATKPSFESGR 80 13 54.17 3 479.241

399.3 42331 58 13 31.2 GFAFVTFESPADAK 49 14 121.52 2 743.8651

399.3 42331 58 13 31.2 ALEAVFGK 22 8 43.32 2 417.7384

399.3 42331 58 13 31.2 DVYLSPR 203 7 39.98 2 425.2276

399.3 42331 58 13 31.2 IVEVLLMK 33 8 38.55 2 472.7959

399.3 42331 58 13 31.2 GPPPSYGGSSR 298 11 41.43 2 531.2588

399.3 42331 58 13 31.2 DYPSSR 223 6 14.44 1 724.3293

399.3 42331 58 13 31.2 DSYSSSRSDLYSSGR 324 15 20.07 3 556.2501

399.3 42331 58 13 31.2 SDRGGGR 381 7 39.98 1 704.339

399.3 42331 58 13 31.2 GLPPSMERGYPPPR 347 14 33.64 2 777.3938

399.3 42331 58 13 31.2 SAPPTRGPPPSYGGSSR 292 17 48.29 3 557.6111

399.3 42331 58 13 31.2 QATKPSFESGR 82 11 2.65 2 604.3069

sp|P52272|HNRPM_HUMAN 481.65 77515 85 24 30.27 INEILSNALK 371 10 79.32 2 557.8244

Heterogeneous nuclear ribonucleoprotein M 481.65 77515 85 24 30.27 AFITNIPFDVK 72 11 71.87 2 632.8517

481.65 77515 85 24 30.27 LGSTVFVANLDYK 201 13 37.19 2 713.8853

481.65 77515 85 24 30.27 GNFGGSFAGSFGGAGGHAPGVAR 627 23 53.01 3 678.9882

481.65 77515 85 24 30.27 FEPYANPTKR 60 10 46.33 3 408.2092

481.65 77515 85 24 30.27 QGGGGGGGSVPGIER 388 15 28.16 2 642.8234

481.65 77515 85 24 30.27 MGPGIDR 403 7 42.99 2 373.1872

481.65 77515 85 24 30.27 GEGERPAQNEK 37 11 26.53 2 607.7959

481.65 77515 85 24 30.27 FESPEVAER 698 9 64.09 2 532.2641

481.65 77515 85 24 30.27 MGLVMDR 436 7 42.99 2 411.2103

481.65 77515 85 24 30.27 VGEVTYVELLMDAEGK 94 16 19.02 2 876.9466

481.65 77515 85 24 30.27 Carbamidomethyl+C(2) GCGVVK 692 6 14.44 1 619.3218

481.65 77515 85 24 30.27 EIDVR 720 5 14.11 1 631.3344

481.65 77515 85 24 30.27 ALPK 281 4 13.78 1 428.2903

481.65 77515 85 24 30.27 MGPVMDRMATGLER 543 14 29.87 2 782.3826

481.65 77515 85 24 30.27 MVPAGMGAGLERMGPVMDR 531 19 25.75 3 658.9833

481.65 77515 85 24 30.27 GGNRFEPYANPTK 56 13 37.19 2 725.852

481.65 77515 85 24 30.27 MGLERMGANSLER 578 13 22.72 2 732.3641

481.65 77515 85 24 30.27 IGSGVERMGAGMGFGLER 478 18 17.29 2 912.4541

481.65 77515 85 24 30.27 MGSVERMGSGIER 443 13 31.97 2 704.8407

481.65 77515 85 24 30.27 Oxidation+M(1) MAAGVEAAAEVAATEIK 0 17 25.51 2 824.4134

481.65 77515 85 24 30.27 Oxidation+M(1) MATGLER 550 7 39.98 2 397.1955

481.65 77515 85 24 30.27 PYANPTKR 62 8 8.77 2 473.7581

481.65 77515 85 24 30.27 ITNIPFDVK 74 9 8.31 2 523.7994

sp|Q9BRA2|TXD17_HUMAN 140.64 13940 17 4 30.89 VTAVPTLLK 89 9 57.76 2 471.305

Thioredoxin domain-containing protein 17 140.64 13940 17 4 30.89 Carbamidomethyl+C(3);

Carbamidomethyl+C(6) SWCPDCVQAEPVVR 40 14 65.08 2 851.8926

140.64 13940 17 4 30.89 TIFAYFTGSKDAGGK 25 15 26.1 3 521.5945

140.64 13940 17 4 30.89 AVPTLLK 91 7 1.32 1 741.4859

sp|Q8NBS9|TXND5_HUMAN 205.85 47628 25 5 11.57 ALAPTWEQLALGLEHSETVK 221 20 74.28 3 731.7211

Thioredoxin domain-containing protein 5 205.85 47628 25 5 11.57 TLAPTWEELSK 354 11 38.42 2 637.8379

205.85 47628 25 5 11.57 Carbamidomethyl+C(6) IAEVDCTAER 375 10 55.08 2 582.2743

205.85 47628 25 5 11.57 EYVESQLQR 287 9 40.64 2 576.2867

205.85 47628 25 5 11.57 PTWEQLALGLEHSETVK 224 17 7.31 3 646.6663

sp|Q01105|SET_HUMAN Protein SET 268.73 33488 35 7 20 VEVTEFEDIK 122 10 142.75 2 604.8066

268.73 33488 35 7 20 EFHLNESGDPSSK 154 13 77.4 3 482.8868

268.73 33488 35 7 20 IDFYFDENPYFENK 136 14 42.82 2 920.915

268.73 33488 35 7 20 DLTK 177 4 13.78 1 476.2662

268.73 33488 35 7 20 QPFFQK 77 6 14.44 2 397.7123

268.73 33488 35 7 20 LNEQASEEILK 57 11 26.53 2 637.3268

268.73 33488 35 7 20 VTEFEDIK 124 8 22.09 1 980.4924

sp|P19338|NUCL_HUMAN Nucleolin 439.83 76614 72 16 22.39 VFGNEIK 370 7 39.98 2 403.725

439.83 76614 72 16 22.39 TGISDVFAK 324 9 40.64 2 469.2528

439.83 76614 72 16 22.39 EVFEDAAEIR 410 10 55.08 2 589.7903

439.83 76614 72 16 22.39 ALELTGLK 362 8 60.44 2 422.7591

439.83 76614 72 16 22.39 NDLAVVDVR 333 9 79.96 2 500.7736

439.83 76614 72 16 22.39 LELQGPR 554 7 54.09 2 406.7327

439.83 76614 72 16 22.39 TLVLSNLSYSATEETLQEVFEK 486 22 47.86 3 834.4289

439.83 76614 72 16 22.39 SISLYYTGEK 457 10 29.21 2 580.7972

439.83 76614 72 16 22.39 GLSEDTTEETLK 577 12 34.65 2 661.8229

439.83 76614 72 16 22.39 VEGTEPTTAFNLFVGNLNFNK 297 21 24.08 3 771.391

439.83 76614 72 16 22.39 GFGFVDFNSEEDAK 610 14 48.26 2 781.3539

439.83 76614 72 16 22.39 TLFVK 572 5 28.22 2 304.1926

439.83 76614 72 16 22.39 KFGYVDFESAEDLEK 347 15 34.64 3 592.948

439.83 76614 72 16 22.39 GGGRGGFGGR 656 10 29.21 2 439.2277

439.83 76614 72 16 22.39 LELTGLK 363 7 0.99 2 387.2404

439.83 76614 72 16 22.39 SLYYTGEK 459 8 1.65 1 960.4668

sp|P43243|MATR3_HUMAN Matrin-3 284.55 94623 58 16 23.49 ITPENLPQILLQLK 132 14 115.49 2 810.4936

284.55 94623 58 16 23.49 IGPYQPNVPVGIDYVIPK 780 18 17.29 2 985.0522

284.55 94623 58 16 23.49 EWSQHINGASHSR 304 13 22.72 3 503.5764

284.55 94623 58 16 23.49 GPSLNPVLDYDHGSR 192 15 20.07 3 542.9365

284.55 94623 58 16 23.49 GDADQASNILASFGLSAR 102 18 17.29 2 896.9496

284.55 94623 58 16 23.49 DLSAAGIGLLAAATQSLSMPASLGR 19 25 13.6 3 791.087

284.55 94623 58 16 23.49 VIHLSNLPHSGYSDSAVLK 496 19 24.36 4 510.0179

284.55 94623 58 16 23.49 GIDLLK 582 6 28.55 1 658.4062

284.55 94623 58 16 23.49 GNLGAGNGNLQGPR 373 14 26.73 3 442.2231

284.55 94623 58 16 23.49 Carbamidomethyl+C(2) LCSLFYTNEEVAK 804 13 22.72 3 525.256

284.55 94623 58 16 23.49 VETSR 393 5 14.11 1 591.3137

284.55 94623 58 16 23.49 NYILMR 524 6 14.44 2 405.2206

284.55 94623 58 16 23.49 TEEGPTLSYGR 149 11 26.53 2 605.2978

284.55 94623 58 16 23.49 VVHIMDFQR 398 9 48.73 3 382.1965

284.55 94623 58 16 23.49 MSKSFQQSSLSR 0 12 47.96 2 693.3417

284.55 94623 58 16 23.49 RTEEGPTLSYGR 148 12 39.21 2 683.3486

sp|Q6S8J3|POTEE_HUMAN POTE 814.85 121363 78 11 13.58 SYELPDGQVITIGNER 938 16 177.16 2 895.9453

ankyrin domain family member E 814.85 121363 78 11 13.58 AGFAGDDAPR 718 10 122.62 2 488.7239

814.85 121363 78 11 13.58 QEYDESGPSIVHR 1059 13 115.53 3 506.2343

814.85 121363 78 11 13.58 Carbamidomethyl+C(2) LCYVALDFEQEMATAASSSSLEK 915 23 90.4 3 850.7308

814.85 121363 78 11 13.58 ELENFMAIEEMKK 531 13 37.04 3 537.9309

814.85 121363 78 11 13.58 IWHHTFYNELR 784 11 38.42 2 758.381

814.85 121363 78 11 13.58 DLIVMLRDTDVNK 155 13 22.72 2 766.4111

814.85 121363 78 11 13.58 Carbamidomethyl+C(2) RCQLNVLDNK 195 10 29.21 3 420.5532

814.85 121363 78 11 13.58 GYRFTTMAER 896 10 30.97 2 616.2979

814.85 121363 78 11 13.58 LMAKALLLYGADIESK 252 16 25.55 3 579.3242

814.85 121363 78 11 13.58 LTSEEESQRLK 498 11 29.54 2 660.35

sp|A5A3E0|POTEF_HUMAN POTE 731.61 121445 68 10 9.67 SYELPDGQVITIGNER 938 16 177.16 2 895.9453

ankyrin domain family member F 731.61 121445 68 10 9.67 AGFAGDDAPR 718 10 122.62 2 488.7239

731.61 121445 68 10 9.67 QEYDESGPSIVHR 1059 13 115.53 3 506.2343

731.61 121445 68 10 9.67 ELENFMAIEEMKK 531 13 37.04 3 537.9309

731.61 121445 68 10 9.67 EKDILHENSTLR 640 12 31.63 3 485.5864

731.61 121445 68 10 9.67 IWHHTFYNELR 784 11 38.42 2 758.381

731.61 121445 68 10 9.67 DLIVMLRDTDVNK 155 13 22.72 2 766.4111

731.61 121445 68 10 9.67 ELPDYLMKILTEHGYR 883 16 33.55 3 660.0011

731.61 121445 68 10 9.67 Phosphoryl STY(8) DILHENSTLR 642 10 58.09 3 426.531

731.61 121445 68 10 9.67 Oxidation+M(17) EKDILHENSTLREEIAMLR 640 19 16.58 3 771.7264

sp|P62263|RS14_HUMAN 153.02 16272 19 4 22.52 TPGPGAQSALR 106 11 38.29 2 527.7856

40S ribosomal protein S14 153.02 16272 19 4 22.52 IEDVTPIPSDSTR 128 13 67.18 2 715.3652

153.02 16272 19 4 22.52 ELGITALHIK 86 10 39.21 2 547.8332

153.02 16272 19 4 22.52 ELGITALHIK 86 10 1.65 3 359.5492

sp|Q6NXT2|H3C_HUMAN Histone H3.3C 119.49 15213 21 5 20 YRPGTVALR 40 9 64.09 2 516.8022

119.49 15213 21 5 20 STELLIR 56 7 57.1 2 416.2484

119.49 15213 21 5 20 DIQLAR 122 6 28.55 2 358.2087

119.49 15213 21 5 20 LPFQR 64 5 14.11 1 660.3884

119.49 15213 21 5 20 YRPGTVALR 40 9 32.86 2 556.7754

sp|P67809|YBOX1_HUMAN 160.51 35924 22 6 24.38 GAEAANVTGPGGVPVQGSK 118 19 65.28 2 848.4415

Nuclease-sensitive element-binding protein 1 160.51 35924 22 6 24.38 RPQYSNPPVQGEVMEGADNQGAGEQGRPVR 204 30 46.62 4 806.6352

160.51 35924 22 6 24.38 NHYR 142 4 13.78 1 589.2839

160.51 35924 22 6 24.38 WFNVR 64 5 28.22 2 361.1903

160.51 35924 22 6 24.38 NEGSESAPEGQAQQR 170 15 20.07 3 529.8956

160.51 35924 22 6 24.38 VLGTVK 58 6 14.44 1 616.404

sp|P84103|SRSF3_HUMAN 156.9 19329 26 7 32.32 AFGYYGPLR 28 9 65.85 2 522.2685

Serine/arginine-rich splicing factor 3 156.9 19329 26 7 32.32 NPPGFAFVEFEDPR 43 14 30.47 2 811.391

156.9 19329 26 7 32.32 NPPGFAFVEFEDPRDAADAVR 43 21 33.19 3 774.0381

156.9 19329 26 7 32.32 VYVGNLGNNGNK 11 12 34.65 2 624.8237

156.9 19329 26 7 32.32 SVWVAR 37 6 14.44 2 359.2056

156.9 19329 26 7 32.32 VYVGNLGNNGNKTELER 11 17 30.07 2 938.9742

156.9 19329 26 7 32.32 GYYGPLR 30 7 1.32 1 825.4197

sp|P07910|HNRPC_HUMAN 330.53 33670 47 9 27.12 VFIGNLNTLVVK 17 12 115.19 2 658.8963

Heterogeneous nuclear ribonucleoproteins C1/C2 330.53 33670 47 9 27.12 GFAFVQYVNER 50 11 104.86 2 665.3336

330.53 33670 47 9 27.12 MIAGQVLDINLAAEPK 73 16 79.19 2 841.9642

330.53 33670 47 9 27.12 VDSLLENLEK 206 10 42.53 2 580.3163

330.53 33670 47 9 27.12 DYYDR 130 5 28.22 2 366.1531

330.53 33670 47 9 27.12 ELTQIK 198 6 14.44 2 366.2153

330.53 33670 47 9 27.12 GDDQLELIKDDEK 275 13 22.72 3 506.5803

330.53 33670 47 9 27.12 LKGDDLQAIK 187 10 29.21 2 550.8171

330.53 33670 47 9 27.12 IGNLNTLVVK 19 10 18.77 2 535.8332

sp|O43852|CALU_HUMAN Calumenin 252.67 37106 30 6 23.17 HLVYESDQNKDGK 271 13 35.28 3 511.58

252.67 37106 30 6 23.17 TFDQLTPEESKER 59 13 31.97 3 527.2577

252.67 37106 30 6 23.17 EQFVEFR 234 7 42.99 2 477.7392

252.67 37106 30 6 23.17 DWILPSDYDHAEAEAR 255 16 30.42 3 629.9601

252.67 37106 30 6 23.17 EEIVDKYDLFVGSQATDFGEALVR 287 24 46.81 3 901.1198

252.67 37106 30 6 23.17 DQLTPEESKER 61 11 2.65 2 666.3242

sp|Q16629|SRSF7_HUMAN 152.47 27366 30 10 26.89 NPPGFAFVEFEDPRDAEDAVR 44 21 22.01 3 793.3737

Serine/arginine-rich splicing factor 7 152.47 27366 30 10 26.89 NPPGFAFVEFEDPR 44 14 30.47 2 811.391

152.47 27366 30 10 26.89 VYVGNLGTGAGK 12 12 39.21 2 568.3107

152.47 27366 30 10 26.89 AFSYYGPLR 29 9 68.86 2 537.2756

152.47 27366 30 10 26.89 Carbamidomethyl+C(3) VICGSR 70 6 28.55 1 691.3524

152.47 27366 30 10 26.89 SGSIK 180 5 14.11 1 491.2785

152.47 27366 30 10 26.89 GELER 24 5 14.11 1 603.3053

152.47 27366 30 10 26.89 YGGETKVYVGNLGTGAGK 6 18 26.22 3 590.969

152.47 27366 30 10 26.89 SYYGPLR 31 7 1.32 1 855.4268

152.47 27366 30 10 26.89 GNLGTGAGK 15 9 2.32 1 774.4143

sp|P24534|EF1B_HUMAN 136.08 24763 25 5 23.56 SPAGLQVLNDYLADK 7 15 47.62 2 802.4156

Elongation factor 1-beta 136.08 24763 25 5 23.56 SSILLDVKPWDDETDMAK 139 18 36.24 3 688.3376

136.08 24763 25 5 23.56 ASLPGVK 66 7 42.99 2 336.208

136.08 24763 25 5 23.56 SIQADGLVWGSSK 163 13 22.72 2 674.3559

136.08 24763 25 5 23.56 SSILLDVKPWDDET 139 14 4.3 2 809.4061

sp|P26368|U2AF2_HUMAN 168.53 53501 31 9 14.95 Carbamidomethyl+C(16) SIEIPRPVDGVEVPGCGK 413 18 44.17 3 636.9941

Splicing factor U2AF 65 kDa subunit 168.53 53501 31 9 14.95 LFIGGLPNYLNDDQVK 260 16 27.39 2 903.4799

168.53 53501 31 9 14.95 ELLTSFGPLK 276 10 55.08 2 552.8191

168.53 53501 31 9 14.95 NFAFLEFR 195 8 35.54 2 522.2698

168.53 53501 31 9 14.95 Carbamidomethyl+C(11) IFVEFTSVFDCQK 431 13 37.04 4 405.6987

168.53 53501 31 9 14.95 QLNENK 9 6 14.44 1 745.3836

168.53 53501 31 9 14.95 Carbamidomethyl+C(13) IPRPVDGVEVPGCGK 416 15 4.3 3 527.2776

168.53 53501 31 9 14.95 Carbamidomethyl+C(14) EIPRPVDGVEVPGCGK 415 16 4.3 2 854.9413

168.53 53501 31 9 14.95 Carbamidomethyl+C(12) PRPVDGVEVPGCGK 417 14 7.27 2 733.8726

sp|Q13151|ROA0_HUMAN 113.35 30840 20 4 19.02 GDVAEGDLIEHFSQFGTVEK 106 20 50.91 3 726.6834

Heterogeneous nuclear ribonucleoprotein A0 113.35 30840 20 4 19.02 LFIGGLNVQTSESGLR 8 16 37.83 2 845.9634

113.35 30840 20 4 19.02 LFVGGLK 99 7 54.09 2 367.2332

113.35 30840 20 4 19.02 GFGFVYFQNHDAADK 139 15 20.07 3 572.5997

sp|P14314|GLU2B_HUMAN 196.22 59425 28 7 14.96 LGGSPTSLGTWGSWIGPDHDK 438 21 41.74 3 723.3507

Glucosidase 2 subunit beta 196.22 59425 28 7 14.96 SLEDQVEMLR 167 10 29.21 2 610.3064

196.22 59425 28 7 14.96 Carbamidomethyl+C(7) YEQGTGCWQGPNR 464 13 22.72 2 776.8395

196.22 59425 28 7 14.96 Carbamidomethyl+C(3) LLCGKETMVTSTTEPSR 482 17 30.07 2 955.4635

196.22 59425 28 7 14.96 VWAAIR 271 6 28.55 2 358.2154

196.22 59425 28 7 14.96 ESLQQMAEVTR 124 11 26.53 2 646.3197

196.22 59425 28 7 14.96 KSLEDQVEMLR 166 11 26.53 2 674.3505

***LMNA*-KD_2**

**Protein score^b^ avg Matched Matched seq peptide seq^h^ seq seq score^m^ z^n^ mz^o^**

**Name^a^ Mass^c^ Products^d^ Peptides^e^ Cover(%)^f^  modification^g^ Start^i^ Length^l^**

sp|P60174|TPIS_HUMAN 1420.5 26669 169 20 73.9 VVLAYEPVWAIGTGK 160 15 232.14 2 801.9468

Triosephosphate isomerase 1420.5 26669 169 20 73.9 Carbamidomethyl+C(2) DCGATWVVLGHSER 85 14 150.41 3 529.5792

1420.5 26669 169 20 73.9 Carbamidomethyl+C(12) IIYGGSVTGATCK 206 13 85.38 2 663.8387

1420.5 26669 169 20 73.9 SNVSDAVAQSTR 194 12 121.88 2 617.8056

1420.5 26669 169 20 73.9 ELASQPDVDGFLVGGASLKPEFVDIINAK 219 29 187.64 3 1010.535

1420.5 26669 169 20 73.9 VVFEQTK 142 7 43.34 2 425.7341

1420.5 26669 169 20 73.9 HVFGESDELIGQK 100 13 162.56 2 729.8666

1420.5 26669 169 20 73.9 Carbamidomethyl+C(9) VPADTEVVCAPPTAYIDFAR 33 20 182.8 3 731.357

1420.5 26669 169 20 73.9 Carbamidomethyl+C(14) VAHALAEGLGVIACIGEK 113 18 178.74 3 603.3245

1420.5 26669 169 20 73.9 QSLGELIGTLNAAK 19 14 79.17 2 707.8991

1420.5 26669 169 20 73.9 VTNGAFTGEISPGMIK 69 16 80.07 2 811.417

1420.5 26669 169 20 73.9 LDEREAGITEK 131 11 29.85 2 630.8301

1420.5 26669 169 20 73.9 KQSLGELIGTLNAAK 18 15 62.83 3 514.9611

1420.5 26669 169 20 73.9 RHVFGESDELIGQK 99 14 43.35 3 538.9417

1420.5 26669 169 20 73.9 GATWVVLGHSER 87 12 7.59 2 656.3456

1420.5 26669 169 20 73.9 PPTAYIDFAR 43 10 7.3 2 575.7978

1420.5 26669 169 20 73.9 Carbamidomethyl+C(1) CGATWVVLGHSER 86 13 7.59 3 491.2411

1420.5 26669 169 20 73.9 PVWAIGTGK 166 9 3.22 2 464.7664

1420.5 26669 169 20 73.9 TWVVLGHSER 89 10 2.9 2 592.3119

1420.5 26669 169 20 73.9 Carbamidomethyl+C(9) VPADTEVVCAPPTAYIDFAR 33 20 4.83 3 725.3597

sp|P07737|PROF1_HUMAN Profilin-1 574.27 15054 79 11 55 TFVNITPAEVGVLVGK 38 16 178.77 2 822.4682

574.27 15054 79 11 55 STGGAPTFNVTVTK 91 14 121.2 2 690.358

574.27 15054 79 11 55 DSPSVWAAVPGK 26 12 89.32 2 607.3139

574.27 15054 79 11 55 SSFYVNGLTLGGQK 56 14 158.9 2 735.8819

574.27 15054 79 11 55 EGVHGGLINK 116 10 43.76 2 512.2825

574.27 15054 79 11 55 TLVLLMGK 108 8 43.66 2 437.7699

574.27 15054 79 11 55 STGGAPTFNVTVTKTDK 91 17 42.21 3 575.2973

574.27 15054 79 11 55 Carbamidomethyl+C(16);

Oxidation+M(11) AGWNAYIDNLMADGTCQDAAIVGYK 1 25 23.33 3 911.7444

574.27 15054 79 11 55 PSVWAAVPGK 28 10 31.94 2 506.2834

574.27 15054 79 11 55 PTFNVTVTK 96 9 29.36 1 1006.5524

574.27 15054 79 11 55 EGVHGGLINK 116 10 1.61 2 503.2775

sp|P62937|PPIA_HUMAN 805.03 18012 122 15 81.21 FEDENFILK 82 9 96.14 2 577.7865

Peptidyl-prolyl cis-trans isomerase A 805.03 18012 122 15 81.21 Carbamidomethyl+C(7) IIPGFMCQGGDFTR 55 14 113.74 2 799.8754

805.03 18012 122 15 81.21 EGMNIVEAMER 133 11 139.47 2 639.7942

805.03 18012 122 15 81.21 VNPTVFFDIAVDGEPLGR 1 18 208.54 2 973.508

805.03 18012 122 15 81.21 VKEGMNIVEAMER 131 13 82.95 3 502.5816

805.03 18012 122 15 81.21 Carbamidomethyl+C(24) HTGPGILSMANAGPNTNGSQFFICTAK 91 27 192.58 3 931.1135

805.03 18012 122 15 81.21 VSFELFADK 19 9 94.89 2 528.2679

805.03 18012 122 15 81.21 TEWLDGK 118 7 43.34 2 424.706

805.03 18012 122 15 81.21 Carbamidomethyl+C(7) KITIADCGQLE 154 11 76.53 2 624.3175

805.03 18012 122 15 81.21 SIYGEKFEDENFILK 76 15 82.84 3 611.3042

805.03 18012 122 15 81.21 Carbamidomethyl+C(7) IIPGFMCQGGDFTRHNGTGGK 55 21 25.24 4 563.2674

805.03 18012 122 15 81.21 VKEGMNIVEAMER 131 13 27.79 3 502.589

805.03 18012 122 15 81.21 TAENFRALSTGEK 31 13 35.7 2 712.3688

805.03 18012 122 15 81.21 Carbamidomethyl+C(5) PGFMCQGGDFTR 57 12 16.89 2 686.799

805.03 18012 122 15 81.21 DENFILK 84 7 9.5 1 878.4566

sp|P23528|COF1_HUMAN Cofilin-1 933.62 18502 117 12 65.06 YALYDATYETK 81 11 133.45 2 669.3138

933.62 18502 117 12 65.06 NIILEEGKEILVGDVGQTVDDPYATFVK 45 28 352.76 3 1021.5349

933.62 18502 117 12 65.06 LGGSAVISLEGKPL 152 14 117.43 2 670.8907

933.62 18502 117 12 65.06 Carbamidomethyl+C(7) HELQANCYEEVKDR 132 14 30.88 3 597.6058

933.62 18502 117 12 65.06 Carbamidomethyl+C(7) MLPDKDCR 73 8 81.58 2 517.7422

933.62 18502 117 12 65.06 EILVGDVGQTVDDPYATFVK 53 20 95.72 2 1083.5581

933.62 18502 117 12 65.06 KEDLVFIFWAPESAPLK 95 17 78.14 3 664.0247

933.62 18502 117 12 65.06 Carbamidomethyl+C(5) AVLFCLSEDK 34 10 62.77 2 591.2967

933.62 18502 117 12 65.06 Carbamidomethyl+C(7) HELQANCYEEVK 132 12 37.72 3 507.2348

933.62 18502 117 12 65.06 VFNDMK 13 6 28.78 2 377.1822

933.62 18502 117 12 65.06 GDVGQTVDDPYATFVK 57 16 38.53 2 856.4192

933.62 18502 117 12 65.06 NIILEEGKEILVGDVGQTVDDPYATFVK 45 28 7.4 3 1015.5258

sp|P22626|ROA2_HUMAN 929.6 37429 123 23 37.96 IDTIEIITDR 137 10 139.15 2 594.823

Heterogeneous nuclear ribonucleoproteins 929.6 37429 123 23 37.96 GGGGNFGPGPGSNFR 213 15 84.82 2 689.3164

A2/B1 929.6 37429 123 23 37.96 GFGFVTFDDHDPVDK 153 15 170.75 3 565.9217

929.6 37429 123 23 37.96 Carbamidomethyl+C(4) LTDCVVMRDPASK 46 13 80.18 3 497.9108

929.6 37429 123 23 37.96 YHTINGHNAEVR 173 12 134.57 3 470.8963

929.6 37429 123 23 37.96 GGNFGFGDSR 203 10 55.52 2 507.2233

929.6 37429 123 23 37.96 LFIGGLSFETTEESLR 22 16 139.75 2 899.9703

929.6 37429 123 23 37.96 TLETVPLER 3 9 97.9 2 529.2978

929.6 37429 123 23 37.96 QEMQEVQSSR 190 10 80.01 2 611.2809

929.6 37429 123 23 37.96 LFVGGIK 113 7 57.57 2 367.2297

929.6 37429 123 23 37.96 EESGKPGAHVTVK 99 13 35.7 3 446.8982

929.6 37429 123 23 37.96 AVAREESGKPGAHVTVK 95 17 18.36 4 434.7403

929.6 37429 123 23 37.96 GFVTFDDHDPVDK 155 13 28.43 2 746.3434

929.6 37429 123 23 37.96 TFDDHDPVDK 158 10 28.43 2 594.7641

929.6 37429 123 23 37.96 Carbamidomethyl+C(3) TDCVVMRDPASK 47 12 7.78 2 689.8288

929.6 37429 123 23 37.96 FVTFDDHDPVDK 156 12 22.14 2 717.833

929.6 37429 123 23 37.96 ETVPLER 5 7 1.29 1 843.4566

929.6 37429 123 23 37.96 TIEIITDR 139 8 8.85 2 480.768

929.6 37429 123 23 37.96 FGFVTFDDHDPVDK 154 14 3.22 3 546.9185

929.6 37429 123 23 37.96 IDTIEIITDR 137 10 1.61 2 585.8208

929.6 37429 123 23 37.96 GFGFVTFDDHDPVDK 153 15 3.22 3 559.9207

929.6 37429 123 23 37.96 YHTINGHNAEVR 173 12 2.25 3 464.894

929.6 37429 123 23 37.96 QEMQEVQSSR 190 10 1.61 2 602.2763

sp|P62258|1433E_HUMAN 553.46 29173 78 14 37.25 Carbamidomethyl+C(3);

14-3-3 protein epsilon Carbamidomethyl+C(4) LICCDILDVLDK 94 12 132.81 2 738.8764

553.46 29173 78 14 37.25 DSTLIMQLLR 215 10 124.92 2 595.3335

553.46 29173 78 14 37.25 HLIPAANTGESK 106 12 82.33 2 619.3318

553.46 29173 78 14 37.25 EAAENSLVAYK 142 11 57.53 2 597.8053

553.46 29173 78 14 37.25 IISSIEQK 61 8 60.9 2 459.2642

553.46 29173 78 14 37.25 YLAEFATGNDRK 130 12 93.42 3 462.2298

553.46 29173 78 14 37.25 YLAEFATGNDR 130 11 45.05 2 628.8037

553.46 29173 78 14 37.25 VFYYK 118 5 28.46 2 360.1883

553.46 29173 78 14 37.25 AAFDDAIAELDTLSEESYK 196 19 16.82 2 1044.4945

553.46 29173 78 14 37.25 NVIGAR 50 6 14.55 1 629.3731

553.46 29173 78 14 37.25 Carbamidomethyl+C(3);

Carbamidomethyl+C(4) LICCDILDVLDKHLIPAANTGESK 94 24 21.93 4 674.5952

553.46 29173 78 14 37.25 YLAEFATGNDRK 130 12 28.71 3 462.2317

553.46 29173 78 14 37.25 Oxidation+M(7) AASDIAMTELPPTHPIR 153 17 50.55 4 459.7396

553.46 29173 78 14 37.25 AEFATGNDRK 132 10 2.25 2 554.7731

sp|P30086|PEBP1_HUMAN 255.45 21056 39 8 43.85 LYTLVLTDPDAPSR 62 14 63.97 2 780.915

Phosphatidylethanolamine-binding protein 1 255.45 21056 39 8 43.85 VLTPTQVK 39 8 67.35 2 443.2719

255.45 21056 39 8 43.85 WSGPLSLQEVDEQPQHPLHVTYAGAAVDELGK 7 32 27.78 4 868.684

255.45 21056 39 8 43.85 NRPTSISWDGLDSGK 47 15 93.27 3 544.937

255.45 21056 39 8 43.85 YVWLVYEQDRPLK 119 13 23.02 3 570.3028

255.45 21056 39 8 43.85 Oxidation+M(10) EWHHFLVVNMK 82 11 26.84 3 485.9091

255.45 21056 39 8 43.85 WLVYEQDRPLK 121 11 2.57 2 723.886

255.45 21056 39 8 43.85 VLTPTQVK 39 8 0.97 2 434.2683

sp|P09936|UCHL1_HUMAN 411.94 24824 59 12 41.26 Carbamidomethyl+C(17) NEAIQAAHDAVAQEGQCR 135 18 124.71 3 656.6346

Ubiquitin carboxyl-terminal hydrolase 411.94 24824 59 12 41.26 LGFEDGSVLK 105 10 94.24 2 532.7829

isozyme L1 411.94 24824 59 12 41.26 Carbamidomethyl+C(7) FSAVALCK 213 8 72.12 2 448.2357

411.94 24824 59 12 41.26 LGVAGQWR 19 8 97.58 2 443.745

411.94 24824 59 12 41.26 MQLKPMEINPEMLNK 0 15 32.66 3 605.9716

411.94 24824 59 12 41.26 MPFPVNHGASSEDTLLK 178 17 25.35 3 614.9673

411.94 24824 59 12 41.26 VYFMK 78 5 14.23 2 344.1779

411.94 24824 59 12 41.26 QIEELK 65 6 14.55 1 759.4179

411.94 24824 59 12 41.26 EFTER 202 5 14.23 2 341.1606

411.94 24824 59 12 41.26 Carbamidomethyl+C(6) SAVALCK 214 7 26.42 1 748.4013

411.94 24824 59 12 41.26 GVAGQWR 20 7 10.43 1 773.4003

411.94 24824 59 12 41.26 Carbamidomethyl+C(7) FSAVALCK 213 8 0.97 2 439.2302

sp|P16949|STMN1_HUMAN Stathmin 348.96 17302 50 11 55.03 ASGQAFELILSPR 14 13 159.55 2 694.8763

348.96 17302 50 11 55.03 AIEENNNFSK 85 10 80.01 2 583.2776

348.96 17302 50 11 55.03 DLSLEEIQK 43 9 48.67 2 537.7874

348.96 17302 50 11 55.03 SKESVPEFPLSPPK 27 14 36.32 3 514.6082

348.96 17302 50 11 55.03 ESKDPADETEAD 137 12 34.95 2 653.7721

348.96 17302 50 11 55.03 QLAEK 70 5 14.23 1 588.3406

348.96 17302 50 11 55.03 EAQMAAKLER 112 10 39.53 2 573.7945

348.96 17302 50 11 55.03 ESVPEFPLSPPKK 29 13 32.26 2 727.9045

348.96 17302 50 11 55.03 DKHIEEVR 126 8 43.66 2 513.2707

348.96 17302 50 11 55.03 SLEEIQK 45 7 1.29 1 846.4546

348.96 17302 50 11 55.03 EENNNFSK 87 8 1.61 2 491.2217

sp|P62805|H4_HUMAN Histone H4 246.87 11367 40 9 52.43 VFLENVIR 60 8 81.58 2 495.2893

246.87 11367 40 9 52.43 ISGLIYEETR 46 10 62.77 2 590.8119

246.87 11367 40 9 52.43 DNIQGITKPAIR 24 12 81.08 3 442.5843

246.87 11367 40 9 52.43 TVTAMDVVYALKR 80 13 35.7 3 489.6006

246.87 11367 40 9 52.43 DAVTYTEHAK 68 10 39.53 3 378.8493

246.87 11367 40 9 52.43 DAVTYTEHAKR 68 11 33.83 2 645.8191

246.87 11367 40 9 52.43 TAMDVVYALKR 82 11 7.78 2 633.8472

246.87 11367 40 9 52.43 IQGITKPAIR 26 10 2.25 2 548.8448

246.87 11367 40 9 52.43 GITKPAIR 28 8 2.25 2 428.272

sp|P10809|CH60_HUMAN 935.84 61054 148 30 43.28 ISSIQSIVPALEIANAHR 250 18 44.58 3 640.3566

60 kDa heat shock protein 935.84 61054 148 30 43.28 VGLQVVAVK 292 9 110.37 2 456.794

935.84 61054 148 30 43.28 LSDGVAVLK 396 9 58.21 2 451.2682

935.84 61054 148 30 43.28 VGEVIVTK 344 8 72.12 2 422.7571

935.84 61054 148 30 43.28 VTDALNATR 420 9 110.37 2 480.7572

935.84 61054 148 30 43.28 TVIIEQSWGSPK 60 12 131.56 2 672.8636

935.84 61054 148 30 43.28 VGGTSDVEVNEK 405 12 54.17 2 617.3026

935.84 61054 148 30 43.28 GYISPYFINTSK 221 12 48.64 2 695.3578

935.84 61054 148 30 43.28 Carbamidomethyl+C(13) AAVEEGIVLGGGCALLR 429 17 83.94 2 842.9587

935.84 61054 148 30 43.28 ALMLQGVDLLADAVAVTMGPK 37 21 120.22 3 705.0516

935.84 61054 148 30 43.28 GVMLAVDAVIAELKK 142 15 80.63 3 519.6371

935.84 61054 148 30 43.28 LVQDVANNTNEEAGDGTTTATVLAR 96 25 26.15 3 854.0884

935.84 61054 148 30 43.28 IGIEIIKR 462 8 57.89 2 471.3086

935.84 61054 148 30 43.28 IPAMTIAK 473 8 100.59 2 422.7452

935.84 61054 148 30 43.28 IGIEIIK 462 7 40.33 2 393.2557

935.84 61054 148 30 43.28 APGFGDNR 301 8 38.89 2 417.1973

935.84 61054 148 30 43.28 FGADAR 31 6 28.78 1 636.3032

935.84 61054 148 30 43.28 GANPVEIR 133 8 38.89 2 428.2341

935.84 61054 148 30 43.28 Carbamidomethyl+C(1) CIPALDSLTPANEDQK 446 16 19.29 3 591.2905

935.84 61054 148 30 43.28 EIGNIISDAMK 180 11 40.28 2 595.812

935.84 61054 148 30 43.28 DGKTLNDELEIIEGMK 202 16 19.29 2 902.9515

935.84 61054 148 30 43.28 Oxidation+M(12) TLNDELEIIEGMK 205 13 23.02 3 507.5832

935.84 61054 148 30 43.28 MLAVDAVIAELKK 144 13 7.45 2 700.9108

935.84 61054 148 30 43.28 DGVAVLK 398 7 24.98 1 701.4165

935.84 61054 148 30 43.28 SDGVAVLK 397 8 9.5 1 788.4502

935.84 61054 148 30 43.28 DALNATR 422 7 1.29 1 760.3966

935.84 61054 148 30 43.28 LAVDAVIAELKK 145 12 7.45 2 635.3934

935.84 61054 148 30 43.28 GLQVVAVK 293 8 9.5 1 813.5164

935.84 61054 148 30 43.28 AVDAVIAELKK 146 11 22.14 2 578.8536

935.84 61054 148 30 43.28 TDALNATR 421 8 9.5 1 861.4421

sp|P11021|GRP78_HUMAN 946.4 72333 146 29 37.16 VEIIANDQGNR 49 11 108.79 2 614.8161

78 kDa glucose-regulated protein 946.4 72333 146 29 37.16 IINEPTAAAIAYGLDK 197 16 105.09 2 830.4537

946.4 72333 146 29 37.16 IINEPTAAAIAYGLDKR 197 17 53.99 3 605.9974

946.4 72333 146 29 37.16 ITITNDQNR 523 9 72.44 2 537.7784

946.4 72333 146 29 37.16 TFAPEEISAMVLTK 138 14 88.47 2 768.9052

946.4 72333 146 29 37.16 NELESYAYSLK 562 11 93.77 2 658.8254

946.4 72333 146 29 37.16 ELEEIVQPIISK 621 12 74.42 2 699.3984

946.4 72333 146 29 37.16 NQLTSNPENTVFDAK 81 15 62.83 2 839.413

946.4 72333 146 29 37.16 VTHAVVTVPAYFNDAQR 164 17 89.14 3 629.9926

946.4 72333 146 29 37.16 ITPSYVAFTPEGER 60 14 114.75 2 783.8968

946.4 72333 146 29 37.16 LTPEEIER 532 8 35.88 2 493.7626

946.4 72333 146 29 37.16 SQIFSTASDNQPTVTIK 447 17 47.62 2 918.9752

946.4 72333 146 29 37.16 TKPYIQVDIGGGQTK 123 15 28.43 3 535.6248

946.4 72333 146 29 37.16 FEELNMDLFR 326 10 39.53 2 657.3159

946.4 72333 146 29 37.16 TWNDPSVQQDIK 101 12 28.71 2 715.8503

946.4 72333 146 29 37.16 VYEGERPLTK 464 10 46.77 3 397.8772

946.4 72333 146 29 37.16 LSSEDK 585 6 28.78 1 678.3369

946.4 72333 146 29 37.16 VVEK 118 4 13.91 1 474.2896

946.4 72333 146 29 37.16 FLPFK 113 5 28.46 2 326.1943

946.4 72333 146 29 37.16 EVEK 290 4 13.91 1 504.2658

946.4 72333 146 29 37.16 AVEEK 596 5 28.46 1 575.3025

946.4 72333 146 29 37.16 LGGK 581 4 28.14 1 374.2397

946.4 72333 146 29 37.16 EFFNGK 376 6 14.55 1 741.3633

946.4 72333 146 29 37.16 AKFEELNMDLFR 324 12 37.72 3 504.9192

946.4 72333 146 29 37.16 KSDIDEIVLVGGSTR 352 15 41.06 3 530.2876

946.4 72333 146 29 37.16 PAYFNDAQR 172 9 3.86 2 541.2607

946.4 72333 146 29 37.16 NEPTAAAIAYGLDKR 199 15 3.86 2 795.4213

946.4 72333 146 29 37.16 SQIFSTASDNQPTV 447 14 7.3 2 747.8625

946.4 72333 146 29 37.16 TPEEIER 533 7 10.43 2 437.2232

sp|Q99497|PARK7_HUMAN Protein DJ-1 259.32 19891 48 15 60.85 Carbamidomethyl+C(14) VTVAGLAGKDPVQCSR 32 16 77.98 3 553.2909

259.32 19891 48 15 60.85 Carbamidomethyl+C(5) DVVICPDASLEDAKK 48 15 89.04 3 553.9437

259.32 19891 48 15 60.85 Carbamidomethyl+C(7) GLIAAICAGPTALLAHEIGFGSK 99 23 37.16 3 756.4096

259.32 19891 48 15 60.85 EGPYDVVVLPGGNLGAQNLSESAAVK 63 26 20.6 3 862.1124

259.32 19891 48 15 60.85 GAEEMETVIPVDVMR 12 15 39.93 2 838.4147

259.32 19891 48 15 60.85 ALVILAK 5 7 43.34 2 364.255

259.32 19891 48 15 60.85 DGLILTSR 148 8 53.12 2 437.7515

259.32 19891 48 15 60.85 EILK 89 4 13.91 1 502.3222

259.32 19891 48 15 60.85 Carbamidomethyl+C(5) DVVICPDASLEDAK 48 14 30.88 2 766.3732

259.32 19891 48 15 60.85 Carbamidomethyl+C(8) KGLIAAICAGPTALLAHEIGFGSK 98 24 23.47 3 799.1125

259.32 19891 48 15 60.85 Carbamidomethyl+C(3) VICPDASLEDAKK 50 13 3.22 2 723.3705

259.32 19891 48 15 60.85 Carbamidomethyl+C(2) ICPDASLEDAKK 51 12 3.22 2 673.8376

259.32 19891 48 15 60.85 Carbamidomethyl+C(1) CPDASLEDAKK 52 11 3.22 2 617.298

259.32 19891 48 15 60.85 Carbamidomethyl+C(12) VAGLAGKDPVQCSR 34 14 7.36 2 729.383

259.32 19891 48 15 60.85 PGGNLGAQNLSESAAVK 72 17 6.76 2 806.9254

sp|P05387|RLA2_HUMAN 118.34 11664 34 5 69.57 YVASYLLAALGGNSSPSAK 2 19 80.08 2 934.9937

60S acidic ribosomal protein P2 118.34 11664 34 5 69.57 ILDSVGIEADDDR 25 13 27.79 2 709.3517

118.34 11664 34 5 69.57 NIEDVIAQGIGK 49 12 78.07 2 628.8459

118.34 11664 34 5 69.57 LASVPAGGAVAVSAAPGSAAPAAGSAPAAAEEK 61 33 60.87 3 925.4834

118.34 11664 34 5 69.57 ILDSVGIEADDDRLNK 25 16 38.21 3 591.636

sp|P0C7M2|RA1L3_HUMAN 485.2 34223 78 15 28.44 IEVIEIMTDR 130 10 155.14 2 609.8199

Putative heterogeneous nuclear 485.2 34223 78 15 28.44 GFAFVTFDDHDSVDK 146 15 96.28 3 567.2535

ribonucleoprotein A1-like 3 485.2 34223 78 15 28.44 LFIGGLSFETTDESLR 15 16 145.31 2 892.9597

485.2 34223 78 15 28.44 Carbamidomethyl+C(9) YHTVNGHNCEVR 166 12 85.85 3 495.889

485.2 34223 78 15 28.44 EDSQRPGAHLTVK 92 13 68.14 3 479.914

485.2 34223 78 15 28.44 KLFIGGLSFETTDESLR 14 17 51.25 3 638.3349

485.2 34223 78 15 28.44 ALSKQEMASASSSQR 179 15 32.66 3 527.6001

485.2 34223 78 15 28.44 RGFAFVTFDDHDSVDK 145 16 19.29 3 619.2901

485.2 34223 78 15 28.44 KIFVGGIK 105 8 70.36 2 431.2801

485.2 34223 78 15 28.44 IFVGGIKEDTEEHHLR 106 16 19.29 4 490.7322

485.2 34223 78 15 28.44 AFVTFDDHDSVDK 148 13 7.45 2 748.3413

485.2 34223 78 15 28.44 FVTFDDHDSVDK 149 12 7.45 2 712.8228

485.2 34223 78 15 28.44 VTFDDHDSVDK 150 11 22.14 2 639.29

485.2 34223 78 15 28.44 Carbamidomethyl+C(8) HTVNGHNCEVR 167 11 8.03 3 441.5434

485.2 34223 78 15 28.44 VIEIMTDR 132 8 1.61 1 976.5075

sp|P07910|HNRPC_HUMAN 402.46 33670 58 9 26.47 VFIGNLNTLVVK 17 12 116.1 2 658.8971

Heterogeneous nuclear ribonucleoproteins C1/C2 402.46 33670 58 9 26.47 GFAFVQYVNER 50 11 155.47 2 665.3341

402.46 33670 58 9 26.47 VDSLLENLEK 206 10 29.53 2 580.3156

402.46 33670 58 9 26.47 MIAGQVLDINLAAEPK 73 16 76.93 2 841.9642

402.46 33670 58 9 26.47 GDDLQAIKK 189 9 48.67 2 494.275

402.46 33670 58 9 26.47 DYYDR 130 5 14.23 2 366.1535

402.46 33670 58 9 26.47 ELTQIK 198 6 14.55 2 366.2168

402.46 33670 58 9 26.47 KSDVEAIFSK 29 10 90.44 2 562.3021

402.46 33670 58 9 26.47 LKGDDLQAIK 187 10 29.53 2 550.8168

sp|P60660|MYL6_HUMAN 257.89 16930 32 6 40.4 ALGQNPTNAEVLK 37 13 59.97 2 677.8701

Myosin light polypeptide 6 257.89 16930 32 6 40.4 HVLVTLGEK 110 9 64.66 2 498.2954

257.89 16930 32 6 40.4 VLDFEHFLPMLQTVAK 63 16 54.49 3 630.0027

257.89 16930 32 6 40.4 EAFQLFDR 13 8 67.35 2 513.2568

257.89 16930 32 6 40.4 NKDQGTYEDYVEGLR 79 15 20.35 3 596.2809

257.89 16930 32 6 40.4 PMLQTVAK 71 8 3.54 2 444.253

sp|P25398|RS12_HUMAN 253.35 14515 27 5 25.76 Carbamidomethyl+C(6) LVEALCAEHQINLIK 63 15 88.5 3 584.3169

40S ribosomal protein S12 253.35 14515 27 5 25.76 Carbamidomethyl+C(8) LGEWVGLCK 84 9 86.68 2 531.2747

253.35 14515 27 5 25.76 TALIHDGLAR 23 10 72.77 2 533.8016

253.35 14515 27 5 25.76 Carbamidomethyl+C(4) EALCAEHQINLIK 65 13 16.12 2 769.906

253.35 14515 27 5 25.76 Carbamidomethyl+C(5) VEALCAEHQINLIK 64 14 3.22 2 819.4303

sp|P61981|1433G_HUMAN 361.26 28302 50 8 39.27 VISSIEQK 61 8 57.89 2 452.2559

14-3-3 protein gamma 361.26 28302 50 8 39.27 DSTLIMQLLR 217 10 124.92 2 595.3335

361.26 28302 50 8 39.27 YLAEVATGEK 132 10 58.53 2 540.7823

361.26 28302 50 8 39.27 AYSEAHEISK 152 10 39.53 2 567.7809

361.26 28302 50 8 39.27 NVTELNEPLSNEER 28 14 44.14 2 822.4017

361.26 28302 50 8 39.27 Carbamidomethyl+C(6) ELEAVCQDVLSLLDNYLIK 91 19 59.64 3 745.7254

361.26 28302 50 8 39.27 Carbamidomethyl+C(22) LGLALNYSVFYYEIQNAPEQACHLAK 172 26 13.44 3 1004.8439

361.26 28302 50 8 39.27 SEAHEISK 154 8 8.85 2 450.7228

sp|P27348|1433T_HUMAN 14-3-3 protein theta 418.3 27764 49 13 35.51 VISSIEQK 60 8 57.89 2 452.2559

418.3 27764 49 13 35.51 DSTLIMQLLR 212 10 124.92 2 595.3335

sp|P27348|1433T_HUMAN 14-3-3 protein theta 418.3 27764 49 13 35.51 Carbamidomethyl+C(3) SICTTVLELLDK 91 12 122.55 2 696.3775

418.3 27764 49 13 35.51 AVTEQGAELSNEER 27 14 66.03 2 766.8666

418.3 27764 49 13 35.51 Carbamidomethyl+C(7) YLAEVACGDDRK 127 12 31.94 3 466.2199

418.3 27764 49 13 35.51 YLIANATNPESK 103 12 37.72 3 440.896

418.3 27764 49 13 35.51 TAFDEAIAELDTLNEDSYK 193 19 16.82 4 537.0078

418.3 27764 49 13 35.51 ISSIEQK 61 7 0.97 1 804.4455

418.3 27764 49 13 35.51 Carbamidomethyl+C(1) CTTVLELLDK 93 10 8.03 2 596.3243

418.3 27764 49 13 35.51 TLIMQLLR 214 8 1.61 2 494.3081

418.3 27764 49 13 35.51 YLIANATNPESK 103 12 2.25 3 435.2181

418.3 27764 49 13 35.51 DSTLIMQLLR 212 10 1.61 2 586.3305

418.3 27764 49 13 35.51 VISSIEQK 60 8 0.97 2 443.2566

sp|P30101|PDIA3_HUMAN 625.98 56782 99 21 40.59 LAPEYEAAATR 62 11 69.54 2 596.3057

Protein disulfide-isomerase A3 625.98 56782 99 21 40.59 ELSDFISYLQR 471 11 75.28 2 685.8536

625.98 56782 99 21 40.59 TFSHELSDFGLESTAGEIPVVAIR 305 24 128.69 3 859.1094

625.98 56782 99 21 40.59 LNFAVASR 296 8 57.89 2 439.2461

625.98 56782 99 21 40.59 EATNPPVIQEEKPK 482 14 85.19 3 527.28

625.98 56782 99 21 40.59 TADGIVSHLK 119 10 62.77 2 520.7876

625.98 56782 99 21 40.59 MDATANDVPSPYEVR 433 15 32.66 2 832.8884

625.98 56782 99 21 40.59 FISDKDASIVGFFDDSFSEAHSEFLK 147 26 42.85 4 735.3462

625.98 56782 99 21 40.59 GIVPLAK 75 7 43.34 2 349.2313

625.98 56782 99 21 40.59 QAGPASVPLR 130 10 65.78 2 498.2874

625.98 56782 99 21 40.59 DASIVGFFDDSFSEAHSEFLK 152 21 22.23 3 783.3637

625.98 56782 99 21 40.59 DLLIAYYDVDYEK 258 13 27.79 2 810.3932

625.98 56782 99 21 40.59 Carbamidomethyl+C(3);

Carbamidomethyl+C(10) VDCTANTNTCNK 82 12 28.71 2 699.303

625.98 56782 99 21 40.59 GFPTIYFSPANK 448 12 24.74 3 447.892

625.98 56782 99 21 40.59 DLIQGK 252 6 14.55 1 673.3842

625.98 56782 99 21 40.59 AASNLR 173 6 28.78 1 631.3541

625.98 56782 99 21 40.59 FLDAGHKLNFAVASR 289 15 28.43 2 823.4496

625.98 56782 99 21 40.59 TVAYTEQKMTSGK 218 13 30.5 3 481.908

625.98 56782 99 21 40.59 PEYEAAATR 64 9 1.93 2 504.2455

625.98 56782 99 21 40.59 PPVIQEEKPK 486 10 45.05 2 582.834

625.98 56782 99 21 40.59 ELSDFISYLQR 471 11 1.93 2 676.8443

sp|P27797|CALR_HUMAN Calreticulin 284.58 48141 44 9 24.7 IKDPDASKPEDWDER 207 15 39.93 3 600.9507

284.58 48141 44 9 24.7 FYALSASFEPFSNK 73 14 73.27 2 804.3904

284.58 48141 44 9 24.7 Carbamidomethyl+C(7) HEQNIDCGGGYVK 98 13 72.4 3 492.8897

284.58 48141 44 9 24.7 FVLSSGK 48 7 43.34 2 369.2114

284.58 48141 44 9 24.7 VHVIFNYK 143 8 35.88 2 510.2861

284.58 48141 44 9 24.7 IDNSQVESGSLEDDWDFLPPKK 185 22 15.07 3 840.4049

284.58 48141 44 9 24.7 IDDPTDSKPEDWDKPEHIPDPDAK 224 24 31.08 5 552.8518

284.58 48141 44 9 24.7 KDPDASKPEDWDER 208 14 3.22 3 563.2584

284.58 48141 44 9 24.7 LEDDWDFLPPKK 195 12 5.47 2 751.8787

sp|P31946|1433B_HUMAN 260.27 28082 35 5 21.14 VISSIEQK 62 8 57.89 2 452.2559

14-3-3 protein beta/alpha 260.27 28082 35 5 21.14 DSTLIMQLLR 214 10 124.92 2 595.3335

260.27 28082 35 5 21.14 AVTEQGHELSNEER 29 14 34.84 3 533.5828

260.27 28082 35 5 21.14 YLIPNATQPESK 105 12 35.66 2 680.8606

260.27 28082 35 5 21.14 QQMGKEYR 77 8 38.89 2 520.2516

sp|P84103|SRSF3_HUMAN 189.69 19329 26 7 33.54 AFGYYGPLR 28 9 81.9 2 522.2688

Serine/arginine-rich splicing factor 3 189.69 19329 26 7 33.54 NPPGFAFVEFEDPR 43 14 34.05 2 811.3885

189.69 19329 26 7 33.54 VELSNGEKR 77 9 33.19 2 516.2756

189.69 19329 26 7 33.54 VYVGNLGNNGNK 11 12 43.74 2 624.8199

189.69 19329 26 7 33.54 SVWVAR 37 6 14.55 2 359.2039

189.69 19329 26 7 33.54 ELDGR 64 5 14.23 1 589.2914

189.69 19329 26 7 33.54 GYYGPLR 30 7 1.29 1 825.4198

sp|P67936|TPM4_HUMAN 266.15 28521 41 7 25 IQLVEEELDR 55 10 109.44 2 622.3274

Tropomyosin alpha-4 chain 266.15 28521 41 7 25 LVILEGELER 132 10 62.77 2 585.8397

266.15 28521 41 7 25 IQALQQQADEAEDR 13 14 52.75 2 807.8913

266.15 28521 41 7 25 AGLNSLEAVK 1 10 29.53 2 501.2782

266.15 28521 41 7 25 LLSDK 190 5 28.46 1 575.3459

266.15 28521 41 7 25 RIQLVEEELDR 54 11 69.54 2 700.3749

266.15 28521 41 7 25 EKAEGDVAALNR 42 12 65.63 3 424.8853

sp|P63104|1433Z_HUMAN 329.68 27745 52 12 40.82 DSTLIMQLLR 212 10 124.92 2 595.3335

14-3-3 protein zeta/delta 329.68 27745 52 12 40.82 Carbamidomethyl+C(3) DICNDVLSLLEK 91 12 84.63 2 709.8645

329.68 27745 52 12 40.82 YLAEVAAGDDKK 127 12 53.16 3 427.2178

329.68 27745 52 12 40.82 SVTEQGAELSNEER 27 14 29.36 2 774.8658

329.68 27745 52 12 40.82 NLLSVAYK 41 8 72.12 2 454.2604

329.68 27745 52 12 40.82 EMQPTHPIR 158 9 40.97 3 370.1853

329.68 27745 52 12 40.82 NELVQK 3 6 14.55 1 730.4039

329.68 27745 52 12 40.82 GIVDQSQQAYQEAFEISK 139 18 27.8 2 1021.0022

329.68 27745 52 12 40.82 IETELR 85 6 43.02 2 380.7106

329.68 27745 52 12 40.82 QQMAR 75 5 14.23 1 633.3067

sp|P63104|1433Z_HUMAN 329.68 27745 52 12 40.82 YLAEVAAGDDK 127 11 26.84 2 576.2853

14-3-3 protein zeta/delta 329.68 27745 52 12 40.82 YLAEVAAGDDKK 127 12 24.74 2 640.3276

sp|P49458|SRP09_HUMAN 120.01 10111 13 3 27.91 Carbamidomethyl+C(7) VTDDLVCLVYK 41 11 60.54 2 662.8451

Signal recognition particle 9 kDa protein 120.01 10111 13 3 27.91 LYLADPMK 16 8 60.9 2 475.7556

120.01 10111 13 3 27.91 LMVAK 71 5 14.23 1 561.3382

sp|P06748|NPM_HUMAN Nucleophosmin 349.67 32575 67 15 35.37 VDNDENEHQLSLR 32 13 130.66 3 523.5765

349.67 32575 67 15 35.37 GPSSVEDIK 239 9 33.19 2 466.2384

349.67 32575 67 15 35.37 MTDQEAIQDLWQWR 277 14 55.82 2 910.4349

349.67 32575 67 15 35.37 TVSLGAGAKDELHIVEAEAMNYEGSPIK 45 28 40.44 4 733.1133

349.67 32575 67 15 35.37 MSVQPTVSLGGFEITPPVVLR 80 21 73.77 2 1114.1174

349.67 32575 67 15 35.37 DELHIVEAEAMNYEGSPIK 54 19 47.48 3 715.6738

349.67 32575 67 15 35.37 FINYVK 267 6 28.78 2 392.2197

349.67 32575 67 15 35.37 LLSISGK 134 7 57.57 2 359.2256

349.67 32575 67 15 35.37 SNQNGK 206 6 14.55 1 647.3103

349.67 32575 67 15 35.37 DNDENEHQLSLR 33 12 22.59 2 735.3393

349.67 32575 67 15 35.37 NDENEHQLSLR 34 11 2.57 2 677.8276

349.67 32575 67 15 35.37 SSVEDIK 241 7 9.5 1 777.3955

349.67 32575 67 15 35.37 ENEHQLSLR 36 9 7.78 2 563.2902

349.67 32575 67 15 35.37 GPSSVEDIK 239 9 1.29 2 457.2347

349.67 32575 67 15 35.37 VDNDENEHQLSLR 32 13 2.57 3 517.9071

sp|P62318|SMD3_HUMAN 147.63 13916 18 4 36.51 FLILPDMLK 69 9 49.18 2 545.3205

Small nuclear ribonucleoprotein Sm D3 147.63 13916 18 4 36.51 VAQLEQVYIR 54 10 65.78 2 609.8469

147.63 13916 18 4 36.51 Carbamidomethyl+C(12) VLHEAEGHIVTCETNTGEVYR 8 21 41.94 3 805.3831

147.63 13916 18 4 36.51 NAPMLK 78 6 14.55 1 673.3666

sp|Q01105|SET_HUMAN Protein SET 219.74 33488 26 4 14.48 VEVTEFEDIK 122 10 94.24 2 604.8058

219.74 33488 26 4 14.48 IDFYFDENPYFENK 136 14 48.79 2 920.915

219.74 33488 26 4 14.48 EFHLNESGDPSSK 154 13 77.42 3 482.886

219.74 33488 26 4 14.48 STEIK 167 5 14.23 1 577.3141

sp|P19338|NUCL_HUMAN Nucleolin 458.64 76614 78 16 20.99 VFGNEIK 370 7 43.34 2 403.7241

458.64 76614 78 16 20.99 EVFEDAAEIR 410 10 55.52 2 589.7903

458.64 76614 78 16 20.99 ALELTGLK 362 8 70.36 2 422.7588

458.64 76614 78 16 20.99 LELQGPR 554 7 43.34 2 406.7338

458.64 76614 78 16 20.99 GIAYIEFK 429 8 54.88 2 470.7601

458.64 76614 78 16 20.99 NDLAVVDVR 333 9 80.66 2 500.7736

458.64 76614 78 16 20.99 SISLYYTGEK 457 10 29.53 2 580.7976

458.64 76614 78 16 20.99 TLVLSNLSYSATEETLQEVFEK 486 22 51.49 3 834.4302

458.64 76614 78 16 20.99 GLSEDTTEETLK 577 12 37.96 2 661.8205

458.64 76614 78 16 20.99 VEGTEPTTAFNLFVGNLNFNK 297 21 38.55 3 771.3882

458.64 76614 78 16 20.99 GFGFVDFNSEEDAK 610 14 46.73 2 781.3531

458.64 76614 78 16 20.99 VAVATPAK 71 8 43.66 2 378.7295

458.64 76614 78 16 20.99 ALVATPGK 116 8 57.89 2 378.7302

458.64 76614 78 16 20.99 TFEEK 444 5 14.23 1 653.3156

458.64 76614 78 16 20.99 VAVATPAKK 71 9 40.97 2 482.764

458.64 76614 78 16 20.99 LELTGLK 363 7 10.43 2 387.2383

sp|P38159|HNRPG_HUMAN 403.81 42331 50 11 22.76 VEQATKPSFESGR 80 13 67.75 3 479.2395

Heterogeneous nuclear ribonucleoprotein G 403.81 42331 50 11 22.76 GFAFVTFESPADAK 49 14 113.63 2 743.8658

403.81 42331 50 11 22.76 IVEVLLMK 33 8 38.89 2 472.7958

403.81 42331 50 11 22.76 GPPPSYGGSSR 298 11 52.3 2 531.2578

403.81 42331 50 11 22.76 ALEAVFGK 22 8 43.66 2 417.7376

403.81 42331 50 11 22.76 LFIGGLNTETNEK 9 13 23.02 2 718.3766

403.81 42331 50 11 22.76 DRDYSDHPSGGSYR 268 14 21.58 3 537.8976

403.81 42331 50 11 22.76 SDLYSSGR 331 8 43.66 2 442.7133

403.81 42331 50 11 22.76 QATKPSFESGR 82 11 7.78 2 604.3061

403.81 42331 50 11 22.76 YSDHPSGGSYR 271 11 2.9 2 613.266

403.81 42331 50 11 22.76 ATKPSFESGR 83 10 2.57 2 540.2806

sp|P07602|SAP_HUMAN 155.99 58112 16 3 7.63 EIVDSYLPVILDIIK 107 15 65.14 2 865.508

Proactivator polypeptide 155.99 58112 16 3 7.63 Carbamidomethyl+C(9) LGPGMADICK 232 10 31.29 2 531.2626

155.99 58112 16 3 7.63 Carbamidomethyl+C(2);

Carbamidomethyl+C(14) TCDWLPKPNMSASCK 92 15 20.35 2 897.8901

sp|Q6S8J3|POTEE_HUMAN POTE 643.37 121363 68 8 7.72 AGFAGDDAPR 718 10 136.14 2 488.7224

ankyrin domain family member E 643.37 121363 68 8 7.72 SYELPDGQVITIGNER 938 16 156 2 895.9456

643.37 121363 68 8 7.72 QEYDESGPSIVHR 1059 13 116.43 3 506.2335

643.37 121363 68 8 7.72 Carbamidomethyl+C(2) LCYVALDFEQEMATAASSSSLEK 915 23 66.5 3 850.7294

643.37 121363 68 8 7.72 GYRFTTMAER 896 10 31.29 2 616.2976

643.37 121363 68 8 7.72 FKGSENSQPEK 389 11 45.05 3 417.5408

643.37 121363 68 8 7.72 Oxidation+M() MVVEVDSMPAASSVK 0 15 20.35 3 522.587

643.37 121363 68 8 7.72 Oxidation+M() SNVGASGDHDDSAMK 77 15 26.37 2 753.8038

sp|P14866|HNRPL_HUMAN 242.51 64132 44 10 18 Carbamidomethyl+C(2) LCFSTAQHAS 579 10 44.3 2 561.2593

Heterogeneous nuclear ribonucleoprotein L 242.51 64132 44 10 18 Carbamidomethyl+C(6) VFNVFCLYGNVEK 398 13 69.51 2 794.8974

242.51 64132 44 10 18 SDALETLGFLNHYQMK 552 16 39.59 3 622.9712

242.51 64132 44 10 18 IEYAKPTR 264 8 57.89 2 489.2732

242.51 64132 44 10 18 AITHLNNNFMFGQK 434 14 21.58 3 545.6058

242.51 64132 44 10 18 LNVFK 272 5 14.23 1 620.3737

242.51 64132 44 10 18 NPNGPYPYTLK 568 11 38.86 2 632.3245

242.51 64132 44 10 18 TPASPVVHIR 97 10 29.53 3 359.5414

242.51 64132 44 10 18 IVIFR 223 5 28.46 2 324.2122

242.51 64132 44 10 18 SERSSSGLLEWESK 538 14 27.02 3 532.2618

sp|P35268|RL22_HUMAN 95.38 14787 11 2 18.75 ITVTSEVPFSK 69 11 54.09 2 604.3327

60S ribosomal protein L22 95.38 14787 11 2 18.75 AGNLGGGVVTIER 52 13 30.5 2 621.8456

sp|Q15056|IF4H_HUMAN 97.28 27385 15 2 12.1 TVATPLNQVANPNSAIFGGARPR 216 23 28.13 3 784.4231

Eukaryotic translation initiation factor 4H 97.28 27385 15 2 12.1 VDIAEGR 112 7 57.57 2 380.2023

sp|P52272|HNRPM_HUMAN 312.84 77515 63 16 25.48 AFITNIPFDVK 72 11 60.54 2 632.8495

Heterogeneous nuclear ribonucleoprotein M 312.84 77515 63 16 25.48 INEILSNALK 371 10 94.24 2 557.8249

312.84 77515 63 16 25.48 ADILEDKDGK 232 10 29.53 2 552.286

312.84 77515 63 16 25.48 GNFGGSFAGSFGGAGGHAPGVAR 627 23 14.6 3 678.986

312.84 77515 63 16 25.48 MGPAMGPALGAGIER 591 15 26.37 2 714.3622

312.84 77515 63 16 25.48 FESPEVAER 698 9 33.19 2 532.2632

312.84 77515 63 16 25.48 Carbamidomethyl+C(2) GCAVVEFK 112 8 43.66 2 455.2268

312.84 77515 63 16 25.48 MGANNLER 557 8 53.12 2 452.7182

312.84 77515 63 16 25.48 MAAPIDR 496 7 43.34 2 387.2021

312.84 77515 63 16 25.48 QGGGGGGGSVPGIER 388 15 20.35 2 642.8191

312.84 77515 63 16 25.48 EVFSMAGVVVR 221 11 38.6 2 597.3303

312.84 77515 63 16 25.48 VGEVTYVELLMDAEGK 94 16 27.4 3 584.9545

312.84 77515 63 16 25.48 HSLSGRPLK 134 9 48.75 2 497.7849

312.84 77515 63 16 25.48 LGGAGMER 410 8 43.66 1 790.3885

312.84 77515 63 16 25.48 MGLERMGANSLER 578 13 30.5 3 488.5739

312.84 77515 63 16 25.48 MGSVERMGSGIER 443 13 42.69 3 470.2258

sp|A5A3E0|POTEF_HUMAN POTE 597.19 121445 60 8 6.88 AGFAGDDAPR 718 10 136.14 2 488.7224

ankyrin domain family member F 597.19 121445 60 8 6.88 SYELPDGQVITIGNER 938 16 156 2 895.9456

597.19 121445 60 8 6.88 QEYDESGPSIVHR 1059 13 116.43 3 506.2335

597.19 121445 60 8 6.88 SNVGTSGDHDDSAMK 40 15 35.04 3 507.5396

597.19 121445 60 8 6.88 ELENFMAIEEMK 531 12 64.63 3 495.2319

597.19 121445 60 8 6.88 ILTEHGYR 891 8 43.66 2 494.758

597.19 121445 60 8 6.88 ALLLYGADIESK 256 12 37.72 3 458.2314

2 597.19 121445 60 8 6.88 QEYDESGPSIVHR 1059 13 2.57 3 500.2317

sp|Q9BRA2|TXD17_HUMAN 143.54 13940 15 4 26.83 VTAVPTLLK 89 9 58.21 2 471.3039

Thioredoxin domain-containing protein 17 143.54 13940 15 4 26.83 Carbamidomethyl+C(3);

Carbamidomethyl+C(6) SWCPDCVQAEPVVR 40 14 29.36 2 851.8913

143.54 13940 15 4 26.83 TIFAYFTGSK 25 10 55.52 2 567.7942

143.54 13940 15 4 26.83 AVPTLLK 91 7 9.5 1 741.482

sp|P24534|EF1B_HUMAN 104.8 24763 23 7 28 LVPVGYGIK 176 9 48.75 2 473.2902

Elongation factor 1-beta 104.8 24763 23 7 28 SSILLDVKPWDDETDMAK 139 18 29.56 3 688.3366

104.8 24763 23 7 28 SIQADGLVWGSSK 163 13 35.7 2 674.3489

104.8 24763 23 7 28 ASLPGVK 66 7 43.34 2 336.2063

104.8 24763 23 7 28 Carbamidomethyl+C(4) LEECVR 157 6 14.55 2 403.2002

104.8 24763 23 7 28 EERLAQYESK 119 10 31.29 3 418.2106

104.8 24763 23 7 28 PVGYGIK 178 7 1.29 2 367.2131

sp|Q15417|CNN3_HUMAN Calponin-3 213.86 36413 24 8 21.88 GPSYGLSAEVK 6 11 41.87 2 554.2885

213.86 36413 24 8 21.88 Carbamidomethyl+C(6) DGIILCELINK 53 11 72.55 2 644.3506

213.86 36413 24 8 21.88 GASQAGMLAPGTR 212 13 49.5 2 608.8125

213.86 36413 24 8 21.88 YDHQAEEDLR 23 10 39.53 3 425.8568

213.86 36413 24 8 21.88 HLYDPK 186 6 14.55 1 772.3928

213.86 36413 24 8 21.88 DIYDQK 226 6 14.55 2 391.1918

213.86 36413 24 8 21.88 GFHTTIDIGVKYAEK 132 15 32.66 2 839.9412

213.86 36413 24 8 21.88 SQAGMLAPGTR 214 11 2.57 2 544.7833

sp|Q16629|SRSF7_HUMAN 121.64 27366 24 6 21.43 NPPGFAFVEFEDPR 44 14 34.05 2 811.3885

Serine/arginine-rich splicing factor 7 121.64 27366 24 6 21.43 VYVGNLGTGAGK 12 12 28.71 2 568.3092

121.64 27366 24 6 21.43 AFSYYGPLR 29 9 49.18 2 537.2762

121.64 27366 24 6 21.43 SPSPK 214 5 28.46 1 515.277

121.64 27366 24 6 21.43 TVWIAR 38 6 14.55 1 745.438

121.64 27366 24 6 21.43 GELER 24 5 28.46 1 603.3044

sp|P14314|GLU2B_HUMAN 238.61 59425 39 11 19.7 LWEEQLAAAK 196 10 36.52 2 579.8105

Glucosidase 2 subunit beta 238.61 59425 39 11 19.7 ILIEDWK 142 7 43.34 2 458.7594

238.61 59425 39 11 19.7 ESLQQMAEVTR 124 11 33.83 2 646.3194

238.61 59425 39 11 19.7 SLEDQVEMLR 167 10 55.52 2 610.3041

238.61 59425 39 11 19.7 LGGSPTSLGTWGSWIGPDHDK 438 21 15.59 3 723.3506

238.61 59425 39 11 19.7 ETMVTSTTEPSR 487 12 37.96 2 669.8179

238.61 59425 39 11 19.7 Carbamidomethyl+C(2) LCPFK 426 5 14.23 1 664.3532

238.61 59425 39 11 19.7 FEEAER 376 6 14.55 1 780.358

238.61 59425 39 11 19.7 YRSEALPTDLPAPSAPDLTEPK 279 22 50.94 3 790.065

238.61 59425 39 11 19.7 LGGSPTSLGTWGSWIGPDHDK 438 21 24.04 4 562.7535

238.61 59425 39 11 19.7 Oxidation+M(6) ESLQQMAEVTR 124 11 26.84 2 654.316

sp|Q6NXT2|H3C_HUMAN Histone H3.3C 112.14 15213 19 6 28.15 YRPGTVALR 40 9 49.18 2 516.8005

112.14 15213 19 6 28.15 STELLIR 56 7 40.33 2 416.2481

112.14 15213 19 6 28.15 EIAQDFNTDLR 72 11 29.85 3 441.2194

112.14 15213 19 6 28.15 LPFQR 64 5 14.23 1 660.388

112.14 15213 19 6 28.15 DIQLAR 122 6 43.02 2 358.2069

112.14 15213 19 6 28.15 RPGTVALR 41 8 9.5 2 435.2647

sp|P15121|ALDR_HUMAN Aldose reductase 167.23 35853 27 7 24.05 TTAQVLIR 243 8 67.35 2 451.2727

167.23 35853 27 7 24.05 Carbamidomethyl+C(10) YKPAVNQIECHPYLTQEK 177 18 17.54 4 555.2774

167.23 35853 27 7 24.05 GIVVTAYSPLGSPDRPWAKPEDPSLLEDPR 203 30 26.76 4 816.4211

167.23 35853 27 7 24.05 REELFIVSK 69 9 58.21 2 560.8244

167.23 35853 27 7 24.05 FPMQR 251 5 14.23 1 678.3325

167.23 35853 27 7 24.05 IAENFK 269 6 14.55 2 361.1957

167.23 35853 27 7 24.05 PDRPWAKPEDPSLLEDPR 215 18 8.3 3 706.6978

sp|P62851|RS25_HUMAN 121.23 13742 28 9 39.2 AALQELLSK 85 9 56.96 2 486.7889

40S ribosomal protein S25 121.23 13742 28 9 39.2 LITPAVVSER 66 10 43.76 2 542.8232

121.23 13742 28 9 39.2 LNNLVLFDK 43 9 33.19 2 538.3077

121.23 13742 28 9 39.2 AQVIYTR 104 7 40.33 2 425.7362

121.23 13742 28 9 39.2 AQVIYTRNTK 104 10 29.53 2 597.3323

121.23 13742 28 9 39.2 DKLNNLVLFDK 41 11 26.84 2 659.8738

121.23 13742 28 9 39.2 DPVNKSGGK 20 9 33.19 2 451.2432

121.23 13742 28 9 39.2 LQELLSK 87 7 1.29 2 415.7511

121.23 13742 28 9 39.2 TPAVVSER 68 8 1.61 2 429.7353

sp|Q8NBS9|TXND5_HUMAN 125.83 47628 20 5 9.03 ALAPTWEQLALGLEHSETVK 221 20 36.03 3 731.7229

Thioredoxin domain-containing protein 5 125.83 47628 20 5 9.03 Carbamidomethyl+C(6) IAEVDCTAER 375 10 39.53 2 582.2739

125.83 47628 20 5 9.03 GYPTLLWFR 260 9 33.19 2 576.8155

125.83 47628 20 5 9.03 YNSMEDAK 105 8 43.66 2 519.1841

125.83 47628 20 5 9.03 PTWEQLALGLEHSETVK 224 17 25.59 3 646.664

sp|Q13151|ROA0_HUMAN 93.84 30840 20 5 19.67 GDVAEGDLIEHFSQFGTVEK 106 20 31.83 3 726.681

Heterogeneous nuclear ribonucleoprotein A0 93.84 30840 20 5 19.67 GFGFVYFQNHDAADK 139 15 20.35 3 572.6006

93.84 30840 20 5 19.67 EDIYSGGGGGGSR 176 13 30.5 2 606.2748

93.84 30840 20 5 19.67 LFVGGLK 99 7 54.56 2 367.2312

93.84 30840 20 5 19.67 AAVVK 154 5 14.23 1 487.3205

sp|Q07021|C1QBP_HUMAN 81.98 31362 24 7 17.02 AFVDFLSDEIKEER 80 14 34.05 3 566.6129

Complement component 1 Q 81.98 31362 24 7 17.02 VEEQEPELTSTPNFVVEVIK 154 20 34.46 2 1144.0956

subcomponent-binding protein 81.98 31362 24 7 17.02 AFVDFLSDEIK 80 11 29.85 2 642.3305

81.98 31362 24 7 17.02 AAAPASPFR 20 9 33.19 1 887.4856

81.98 31362 24 7 17.02 LPLLR 1 5 14.23 1 611.4215

81.98 31362 24 7 17.02 VDFLSDEIKEER 82 12 7.59 2 740.3757

81.98 31362 24 7 17.02 DFLSDEIKEER 83 11 2.9 2 690.8331

sp|P26368|U2AF2_HUMAN 177.58 53501 33 9 19.16 Carbamidomethyl+C(16) SIEIPRPVDGVEVPGCGK 413 18 67.04 3 636.9959

Splicing factor U2AF 65 kDa subunit 177.58 53501 33 9 19.16 ELLTSFGPLK 276 10 43.76 2 552.8168

177.58 53501 33 9 19.16 NFAFLEFR 195 8 35.88 2 522.2704

177.58 53501 33 9 19.16 LGGLTQAPGNPVLAVQINQDK 174 21 24.42 3 711.7254

177.58 53501 33 9 19.16 AFNLVK 286 6 14.55 2 346.2074

177.58 53501 33 9 19.16 SVDETTQAMAFDGIIFQGQSLK 203 22 20.81 3 796.0515

177.58 53501 33 9 19.16 SKPLTR 61 6 14.55 1 701.4308

177.58 53501 33 9 19.16 Carbamidomethyl+C(12) PRPVDGVEVPGCGK 417 14 21.85 2 733.8693

177.58 53501 33 9 19.16 ELLTSFGPLK 276 10 1.61 2 543.8154

sp|P63241|IF5A1_HUMAN 155.43 16832 48 9 49.35 IVEMSTSK 39 8 72.12 2 447.7317

Eukaryotic translation initiation factor 5A-1 155.43 16832 48 9 49.35 NDFQLIGIQDGYLSLLQDSGEVR 86 23 73.24 3 860.7726

155.43 16832 48 9 49.35 VHLVGIDIFTGK 55 12 151.02 3 433.5805

155.43 16832 48 9 49.35 HGHAK 50 5 14.23 1 549.2847

155.43 16832 48 9 49.35 Carbamidomethyl+C(4) GRPCKIVEMSTSK 34 13 23.02 3 498.2597

155.43 16832 48 9 49.35 Carbamidomethyl+C(6) KYEDICPSTHNMDVPNIK 67 18 83.61 3 721.0077

155.43 16832 48 9 49.35 RNDFQLIGIQDGYLSLLQDSGEVR 85 24 28.64 3 912.8066

155.43 16832 48 9 49.35 NDFQLIGIQDGYLSLLQDSGEVREDLR 86 27 13.13 5 619.5134

155.43 16832 48 9 49.35 VEMSTSK 40 7 0.97 1 781.3743

sp|O14979|HNRDL_HUMAN 146.21 46437 31 8 15.95 GFGFVLFK 189 8 57.89 2 457.7584

Heterogeneous nuclear ribonucleoprotein D-like 146.21 46437 31 8 15.95 Carbamidomethyl+C(3) GFCFITYTDEEPVKK 274 15 28.43 3 611.9636

146.21 46437 31 8 15.95 Carbamidomethyl+C(7) FGEVVDCTIK 170 10 93.45 2 584.2881

146.21 46437 31 8 15.95 MFIGGLSWDTSK 149 12 28.71 2 671.3304

146.21 46437 31 8 15.95 VLELK 204 5 14.23 1 601.3894

146.21 46437 31 8 15.95 VFVGGLSPDTSEEQIK 234 16 19.29 3 569.2863

146.21 46437 31 8 15.95 MFIGGLSWDTSKK 149 13 42.6 3 490.5826

146.21 46437 31 8 15.95 Carbamidomethyl+C(1) CFITYTDEEPVKK 276 13 7.45 2 815.3966

sp|P67809|YBOX1_HUMAN 94.66 35924 22 7 31.48 GAEAANVTGPGGVPVQGSK 118 19 45.79 2 848.4415

Nuclease-sensitive element-binding protein 1 94.66 35924 22 7 31.48 RPQYSNPPVQGEVMEGADNQGAGEQGRPVR 204 30 12.35 4 806.6355

94.66 35924 22 7 31.48 WFNVR 64 5 28.46 2 361.1914

94.66 35924 22 7 31.48 NFNYR 283 5 28.46 2 357.171

94.66 35924 22 7 31.48 VLGTVK 58 6 14.55 1 616.4036

94.66 35924 22 7 31.48 EDGNEEDKENQGDETQGQQPPQR 256 23 24.68 3 876.7098

94.66 35924 22 7 31.48 RPENPKPQDGKETK 290 14 27.02 3 541.9549

sp|O43852|CALU_HUMAN Calumenin 148.93 37106 25 6 22.22 HLVYESDQNKDGK 271 13 33.06 3 511.582

148.93 37106 25 6 22.22 TFDQLTPEESKER 59 13 23.02 3 527.2542

148.93 37106 25 6 22.22 TFDQLTPEESK 59 11 60.54 2 647.815

148.93 37106 25 6 22.22 Carbamidomethyl+C(5);

Carbamidomethyl+C(9) QFLMCLSLCTAFALSKPTEK 4 20 30.7 4 587.0578

148.93 37106 25 6 22.22 EEIVDKYDLFVGSQATDFGEALVR 287 24 24.46 3 901.1212

148.93 37106 25 6 22.22 DQLTPEESKER 61 11 2.57 2 666.3246

***LMNA*-KD_3**

**Protein score^b^ avg Matched Matched seq peptide seq^h^ seq seq score^m^ z^n^ mz^o^**

**Name^a^ Mass^c^ Products^d^ Peptides^e^ Cover(%)^f^  modification^g^ Start^i^ Length^l^**

sp|P60174|TPIS_HUMAN 1279 26669 166 21 78.31 VVLAYEPVWAIGTGK 160 15 216.01 2 801.9479

Triosephosphate isomerase 1279 26669 166 21 78.31 Carbamidomethyl+C(12) IIYGGSVTGATCK 206 13 80.13 2 663.8384

1279 26669 166 21 78.31 Carbamidomethyl+C(2) DCGATWVVLGHSER 85 14 121.36 3 529.5797

1279 26669 166 21 78.31 SNVSDAVAQSTR 194 12 136.7 2 617.8061

1279 26669 166 21 78.31 VVFEQTK 142 7 43.31 2 425.7337

1279 26669 166 21 78.31 ELASQPDVDGFLVGGASLKPEFVDIINAK 219 29 129.66 3 1010.5342

1279 26669 166 21 78.31 HVFGESDELIGQK 100 13 108.03 3 486.9084

1279 26669 166 21 78.31 Carbamidomethyl+C(9) VPADTEVVCAPPTAYIDFAR 33 20 121.5 3 731.3558

1279 26669 166 21 78.31 QSLGELIGTLNAAK 19 14 109.44 2 707.8987

1279 26669 166 21 78.31 Carbamidomethyl+C(14) VAHALAEGLGVIACIGEK 113 18 216.31 3 603.324

1279 26669 166 21 78.31 VTNGAFTGEISPGMIK 69 16 62.23 2 811.4194

1279 26669 166 21 78.31 LDEREAGITEK 131 11 41.83 2 630.8297

1279 26669 166 21 78.31 KQSLGELIGTLNAAK 18 15 69.07 3 514.9612

1279 26669 166 21 78.31 RHVFGESDELIGQK 99 14 36.29 3 538.9419

1279 26669 166 21 78.31 KFFVGGNWK 5 9 58.18 2 541.7917

1279 26669 166 21 78.31 Carbamidomethyl+C(9) VPADTEVVCAPPTAYIDFARQK 33 22 23.8 3 816.7422

1279 26669 166 21 78.31 Oxidation+M(14) VTNGAFTGEISPGMIK 69 16 19.27 3 546.6097

1279 26669 166 21 78.31 GATWVVLGHSER 87 12 7.58 2 656.3435

1279 26669 166 21 78.31 PPTAYIDFAR 43 10 4.84 2 575.7983

1279 26669 166 21 78.31 Carbamidomethyl+C(3) IACIGEK 124 7 14.44 2 395.7067

1279 26669 166 21 78.31 TWVVLGHSER 89 10 2.9 2 592.3178

sp|P62937|PPIA_HUMAN 828.27 18012 110 14 64.85 FEDENFILK 82 9 96.07 2 577.7855

Peptidyl-prolyl cis-trans isomerase A 828.27 18012 110 14 64.85 VSFELFADK 19 9 86.62 2 528.2695

828.27 18012 110 14 64.85 Carbamidomethyl+C(7) IIPGFMCQGGDFTR 55 14 94.68 2 799.8763

828.27 18012 110 14 64.85 EGMNIVEAMER 133 11 108.72 2 639.7947

828.27 18012 110 14 64.85 Carbamidomethyl+C(24) HTGPGILSMANAGPNTNGSQFFICTAK 91 27 222.31 3 931.1147

828.27 18012 110 14 64.85 VNPTVFFDIAVDGEPLGR 1 18 199.74 2 973.5115

828.27 18012 110 14 64.85 VKEGMNIVEAMER 131 13 68.09 3 502.5821

828.27 18012 110 14 64.85 Carbamidomethyl+C(7) KITIADCGQLE 154 11 108.72 2 624.3184

828.27 18012 110 14 64.85 SIYGEKFEDENFILK 76 15 78.56 3 611.3049

828.27 18012 110 14 64.85 FELFADK 21 7 9.49 1 869.4345

828.27 18012 110 14 64.85 Carbamidomethyl+C(5) PGFMCQGGDFTR 57 12 16.88 2 686.7983

828.27 18012 110 14 64.85 DENFILK 84 7 9.49 1 878.4555

828.27 18012 110 14 64.85 SFELFADK 20 8 9.49 1 956.4684

828.27 18012 110 14 64.85 Carbamidomethyl+C(20) GILSMANAGPNTNGSQFFICTAK 95 23 7.89 3 800.3952

sp|P16949|STMN1_HUMAN Stathmin 420.87 17302 43 7 36.24 ASGQAFELILSPR 14 13 179.69 2 694.879

420.87 17302 43 7 36.24 AIEENNNFSK 85 10 72.72 2 583.2755

420.87 17302 43 7 36.24 DLSLEEIQK 43 9 119.84 2 537.7873

420.87 17302 43 7 36.24 SKESVPEFPLSPPK 27 14 42.01 3 514.6061

420.87 17302 43 7 36.24 DKHIEEVR 126 8 43.63 2 513.2683

420.87 17302 43 7 36.24 SLEEIQK 45 7 24.96 1 846.4527

420.87 17302 43 7 36.24 EENNNFSK 87 8 1.61 2 491.2198

sp|P07737|PROF1_HUMAN Profilin-1 519.77 15054 69 9 62.86 STGGAPTFNVTVTK 91 14 124.12 2 690.3604

519.77 15054 69 9 62.86 TFVNITPAEVGVLVGK 38 16 174.63 2 822.4682

519.77 15054 69 9 62.86 TLVLLMGK 108 8 57.86 2 437.7705

519.77 15054 69 9 62.86 DSPSVWAAVPGK 26 12 78.01 2 607.3147

519.77 15054 69 9 62.86 SSFYVNGLTLGGQK 56 14 59.24 2 735.8816

519.77 15054 69 9 62.86 EGVHGGLINK 116 10 29.51 2 512.2813

519.77 15054 69 9 62.86 DSLLQDGEFSMDLR 75 14 43.31 2 813.3835

519.77 15054 69 9 62.86 PSVWAAVPGK 28 10 31.91 2 506.2854

519.77 15054 69 9 62.86 EGVHGGLINK 116 10 1.61 2 503.2784

sp|P23528|COF1_HUMAN Cofilin-1 934.45 18502 109 12 71.69 YALYDATYETK 81 11 111.73 2 669.316

934.45 18502 109 12 71.69 LGGSAVISLEGKPL 152 14 87.14 2 670.8921

934.45 18502 109 12 71.69 NIILEEGKEILVGDVGQTVDDPYATFVK 45 28 383.91 3 1021.5372

934.45 18502 109 12 71.69 Carbamidomethyl+C(7) HELQANCYEEVKDR 132 14 29.34 3 597.6051

934.45 18502 109 12 71.69 Carbamidomethyl+C(7) MLPDKDCR 73 8 81.53 2 517.7413

934.45 18502 109 12 71.69 NIILEEGK 45 8 53.08 2 458.2584

934.45 18502 109 12 71.69 EILVGDVGQTVDDPYATFVK 53 20 97.31 2 1083.5625

934.45 18502 109 12 71.69 KEDLVFIFWAPESAPLK 95 17 72.42 3 664.0233

934.45 18502 109 12 71.69 Carbamidomethyl+C(5) AVLFCLSEDK 34 10 60.17 2 591.2961

934.45 18502 109 12 71.69 MIYASSKDAIK 114 11 33.81 2 613.8312

934.45 18502 109 12 71.69 VFNDMK 13 6 42.99 2 377.1811

934.45 18502 109 12 71.69 NIILEEGKEILVGDVGQTVDDPYATFVK 45 28 7.42 3 1015.5317

sp|P22626|ROA2_HUMAN 874.33 37429 119 29 49.29 IDTIEIITDR 137 10 155.05 2 594.8229

Heterogeneous nuclear ribonucleoproteins 874.33 37429 119 29 49.29 GGGGNFGPGPGSNFR 213 15 85.3 2 689.3202

A2/B1 874.33 37429 119 29 49.29 GFGFVTFDDHDPVDK 153 15 141.1 3 565.9226

874.33 37429 119 29 49.29 NYYEQWGK 38 8 57.86 2 544.2477

874.33 37429 119 29 49.29 Carbamidomethyl+C(4) LTDCVVMRDPASK 46 13 72.86 3 497.9107

874.33 37429 119 29 49.29 LFVGGIKEDTEEHHLR 113 16 107.33 4 470.7416

874.33 37429 119 29 49.29 GGNFGFGDSR 203 10 55.49 2 507.2272

874.33 37429 119 29 49.29 LFIGGLSFETTEESLR 22 16 70.34 2 899.9703

874.33 37429 119 29 49.29 QEMQEVQSSR 190 10 58.5 2 611.2796

874.33 37429 119 29 49.29 TLETVPLER 3 9 49.15 2 529.2978

874.33 37429 119 29 49.29 LFVGGIK 113 7 54.52 2 367.2316

874.33 37429 119 29 49.29 YHTINGHNAEVR 173 12 40.7 3 470.9024

874.33 37429 119 29 49.29 DYFEEYGK 129 8 43.63 2 525.7268

874.33 37429 119 29 49.29 EQFR 17 4 13.9 1 579.2949

874.33 37429 119 29 49.29 GFGFVTFSSMAEVDAAMAARPHSIDGR 62 27 19.99 4 707.5858

874.33 37429 119 29 49.29 KLFIGGLSFETTEESLR 21 17 36.54 3 643.0091

874.33 37429 119 29 49.29 GFVTFDDHDPVDK 155 13 3.22 2 746.3434

874.33 37429 119 29 49.29 VGGIKEDTEEHHLR 115 14 7.36 3 540.6076

874.33 37429 119 29 49.29 VTFDDHDPVDK 157 11 7.45 2 644.2989

874.33 37429 119 29 49.29 Carbamidomethyl+C(2) DCVVMRDPASK 48 11 2.58 2 639.3058

874.33 37429 119 29 49.29 FVGGIKEDTEEHHLR 114 15 3.55 3 589.6322

874.33 37429 119 29 49.29 TFDDHDPVDK 158 10 7.45 2 594.7676

874.33 37429 119 29 49.29 FVTFDDHDPVDK 156 12 3.22 2 717.833

874.33 37429 119 29 49.29 Carbamidomethyl+C(3) TDCVVMRDPASK 47 12 22.57 2 689.8246

874.33 37429 119 29 49.29 FGFVTFDDHDPVDK 154 14 3.22 3 546.922

874.33 37429 119 29 49.29 ETVPLER 5 7 9.49 1 843.456

874.33 37429 119 29 49.29 IDTIEIITDR 137 10 1.61 2 585.8187

874.33 37429 119 29 49.29 GFGFVTFDDHDPVDK 153 15 3.22 3 559.9225

874.33 37429 119 29 49.29 Carbamidomethyl+C(4) LTDCVVMRDPASK 46 13 2.58 3 491.9045

sp|P62258|1433E_HUMAN 471.27 29173 74 13 42.75 NLLSVAYK 42 8 67.31 2 454.2619

14-3-3 protein epsilon 471.27 29173 74 13 42.75 Carbamidomethyl+C(3);

Carbamidomethyl+C(4) LICCDILDVLDK 94 12 132.72 2 738.8758

471.27 29173 74 13 42.75 DSTLIMQLLR 215 10 121.83 2 595.334

471.27 29173 74 13 42.75 YLAEFATGNDRK 130 12 54.13 3 462.2287

471.27 29173 74 13 42.75 HLIPAANTGESK 106 12 57.14 2 619.3297

471.27 29173 74 13 42.75 YLAEFATGNDR 130 11 54.05 2 628.8027

471.27 29173 74 13 42.75 AAFDDAIAELDTLSEESYK 196 19 16.8 2 1044.498

471.27 29173 74 13 42.75 IISSIEQK 61 8 43.63 2 459.262

471.27 29173 74 13 42.75 AASDIAMTELPPTHPIR 153 17 18.34 3 607.3261

471.27 29173 74 13 42.75 VFYYK 118 5 28.44 2 360.19

471.27 29173 74 13 42.75 NVIGAR 50 6 31.78 1 629.3736

471.27 29173 74 13 42.75 Carbamidomethyl+C(3);

Carbamidomethyl+C(4) LICCDILDVLDKHLIPAANTGESK 94 24 46.46 4 674.5928

471.27 29173 74 13 42.75 AEFATGNDRK 132 10 2.26 2 554.7733

sp|P10809|CH60_HUMAN 1081.45 61054 153 29 36.47 ISSIQSIVPALEIANAHR 250 18 65.05 3 640.3583

60 kDa heat shock protein 1081.45 61054 153 29 36.47 VGLQVVAVK 292 9 96.07 2 456.7944

1081.45 61054 153 29 36.47 LSDGVAVLK 396 9 80.6 2 451.2687

1081.45 61054 153 29 36.47 VTDALNATR 420 9 110.3 2 480.7564

1081.45 61054 153 29 36.47 IGIEIIK 462 7 71.76 2 393.2564

1081.45 61054 153 29 36.47 VGEVIVTK 344 8 81.53 2 422.758

1081.45 61054 153 29 36.47 TVIIEQSWGSPK 60 12 131.47 2 672.8629

1081.45 61054 153 29 36.47 GYISPYFINTSK 221 12 89.26 2 695.3584

1081.45 61054 153 29 36.47 TLNDELEIIEGMK 205 13 35.67 2 752.884

1081.45 61054 153 29 36.47 VGGTSDVEVNEK 405 12 84.58 2 617.3025

1081.45 61054 153 29 36.47 GIIDPTK 516 7 40.3 2 372.2177

1081.45 61054 153 29 36.47 Carbamidomethyl+C(13) AAVEEGIVLGGGCALLR 429 17 101.66 2 842.9587

1081.45 61054 153 29 36.47 GVMLAVDAVIAELKK 142 15 56.76 3 519.6365

1081.45 61054 153 29 36.47 IGIEIIKR 462 8 43.63 2 471.3091

1081.45 61054 153 29 36.47 LVQDVANNTNEEAGDGTTTATVLAR 96 25 25.47 3 854.0885

1081.45 61054 153 29 36.47 ALMLQGVDLLADAVAVTMGPK 37 21 93.4 3 705.0486

1081.45 61054 153 29 36.47 IPAMTIAK 473 8 51.84 2 422.746

1081.45 61054 153 29 36.47 SIDLK 82 5 14.22 1 575.3367

1081.45 61054 153 29 36.47 VGGTSDVEVNEKK 405 13 27.76 2 681.3554

1081.45 61054 153 29 36.47 Oxidation+M(2) IMQSSSEVGYDAMAGDFVNMVEK 493 23 14.58 4 631.786

1081.45 61054 153 29 36.47 MLAVDAVIAELKK 144 13 7.45 2 700.9128

1081.45 61054 153 29 36.47 GEVIVTK 345 7 0.97 1 745.4388

1081.45 61054 153 29 36.47 DALNATR 422 7 1.29 1 760.3923

1081.45 61054 153 29 36.47 DGVAVLK 398 7 40.95 1 701.417

1081.45 61054 153 29 36.47 SDGVAVLK 397 8 58.18 1 788.4494

1081.45 61054 153 29 36.47 AVDAVIAELKK 146 11 22.13 2 578.8529

1081.45 61054 153 29 36.47 GLQVVAVK 293 8 9.49 1 813.5174

1081.45 61054 153 29 36.47 LAVDAVIAELKK 145 12 7.45 2 635.3958

1081.45 61054 153 29 36.47 TDALNATR 421 8 1.29 2 431.2243

sp|P0C7M2|RA1L3_HUMAN 595.76 34223 84 12 38.13 IEVIEIMTDR 130 10 123.59 2 609.8214

Putative heterogeneous nuclear ribonucleoprotein 595.76 34223 84 12 38.13 EDSQRPGAHLTVK 92 13 64.69 3 479.9149

A1-like 3 OS 595.76 34223 84 12 38.13 GFAFVTFDDHDSVDK 146 15 149.67 3 567.255

595.76 34223 84 12 38.13 LFIGGLSFETTDESLR 15 16 88.33 2 892.9593

595.76 34223 84 12 38.13 Carbamidomethyl+C(9) YHTVNGHNCEVR 166 12 90.35 3 495.8887

595.76 34223 84 12 38.13 GFGFVTYATVEEVDAAMNARPHK 55 23 84.27 4 628.302

595.76 34223 84 12 38.13 KLFIGGLSFETTDESLR 14 17 42.19 3 638.3366

595.76 34223 84 12 38.13 RGFAFVTFDDHDSVDK 145 16 38.18 3 619.2908

595.76 34223 84 12 38.13 KIFVGGIK 105 8 67.31 2 431.281

595.76 34223 84 12 38.13 GGNFGGRNSGPYGGGGQYFAKPR 277 23 20.99 3 767.7012

595.76 34223 84 12 38.13 EEVDAAMNARPHK 65 13 5.8 2 734.3569

595.76 34223 84 12 38.13 EDSQRPGAHLTVK 92 13 2.58 3 473.9094

sp|P63104|1433Z_HUMAN 333.92 27745 51 12 38.37 NLLSVAYK 41 8 67.31 2 454.2619

14-3-3 protein zeta/delta 333.92 27745 51 12 38.37 FLIPNASQAESK 103 12 71.36 2 652.8465

333.92 27745 51 12 38.37 DSTLIMQLLR 212 10 121.83 2 595.334

333.92 27745 51 12 38.37 Carbamidomethyl+C(3) DICNDVLSLLEK 91 12 37.68 2 709.8656

333.92 27745 51 12 38.37 GIVDQSQQAYQEAFEISKK 139 19 24.58 3 723.6942

333.92 27745 51 12 38.37 VFYLK 115 5 28.44 2 335.1985

333.92 27745 51 12 38.37 QQMAR 75 5 14.22 1 633.3136

333.92 27745 51 12 38.37 YLAEVAAGDDK 127 11 41.83 2 576.2898

1 333.92 27745 51 12 38.37 IETELR 85 6 42.99 2 380.7096

333.92 27745 51 12 38.37 NELVQK 3 6 14.54 1 730.4175

333.92 27745 51 12 38.37 PNASQAESK 106 9 2.26 1 931.4385

333.92 27745 51 12 38.37 DSTLIMQLLR 212 10 1.61 2 586.3293

sp|P62805|H4_HUMAN Histone H4 353.6 11367 44 10 55.34 VFLENVIR 60 8 72.08 2 495.2894

353.6 11367 44 10 55.34 ISGLIYEETR 46 10 93.39 2 590.8122

353.6 11367 44 10 55.34 DNIQGITKPAIR 24 12 57.14 3 442.5846

353.6 11367 44 10 55.34 DAVTYTEHAK 68 10 72.72 2 567.7767

353.6 11367 44 10 55.34 TVTAMDVVYALKR 80 13 74.35 3 489.6011

353.6 11367 44 10 55.34 GGVK 41 4 13.9 1 360.223

353.6 11367 44 10 55.34 QGITKPAIR 27 9 2.26 2 492.298

353.6 11367 44 10 55.34 TAMDVVYALKR 82 11 2.58 2 633.8492

353.6 11367 44 10 55.34 IQGITKPAIR 26 10 2.26 2 548.8474

353.6 11367 44 10 55.34 GITKPAIR 28 8 8.03 2 428.2735

sp|P09936|UCHL1_HUMAN 474.51 24824 67 10 37.67 LGFEDGSVLK 105 10 79.96 2 532.782

Ubiquitin carboxyl-terminal 474.51 24824 67 10 37.67 MPFPVNHGASSEDTLLK 178 17 30.42 3 614.9744

hydrolase isozyme L1 474.51 24824 67 10 37.67 LGVAGQWR 19 8 97.51 2 443.7463

474.51 24824 67 10 37.67 Carbamidomethyl+C(17) NEAIQAAHDAVAQEGQCR 135 18 131.14 3 656.6366

474.51 24824 67 10 37.67 Carbamidomethyl+C(7) FSAVALCK 213 8 81.53 2 448.2366

474.51 24824 67 10 37.67 MQLKPMEINPEMLNK 0 15 59.56 3 605.972

474.51 24824 67 10 37.67 Carbamidomethyl+C(2) VCREFTER 199 8 35.85 2 548.7643

474.51 24824 67 10 37.67 Oxidation+M(1) MPFPVNHGASSEDTLLK 178 17 25.95 3 620.3105

474.51 24824 67 10 37.67 Carbamidomethyl+C(6) SAVALCK 214 7 26.4 1 748.402

474.51 24824 67 10 37.67 Carbamidomethyl+C(7) FSAVALCK 213 8 0.97 2 439.2339

sp|P61981|1433G_HUMAN 376.04 28302 57 8 35.63 NLLSVAYK 42 8 67.31 2 454.2619

14-3-3 protein gamma 376.04 28302 57 8 35.63 VISSIEQK 61 8 43.63 2 452.2573

376.04 28302 57 8 35.63 DSTLIMQLLR 217 10 121.83 2 595.334

376.04 28302 57 8 35.63 YLAEVATGEK 132 10 39.51 2 540.7818

376.04 28302 57 8 35.63 NVTELNEPLSNEER 28 14 82.12 2 822.4017

376.04 28302 57 8 35.63 Carbamidomethyl+C(6) ELEAVCQDVLSLLDNYLIK 91 19 40.99 3 745.7164

376.04 28302 57 8 35.63 AYSEAHEISK 152 10 31.27 2 567.7792

376.04 28302 57 8 35.63 ATVVESSEK 143 9 100.85 2 475.2436

sp|P11021|GRP78_HUMAN 835.9 72333 140 24 38.84 VEIIANDQGNR 49 11 108.72 2 614.8177

78 kDa glucose-regulated protein 835.9 72333 140 24 38.84 IINEPTAAAIAYGLDKR 197 17 57.5 3 605.9966

835.9 72333 140 24 38.84 IINEPTAAAIAYGLDK 197 16 77.92 2 830.4543

835.9 72333 140 24 38.84 NELESYAYSLK 562 11 70.04 2 658.8233

835.9 72333 140 24 38.84 ITPSYVAFTPEGER 60 14 99.87 2 783.8992

835.9 72333 140 24 38.84 ITITNDQNR 523 9 58.18 2 537.778

835.9 72333 140 24 38.84 ELEEIVQPIISK 621 12 35.62 2 699.398

835.9 72333 140 24 38.84 TWNDPSVQQDIK 101 12 24.71 2 715.8514

835.9 72333 140 24 38.84 TFAPEEISAMVLTK 138 14 73.21 2 768.906

835.9 72333 140 24 38.84 NQLTSNPENTVFDAK 81 15 56.55 2 839.4117

835.9 72333 140 24 38.84 VTHAVVTVPAYFNDAQR 164 17 68.54 3 629.9934

835.9 72333 140 24 38.84 SQIFSTASDNQPTVTIK 447 17 48.63 2 918.9772

835.9 72333 140 24 38.84 FEELNMDLFR 326 10 79.96 2 657.3164

835.9 72333 140 24 38.84 DAGTIAGLNVMR 185 12 40.7 2 609.3207

835.9 72333 140 24 38.84 TKPYIQVDIGGGQTK 123 15 20.33 3 535.6221

835.9 72333 140 24 38.84 SDIDEIVLVGGSTR 353 14 21.55 2 730.8854

835.9 72333 140 24 38.84 IEWLESHQDADIEDFK 601 16 26.97 3 658.9732

835.9 72333 140 24 38.84 VYEGERPLTK 464 10 39.51 3 397.8772

835.9 72333 140 24 38.84 VVEK 118 4 13.9 1 474.2912

835.9 72333 140 24 38.84 KSDIDEIVLVGGSTR 352 15 55.76 3 530.2883

835.9 72333 140 24 38.84 AKFEELNMDLFR 324 12 35.62 3 504.919

835.9 72333 140 24 38.84 LSLVAAMLLLLSAARAEEEDK 2 21 25.23 5 449.4515

835.9 72333 140 24 38.84 NEPTAAAIAYGLDKR 199 15 7.3 2 795.4179

835.9 72333 140 24 38.84 PAYFNDAQR 172 9 7.3 2 541.2594

sp|P27348|1433T_HUMAN 401.68 27764 55 11 35.51 NLLSVAYK 41 8 67.31 2 454.2619

14-3-3 protein theta 401.68 27764 55 11 35.51 VISSIEQK 60 8 43.63 2 452.2573

401.68 27764 55 11 35.51 DSTLIMQLLR 212 10 121.83 2 595.334

401.68 27764 55 11 35.51 AVTEQGAELSNEER 27 14 30.85 3 511.5789

401.68 27764 55 11 35.51 Carbamidomethyl+C(3) SICTTVLELLDK 91 12 118.5 2 696.3763

401.68 27764 55 11 35.51 YLIANATNPESK 103 12 28.69 2 660.8429

401.68 27764 55 11 35.51 Carbamidomethyl+C(7) YLAEVACGDDR 127 11 26.82 2 634.7862

401.68 27764 55 11 35.51 VESELR 85 6 14.54 1 732.3848

401.68 27764 55 11 35.51 TELIQK 3 6 14.54 2 366.2143

401.68 27764 55 11 35.51 ISSIEQK 61 7 0.97 1 804.4408

401.68 27764 55 11 35.51 VISSIEQK 60 8 0.97 2 443.2526

sp|P63241|IF5A1_HUMAN 215.58 16832 30 6 41.56 IVEMSTSK 39 8 72.08 2 447.7317

Eukaryotic translation initiation factor 5A-1 215.58 16832 30 6 41.56 NGFVVLK 27 7 88.99 2 388.734

215.58 16832 30 6 41.56 Carbamidomethyl+C(5) YEDICPSTHNMDVPNIK 68 17 29.17 3 678.3083

215.58 16832 30 6 41.56 NDFQLIGIQDGYLSLLQDSGEVR 86 23 38.1 3 860.772

215.58 16832 30 6 41.56 HGHAK 50 5 14.22 1 549.2843

215.58 16832 30 6 41.56 NDFQLIGIQDGYLSLLQDSGEVREDLR 86 27 13.11 5 619.516

327.96 21056 43 8 41.71 LYEQLSGK 179 8 60.87 2 469.2505

327.96 21056 43 8 41.71 VLTPTQVK 39 8 57.86 2 443.2716

327.96 21056 43 8 41.71 LYTLVLTDPDAPSR 62 14 82.12 2 780.9162

327.96 21056 43 8 41.71 YVWLVYEQDRPLK 119 13 35.67 3 570.3034

327.96 21056 43 8 41.71 NRPTSISWDGLDSGK 47 15 94.45 3 544.9371

327.96 21056 43 8 41.71 GNDISSGTVLSDYVGSGPPK 93 20 35.99 2 975.48

327.96 21056 43 8 41.71 WLVYEQDRPLK 121 11 2.58 2 723.8861

327.96 21056 43 8 41.71 VLTPTQVK 39 8 0.97 2 434.2673

sp|Q99497|PARK7_HUMAN Protein DJ-1 234.8 19891 40 10 48.15 Carbamidomethyl+C(14) VTVAGLAGKDPVQCSR 32 16 69.34 3 553.2927

234.8 19891 40 10 48.15 Carbamidomethyl+C(5) DVVICPDASLEDAKK 48 15 109.22 3 553.9438

234.8 19891 40 10 48.15 Carbamidomethyl+C(7) GLIAAICAGPTALLAHEIGFGSK 99 23 50.21 3 756.4113

234.8 19891 40 10 48.15 EGPYDVVVLPGGNLGAQNLSESAAVK 63 26 13.43 3 862.1107

234.8 19891 40 10 48.15 ALVILAK 5 7 43.31 2 364.2545

234.8 19891 40 10 48.15 EILK 89 4 13.9 1 502.3201

234.8 19891 40 10 48.15 Carbamidomethyl+C(5) DVVICPDASLEDAKK 48 15 39.9 2 830.4107

234.8 19891 40 10 48.15 Carbamidomethyl+C(3) VICPDASLEDAKK 50 13 7.45 2 723.3718

234.8 19891 40 10 48.15 Carbamidomethyl+C(2) ICPDASLEDAKK 51 12 3.22 2 673.8395

234.8 19891 40 10 48.15 Carbamidomethyl+C(12) VAGLAGKDPVQCSR 34 14 15.46 2 729.3818

sp|P31946|1433B_HUMAN 270.79 28082 35 6 20.73 NLLSVAYK 43 8 67.31 2 454.2619

14-3-3 protein beta/alpha 270.79 28082 35 6 20.73 VISSIEQK 62 8 43.63 2 452.2573

270.79 28082 35 6 20.73 DSTLIMQLLR 214 10 121.83 2 595.334

270.79 28082 35 6 20.73 AVTEQGHELSNEER 29 14 29.34 3 533.5824

270.79 28082 35 6 20.73 YLSEVASGDNK 129 11 29.83 2 591.7897

270.79 28082 35 6 20.73 TEQGHELSNEER 31 12 2.9 2 714.8261

sp|P25398|RS12_HUMAN 182.07 14515 27 5 26.52 Carbamidomethyl+C(6) LVEALCAEHQINLIK 63 15 91.99 3 584.3182

40S ribosomal protein S12 182.07 14515 27 5 26.52 Carbamidomethyl+C(8) LGEWVGLCK 84 9 66.38 2 531.2776

182.07 14515 27 5 26.52 Carbamidomethyl+C(5);

Carbamidomethyl+C(7) KVVGCSCVVVK 101 11 40.25 2 617.8379

182.07 14515 27 5 26.52 Oxidation+M(11) AEEGIAAGGVMDVNTALQEVLK 1 22 23.8 3 744.3734

182.07 14515 27 5 26.52 Carbamidomethyl+C(4) EALCAEHQINLIK 65 13 28.41 2 769.9054

sp|P27797|CALR_HUMAN Calreticulin 378.3 48141 63 9 29.26 IKDPDASKPEDWDER 207 15 28.41 3 600.9501

378.3 48141 63 9 29.26 EQFLDGDGWTSR 24 12 87.59 2 705.821

378.3 48141 63 9 29.26 Carbamidomethyl+C(7) HEQNIDCGGGYVK 98 13 72.35 3 492.8916

378.3 48141 63 9 29.26 FYALSASFEPFSNK 73 14 116.43 2 804.3947

378.3 48141 63 9 29.26 FVLSSGK 48 7 57.53 2 369.2109

378.3 48141 63 9 29.26 VHVIFNYK 143 8 35.85 2 510.2872

378.3 48141 63 9 29.26 IDDPTDSKPEDWDKPEHIPDPDAK 224 24 94.14 5 552.8493

378.3 48141 63 9 29.26 KPEDWDEEMDGEWEPPVIQNPEYK 248 24 45.89 3 987.4389

378.3 48141 63 9 29.26 WIESK 36 5 14.22 2 331.6796

sp|P67936|TPM4_HUMAN 255.88 28521 41 8 30.24 IQLVEEELDR 55 10 121.83 2 622.3266

Tropomyosin alpha-4 chain 255.88 28521 41 8 30.24 LVILEGELER 132 10 77.91 2 585.8386

255.88 28521 41 8 30.24 Carbamidomethyl+C(19) EENVGLHQTLDQTLNELNCI 228 20 16.15 3 780.706

255.88 28521 41 8 30.24 AEGDVAALNR 44 10 31.27 2 508.2629

255.88 28521 41 8 30.24 AGLNSLEAVK 1 10 31.27 2 501.2775

255.88 28521 41 8 30.24 KLVILEGELER 131 11 44.84 3 433.5895

255.88 28521 41 8 30.24 RIQLVEEELDR 54 11 41.83 2 700.3757

255.88 28521 41 8 30.24 TIDDLEEKLAQAK 215 13 22.99 3 491.9351

sp|P30101|PDIA3_HUMAN 650.45 56782 94 19 38.61 LAPEYEAAATR 62 11 76.48 2 596.3053

Protein disulfide-isomerase A3 650.45 56782 94 19 38.61 YGVSGYPTLK 94 10 77.91 2 542.7865

650.45 56782 94 19 38.61 LNFAVASR 296 8 57.86 2 439.2456

650.45 56782 94 19 38.61 EATNPPVIQEEKPK 482 14 58.99 3 527.278

650.45 56782 94 19 38.61 SEPIPESNDGPVK 366 13 47.46 2 684.8404

650.45 56782 94 19 38.61 TFSHELSDFGLESTAGEIPVVAIR 305 24 75.46 3 859.1068

650.45 56782 94 19 38.61 TADGIVSHLK 119 10 55.49 2 520.7878

650.45 56782 94 19 38.61 GFPTIYFSPANK 448 12 85.28 2 671.3503

650.45 56782 94 19 38.61 FLQDYFDGNLK 351 11 26.82 2 680.3346

650.45 56782 94 19 38.61 Carbamidomethyl+C(3);

Carbamidomethyl+C(10) VDCTANTNTCNK 82 12 58.9 2 699.298

650.45 56782 94 19 38.61 GIVPLAK 75 7 43.31 2 349.2323

650.45 56782 94 19 38.61 FISDKDASIVGFFDDSFSEAHSEFLK 147 26 52.09 4 735.3438

650.45 56782 94 19 38.61 DLIQGK 252 6 14.54 1 673.3813

650.45 56782 94 19 38.61 FISDK 147 5 14.22 1 609.3227

650.45 56782 94 19 38.61 DLLIAYYDVDYEK 258 13 46.46 3 540.6089

650.45 56782 94 19 38.61 LAAASDVLELTDDNFESR 20 18 37.86 2 983.4866

650.45 56782 94 19 38.61 PPVIQEEKPK 486 10 7.58 2 582.8343

650.45 56782 94 19 38.61 TNPPVIQEEKPK 484 12 2.9 3 460.5861

650.45 56782 94 19 38.61 EATNPPVIQEEKPK 482 14 16.88 3 521.2773

sp|P62318|SMD3_HUMAN 124.76 13916 23 5 44.44 VAQLEQVYIR 54 10 55.49 2 609.8447

Small nuclear ribonucleoprotein Sm D3 124.76 13916 23 5 44.44 FLILPDMLK 69 9 66.38 2 545.3224

124.76 13916 23 5 44.44 Carbamidomethyl+C(12) VLHEAEGHIVTCETNTGEVYR 8 21 15.57 3 805.3836

124.76 13916 23 5 44.44 NAPMLK 78 6 31.78 1 673.3666

124.76 13916 23 5 44.44 GMGRGNIFQK 114 10 36.5 2 554.2896

sp|P38159|HNRPG_HUMAN 382.05 42331 48 11 23.02 VEQATKPSFESGR 80 13 67.7 3 479.2394

Heterogeneous nuclear ribonucleoprotein G 382.05 42331 48 11 23.02 LFIGGLNTETNEK 9 13 27.76 2 718.3785

382.05 42331 48 11 23.02 GPPPSYGGSSR 298 11 87.69 2 531.2566

382.05 42331 48 11 23.02 GFAFVTFESPADAK 49 14 87.14 2 743.8659

382.05 42331 48 11 23.02 ALEAVFGK 22 8 57.86 2 417.7381

382.05 42331 48 11 23.02 DRDYSDHPSGGSYR 268 14 41.8 3 537.8978

382.05 42331 48 11 23.02 DYTYR 239 5 14.22 2 359.1668

382.05 42331 48 11 23.02 GGSGGTRGPPSR 113 12 24.71 2 543.2735

382.05 42331 48 11 23.02 Oxidation+M(20) GFAFVTFESPADAKDAARDMNGK 49 23 14.58 3 821.0532

382.05 42331 48 11 23.02 QATKPSFESGR 82 11 7.77 2 604.308

382.05 42331 48 11 23.02 YSDHPSGGSYR 271 11 2.9 2 613.2599

sp|P84103|SRSF3_HUMAN 107.66 19329 16 4 12.8 AFGYYGPLR 28 9 64.62 2 522.2696

Serine/arginine-rich splicing factor 3 107.66 19329 16 4 12.8 VYVGNLGNNGNK 11 12 31.91 2 624.8218

107.66 19329 16 4 12.8 VELSNGEKR 77 9 40.95 2 556.2532

107.66 19329 16 4 12.8 GYYGPLR 30 7 1.29 1 825.4251

sp|P07910|HNRPC_HUMAN 247.14 33670 41 9 27.45 VFIGNLNTLVVK 17 12 116.03 2 658.8973

Heterogeneous nuclear ribonucleoproteins C1/C2 247.14 33670 41 9 27.45 GFAFVQYVNER 50 11 82.92 2 665.3332

247.14 33670 41 9 27.45 GDDLQAIKK 189 9 49.15 2 494.2723

247.14 33670 41 9 27.45 MIAGQVLDINLAAEPK 73 16 25.8 2 841.9629

247.14 33670 41 9 27.45 DYYDR 130 5 14.22 2 366.1533

247.14 33670 41 9 27.45 ELTQIK 198 6 14.54 2 366.2162

247.14 33670 41 9 27.45 KSDVEAIFSK 29 10 29.51 2 562.3035

247.14 33670 41 9 27.45 LKGDDLQAIK 187 10 58.5 2 550.8189

247.14 33670 41 9 27.45 VDSLLENLEKIEK 206 13 37.46 2 765.4239

sp|Q8NBS9|TXND5_HUMAN 206.88 47628 32 8 17.36 ALAPTWEQLALGLEHSETVK 221 20 64.06 3 731.7169

Thioredoxin domain-containing protein 5 206.88 47628 32 8 17.36 TLAPTWEELSK 354 11 38.58 2 637.835

206.88 47628 32 8 17.36 Carbamidomethyl+C(6) IAEVDCTAER 375 10 39.51 2 582.2729

206.88 47628 32 8 17.36 FVLSQAKDEL 422 10 29.51 2 575.3133

206.88 47628 32 8 17.36 EFPGLAGVK 366 9 40.95 2 459.2569

206.88 47628 32 8 17.36 VDQYK 273 5 14.22 1 652.33

206.88 47628 32 8 17.36 LFKPGQEAVK 140 10 36.5 3 372.8801

206.88 47628 32 8 17.36 ALAPTWEQLALGLEHSETVK 221 20 34.43 3 758.3812

sp|P60660|MYL6_HUMAN 197.24 16930 33 6 37.75 HVLVTLGEK 110 9 49.15 2 498.2968

Myosin light polypeptide 6 197.24 16930 33 6 37.75 ALGQNPTNAEVLK 37 13 35.67 2 677.8722

197.24 16930 33 6 37.75 VLDFEHFLPMLQTVAK 63 16 27.37 3 630.0022

197.24 16930 33 6 37.75 EAFQLFDR 13 8 57.86 2 513.256

197.24 16930 33 6 37.75 Carbamidomethyl+C(6) ILYSQCGDVMR 26 11 33.81 2 671.323

197.24 16930 33 6 37.75 PMLQTVAK 71 8 3.55 2 444.2543

sp|P19338|NUCL_HUMAN Nucleolin 430.08 76614 69 14 19.3 VFGNEIK 370 7 40.3 2 403.7241

430.08 76614 69 14 19.3 LELQGPR 554 7 43.31 2 406.7334

430.08 76614 69 14 19.3 EVFEDAAEIR 410 10 39.51 2 589.7902

430.08 76614 69 14 19.3 ALELTGLK 362 8 60.87 2 422.7591

430.08 76614 69 14 19.3 NDLAVVDVR 333 9 64.62 2 500.7747

430.08 76614 69 14 19.3 SISLYYTGEK 457 10 55.49 2 580.7992

430.08 76614 69 14 19.3 GIAYIEFK 429 8 38.86 2 470.7599

430.08 76614 69 14 19.3 GLSEDTTEETLK 577 12 37.93 2 661.8249

430.08 76614 69 14 19.3 TLVLSNLSYSATEETLQEVFEK 486 22 33.91 3 834.4314

430.08 76614 69 14 19.3 FGYVDFESAEDLEK 348 14 21.55 2 824.8781

430.08 76614 69 14 19.3 GFGFVDFNSEEDAK 610 14 68.44 2 781.3561

430.08 76614 69 14 19.3 ALVATPGK 116 8 67.31 2 378.7304

430.08 76614 69 14 19.3 KVVVSPTK 62 8 38.86 2 429.281

430.08 76614 69 14 19.3 SLYYTGEK 459 8 1.61 1 960.4665

sp|P52272|HNRPM_HUMAN 414.86 77515 70 18 27.53 INEILSNALK 371 10 43.73 2 557.826

Heterogeneous nuclear ribonucleoprotein M 414.86 77515 70 18 27.53 AFITNIPFDVK 72 11 55.26 2 632.8496

414.86 77515 70 18 27.53 MGANSLER 570 8 35.85 2 439.2131

414.86 77515 70 18 27.53 LGSTVFVANLDYK 201 13 52.48 2 713.8827

414.86 77515 70 18 27.53 GNFGGSFAGSFGGAGGHAPGVAR 627 23 43.9 3 678.9883

414.86 77515 70 18 27.53 MAAPIDR 496 7 43.31 2 387.2016

414.86 77515 70 18 27.53 QGGGGGGGSVPGIER 388 15 28.41 2 642.8199

414.86 77515 70 18 27.53 MGPGIDR 403 7 43.31 2 373.1891

414.86 77515 70 18 27.53 MGPAMGPALGAGIER 591 15 20.33 3 476.5822

414.86 77515 70 18 27.53 Carbamidomethyl+C(2) GCGVVK 692 6 14.54 1 619.3179

414.86 77515 70 18 27.53 ALPK 281 4 13.9 1 428.2857

414.86 77515 70 18 27.53 MGLAMGGGGGASFDR 606 15 20.33 3 461.8756

414.86 77515 70 18 27.53 AAGVEAAAEVAATEIK 1 16 25.8 2 750.891

414.86 77515 70 18 27.53 MGPLGLDHMASSIER 456 15 32.63 2 807.3979

414.86 77515 70 18 27.53 ADILEDKDGK 232 10 29.51 2 552.2848

414.86 77515 70 18 27.53 MGPLGLDHMASSIERMGQTMER 456 22 23.8 3 816.3876

414.86 77515 70 18 27.53 Carbamidomethyl+C(2) GCAVVEFKMEESMK 112 14 36.29 2 822.8818

414.86 77515 70 18 27.53 MGANSLERMGLER 570 13 27.76 3 488.5704

sp|Q9BRA2|TXD17_HUMAN 112.85 13940 12 4 26.83 VTAVPTLLK 89 9 40.95 2 471.3038

Thioredoxin domain-containing protein 17 112.85 13940 12 4 26.83 TIFAYFTGSK 25 10 44.28 2 567.7956

112.85 13940 12 4 26.83 Carbamidomethyl+C(3);

Carbamidomethyl+C(6) SWCPDCVQAEPVVR 40 14 21.55 2 851.8875

112.85 13940 12 4 26.83 AVPTLLK 91 7 9.49 1 741.4801

sp|P62263|RS14_HUMAN 173.74 16272 21 4 23.84 TPGPGAQSALR 106 11 76.48 2 527.7841

40S ribosomal protein S14 173.74 16272 21 4 23.84 IEDVTPIPSDSTR 128 13 42.57 2 715.3632

173.74 16272 21 4 23.84 ELGITALHIK 86 10 55.49 2 547.8337

173.74 16272 21 4 23.84 Carbamidomethyl+C(1) CKELGITALHIK 84 12 48.35 3 461.5955

sp|Q6S8J3|POTEE_HUMAN POTE 538.01 121363 57 9 8.74 SYELPDGQVITIGNER 938 16 155.88 2 895.9475

ankyrin domain family member E 538.01 121363 57 9 8.74 QEYDESGPSIVHR 1059 13 130.58 3 506.2341

538.01 121363 57 9 8.74 IWHHTFYNELR 784 11 26.82 3 505.9183

538.01 121363 57 9 8.74 Carbamidomethyl+C(2) LCYVALDFEQEMATAASSSSLEK 915 23 70.88 3 850.7329

538.01 121363 57 9 8.74 QEYDESGPSIVHRK 1059 14 21.55 3 548.9365

538.01 121363 57 9 8.74 DVLHENSTLR 642 10 29.51 2 592.3069

538.01 121363 57 9 8.74 GYRFTTMAER 896 10 36.5 3 411.2018

538.01 121363 57 9 8.74 YHVRGEDLDK 132 10 29.51 2 616.305

538.01 121363 57 9 8.74 QEYDESGPSIVHR 1059 13 2.58 3 500.2315

sp|P49458|SRP09_HUMAN 102.83 10111 13 3 31.4 Carbamidomethyl+C(7) VTDDLVCLVYK 41 11 55.26 2 662.8439

Signal recognition particle 9 kDa protein 102.83 10111 13 3 31.4 LMVAKEAR 71 8 43.63 2 459.2603

102.83 10111 13 3 31.4 LYLADPMK 16 8 43.63 2 475.7557

sp|A5A3E0|POTEF_HUMAN POTE 469.59 121445 39 5 5.12 SYELPDGQVITIGNER 938 16 155.88 2 895.9475

ankyrin domain family member F 469.59 121445 39 5 5.12 QEYDESGPSIVHR 1059 13 130.58 3 506.2341

469.59 121445 39 5 5.12 IWHHTFYNELR 784 11 26.82 3 505.9183

469.59 121445 39 5 5.12 QEYDESGPSIVHRK 1059 14 21.55 3 548.9365

469.59 121445 39 5 5.12 VVEVDSMPAASSVK 1 14 21.55 3 473.5776

sp|Q07021|C1QBP_HUMAN 124.92 31362 27 6 23.4 AFVDFLSDEIKEER 80 14 82.12 3 566.6141

Complement component 1 Q 124.92 31362 27 6 23.4 Carbamidomethyl+C(6) ALVLDCHYPEDEVGQEDEAESDIFSIR 180 27 23.11 3 1046.1507

subcomponent-binding protein 124.92 31362 27 6 23.4 VEEQEPELTSTPNFVVEVIK 154 20 28.03 3 763.064

124.92 31362 27 6 23.4 LPLLR 1 5 14.22 1 611.4245

124.92 31362 27 6 23.4 VDFLSDEIKEER 82 12 16.88 2 740.375

124.92 31362 27 6 23.4 DFLSDEIKEER 83 11 7.58 2 690.8415

sp|P26368|U2AF2_HUMAN 136.35 53501 26 8 13.26 Carbamidomethyl+C(16) SIEIPRPVDGVEVPGCGK 413 18 40.94 3 636.9942

Splicing factor U2AF 65 kDa subunit 136.35 53501 26 8 13.26 ELLTSFGPLK 276 10 46.74 2 552.8168

136.35 53501 26 8 13.26 NFAFLEFR 195 8 35.85 2 522.2686

136.35 53501 26 8 13.26 LGGLTQAPGNPVLAVQINQDK 174 21 15.57 3 711.7298

136.35 53501 26 8 13.26 SKPLTR 61 6 14.54 1 701.4239

136.35 53501 26 8 13.26 Oxidation+M(2) AMQGLTGRK 444 9 33.16 2 489.2626

136.35 53501 26 8 13.26 Carbamidomethyl+C(12) PRPVDGVEVPGCGK 417 14 7.28 2 733.8735

136.35 53501 26 8 13.26 ELLTSFGPLK 276 10 1.61 2 543.8163

sp|Q15056|IF4H_HUMAN 117.92 27385 16 3 11.69 EALTYDGALLGDR 96 13 47.46 2 697.3545

Eukaryotic translation initiation factor 4H 117.92 27385 16 3 11.69 VDIAEGR 112 7 54.52 2 380.2017

117.92 27385 16 3 11.69 GGSRPGDRR 166 9 40.95 2 479.2521

sp|P62851|RS25_HUMAN 115.91 13742 16 6 23.2 LITPAVVSER 66 10 65.73 2 542.824

40S ribosomal protein S25 115.91 13742 16 6 23.2 AALQELLSK 85 9 49.15 2 486.7906

115.91 13742 16 6 23.2 ATYDK 52 5 28.44 1 597.2873

115.91 13742 16 6 23.2 DPVNK 20 5 14.22 1 572.3051

115.91 13742 16 6 23.2 TPAVVSER 68 8 8.84 2 429.7369

115.91 13742 16 6 23.2 LQELLSK 87 7 1.29 2 415.7507

sp|Q01105|SET_HUMAN Protein SET 138.06 33488 17 4 12.76 VEVTEFEDIK 122 10 108.4 2 604.8072

138.06 33488 17 4 12.76 IDFYFDENPYFENK 136 14 29.34 2 920.9157

138.06 33488 17 4 12.76 EFHLNESGDPSSK 154 13 22.99 3 482.8854

138.06 33488 17 4 12.76 TEFEDIK 125 7 1.61 2 441.219

sp|O14979|HNRDL_HUMAN 150.46 46437 32 7 13.1 GFGFVLFK 189 8 67.31 2 457.7576

Heterogeneous nuclear ribonucleoprotein D-like 150.46 46437 32 7 13.1 MFIGGLSWDTSK 149 12 24.71 2 671.331

150.46 46437 32 7 13.1 VFVGGLSPDTSEEQIK 234 16 19.27 3 569.2948

150.46 46437 32 7 13.1 Carbamidomethyl+C(7) FGEVVDCTIK 170 10 95.15 2 584.2879

150.46 46437 32 7 13.1 LDGK 212 4 13.9 1 432.2454

150.46 46437 32 7 13.1 VLELK 204 5 28.44 1 601.3896

150.46 46437 32 7 13.1 Oxidation+M(1) MFIGGLSWDTSK 149 12 39.6 3 453.2194

sp|Q15417|CNN3_HUMAN Calponin-3 129.88 36413 18 5 15.81 Carbamidomethyl+C(6) DGIILCELINK 53 11 29.83 2 644.3567

129.88 36413 18 5 15.81 YDHQAEEDLR 23 10 58.5 3 425.8569

129.88 36413 18 5 15.81 GMSVYGLGR 256 9 33.16 2 470.2464

129.88 36413 18 5 15.81 GPSYGLSAEVK 6 11 38.82 2 554.2854

129.88 36413 18 5 15.81 GFHTTIDIGVK 132 11 26.82 2 594.3219

sp|P24534|EF1B_HUMAN 105.97 24763 16 4 15.11 SPAGLQVLNDYLADK 7 15 51.11 2 802.4152

Elongation factor 1-beta 105.97 24763 16 4 15.11 LVPVGYGIK 176 9 48.73 2 473.2916

105.97 24763 16 4 15.11 EERLAQYESK 119 10 29.51 2 626.8142

105.97 24763 16 4 15.11 PVGYGIK 178 7 9.49 2 367.2128

sp|Q16629|SRSF7_HUMAN 103.34 27366 24 8 20.17 VYVGNLGTGAGK 12 12 39.6 2 568.3102

Serine/arginine-rich splicing factor 7 103.34 27366 24 8 20.17 AFSYYGPLR 29 9 40.95 2 537.277

103.34 27366 24 8 20.17 SGSIK 180 5 14.22 1 491.278

103.34 27366 24 8 20.17 GLDGK 65 5 14.22 1 489.2625

103.34 27366 24 8 20.17 GELER 24 5 28.44 1 603.3074

103.34 27366 24 8 20.17 TVWIAR 38 6 14.54 2 373.2244

103.34 27366 24 8 20.17 Carbamidomethyl+C(8) GLDGKVICGSR 65 11 38.82 2 581.311

103.34 27366 24 8 20.17 VYVGNLGTGAGKGELER 12 17 18.34 3 573.9757

sp|P14866|HNRPL_HUMAN 114.36 64132 20 6 8.83 Carbamidomethyl+C(6) VFNVFCLYGNVEK 398 13 35.67 2 794.8976

Heterogeneous nuclear ribonucleoprotein L 114.36 64132 20 6 8.83 ISRPGDSDDSR 178 11 41.83 3 402.1867

114.36 64132 20 6 8.83 LNVFK 272 5 14.22 1 620.3744

114.36 64132 20 6 8.83 IVIFR 223 5 28.44 2 324.2125

114.36 64132 20 6 8.83 SKPGAAMVEMADGYAVDR 416 18 36.44 3 623.296

114.36 64132 20 6 8.83 SRPGDSDDSR 179 10 8.38 2 546.2429

sp|Q6NXT2|H3C_HUMAN Histone H3.3C 110.95 15213 17 4 20 STELLIR 56 7 54.52 2 416.2485

110.95 15213 17 4 20 YRPGTVALR 40 9 49.15 2 516.8008

110.95 15213 17 4 20 DIQLAR 122 6 42.99 2 358.2078

110.95 15213 17 4 20 LPFQR 64 5 14.22 1 660.3815

sp|P06748|NPM_HUMAN Nucleophosmin 338.8 32575 60 15 39.12 VDNDENEHQLSLR 32 13 99.12 3 523.5756

338.8 32575 60 15 39.12 GPSSVEDIK 239 9 72.4 2 466.2373

338.8 32575 60 15 39.12 TVSLGAGAKDELHIVEAEAMNYEGSPIK 45 28 17.03 4 733.1154

338.8 32575 60 15 39.12 MSVQPTVSLGGFEITPPVVLR 80 21 118.05 3 743.073

338.8 32575 60 15 39.12 DELHIVEAEAMNYEGSPIK 54 19 45.91 4 537.0078

338.8 32575 60 15 39.12 FINYVK 267 6 42.99 2 392.2196

338.8 32575 60 15 39.12 LLSISGK 134 7 43.31 2 359.2265

338.8 32575 60 15 39.12 VTLATLK 73 7 57.53 2 373.2381

338.8 32575 60 15 39.12 MTDQEAIQDLWQWR 277 14 27 2 910.4294

338.8 32575 60 15 39.12 NAQKSNQNGK 202 10 43.73 2 544.7786

338.8 32575 60 15 39.12 DNDENEHQLSLR 33 12 2.58 2 735.3396

338.8 32575 60 15 39.12 NDENEHQLSLR 34 11 2.58 2 677.8258

338.8 32575 60 15 39.12 SSVEDIK 241 7 1.29 1 777.395

338.8 32575 60 15 39.12 VDNDENEHQLSLR 32 13 2.58 3 517.9078

338.8 32575 60 15 39.12 GPSSVEDIK 239 9 9.49 2 457.2365

sp|P07602|SAP_HUMAN 127.11 58112 18 5 10.31 Carbamidomethyl+C(9) LGPGMADICK 232 10 36.5 2 531.2627

Proactivatorpolypeptide 127.11 58112 18 5 10.31 EIVDSYLPVILDIIK 107 15 28.99 2 865.5057

127.11 58112 18 5 10.31 LVGYLDR 414 7 43.31 2 418.2329

127.11 58112 18 5 10.31 Carbamidomethyl+C(4) IGACPSAHKPLLGTEK 478 16 38.18 2 839.9426

127.11 58112 18 5 10.31 HEVPAK 304 6 14.54 2 340.6879

sp|P15121|ALDR_HUMAN Aldose reductase 129.39 35853 27 8 26.9 TTAQVLIR 243 8 53.08 2 451.2748

129.39 35853 27 8 26.9 REELFIVSK 69 9 33.16 2 560.8236

129.39 35853 27 8 26.9 GIVVTAYSPLGSPDRPWAKPEDPSLLEDPR 203 30 27.72 4 816.4152

129.39 35853 27 8 26.9 Carbamidomethyl+C(2);

Carbamidomethyl+C(7) VCALLSCTSHKDYPFHEEF 297 19 25.98 3 780.6861

129.39 35853 27 8 26.9 TLSDLK 95 6 14.54 1 676.385

129.39 35853 27 8 26.9 Carbamidomethyl+C(2);

Carbamidomethyl+C(7) VCALLSCTSHK 297 11 38.58 3 425.8728

129.39 35853 27 8 26.9 NLVVIPK 256 7 43.31 2 391.7586

129.39 35853 27 8 26.9 IAENFK 269 6 14.54 2 361.1963

sp|P43243|MATR3_HUMAN Matrin-3 226.07 94623 43 11 14.52 ITPENLPQILLQLK 132 14 42.01 2 810.4942

226.07 94623 43 11 14.52 IGPYQPNVPVGIDYVIPK 780 18 24.93 2 985.0527

226.07 94623 43 11 14.52 LAEPYGK 515 7 43.31 2 389.2144

226.07 94623 43 11 14.52 GPSLNPVLDYDHGSR 192 15 28.99 3 542.9382

226.07 94623 43 11 14.52 SFQQSSLSR 3 9 33.16 2 520.263

226.07 94623 43 11 14.52 GIDLLK 582 6 46 1 658.4088

226.07 94623 43 11 14.52 VVHIMDFQR 398 9 72.4 3 382.1965

226.07 94623 43 11 14.52 VIHLSNLPHSGYSDSAVLK 496 19 24.58 4 510.0182

226.07 94623 43 11 14.52 GPLPLSSQHR 92 10 29.51 2 546.2938

226.07 94623 43 11 14.52 SQAFIEMETR 532 10 43.73 3 404.5299

226.07 94623 43 11 14.52 NYILMR 524 6 14.54 2 405.2167

a) Protein name; b) Protein score by PLGS software; c) Protein average mass; d) Number of matched products; e) Number of matched peptides; f) Percentage of protein sequence coverage; g) Peptide sequence; h) Position of the peptide starting aminoacid; e) Peptide length; m) Peptide score by PLGS software; n) Precursor charge state; o) Precursor mass.
