# Supplementary material for: LMNA Knock-Down Affects Differentiation and Progression of Human Neuroblastoma Cells
Source: PLoS One. 2012 Sep 26;7(9):e45513. doi: 10.1371/journal.pone.0045513 (PMC3458895; doi:10.1371/journal.pone.0045513)
Supplement: Table S7 — Protein specifications of the network shown in Figure 4 . (DOC) [file pone.0045513.s009.doc]

**Table S7**

Protein specifications of the network shown in Figure 4.

| **Gene Symbol** | **Entrez Gene Name** | **GenPept/UniProt/** |
| --- | --- | --- |
|  |  | **Swiss-Prot Accession** |
| ACTN4 | actinin, alpha 4 | O43707 |
| ANXA5 | annexin A5 | P08758 |
| C1QBP | complement component 1, q subcomponent binding protein | Q07021 |
| CFL1 | cofilin 1 (non-muscle) | P23528 |
| EEF1B2 | eukaryotic translation elongation factor 1 beta 2 | P24534 |
| EIF5A | eukaryotic translation initiation factor 5A | P63241 |
| H3F3C | H3 histone, family 3C | Q6NXT2 |
| HNRNPA2B1 | heterogeneous nuclear ribonucleoprotein A2/B1 | P22626 |
| HNRNPL | heterogeneous nuclear ribonucleoprotein L | P14866 |
| NCL | nucleolin | P19338 |
| NPM1 | nucleophosmin (nucleolar phosphoprotein B23, numatrin) | P06748 |
| (includes EG:4869) |  |  |
| PEBP1 | phosphatidylethanolamine binding protein 1 | P30086 |
| PPIA | peptidylprolyl isomerase A (cyclophilin A) | P62937 |
| (includes EG:5478) |  |  |
| RPLP0 | ribosomal protein, large, P0 | P05388 |
| (includes EG:6175) |  |  |
| RPS14 | ribosomal protein S14 | P62263 |
| RPS18 | ribosomal protein S18 | P62269 |
| RPS4X | ribosomal protein S4, X-linked | P62701 |
| RPS9 | ribosomal protein S9 | P46781 |
| SET | SET nuclear oncogene | Q01105 |
| SRSF3 | serine/arginine-rich splicing factor 3 | P84103 |
| TPI1 | triosephosphate isomerase 1 | P60174 |
| TXNDC17 | thioredoxin domain containing 17 | Q9BRA2 |
| YWHAB | tyrosine 3-monooxygenase/tryptophan 5-monooxygenase | P31946 |
|  | activation protein, beta polypeptide |  |
| YWHAE | tyrosine 3-monooxygenase/tryptophan 5-monooxygenase | P62258 |
|  | activation protein, epsilon polypeptide |  |
| YWHAG | tyrosine 3-monooxygenase/tryptophan 5-monooxygenase | P61981 |
|  | activation protein, gamma polypeptide |  |
| YWHAQ | tyrosine 3-monooxygenase/tryptophan 5-monooxygenase | P27348 |
| (includes EG:10971) | activation protein, theta polypeptide |  |
| YWHAZ | tyrosine 3-monooxygenase/tryptophan 5-monooxygenase | P63104 |
|  | activation protein, zeta polypeptide |  |
