# Supplementary material for: LMNA Knock-Down Affects Differentiation and Progression of Human Neuroblastoma Cells
Source: PLoS One. 2012 Sep 26;7(9):e45513. doi: 10.1371/journal.pone.0045513 (PMC3458895; doi:10.1371/journal.pone.0045513)
Supplement: Table S8 — Primers sequences for SYBR green assays. (DOC) [file pone.0045513.s010.doc]

**Table S8**

Primers sequences for SYBR green assays

| **Gene Symbol** | **Unigene** | **F primer^a^** | **R primer^b^** |
| --- | --- | --- | --- |
| *LMNA* | Hs.594444 | AGCAAAGTGCGTGAGGAGTT | AGGTCACCCTCCTTCTTGGT |
| *TH* | Hs.435609 | ACGCCAAGGACAAGCTCA | AGCGTGTACGGGTCGAACT |
| *GAPDH* | Hs.544577 | AGCCACATCGCTCAGACA | GCCCAATACGACCAAATCC |
| *ENO2* | Hs.511915 | ACTTTGTCAGGGACTATCCTGTG | TCCCTACATTGGCTGTGAACT |
| *MAPT* | Hs.101174 | ACCACAGCCACCTTCTCCT | CAGCCATCCTGGTTCAAAGT |
| *TBP* | Hs.590872 | GCTGGCCCATAGTGATCTTT | CTTCACACGCCAAGAAACAGT |
| *PPIA* | Hs.356331) | ATGCTGGACCCAACACAAAT | TCTTTCACTTTGCCAAACACC |

a) F, forward primer. b) R, reverse primer.
