# Supplementary material for: LMNA Knock-Down Affects Differentiation and Progression of Human Neuroblastoma Cells
Source: PLoS One. 2012 Sep 26;7(9):e45513. doi: 10.1371/journal.pone.0045513 (PMC3458895; doi:10.1371/journal.pone.0045513)
Supplement: Table S9 — TaqMan assays validated for the indicated human genes. (DOC) [file pone.0045513.s011.doc]

**Table S9**

TaqMan assays validated for the indicated human genes.
