# Supplementary material for: LMNA Knock-Down Affects Differentiation and Progression of Human Neuroblastoma Cells
Source: PLoS One. 2012 Sep 26;7(9):e45513. doi: 10.1371/journal.pone.0045513 (PMC3458895; doi:10.1371/journal.pone.0045513)
Supplement: Materials and Methods S1 — Supporting materials and methods and supporting references. (DOC) [file pone.0045513.s012.doc]

**Supporting Materials and Methods**

Western blot analysis

Total protein was isolated from cells as previously described [1], and 30 µg was subjected to SDS–polyacrylamide gel electrophoresis. The blots were incubated with the following primary anti-human antibodies: an anti-Lamin A/C polyclonal antibody (N-18; Santa Cruz Biotechnology); an anti-dopamine-β-hydroxylase polyclonal antibody (AB1585, Chemicon); an anti-tyrosine hydroxyase polyclonal antibody (Cell Signaling); and an anti-GAPDH monoclonal antibody (6C5, Chemicon). The membranes were then incubated with a secondary antibody (anti-mouse, anti-rabbit or anti-goat IgG) conjugated to Alexa Fluor 680 (Invitrogen) or IRDye 800 (LI-COR Biosciences) and were analyzed using the Licor Odyssey Infrared Image System in the 700- or 800-nm channel.

Colony formation Assay

One thousand untreated or drug-treated cells were seeded at clonal density on a 35-mm Ø dish in complete medium. Ten days after seeding, the cells were fixed for 1 hour with a solution of 1% Coomassie Blue R250 (Bio-Rad) in ethanol. The dishes were then accurately washed with ddH_2_O and the colonies were counted. Cell survival following drug treatment is expressed as the percentage of colonies formed out of cells seeded compared to untreated controls (% cell survival). A total of three independent experiments were performed.

Neurite quantification

After a 5-day RA treatment period, the cells were viewed with a Leica DM IRB inverted phase contrast microscope at 200X original magnification. The cells were scored as differentiated if the length of the neurite extensions was at least two times the diameter of the cell body. The neurites were evaluated based on the percentage of cells bearing neuritis as determined from the photographs of the cell cultures, which facilitated tracing the individual neurites and branch points. A total of >300 cells was examined in 5 randomly chosen fields in each treated and untreated sample.

Fluorescence and immunofluorescence microscopy

Cells were seeded on coverglass supports in complete medium and were treated or not with RA for the indicated amounts of time. To study the localization of NF200, the cells were fixed with 4% (w/v) paraformaldehyde then permeabilized in PBS containing 0.1% Triton-X100. NF200 was detected using the anti-NF200 monoclonal antibody (N52; Sigma). Alexa Fluor 594 goat anti-mouse was used as a secondary antibody. To study Lamin A/C and F-actin localization, the cells were fixed in 2% paraformaldehyde in PBS containing 2% sucrose for 30 min, were permeabilized in PBS containing 0.2% Tween-20 and were blocked with 5% non-fat dry milk in PBS. Lamin A/C was detected using an anti-Lamin A/C monoclonal antibody (Jol2; Chemicon). Alexa Fluor 594 goat anti-mouse was used as a secondary antibody. After the immunostaining, the cells were incubated for 30 min with 5 μg/ml TRITC-phalloidin (Sigma) in PBS. The nuclei were stained with 1 µg/ml DAPI for 5 min in PBS. Finally, the cells were washed in PBS and briefly rinsed in ddH_2_O and glass coverslips were mounted in ProLong Gold anti-Fade Reagent (Molecular Probes). Images were acquired using a confocal laser scanning microscope (Leica Confocal Microsystem TCS SP5). The images were processed using Leica Application Suite 6000; the brightness and contrast of the acquired images were adjusted, and the figures were generated using Adobe Photoshop 7.0.

Total RNA preparation

Total RNA was isolated from each cell culture or tumor sample using NucleoSpin RNA II silica columns (Macherey-Nagel). Specifically for tumor samples, tissues were surgically excised immediately after the mice were sacrificed and were flash-frozen in liquid nitrogen then stored at -80°C until further use. The frozen tissues were homogenized with a tissue homogenizer in the RNA lysis buffer from the NucleoSpin RNA II extraction kit then extracted according to the manufacturer’s instructions. The RNA quantity was determined by absorbance at 260 nm using a NanoDrop UV-VIS spectrophotometer. The quality of each sample was checked using the Agilent BioAnalyzer 2100 (Agilent RNA 6000 Nanokit): samples with an RNA integrity number (RIN) lower than 8.0 were discarded [2].

Real‐time RT‐PCR analysis

RNA (500 ng) was retro-transcribed using the High-Capacity cDNA Reverse Transcription Kit (Applied Biosystems) according to the manufacturer’s instructions. Equal amounts of cDNA were then subjected to quantitative real-time RT-PCR analysis using an Applied Biosystems 7900HT thermal cycler, and the SensiMix SYBR Kit (Bioline) or the TaqMan Gene Expression Master Mix (Applied Biosystems). The specific primers, which were used at a concentration of 200 nM for the SYBR green assays, are listed in Table S8. The specific inventoried TaqMan assays (Applied Biosystems), which were used following the manufacturer's instructions, are listed in Table S9. Each experiment was performed using biological triplicates and technical quadruplicates. The expression data were normalized using the Ct values of the internal controls *GAPDH*, *TBP*, *18S* and *PPIA.*

Whole genome expression profiling

The gene expression profiling was performed using the Agilent one-color microarray platform (http://www.genomics.agilent.com/). The cyanine 3-CTP labeled cRNA was prepared using the Agilent Low Input Linear Amplification Kit and was hybridized to an Agilent 4x44K whole human genome oligonucleotide microarray (G4112F, 41000 unique probes) as previously described.[3] The post-hybridization image acquisition was accomplished using the Agilent Scanner G2564B. Data extraction from the 20-bit TIFF images was accomplished using the Agilent Feature Extraction version 10.1 software using the standard Agilent one-color gene expression extraction protocol (GE1_107_Sep09). For all of the subsequent analyses, the “gProcessedSignal” data column of the Feature Extraction *.txt output file, which contained the median signals corrected by spatial and multiplicative detrending, was used. Data quality filtering was performed using Agilent GeneSpring GX 11.0, discarding features with the Feature Extraction flag gIsWellAboveBG=0 in any of the samples. This is a robust statistical method approximately equivalent to discarding features with a signal-to-noise ratio a ratio between 2.0 and 3.0, wherein the signal = (median of the spot - median spot background level) and the noise is the standard deviation of the median spot background. Differential gene lists were obtained from the quality-filtered data using Microsoft Excel. A threshold of 350.0 in the absolute expression level was chosen to obtain a higher stringency and a smaller relative error of the signal. Differentially expressed genes were identified as those showing an average fold change ratio larger than 2.0 and lower than 0.5 on a linear scale. The functional annotation of the differential gene lists was performed using the DAVID web tool (<http://david.abcc.ncifcrf.gov/>) [4] and gene clustering analysis was performed using MeV 4.4 (MultiExperiment Viewer, TM4 suite, http://www.tm4.org/mev/) [5] as previously described. Subsets of differentially expressed genes associated with specific cancer features were selected based on the gene annotations in the Cancer Gene Index of the National Cancer Institute (NCI). The database is a file in XML format that can be downloaded from the following URL: <https://wiki.nci.nih.gov/display/ICR/The+Cancer+Gene+Index+Gene-Disease+and+Gene-Compound+XML+Documents>. The current Index version (April 2009) is a collection of records on approximately 7000 human genes identified as having an association with cancer, based on data mining of Medline. A gene is included in the database if the name co-occurs in a single Medline sentence with a cancer disease or compound/treatment term found in the NCI thesaurus. The gene symbols are those defined by the HUGO Gene Nomenclature Committee, and the annotations of Agilent microarray probes were updated 15 January 2010. We have compiled three gene symbol lists by extracting genes associated to the following three Boolean queries from the whole Cancer Gene Index: List1, “migration OR invasion OR invasive OR metastasis OR adhesion OR adhesive”; List2, “drug resistance OR drug resistant OR survival OR survive OR sensitive OR sensitivity”; and List3, “aggressive OR aggression OR progression OR progressive”. A gene was included in our lists if any of the terms in the corresponding query appeared in the gene annotation records. We selected subsets of genes differentially expressed in our experiments and with a match in at least one of the three lists for specific analysis.

In vitro Cell Migration and Invasion Assay

The Boyden chamber was used to evaluate the migration and invasion of neuroblastoma cells in response to the conditioned medium of NIH3T3 cells as previously described.[6] Briefly, 5×10^5^ cells were added to the upper chamber, and the lower compartment was filled with 200 μl of NIH3T3 CM or with DMEM supplemented with 0.1% bovine serum albumin (BSA) to evaluate random migration and invasion (negative control). The compartments were separated by an 8-μm pore-size polycarbonate filter (Costar Corp.) coated with Matrigel (25 µg/filter) for chemoinvasion or gelatin (5 µg/ml; Sigma) for chemotaxis. After incubation for 6 hours in a humidified 5% CO_2_ atmosphere at 37°C, the cells on the upper side of the filter were removed mechanically, and cells that had invaded or migrated to the lower surface of the filter were fixed in ethanol and stained with crystal violet. Crystal violet was solubilized from the filter after incubation with 10% (v/v) acetic acid for 1 hour. The relative absorbance was measured with a spectrophotometer (570 nm).

Zymography of Gelatinolytic Activity

Subconfluent cells were incubated for 24 hours in complete medium containing 1% FBS. Supernatants were collected and centrifuged (2,000 *g*, 10 min, 4°C) to remove cellular debris. The conditioned media (CM) of the cells were concentrated with Centricon-30 concentrators (Amicon). Each sample derived from 4x10^3^ cells was subjected to SDS-PAGE on a 10% polyacrylamide gel with 0.2% (w/v) gelatin. Gel electrophoresis was performed under non-reducing conditions without boiling. The gel was rinsed twice for 30 min in 2.5% (v/v) Triton X-100 to remove SDS and to renature the proteins and was incubated with activation buffer (50 mM Tris-HCl pH 7.6; 5 mM CaCl_2_;1 µM ZnCl_2_; 1% Triton X-100) overnight at 37°C with constant shaking. The gel was stained with 0.1% (w/v) Coomassie Brilliant Blue R-250 in 50% (v/v) methanol and 10% (v/v) acetate then destained in 10% (v/v) methanol and 10% (v/v) acetate. Enzymatic activity was detected as a white band on the resulting blue background of undigested gelatin. The presence of matrix metalloproteinase-2 (MMP2) protein in the CM was verified by western blot analysis. CM loaded in Laemmli buffer was boiled and electrophoresed under reducing conditions using 11% SDS-PAGE and the polyclonal anti-MMP2 (4022, Cell Signaling) antibody was used. Red Ponceau (Sigma) staining (0.1% in acetic acid) of the filter was used to check equal loading of the proteins. The experiments were repeated three times.

P-glycoprotein immunostaining

Cells (10^6^/sample) were washed in washing buffer (WB; PBS, 10 mM NaN_3_, EDTA 0.002%) and purified mouse anti-P-glycoprotein (4E3.16, Calbiochem) or control IgG diluted in complete medium at 12.5 μg/mL was added to the samples which were then incubated for 1 hour on ice. The cells were washed twice with WB and were incubated in WB containing goat anti-mouse RPE (Southern Biotech Associates) for 40 min. After washing twice in WB, cells were resuspended in 300 μl of PBS for flow cytometric analysis. Ten thousand events per sample were acquired using a FACScan cytofluorimeter.

Calcein/AM Retention Assay

Cells (5×10^5^/sample) were suspended in 1 mL of complete medium and were incubated with or without verapamil (5 µM, Sigma) for 30 min at 37°C. Then, cells were washed twice in PBS containing 0.2% BSA to eliminate any verapamil retained in the cells. The washed cells were suspended into the same medium and were incubated with calcein/AM (100 nM, Sigma) for 20 min at 37°C. Finally, cells were washed twice in PBS containing 0.2% BSA, cooled to 4°C and analysed by flow cytometry. Ten-thousand events per sample were acquired using a FACScan cytofluorimeter.

Proteome profiling by nLC-MS^E^

Sample preparation, data acquisition and data processing were performed as previously described.[7] Protein extracts from the different conditions in separated experiments were precipitated with a mix of ethanol, methanol and acetone (ratio 2:1:1, v/v) then dissolved in 100 mM Tris/HCl pH 7.9 containing 6 M urea and 0.1% CHAPS and sonicated. The reduction of proteins was performed by adding 100 mM DTT (1 hour at 36°C) and 200 mM iodoacetamide (1hour at room temperature). Protein samples at a final concentration of 2 µg/µl were digested with 1:20 (w/w) sequence-grade trypsin (Promega) at 36°C overnight. The reactions were stopped by adding 1 µl of 10% (v/v) TFA. A total of 0.6 µg of the protein digestion was loaded onto the nanoACQUITY UPLC System (Waters Corp.) coupled to a Q-Tof Premier mass spectrometer (Waters Corp.). Prior to loading, an enolase digestion from *Saccharomyces cerevisiae* (Waters, Corp.) was added to the sample as internal standard at final concentration of 200 fmol. Samples were injected onto a Symmetry C18 5 µm, 180 µm × 20 mm precolumn (Waters Corp.) for preconcetration and desalting and were subsequently separated using a NanoEase^TM^ BEH C18 1.7 µm, 75 µm × 25 cm nanoscale LC column (Waters Corp.) maintained at 35°C. Mobile phase A was water with 0.1 % formic acid, and mobile phase B was 0.1 % formic acid in acetonitrile. Peptides were eluted by a gradient of 3-40 % mobile phase B over 150 min at a flow rate of 250 nl/min followed by a gradient of 40-90 % mobile phase B over 5-minute and a 15-minute rinse with 90 % mobile phase B. The Q-Tof Premier mass spectrometer (Waters Corp.) was programmed to step between low (4 eV) and high (15-40 eV) collision energies using a scan time of 1.5 s over 50-1990 m/z. Samples from each condition were run at least in triplicate. The best replica for each condition was used for the subsequent analysis. The Continuum LC-MS data were processed and searched using ProteinLynx GlobalServer v2.3 (PLGS) (Waters Corporation). Protein identification was performed using the embedded ion accounting algorithm of the software and by searching a Uniprot/SWISSProt human database release 2010_11 (20259 entries), to which the sequence from the enolase of *Saccharomyces cerevisiae* was appended. The parameters for the database search were as follows: automatic tolerance for precursor ions, automatic tolerance for product ions, minimum of 3 fragment ions matched per peptide, minimum of 7 fragment ions matched per protein, minimum of 2 peptides matched per protein, 1 missed cleavage, and carbamidomethylation and oxidation of methionine as modifications. The false positive rate (FPR) of the identification algorithm is typically 3 to 4 % with a randomized database appended to the original one, which is five times the size of the original utilized database. The experimental reproducibility was sufficient because the observed values were those expected.[7,8] For both of the replicate conditions, the distribution of mass error was under 15 ppm, the retention time coefficient of variation expressed as percentage (% CV RT) was under 10% with most of the species under 5 %, and the intensity coefficient of variation expressed as percentage (% CV intensity) had a Gaussian distribution with all values under 4.5 %. The identified proteins displayed in the protein table were normalized against the P00924 entry (Enolase *Saccharomyces cerevisiae*), and peptides from Enolase *Saccharomyces cerevisiae* digestion that were the most reproducible for retention time and intensity deriving (m/z 1159.60, m/z 1286.7148, m/z, m/z 1288.70, m/z 1578.80, m/z 1755.94, m/z 1840.89, m/z 2441.10) were used to normalize the EMRTs table, the list of paired peptide exact masses and retention time. The list of normalized proteins was screened according the following criteria: proteins that were identified in at least 2 out of 3 injections of the same conditions; proteins with 0<*p*<0.05 or 0.95<*p*<1, and proteins with an expression level ratio between the conditions above 1.3 on decimal scale (Tables S5 and S6). If 0<*p*<0.05, the likelihood of down-regulation is greater than 95 %, and if 0.95<*p*<1 the likelihood of up-regulation is greater than 95 %. Setting the threshold of ratio at 1.3 on a decimal scale allowed us to consider the average relative fold-change ± 0.30 on a natural log scale. This setting is typically 2-3 times higher than the estimated error of the intensity measurements. [8,9]

Ingenuity Pathway Analysis (IPA)

The modulated genes or protein, identified by microarray and shotgun proteomic analysis were further analyzed by Ingenuity Pathway Analysis (IPA v.8.8; Ingenuity Systems, Mountain View, CA). IPA highlights protein networks or pathways starting from a continuous updated database of known protein-protein interactions based on direct (physical) and indirect (functional) associations. The algorithm yields a probability score for each possible network. Scores of 10 or higher (negative log of the *p* value) have a high confidence of not being generated by random chance alone, and only pathways with scores of 10 or higher were considered in the present work.

**Supporting** **References**

1. Gatti G, Maresca G, Natoli M, Florenzano F, Nicolin A, et al. (2009) MYC prevents apoptosis and enhances endoreduplication induced by paclitaxel. PLoS One 4: e5442.

2. Schroeder A, Mueller O, Stocker S, Salowsky R, Leiber M, et al. (2006) The RIN: an RNA integrity number for assigning integrity values to RNA measurements. BMC Mol Biol 7: 3.

3. Natoli M, Leoni BD, D'Agnano I, D'Onofrio M, Brandi R, et al. (2011) Cell growing density affects the structural and functional properties of Caco-2 differentiated monolayer. J Cell Physiol 226: 1531-1543.

4. Huang dW, Sherman BT, Lempicki RA (2009) Systematic and integrative analysis of large gene lists using DAVID bioinformatics resources. Nat Protoc 4: 44-57.

5. Saeed AI, Bhagabati NK, Braisted JC, Liang W, Sharov V, et al. (2006) TM4 microarray software suite. Methods Enzymol 411: 134-193.

6. Del Bufalo D, Trisciuoglio D, Scarsella M, D'Amati G, Candiloro A, et al. (2004) Lonidamine causes inhibition of angiogenesis-related endothelial cell functions. Neoplasia 6: 513-522.

7. D'Aguanno S, D'Alessandro A, Pieroni L, Roveri A, Zaccarin M, et al. (2010) New insights into neuroblastoma cisplatin resistance: a comparative proteomic and meta-mining investigation. J Proteome Res .

8. Vissers JP, Langridge JI, Aerts JM (2007) Analysis and quantification of diagnostic serum markers and protein signatures for Gaucher disease. Mol Cell Proteomics 6: 755-766.

9. Chambery A, Colucci-D'Amato L, Vissers JP, Scarpella S, Langridge JI, et al. (2009) Proteomic profiling of proliferating and differentiated neural mes-c-myc A1 cell line from mouse embryonic mesencephalon by LC-MS. J Proteome Res 8: 227-238.

10. Takai D, Yagi Y, Wakazono K, Ohishi N, Morita Y, et al. (2001) Silencing of HTR1B and reduced expression of EDN1 in human lung cancers, revealed by methylation-sensitive representational difference analysis. Oncogene 20: 7505-7513.

11. Mostaan LV, Khorsandi MT, Sharifian SM, Shandiz FH, Mirashrafi F, et al. (2011) Correlation between E-cadherin and CD44 adhesion molecules expression and cervical lymph node metastasis in oral tongue SCC: Predictive significance or not. Pathol Res Pract 207: 448-451.

12. Bi X, Tong C, Dockendorff A, Bancroft L, Gallagher L, et al. (2008) Genetic deficiency of decorin causes intestinal tumor formation through disruption of intestinal cell maturation. Carcinogenesis 29: 1435-1440.

13. Yoshimizu T, Miroglio A, Ripoche MA, Gabory A, Vernucci M, et al. (2008) The H19 locus acts in vivo as a tumor suppressor. Proc Natl Acad Sci U S A 105: 12417-12422.

14. Nikolopoulos SN, Spengler BA, Kisselbach K, Evans AE, Biedler JL, et al. (2000) The human non-muscle alpha-actinin protein encoded by the ACTN4 gene suppresses tumorigenicity of human neuroblastoma cells. Oncogene 19: 380-386.

15. Salido M, Pijuan L, Martinez-Aviles L, Galvan AB, Canadas I, et al. (2011) Increased ALK gene copy number and amplification are frequent in non-small cell lung cancer. J Thorac Oncol 6: 21-27.

16. Choi HS, Yim SH, Xu HD, Jung SH, Shin SH, et al. (2010) Tropomyosin3 overexpression and a potential link to epithelial-mesenchymal transition in human hepatocellular carcinoma. BMC Cancer 10: 122.

17. Rickman DS, Tyagi R, Zhu XX, Bobek MP, Song S, et al. (2001) The gene for the axonal cell adhesion molecule TAX-1 is amplified and aberrantly expressed in malignant gliomas. Cancer Res 61: 2162-2168.

18. Li Y, Lu W, He X, Schwartz AL, Bu G (2004) LRP6 expression promotes cancer cell proliferation and tumorigenesis by altering beta-catenin subcellular distribution. Oncogene 23: 9129-9135.

19. Cook M, Yu XM, Chen H (2010) Notch in the development of thyroid C-cells and the treatment of medullary thyroid cancer. Am J Transl Res 2: 119-125.

20. Berger A, Santic R, Hauser-Kronberger C, Schilling FH, Kogner P, et al. (2005) Galanin and galanin receptors in human cancers. Neuropeptides 39: 353-359.

21. Ripka S, Riedel J, Neesse A, Griesmann H, Buchholz M, et al. (2010) Glutamate receptor GRIA3--target of CUX1 and mediator of tumor progression in pancreatic cancer. Neoplasia 12: 659-667.

22. Harper K, Arsenault D, Boulay-Jean S, Lauzier A, Lucien F, et al. (2010) Autotaxin promotes cancer invasion via the lysophosphatidic acid receptor 4: participation of the cyclic AMP/EPAC/Rac1 signaling pathway in invadopodia formation. Cancer Res 70: 4634-4643.
